# Xeniaphyllane-Type Diterpenoids from the Soft Coral *Sclerophytum humesi*: Resolving Absolute Configurations and Anti-Alzheimer’s Potential

**DOI:** 10.1021/acs.jnatprod.6c00535

**Published:** 2026-07-09

**Authors:** Phuong Vu Luu, Cuong-Quoc Nguyen, Huong Lien Ton-Nu, Thuy-Tien Thi Phan, Quoc-Dung Tran Huynh, Ngoc-Thac Pham, Huong-Giang Le, Lo-Yun Chen, Yu-Chia Chang, Jui-Hsin Su, Bo-Rong Peng, Kuei-Hung Lai

**Affiliations:** † Graduate Institute of Pharmacognosy, College of Pharmacy, 38032 Taipei Medical University, Taipei 110301, Taiwan; ‡ Department of Health Sciences, College of Natural Sciences, 95400 Can Tho University, Can Tho 94000, Vietnam; § Department of Chemistry, College of Natural Sciences, Can Tho University, Can Tho 94000, Vietnam; ∥ Graduate Institute of Biomedical Materials and Tissue Engineering, College of Biomedical Engineering, Taipei Medical University, Taipei 110301, Taiwan; ⊥ Institute of Pharmaceutical Education and Research, Binh Duong University, Thu Dau Mot, Binh Duong 820000, Vietnam; # Institute of Biological Chemistry, 38017 Academia Sinica, Taipei 115024, Taiwan; ¶ Department of Pharmacognosy and Traditional Pharmacy, School of Pharmacy, 249295 University of Medicine and Pharmacy at Ho Chi Minh City, Ho Chi Minh City 700000, Vietnam; ∇ PhD Program in Clinical Drug Development of Herbal Medicine, College of Pharmacy, Taipei Medical University, Taipei 110301, Taiwan; ○ Graduate Institute of Healthy Industry Technology, Center for Drug Research and Development, College of Human Ecology, 63113 Chang Gung University of Science and Technology, Taoyuan 333324, Taiwan; †† Department of Cosmetic Science, Chang Gung University of Science and Technology, Taoyuan City 33303, Taiwan; ‡‡ 63454 National Museum of Marine Biology & Aquarium, Pingtung 94450, Taiwan; §§ Department of Marine Biotechnology and Resources, National Sun Yat-sen University, Kaohsiung 804, Taiwan; ⊥⊥ Department of Biochemistry and Molecular Cell Biology, School of Medicine, College of Medicine, Taipei Medical University, Taipei 110301, Taiwan; ## Traditional Herbal Medicine Research Center, Taipei Medical University Hospital, Taipei 110301, Taiwan; ¶¶ PhD Program in Drug Discovery and Development Industry, College of Pharmacy, Taipei Medical University, Taipei 110301, Taiwan

## Abstract

A combined bioassay- and ^1^H NMR-guided isolation strategy led to the discovery of four new terpenoids, including three xeniaphyllane-type diterpenoids, sclerohumins P–R (**1–3**), and a norcaryophyllene-type, sclerophyllene A (**4**), from the soft coral *Sclerophytum humesi*. The structures and absolute configurations of these compounds were elucidated by comprehensive spectroscopic analysis, including NMR, HRESIMS, SOR, TDDFT-ECD, and DP4+ probability analysis. The stereogenic centers at C-15 in related xeniaphyllanes were proposed for the first time based on comparative SOR analysis. Compounds **1** and **2** possess a tricyclic 4/9-fused carbocyclic framework featuring an epoxide moiety, whereas compounds **3** and **4** exhibit related bicyclic scaffolds. A plausible biogenetic pathway originating from geranylgeranyl pyrophosphate (GGPP) was proposed to account for their structural diversity. Compounds **1** and **2** showed potent AChE inhibitory activity (IC_50_ = 1.7 and 4.4 μM, respectively), while being inactive against BChE and noncytotoxic toward normal cell lines (HEK293 and Vero). Mechanistic studies combining enzyme kinetics and molecular docking revealed that **1** and **2** act as mixed-type AChE inhibitors, with *K*_i_ values of 1.02 and 1.87 μM, respectively. These findings represent the first report of xeniaphyllane-type diterpenoids with potential for neurotherapeutic development.

## Graphical abstract

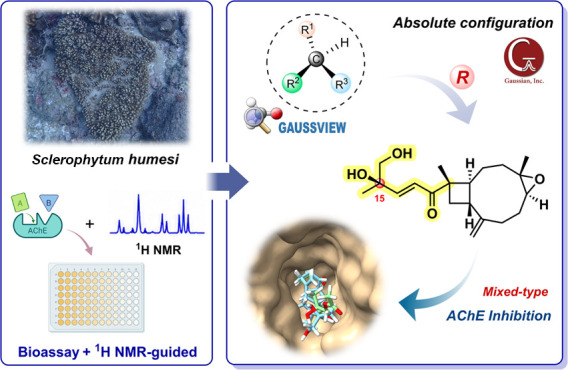

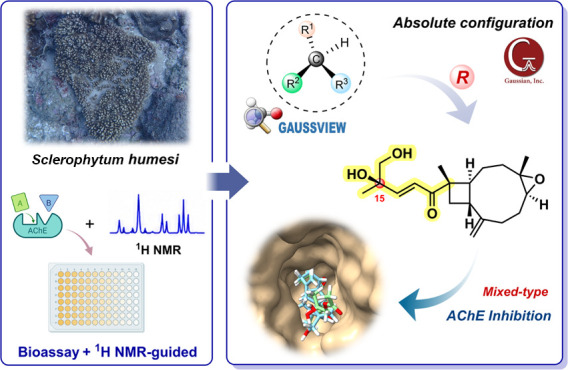

Alzheimer’s disease (AD) is the most prevalent neurodegenerative disorder and the leading cause of dementia in the elderly population worldwide.^1,2^ It is pathologically characterized by progressive cognitive decline accompanied by extensive neuronal loss, synaptic dysfunction, and brain atrophy. At the molecular level, AD is defined by the extracellular accumulation of amyloid-β (Aβ) plaques and the intracellular formation of neurofibrillary tangles composed of hyperphosphorylated tau protein.^3^ These pathological hallmarks collectively disrupt neuronal integrity and axonal transport, ultimately leading to neuronal dysfunction and cell death.^4^ Among the neuronal populations affected during AD progression, cholinergic neurons in the basal forebrain are particularly vulnerable. Their degeneration results in a marked reduction in acetylcholine (ACh), a neurotransmitter that plays a central role in learning, memory, and attention. This observation forms the basis of the cholinergic hypothesis, which proposes that cognitive impairment in AD is largely attributable to a deficit in central cholinergic neurotransmission. Consequently, strategies aimed at enhancing synaptic ACh levels have remained a cornerstone of symptomatic AD treatment.^4,5^

Acetylcholinesterase (AChE) and butyrylcholinesterase (BChE) are the two principal enzymes responsible for ACh hydrolysis in the nervous system. AChE is the dominant enzyme regulating ACh levels in healthy brains; however, its activity progressively decreases during AD progression. In contrast, BChE activity is maintained or even increased in the AD brain, particularly in advanced stages, where it becomes a major contributor to ACh degradation. Therefore, both selective and dual cholinesterase inhibition strategies have attracted considerable attention for the symptomatic management of AD, depending on the stage and pathological progression of the disease.^6,7^ Clinically used anti-Alzheimer’s drugs such as donepezil, galantamine, and rivastigmine exert their therapeutic effects primarily through reversible or pseudoirreversible inhibition of cholinesterases. However, their clinical efficacy remains limited, and these agents do not address the underlying neurodegenerative pathology of the disease.^8,9^

Marine organisms, particularly soft corals, are important sources of structurally diverse and bioactive secondary metabolites. Species of the genus *Sclerophytum* are known to produce pharmacologically relevant terpenoids, among which xeniaphyllane-type diterpenoids constitute a characteristic class featuring a bicyclo[7.2.0]­undecane core typically bearing a 4-methylpentyl side chain.^10^ These compounds were first reported in the 1970s from soft corals of the genus *Xenia* (family Xeniidae)^11–13^ and have since been identified predominantly in species of the genus *Sinularia* (family Alcyoniidae),^14–19^ highlighting their chemotaxonomic and pharmacological significance. Their flexible three-dimensional architectures may favor interactions with biological targets, making them attractive scaffolds for drug discovery.^20,21^ However, overlapping proton signals and side-chain stereogenic centers often hinder stereochemical assignment. To date, the relative configurations of several xeniaphyllane-type diterpenoids remain unresolved, including gibberosins G-J,^16^ gibberosins O–P,^17^ and gibberosin S.^17^ Accordingly, further studies on the isolation and stereochemical characterization of xeniaphyllanes are warranted.

As part of our ongoing investigation of secondary metabolites with potential biological activity from the soft coral *Sclerophytum humesi*, a bioassay- and ^1^H NMR-guided isolation approach was employed. This strategy led to the isolation of several terpenoids featuring a 4/9-fused carbocyclic skeleton (Figure 1). Given the urgent need for new cholinesterase inhibitors with improved efficacy, their inhibitory activities against AChE and BChE were evaluated. Herein, we report the isolation, structural elucidation, absolute configuration determination, and cholinesterase inhibitory activities of these 4/9-fused terpenoids. Notably, compounds **1** and **2** exhibited selective AChE inhibitory activity, providing new biological insights into xeniaphyllane-type diterpenoids and their potential relevance for cholinesterase-targeted drug discovery.

**Figure 1:**
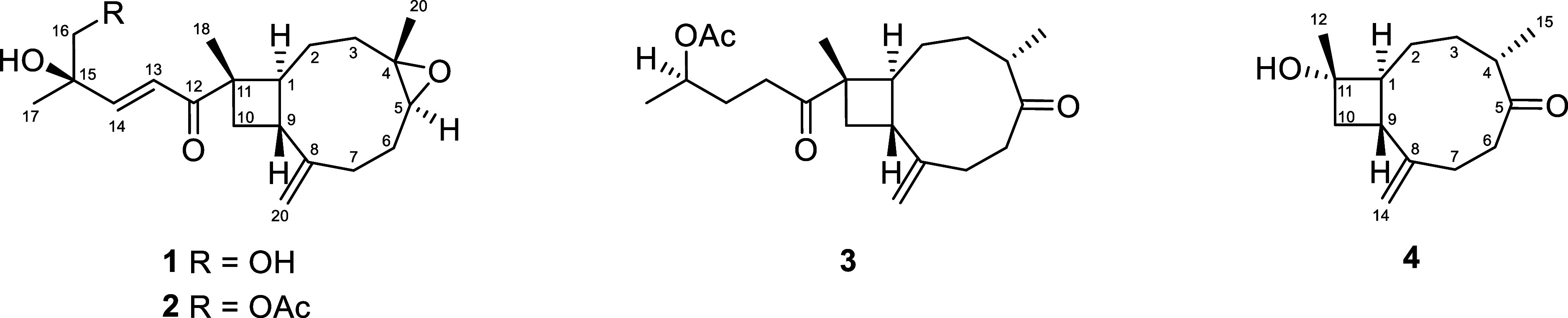
Structures of compounds **1–4** isolated from the soft coral *Sclerophytum humesi*.

## Result and Discussion

### Bioassay- and ^1^H NMR-Guided Isolation

The *Sclerophytum humesi* ethyl acetate extract (SH-EA) was subjected to medium-pressure liquid chromatography (MPLC) using a stepwise gradient of *n*-hexane (Hex) and ethyl acetate (EtOAc), with a flow rate of 20 mL/min over 180 min, gradually increasing from Hex/EtOAc (100:0) to (0:100), to yield 11 fractions (Fr.1-Fr.11). Bioassay-guided screening against acetylcholinesterase (AChE) revealed that Fr.7 exhibited the most potent inhibitory activity, with 95.8% inhibition at 50 μg/mL. Subsequent purification of Fr.7 by reversed-phase high-performance liquid chromatography (RP-HPLC) yielded four subfractions (Fr.7A-Fr.7D), which were further evaluated for AChE inhibitory activity. Among them, Fr.7D displayed dose-dependent inhibition, with inhibition rates of 56.8%, 75.2%, and 98.6% at concentrations of 3.125, 6.25, and 12.5 μg/mL, respectively (Figure 2A). Further ^1^H NMR analysis of Fr.7D revealed well-resolved signals corresponding to oxygenated methine proton at δ_H_ 2.95, olefinic protons at δ_H_ 6.54 and 6.97–6.90, as well as exomethylene protons at δ_H_ 4.93 and 5.02. These resonances are characteristic of xeniaphyllane-type diterpenoids, suggesting the presence of structurally interesting constituents (Figure 2B). Therefore, Fr.7D was selected for further purification by RP-HPLC, leading to the isolation of compounds **1**–**4** (Figure 1).

**Figure 2:**
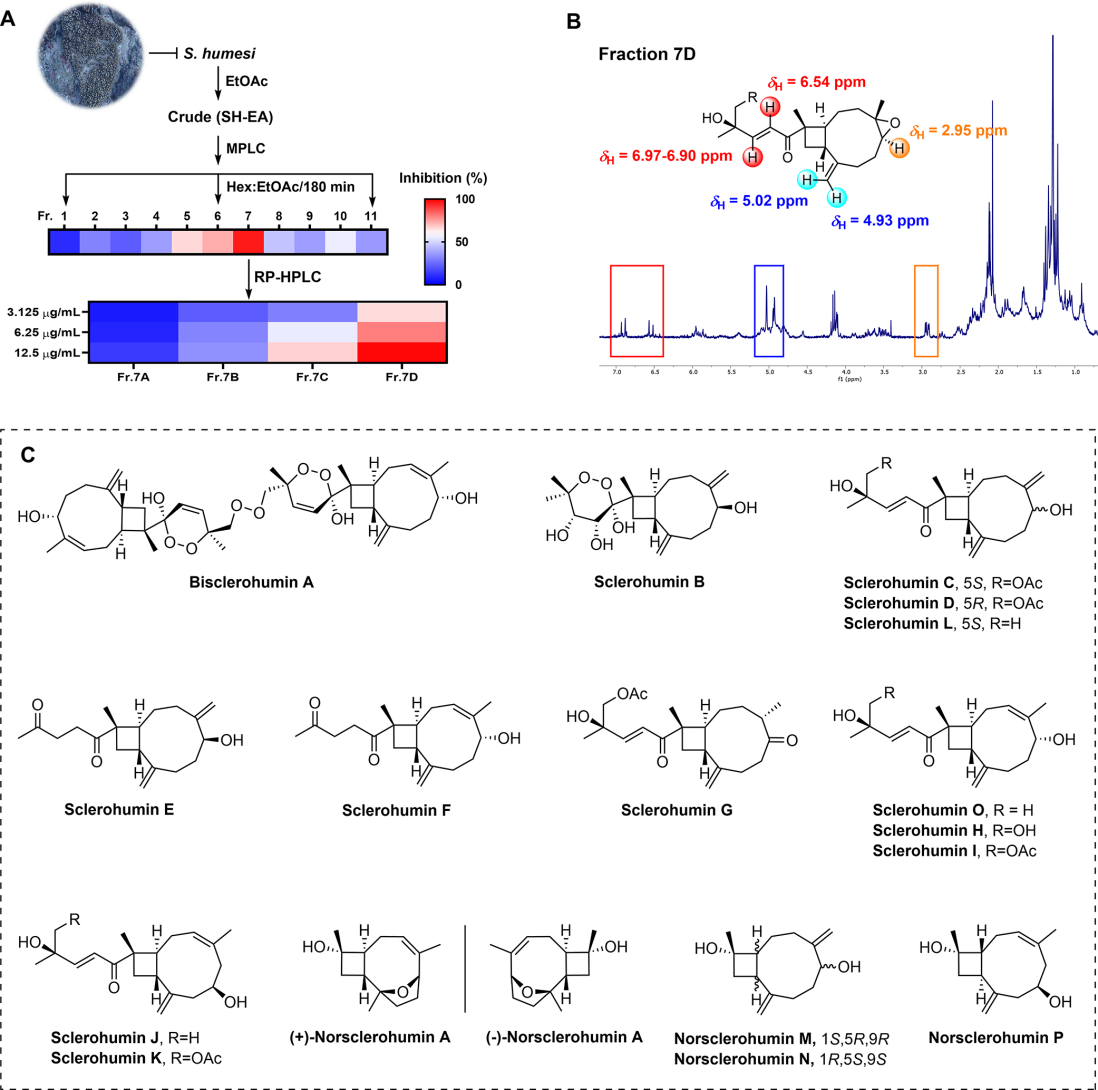
Bioactivity screening and structural insights of metabolites. (A) Acetylcholinesterase (AChE) inhibitory activity of fractions obtained from the soft coral *Sclerophytum humesi*. (B) ^1^H NMR spectrum of fraction 7D, showing characteristic proton signals indicative of a xeniaphyllane-type skeleton. (C) Reported compounds featuring a 4/9-fused ring system isolated from the soft coral *Sclerophytum humesi*.^10,20,21^

### Structural Elucidation

Sclerohumin P (**1**) was isolated as a colorless oil. HRESIMS analysis revealed a protonated molecule at *m*/*z* 335.2216 ([M + H]^+^; calculated for C_20_H_31_O_4_, *m*/*z* 335.2222), corresponding to six degrees of unsaturation (double-bond equivalents, DBEs). The IR spectrum exhibited characteristic absorptions for hydroxy groups (3412 cm^–1^) and conjugated carbonyl functionalities (1624 cm^–1^). The ^1^H NMR spectrum (Table 1) displayed resonances attributable to three tertiary methyl groups at δ_H_ 1.22 (3H, *s*), 1.28 brd (*J* = 1.1 Hz, ^4^ *J*_17,14_), 1.33 (3H, *s*), two exomethylene protons at δ_H_ 4.93 (1H, *d*, *J* = 1.5 Hz), 5.02 (1H, *d*, *J* = 1.5 Hz), three methine protons at δ_H_ 2.45 (1H, *td*, *J* = 9.8, 3.9 Hz), 2.83 (1H, *td*, *J* = 10.1, 8.6 Hz), 2.95 (1H, *ddd*, *J* = 10.7, 4.1, 1.8 Hz), and two olefinic protons at δ_H_ 6.54 (1H, *d*, *J* = 15.5 Hz), and 6.97 (1H, *d*, *J* = 15.5 Hz). Analysis of the ^13^C NMR, DEPT, and HSQC spectra (Table 1) revealed 20 carbon signals, including one conjugated carbonyl carbon (δ_C_ 205.8), a terminal double bond (δ_C_ 114.5 and 152.5), two olefinic (δ_C_ 123.7 and 152.8), three sp^3^ quaternary (δ_C_ 48.8, 61.5 and 74.3), three sp^3^ methines (δ_C_ 46.6, 48.4, and 65.3), six sp^3^ methylenes (δ_C_ 29.0, 30.3, 31.0, 36.2, 39.6, and 70.2), and three methyl (δ_C_ 17.2, 17.4, and 24.3). The presence of two carbon–carbon double bonds and one conjugated carbonyl group accounted for three out of the six DBEs, indicating that **1** possesses a tricyclic system. The planar structure of compound **1** was identical to that of the previously reported compound gibberosin G in 2007.^16^ However, to date, the relative configuration (RC) at the C-15 stereogenic center in the side chain has not yet been assigned.

**Table 1:** ^1^H and ^13^C NMR Data of Compounds **1**–**4**

no.		1^a^		2^a^		3^a^		4^a^
	δ_H_ (*J* in Hz)	δ_C_	δ_H_ (*J* in Hz)	δ_C_	δ_H_ (*J* in Hz)	δ_C_	δ_H_ (*J* in Hz)	δ_C_
1	2.45, *td* (9.8, 3.9)	46.6, CH	2.45, *td* (9.8, 3.3)	46.6, CH	1.94, *m*	47.5, CH	1.66, overlapped	56.6, CH
2α	1.88, *m*	29.0, CH_2_	1.86, *m*	29.0, CH_2_	1.76, *m*	28.5, CH_2_	1.28, *m*	27.8, CH_2_
2β	1.66, *m*		1.66, *m*		1.44, *dddd* (13.9, 10.7, 8.4, 2.5)		1.81, *m*	
3α	1.08, *tdd* (12.6, 4.9, 2.3)	39.6, CH_2_	1.07, *td* (12.2, 5.1)	39.7, CH_2_	1.68, *m*	31.3, CH_2_	1.66, overlapped	32.4, CH_2_
3β	2.06, *m*		2.08, *m*		1.89, *m*		1.82, *m*	
4		61.5, qC		61.5, qC	2.63, overlapped	48.5, CH	2.61, *m*	48.7, CH
5	2.95, *ddd* (10.7, 4.1, 1.8)	65.3, CH	2.94, *dd* (10.6, 3.9)	65.3, CH		219.8, qC		219.5, qC
6α	2.22, *m*	31.0, CH_2_	2.22, *m*	31.0, CH_2_	2.46, overlapped	43.7, CH_2_	2.48, *m*	43.6, CH_2_
6β	1.34, *m*		1.34, *m*		2.63, overlapped		2.65, *m*	
7α	2.33, *ddd* (12.6, 8.1, 3.3)	30.3, CH_2_	2.33, *ddd* (12.6, 8.2, 3.5)	30.3, CH_2_	2.46, overlapped	32.7, CH_2_	2.51, *m*	32.7, CH_2_
7β	2.18, *m*		2.18, *m*				2.44, *m*	
8		152.5, qC		152.5, qC		153.2, qC		153.1, qC
9	2.83, *td* (10.1, 8.6)	48.4, CH	2.83, *td* (10.1, 8.6)	48.3, CH	2.54, *m*	43.5, CH	1.94, *m*	41.2, CH
10α	2.15, *m*	36.2, CH_2_	2.14, *m*	36.2, CH_2_	1.67, *m*	34.7, CH_2_	1.82, *m*	41.8, CH_2_
10β	1.84, *m*		1.84, *m*		2.03, *m*		1.89, *dd* (10.4, 7.6)	
11		48.8, qC		48.8, qC		48.6, qC		71.2, qC
12		205.8, qC		205.5, qC		215.6, qC	1.13, *s*	21.5, CH_3_
13	6.54, *d* (15.5)	123.7, CH	6.54, dd (15.5, 0.9)	124.2, CH	2.41, *m*	33.5, CH_2_		
					2.50, *m*			
14	6.97, *d* (15.5)	152.8, CH	6.90, dd (15.5, 1.0)	151.2, CH	1.73, *m*	30.6, CH_2_	4.93, *d* (1.5)	112.5, CH_2_
					1.82, *m*		4.94, *d* (1.4)	
15		74.3, qC		72.7, qC	4.87, *m*	71.6, CH	1.02, *d* (6.8)	17.3, CH_3_
16	3.44, *dd* (3.9, 1.3)	70.2, CH_2_	4.01, *dd* (11.1, 1.2)	71.1, CH_2_				
			4.07, *dd* (11.1, 5.0)					
17	1.28, *brd* (1.1)	24.3, CH_3_	1.32, *brd* (1.4)	24.7, CH_3_	1.22, *d* (6.3)	20.3, CH_3_		
18	1.33, *s*	17.2, CH_3_	1.33, *s*	17.2, CH_3_	1.21, *s*	17.2, CH_3_		
19	4.93, *d* (1.5)	114.5, CH_2_	4.94, *d* (1.5)	114.6, CH_2_	4.93, *s*	113.0, CH_2_		
	5.02, *d* (1.5)		5.03, *d* (1.5)					
20	1.22, *s*	17.4, CH_3_	1.23, *s*	17.4, CH_3_	1.03, *d* (6.8)	16.7, CH_3_		
OAc				172.4, qC		172.7, qC		
			2.05, *s*	20.7, CH_3_	2.03, *s*	21.2, CH_3_		

a Spectra recorded in CD_3_OD at 600 MHz (^1^H NMR) and 150 MHz (^13^C NMR).

In this study, comprehensive spectroscopic analysis combined with quantum chemical calculations was employed to address the unresolved stereochemical assignments. The NOESY spectroscopic data of compound **1** were fully consistent with those reported for gibberosin G,^16^ indicating that the RC of the conserved bicyclic framework is retained. Specifically, key NOE correlations between H_3_-18 (δ_H_ 1.33)/H-9 (δ_H_ 2.83), H-9/H-2β (δ_H_ 1.66), and H-9/H_3_-20 (δ_H_ 1.22) suggested that H_3_-18, H_3_-20, and H-9 are cofacial and located on the same face of the molecular framework (assigned as the β-face). In contrast, correlations between H-1 (δ_H_ 2.45)/H-5 (δ_H_ 2.95) and H-1/H-3α (δ_H_ 1.08), indicated that H-1 and H-5 are cofacial and located on the opposite face (assigned as the α-face).^10,19,22^ However, considering the inherent conformational flexibility of the nine-membered ring system together with the weak NOE correlations associated with the side chain attached at C-11 and the stereogenic center at C-15, the RC could not be unambiguously established based solely on the experimental NMR data. Therefore, the RC was unambiguously determined using GIAO-NMR chemical shift calculations performed on all low-energy conformers, with Boltzmann-weighted averaging applied to account for the conformational flexibility (see the Supporting Information).^10,20,22–24^ Eight possible stereoisomers, (5S*,11S*,15S*)-**1a**, (5S*,11S*,15R*)-**1b**, (5S*,11R*,15S*)-**1c**, (5S*,11R*,15R*)-**1d**, (5R*,11S*,15S*)-**1e**, (5R*,11S*,15R*)-**1f**, (5R*,11R*,15S*)-**1g**, (5R*,11R*,15R*)-**1h** were evaluated. The DP4+ analysis unambiguously identified (5S*,11S*,15R*)-**1b** as the most probable structure with 100% probability, which was further supported by a strong correlation between calculated and experimental ^13^C NMR data (*R* ^2^ = 0.9978) (see the Supporting Information).

Compound **1** exhibited a specific optical rotation (SOR) of [α]_D_^25^ −15.2 (*c* 0.3, CHCl_3_), which is opposite in sign to that reported for gibberosin G measured under identical conditions.^16^ SOR calculations were performed for the 15*S* and 15*R* configurations, yielding SOR values of +32.8 and −22.4, respectively (Figure 5A). The sign inversion indicates that the difference in SOR arises from stereochemical variation at C-15, suggesting compound **1** as the 15*R*, while the C-15 configuration of gibberosin G was assigned as 15*S*. The absolute configuration (AC) of compound **1** was determined by TDDFT-ECD calculations.^10,20–23^ The calculated ECD spectrum for the (15*R*)-isomer showed excellent agreement with the experimental curve, whereas the (15*S*)-isomer exhibited opposite Cotton effects (CE) in the 220–250 nm region (Figure 5C). Notably, a single stereogenic center at C-15 can markedly affect the ECD spectrum.^19,25^ These results establish the AC of compound **1** as 1*S*, 4*S*, 5*S*, 9*R*, 11*S*, 15*R*, consistent with its SOR, and identify it as the C-15 epimer of gibberosin G.

**Figure 3:**
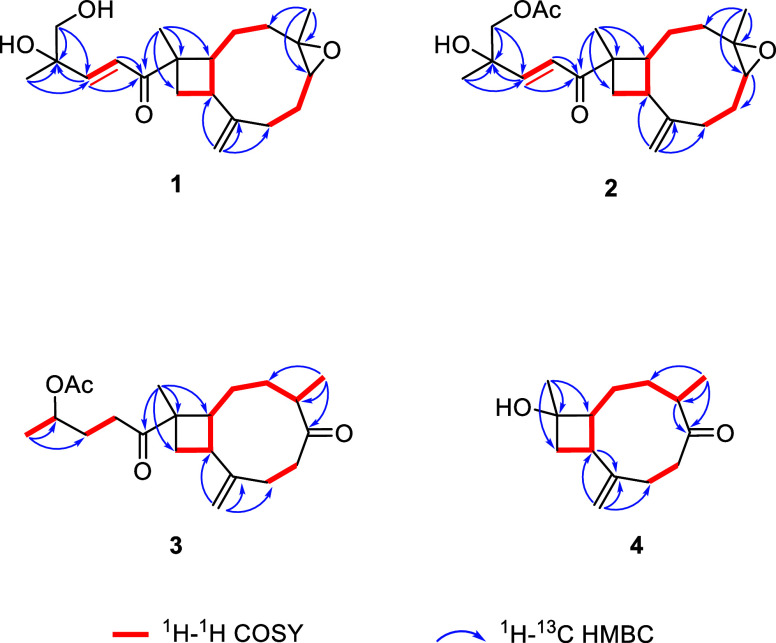
Selected COSY (red bold) and HMBC (blue arrows) correlations observed in compounds **1–4**.

**Figure 4:**
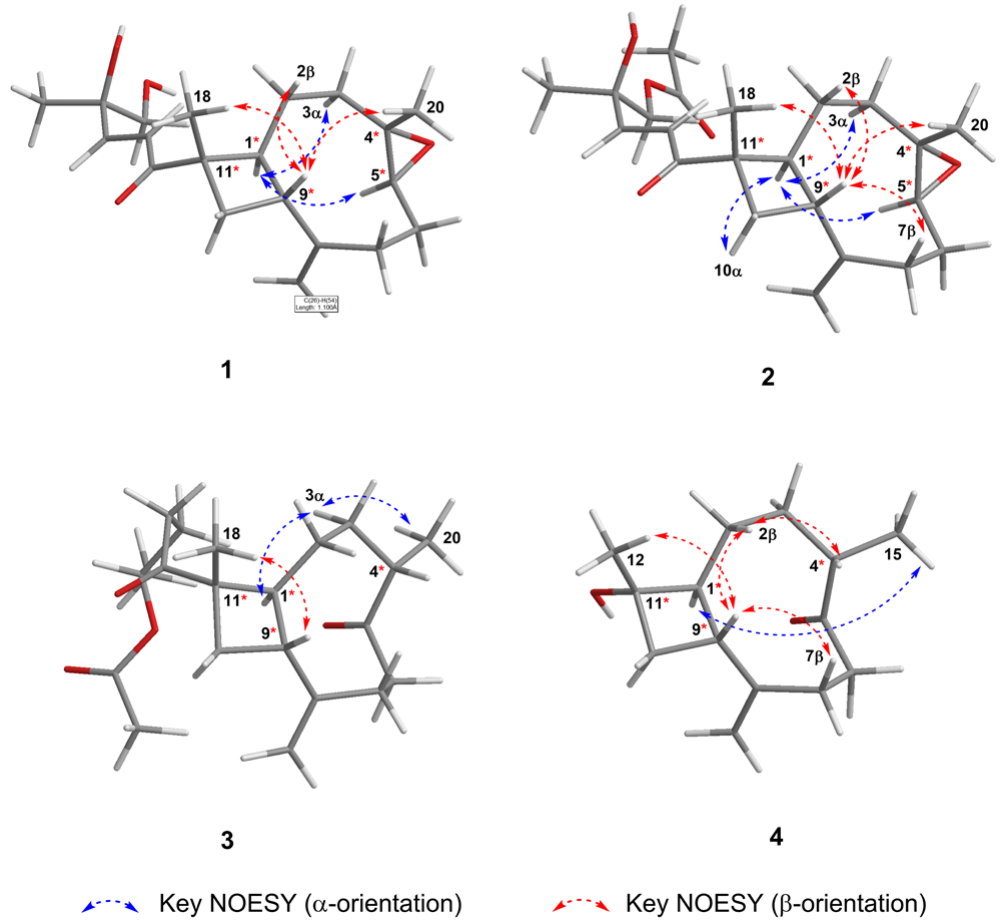
Key NOESY (dashed arrows) correlations observed in compounds **1–4**.

**Figure 5:**
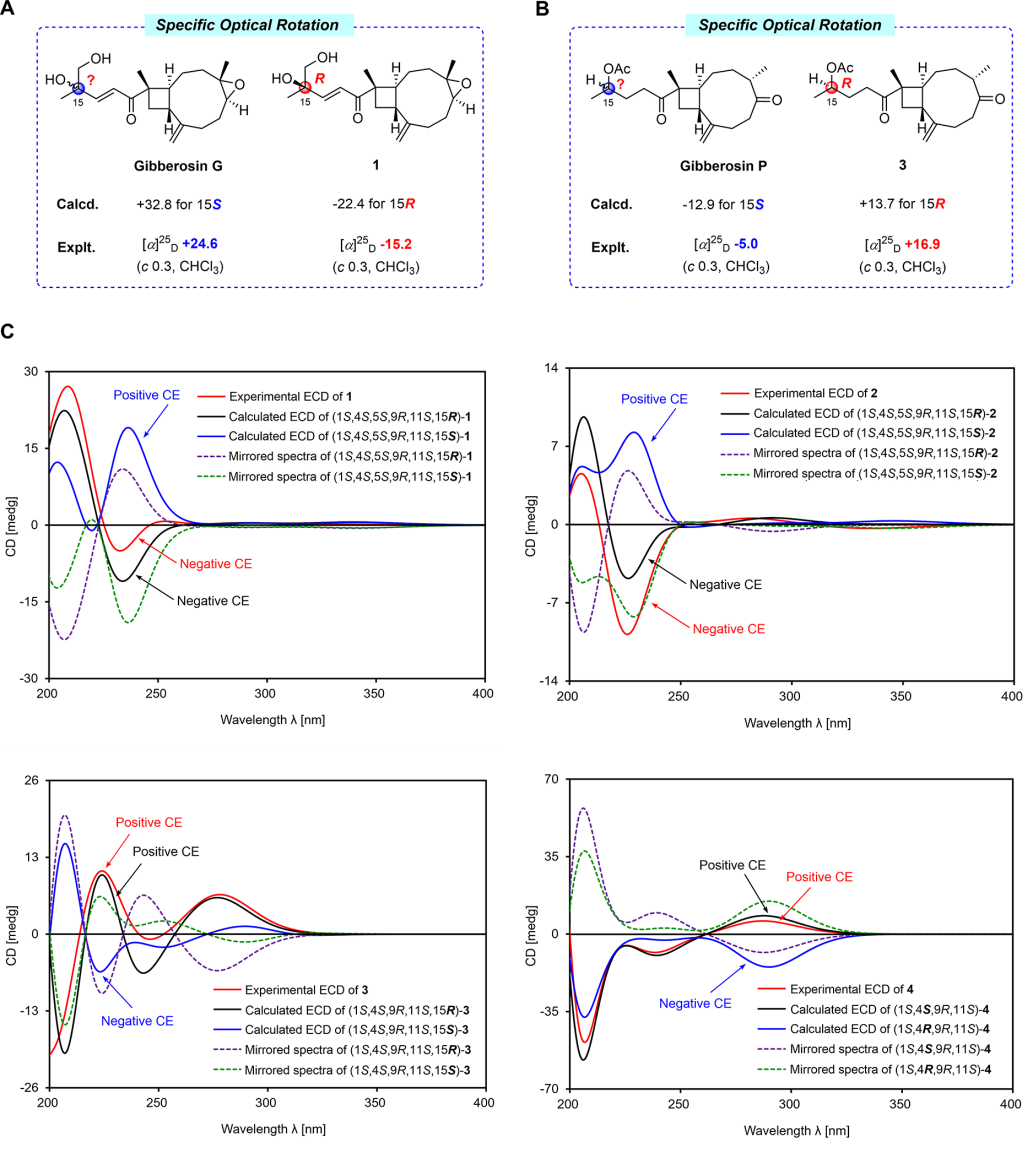
Comparison of specific optical rotation (SOR) and calculated TDDFT-ECD spectra for assignment of absolute configurations. (A) SOR values of compound **1** and its corresponding 15*S*/15*R*-configured diastereomers. (B) SOR values of compound **3** and its corresponding 15*S*/15*R*-configured diastereomers. (C) Comparison of experimental and calculated TDDFT-ECD spectra of compounds **1–4**.

Sclerohumin Q (**2**) was obtained as a colorless oil. HRESIMS analysis revealed a protonated molecule at *m/z* 377.2321 ([M + H]^+^; calculated for C_22_H_33_O_5_, *m/z* 377.2328). Compared with **1**, compound **2** exhibited an increase of C_2_H_2_O (42 mDa) in its molecular formula. The IR spectrum showed characteristic absorption bands at 3344, 1683, and 1625 cm^–1^, attributable to hydroxy, ester carbonyl, and conjugated carbonyl group, respectively. The NMR data of compound **2** (Table 1) closely resembled those of compound **1**, except for a deshielded CH_2_–16, as evidenced by downfield shifts (Δδ_H_ + 0.57, +0.63; Δδ_C_ + 0.9), along with the appearance of additional proton and carbon signals [δ_H_ 2.05 (3H, *d*, *J* = 1.1 Hz); δ_C_ 172.4, 20.7]. These observations indicate that compound **2** is the 16-*O*-acetyl derivative of compound **1** (Figure 1). Accordingly, the C-15 resonance in **2** (Δδ_C_ −1.6) was observed to be more shielded than that in **1**, which is attributed to the *γ*-gauche effect induced by the acetyl group at C-16.^26–28^

Based on the close chemical shifts of the tricyclic framework and the key NOE correlations observed, consistent with those of **1**, the RC of C-1, C-4, C-5, C-9, and C-11 in **2** was assigned as 1S*, 4S*, 5S*, 9R*, 11S* (Figure 4). The C-15 configuration was assigned by GIAO-NMR calculations,^20,21^ giving 100% DP4+ probability for the 15R* isomer according to DP4+ analysis (Figure 6B,C), with mean absolute error (MAE) values of 0.22 (^1^H) and 3.59 (^13^C) (Figure 6D). The planar structure and the RC of **2** was identical to that of the previously reported compound gibberosin H.^16^ However, similar to compound **1**, the configuration at C-15 was not fully established in the original report. Therefore, SOR calculations were performed to further confirm the stereochemical assignments of both compound **2** and gibberosin H (see the Supporting Information). The AC of **2** was determined by comparison of its calculated and experimental ECD spectra. The calculated TDDFT-ECD spectrum of the (1*S*, 4*S*, 5*S*, 9*R*, 11*S*, 15*R*)-**2** stereoisomer showed good agreement with the experimental spectrum (Figure 5C), establishing the AC of **2** as 1*S*, 4*S*, 5*S*, 9*R*, 11*S*, 15*R* and identifying it as the C-15 epimer of gibberosin H.

**Figure 6:**
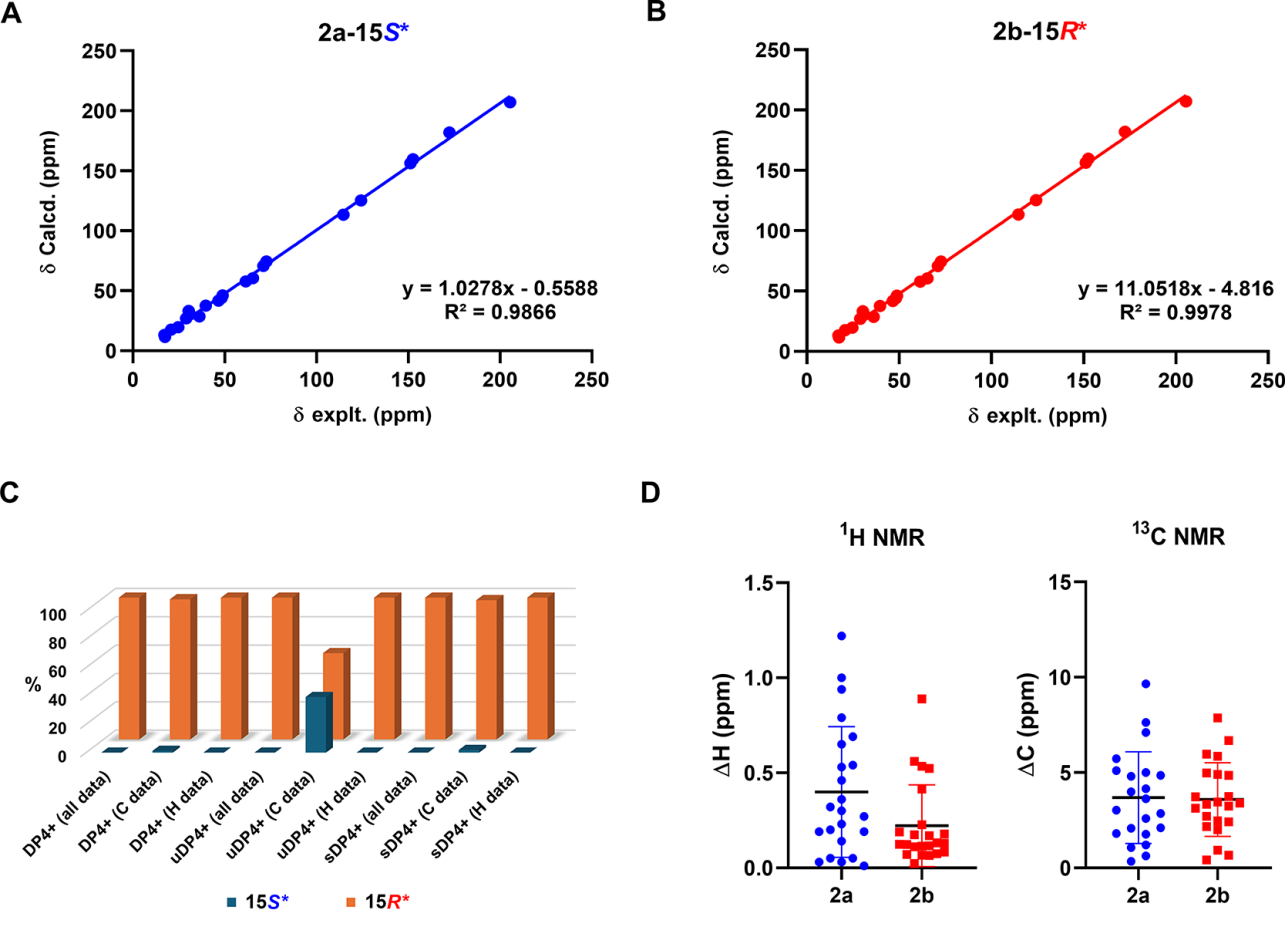
Computational and statistical analysis for stereochemical assignment of compound **2**. (A) Linear correlations of the calculated isomers **2a-**15S* with the experimentally observed ^13^C NMR chemical shifts. (B) Linear correlations of the calculated isomers **2b-**15R* with the experimentally observed ^13^C NMR chemical shifts. (C) DP4+ results obtained using experimental data of compound **2** *versus* isomers **2a-**15S* and **2b-**15R*. (D) Comparison of the corrected calculated NMR chemical shifts of *versus* isomers **2a-**15S* and **2b-**15R* with the experimental shifts of compound **2**.

Sclerohumin R (**3**) was isolated as a colorless oil. HRESIMS analysis revealed a protonated molecule at *m/z* 349.2375 ([M + H]^+^; calculated for C_21_H_33_O_4_, *m/z* 349.2379), corresponding to six DBEs. The IR spectrum exhibited characteristic absorption bands attributable to ketone (1732 cm^–1^) and ester carbonyl (1699 cm^–1^) functionalities. The NMR data of compound **3** (Table 1) indicated a bicyclic framework similar to that of the diterpenoid sclerohumin G,^10^ except that the side chain of compound **3** contains a ketone carbonyl group at δ_C_ 215.6, two methylene groups at [δ_H_ 2.41 (1H, *m*), 2.50 (1H, *m*); δ_C_ 33.5] and [δ_H_ 1.73 (1H, *m*), 1.82 (1H, *m*); δ_C_ 30.6], as well as an oxygenated methine at [δ_H_ 4.87 (1H, *m*); δ_C_ 71.6]. This assignment was further supported by ^1^H–^1^H COSY correlations between H_2_-13 (δ_H_ 2.41, 2.50)/H-14 (δ_H_ 1.73, 1.82), and H_3_-17 (δ_H_ 1.22)/H-15 (δ_H_ 4.87), along with key HMBC correlations from H_3_-17 to carbons C-14/C-15, and from H_3_-18 to C-12 (Figure 3).

The RC of compound **3** was determined based on NOESY experiments and by comparison with those of related compounds. Key NOE correlations between H-1 (δ_H_ 1.94)/H-3α (δ_H_ 1.68) and H-3α/H_3_-20 (δ_H_ 1.03), indicated that H-1 and H_3_-20 are α-face. In contrast, H_3_-18 (δ_H_ 1.21)/H-9 (δ_H_ 2.54), suggested that H_3_-18 and H-9 are β-face. However, unambiguous assignment of the RC of compound **3** based solely on NOE correlations proved difficult. The stereogenic center at C-15 is located on a flexible side chain and does not show definitive NOE interactions with the bicyclic core. In addition, the overlap of the H-4 (δ_H_ 2.63) and H-6β (δ_H_ 2.63) signals further complicated the NOE analysis. Moreover, C-4 is associated with a methyl-bearing stereogenic center embedded within the conformationally flexible nine-membered ring system, which may significantly affect the overall conformational preferences of the molecule. Therefore, GIAO-NMR chemical shift calculations were employed to assist in the configurational assignment. Accordingly, eight possible stereoisomers, namely (4S*, 11S*, 15S*)-**3a**, (4S*, 11S*, 15R*)-**3b**, (4S*, 11R*, 15S*)-**3c**, (4S*, 11R*, 15R*)-**3d**, (4R*, 11S*, 15S*)-**3e**, (4R*, 11S*, 15R*)-**3f**, (4R*, 11R*, 15S*)-**3g**, (4R*, 11R*, 15R*)-**3h** were generated for GIAO-NMR calculations. The isomer (4S*, 11S*, 15R*)-**3b** configuration showed an excellent agreement with the experimental data, achieving 100% probability by DP4+ analysis (see the Supporting Information). The RC at C-1, C-4, C-9, and C-11 of compound **3** matched those of gibberosin P.^17^ However, compound **3** exhibited an opposite sign of SOR compared to gibberosin P, indicating a stereochemical difference at C-15. This was supported by SOR calculations (see the Supporting Information), suggesting that **3** is the C-15 epimer of gibberosin P. Finally, the AC of **3** was further established as 1*S*, 4*S*, 9*R*, 11*S*, 15*R* based on ECD analysis (Figure 5C).

Sclerophyllene A (**4**) was obtained as a colorless oil. HRESIMS analysis revealed a protonated molecule at *m/z* 223.1692 ([M + H]^+^; calculated for C_14_H_23_O_2_, *m/z* 223.1698), corresponding to four DBEs. The IR spectrum displayed absorption bands corresponding to a hydroxy (3271 cm^–1^) and ketone carbonyl (1641 cm^–1^) functionality. Detailed analysis of the NMR data (Table 1) revealed that compound **4** shares the same bicyclic 4/9-fused ring system as **3**, except for the absence of the side chain, which is replaced by a hydroxy group at C-11 (δ_C_ 72.1) (Figure 1). This is evidenced by highly similar chemical shift, key COSY and HMBC correlations within the core skeleton (Figure 3). Therefore, the planar structure of the 4/9-fused ring system in **4** was established to be identical to that of **3**. The RC of compound **4** was established on the basis of NOE correlations. However, unambiguous assignment proved challenging due to signal overlap between H-1 (δ_H_ 1.66) and H-3β (δ_H_ 1.66). Accordingly, four possible stereoisomers, (1S*, 4S*)-**4a**, (1S*, 4R*)-**4b**, (1R*, 4S*)-**4c**, (1R*, 4R*)-**4d**, were generated for GIAO-NMR calculations. The **4a** stereoisomer showed excellent agreement with the experimental data, with a DP4+ probability of 100% (see the Supporting Information). This result supports the assignment of the RC of compound **4** as 1S*, 4S*, 9R*, 11S*. Finally, the calculated TDDFT-ECD spectrum of the (1*S*, 4*S*, 9*R*, 11*S*)-**4** stereoisomer exhibited strong agreement with the experimental spectrum (Figure 5C), thereby confirming the AC as 1*S*, 4*S*, 9*R*, 11*S*.

#### Proposed Biosynthetic Pathway

The biosynthesis originates from geranylgeranyl pyrophosphate (GGPP), in which the departure of the pyrophosphate (-OPP) group, catalyzed by terpene synthase, initiates carbocation formation (Scheme 1).^29^ Based on experimentally characterized xeniaphyllene synthases and related mechanistic studies, the cyclization likely proceeds through an initial 1,11-cyclization followed by a concerted 2,10-cyclization to generate a carbocation intermediate, ultimately affording the fused-ring xeniaphyllene skeleton (**i–v**), consistent with reported coral diterpene biosynthesis.^29–33^ Subsequent oxidative transformations are proposed to occur through enzyme-mediated or nonenzymatic processes, including epoxidation and allylic oxidation. In particular, the conversion from (**vi**) to (**viii**) is proposed to proceed through hydroxylation at C-15 accompanied by double-bond migration to generate intermediate (**vii**), followed by allylic oxidation to afford the α,β-unsaturated ketone moiety in (**viii**). Further hydroxylation and acetylation steps afford compounds **1** and **2**, respectively.^32^ Subsequent hydroxylation and acetylation steps lead to the formation of compounds **1** and **2**, respectively. Compound **3** may arise from a common xeniaphyllene intermediate (**v**), followed by sequential reduction, acetylation, and oxidation to yield the final structure. Collectively, these enzyme-catalyzed cyclization, rearrangement, and oxidative transformations from a common GGPP-derived precursor account for the structural diversity observed within the xeniaphyllane diterpenoid family.

**Scheme 1:**
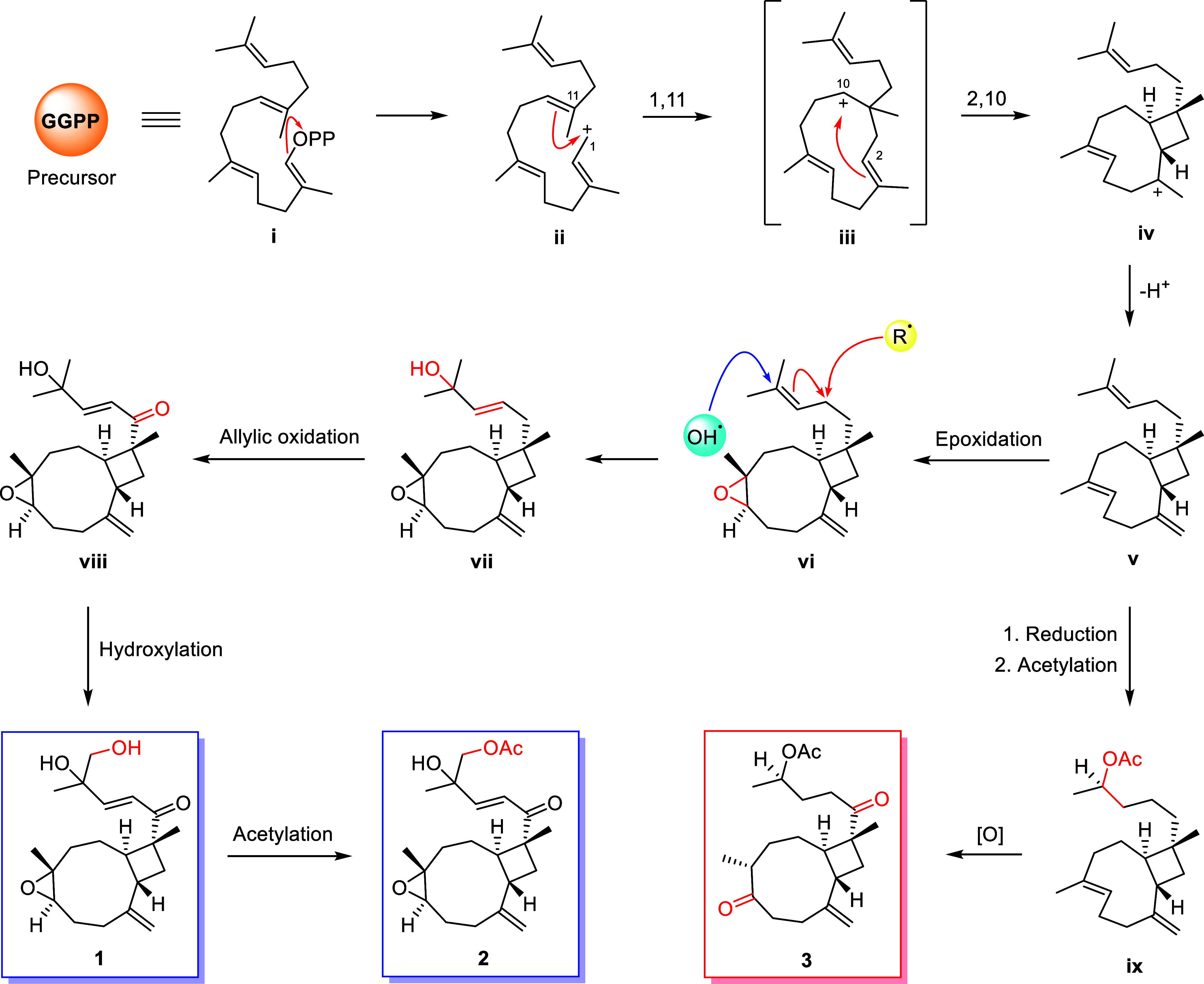
Proposed Biogenetic Pathway for Compounds 1–3

### Bioactivity Characterization

The anti-Alzheimer’s potential of compounds **1–4** was evaluated based on their inhibitory activities against AChE and BChE, along with cytotoxicity assessment in normal cell lines (HEK293 and Vero). Compounds **1** and **2** exhibited potent AChE inhibitory activity, with IC_50_ values of 1.7 ± 0.2 and 4.4 ± 0.5 μM, respectively, comparable to the positive control galantamine (IC_50_ = 3.9 ± 0.1 μM). In contrast, compounds **3** and **4** showed weaker activity against AChE (IC_50_ > 10 μM), indicating a significant reduction in inhibitory potency (Figure 7, Table 2). All compounds were inactive against BChE, with IC_50_ values greater than 70 μM, indicating selectivity for AChE over BChE. Compounds **1** and **2** exhibited selectivity indices (SI) of 43.3 and 18.7, respectively. Importantly, none of the tested compounds exhibited cytotoxic effects against HEK293 and Vero cell lines (CC_50_ > 50 μM), indicating a favorable safety profile in normal cells. Overall, compounds **1** and **2** can be considered promising AChE inhibitors with selective activity and low cytotoxicity, while compounds **3** and **4** are regarded as inactive (IC_50_ > 10 μM) under the tested conditions.

**Figure 7:**
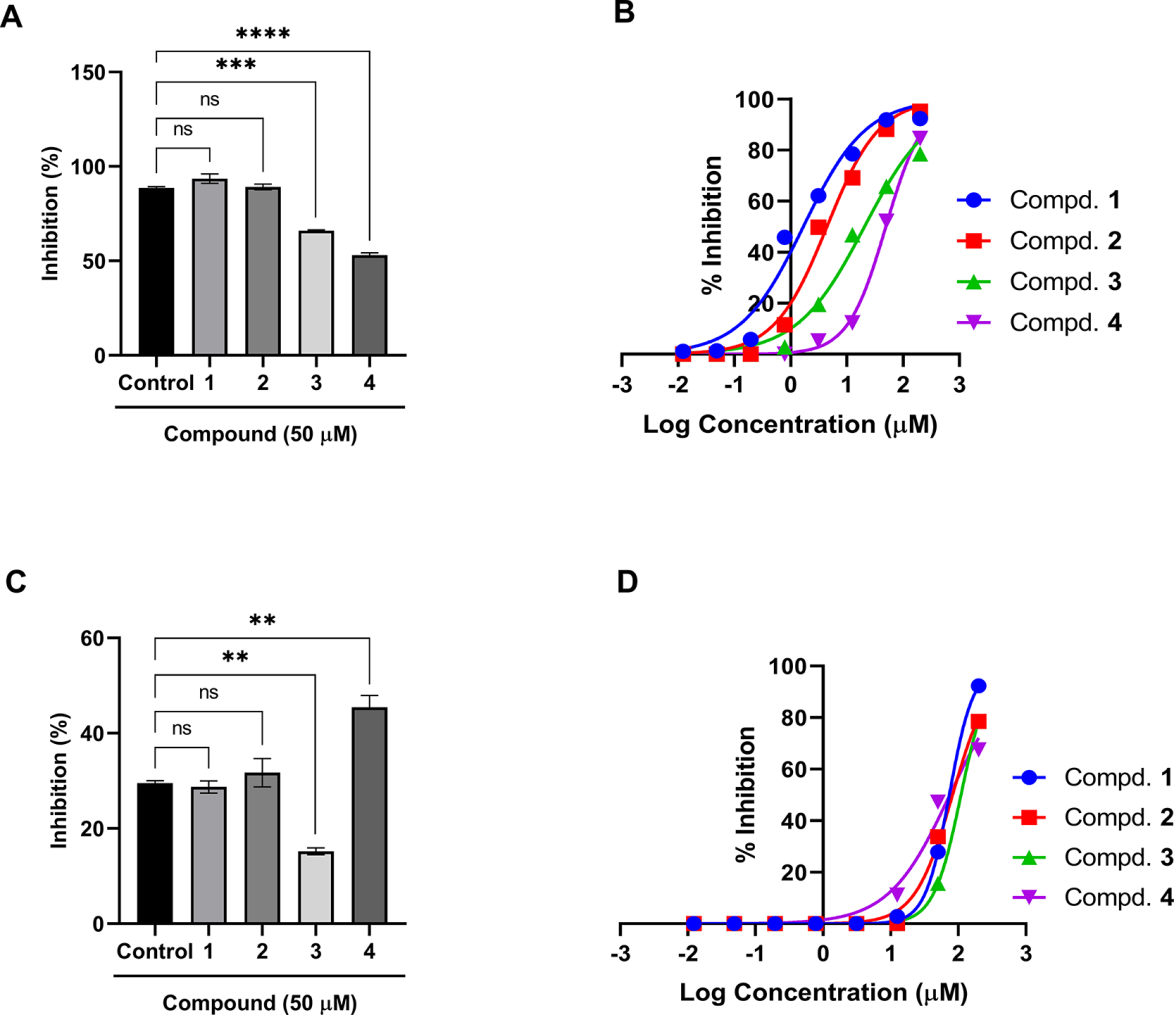
AChE and BChE inhibitory potential of compounds **1–4** toward Alzheimer’s disease. (A) The percentage inhibition and (B) dose–response curves of AChE at 50 μM for the tested compounds **1–4**. (C) The percentage inhibition and (D) dose–response curves of BChE at 50 μM for the tested compounds **1–4**. Data were expressed as mean values ±SD, n = 3. *p < 0.05, **p < 0.01, ***p < 0.001, ****p < 0.0001.

**Table 2:** Anti-Alzheimer’s Enzyme Inhibitory Activities and Cytotoxicity Profiles in Normal Cells Lines of Compounds **1–4**

sample	IC_50_ (μM)^a^		SI^c^	CC_50_ (μM)^b^	
	AChE	BChE		HEK293	Vero
**1**	1.7 ± 0.2	73.6 ± 2.5	43.3	>50	>50
**2**	4.4 ± 0.5	82.2 ± 1.7	18.7	>50	>50
**3**	20.6 ± 0.9	109.5 ± 1.4	5.3	>50	>50
**4**	49.2 ± 1.4	74.8 ± 0.8	1.5	>50	>50
Galantamine^d^	3.9 ± 0.1	87.8 ± 0.7	22.5	NT^e^	NT^e^
Quercetin^d^	NT^e^	NT^e^	-	>50	>50

a Half-maximal inhibitory concentration.

b Half-maximal cytotoxic concentration. IC_50_ and CC_50_ values were determined by regression analyses and expressed as the means ± SD of three replicate determinations.

c SI is the AChE selectivity index defined as IC_50_ BChE/IC_50_ AChE affinity ratio.

d Positive control.

e NT: not tested.

To clarify the molecular basis underlying the inhibitory activity of compounds **1** and **2**, a combination of enzyme kinetic and molecular docking studies was performed. Using a Lineweaver–Burk plot to analyze AChE inhibition by two phytochemicals, the study observed that the trendlines corresponding to different inhibitor concentrations intersected the *Y*-axis (1/*V*_max_) at distinct points, indicating variations of *V*_max_ (Figure 8A,B). Enzyme kinetics also revealed that *K*_m_ increased and *V*_max_ decreased with rising inhibitor concentrations, leading to the conclusion that both compounds inhibit AChE through a mixed inhibition mechanism. The corresponding secondary plots afforded *K*_i_ values of 1.02 μM for compound **1** and 1.87 μM for compound **2**, suggesting a stronger affinity of compound **1** toward the AchE complex. These results indicate that compounds **1** and **2** can bind to both the catalytic active site and peripheral regions of AChE.

**Figure 8:**
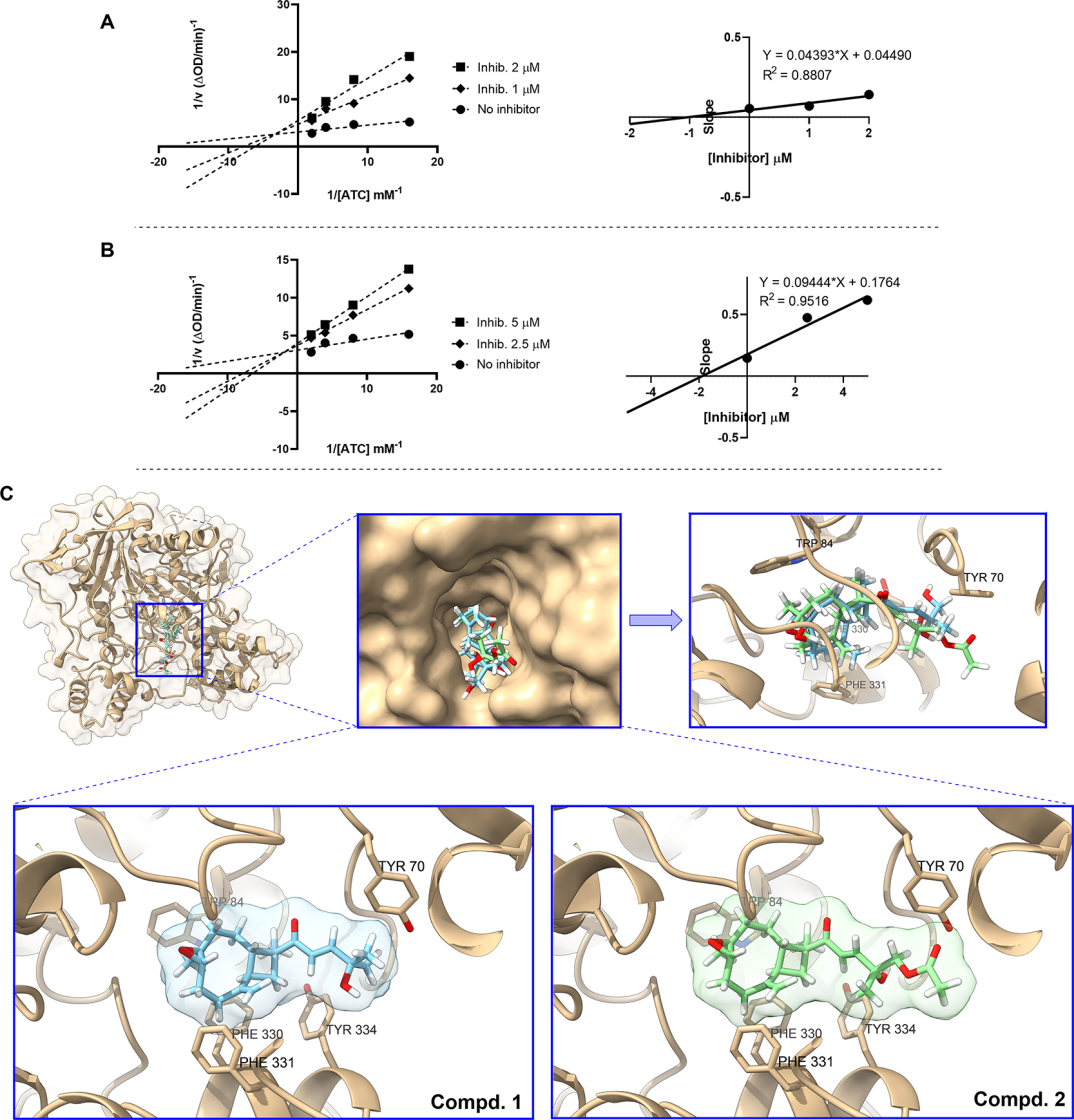
AChE inhibition kinetics and docking analysis of compounds **1** and **2**. (A) Lineweaver–Burk plots and secondary slope analysis for compound **1**. (B) Lineweaver–Burk plots and corresponding slope analysis for compound **2**. (C) Molecular docking showing the binding modes of compounds **1** and **2** in the enzyme active site and their interactions with key residues.

Molecular docking studies further supported the kinetic observations by providing detailed insights into ligand-AChE enzyme interactions (Figure 8C).^34–36^ Structurally, AChE and BChE share considerable similarity, with sequence identity ranging between 50 and 55% depending on species.^20^ Galanthamine was used as a reference ligand, and the docking protocol was validated by reproducing its binding pose with an RMSD of 0.99 Å (binding energy −12.29 kJ/mol), confirming the model’s reliability (not shown data). Docking results revealed that compound **1** exhibited the lowest predicted binding energy (−12.82 kJ/mol), approximately 2.11 kJ/mol lower than compound **2** (−10.71 kJ/mol), suggesting stronger affinity for the AChE active site. Compound **1** was found to establish hydrogen bonding interactions with Asp^72^, in addition to extensive π-alkyl interactions with aromatic residues including Trp^84^, Trp^279^, Phe^330^, Phe^331^, and Tyr^334^. The ligand was further stabilized by van der Waals contacts with residues Gly^118^, Gly^119^, Ser^81^, Ser^122^, His^440^, and Phe2^90^, allowing it to fit well within the active-site gorge. In contrast, compound **2** displayed a different interaction profile, predominantly engaging in π-alkyl interactions with key aromatic residues such as Trp^84^, Phe^330^, and Phe^331^, along with van der Waals contacts involving Tyr^70^, Asp^72^, Gly^118^, Gly^119^, Ser^81^, Ser^200^, His^440^, Glu^199^, and Phe^290^. Notably, no significant hydrogen bonding interactions were observed for compound **2**, which may result in weaker stabilization within the binding pocket. Overall, the differences in binding modes and interaction patterns between compounds **1** and **2** provide a clear molecular explanation for their distinct inhibitory activities. The presence of hydrogen bonding and more extensive interactions in compound **1** likely contributes to its stronger binding affinity and lower *K*_i_ value, whereas the interaction pattern of compound **2** is mainly governed by hydrophobic contacts, resulting in comparatively reduced inhibitory potency.

## Conclusions

In summary, four new terpenoids, including three xeniaphyllane-type diterpenoids, sclerohumins P–R (**1–3**), and a norcaryophyllene-type, sclerophyllene A (**4**), were obtained from the soft coral *Sclerophytum humesi* by combined bioassay- and ^1^H NMR-guided isolation. Their structures and absolute configurations were established by combined spectroscopic analyses and quantum chemical calculations. The stereogenic centers at C-15 in related xeniaphyllanes were proposed for the first time based on comparative SOR analysis. Compounds **1** and **2**, featuring a tricyclic 4/9-fused carbocyclic framework, exhibited potent and selective AChE inhibitory activity (IC_50_ = 1.7 and 4.4 μM; SI = 43.3 and 18.7, respectively), while being noncytotoxic toward HEK293 and Vero normal cell lines. Mechanistic investigations indicated that these compounds act as mixed-type AChE inhibitors, with *K*_i_ values of 1.02 and 1.87 μM for compounds **1** and **2**, respectively, as supported by enzyme kinetics and molecular docking. These results highlight the xeniaphyllane-type diterpenoid scaffold as a structurally interesting chemotype for acetylcholinesterase inhibition and provide a basis for further exploration of its cholinesterase-related biological properties.

## Experimental Section

### General Experimental Procedures

The compounds were isolated and purified from the crude extracts through a stepwise chromatographic workflow. Initial fractionation was carried out by fast column chromatography (FCC) using a SepaBean machine 2 (China) packed with silica gel. Further purification was achieved by high-performance liquid chromatography (HPLC). Semipreparative separations were performed on a Shimadzu LC-2050 system (Kyoto, Japan) equipped with a Galaksil EF-C_18_–H reverse-phase column (120 Å, 5 μm, 10 × 250 mm, China), enabling separation based on hydrophobic interactions. Structural characterization of the isolated metabolites was performed using multiple spectroscopic techniques. NMR spectra were recorded on an Agilent 600 MHz DD2 spectrometer (USA), including both 1D and 2D experiments, with methanol-*d*_4_ as the lock solvent. Detailed structural assignments were supported by gCOSY, gHSQC, gHMBC, and gNOESY analyses. High-resolution electrospray ionization mass spectrometry (HRESIMS) data were acquired using a Shimadzu LC–MS 9030 system (Japan). Infrared (IR) spectra were measured on an IRAffinity-1S FTIR spectrometer (Japan), while UV spectra were recorded using a U-3310 UV–vis spectrophotometer (Japan). Electronic circular dichroism (ECD) spectra were obtained on a Jasco J-715 spectropolarimeter, and optical rotation measurements were performed in methanol using a Jasco P-2000 polarimeter (Japan). The normal cell lines, embryonic kidney (HEK-293, CRL-1573) and monkey kidney epithelial (Vero, CCL-81) were obtained from the ATCC.

### Soft Coral Material

Specimens of the soft coral *Sclerophytum humesi* (voucher No. SH-2023) were collected by scuba diving at a depth of approximately 10–15 m off the coast of Pingtung County, Taiwan, in November 2023. Immediately after collection, the samples were frozen and subsequently stored at −20 °C until extraction. The authenticated voucher specimen was deposited in the Herbarium of the College of Pharmacy, Taipei Medical University, Taipei, Taiwan.

### Extraction and Isolation

The lyophilized specimen of *Sclerophytum humesi* (300 g) underwent thorough extraction using EtOAc at room temperature. The resulting extracts were evaporated under reduced pressure to obtain a brown residue (SH-EA), which weighed 18.1 g. The residue SH-EA (18.1 g) was subjected to fractionation using silica gel-FCC with a stepwise gradient elution. The separation was performed at a flow rate of 20 mL/min over a period of 180 min, utilizing a solvent system composed of Hex and EtOAc. The elution gradient gradually transitioned from Hex/EtOAc (100:0) to (0:100) to obtained 11 fractions (Fr.1-Fr.11).

To enable the targeted isolation of bioactive diterpenoid metabolites, a combined bioassay- and ^1^H NMR-guided strategy was employed. Bioassay-guided screening against AChE revealed that fraction Fr.7 exhibited the most potent inhibitory activity, with 95.8% inhibition at 50 μg/mL. Further evaluation demonstrated that subfraction Fr.7D displayed dose-dependent inhibitory activity, with inhibition rates of 56.8%, 75.2%, and 98.6% at 3.125, 6.25, and 12.5 μg/mL, respectively (Figure 2A). Subsequent ^1^H NMR analysis of Fr.7D revealed well-resolved signals, including an oxygenated methine proton at δ_H_ 2.95, olefinic protons at δ_H_ 6.54 and 6.97–6.90, and exomethylene protons at δ_H_ 4.93 and 5.02. These resonances are characteristic of xeniaphyllane-type diterpenoids, suggesting the presence of structurally interesting constituents (Figure 2B,C).

Accordingly, fraction 7D (512.8 mg) was selected for further purification and subjected to RP-HPLC using an isocratic elution system. The mobile phase consisted of a mixture of acidic water (0.1% formic acid) and acetonitrile (CH_3_CN) in a 40:60 ratio. Photodiode array (PDA) detection was carried out at wavelengths of 203 and 254 nm, resulting in the collection of four subfractions (7A-7D). Subfraction 7D (42.5 mg) was purified by reversed-phase chromatography on an EF-C_18_–H column under isocratic conditions, employing a mobile phase composed of 38% CH_3_CN in acidified water. This procedure afforded compound **4** (1.25 mg, *t*_R_ = 15.8 min), compound **1** (3.55 mg, *t*_R_ = 17.6 min), compound **3** (0.95 mg, *t*_R_ = 20.2 min), and compound **2** (3.80 mg, *t*_R_ = 21.8 min).

### Compound Characterization

*Sclerohumin P* (**1**): colorless oil; [α]_D_^25^ −15.2 (*c* 0.3, CHCl_3_); UV (MeOH) λ_max_ 230 nm; ECD (*c* 0.2 × 10^–3^ M, MeOH) λ_max_ (Δε) 208 (+27.1), 233 (−5.0) nm. IR ν_max_ (neat) 3412, 2968, 2931, 2866, 1681, 1624, 1454, 1375, 1043 cm^–1^; HRESIMS *m*/*z* [M + H]^+^ Calcd for C_20_H_31_O_4_ 335.2222; Found 335.2216. ^1^H (600 MHz, CD_3_OD) and ^13^C (150 MHz, CD_3_OD) NMR data are presented in Table 1.

*Sclerohumin Q* (**2**): colorless oil; [α]_D_^25^ −12.6 (*c* 0.3, CHCl_3_); UV (MeOH) λ_max_ 225 nm; ECD (*c* 0.1 × 10^–3^ M, MeOH) λ_max_ (Δε) 205 (+4.55), 226 (−9.82) nm; IR ν_max_ (neat) 3344, 2966, 2935, 2831, 1683, 1625, 1452, 1375, 1024 cm^–1^; HRESIMS *m*/*z* [M + H]^+^ Calcd for C_22_H_33_O_5_ 377.2328; Found 377.2321. ^1^H (600 MHz, CD_3_OD) and ^13^C (150 MHz, CD_3_OD) NMR data are presented in Table 1.

*Sclerohumin R* (**3**): colorless oil; [α]_D_^25^ +16.9 (*c* 0.3, CHCl_3_); UV (MeOH) λ_max_ 203 nm; ECD (*c* 0.1 × 10^–3^ M, MeOH) λ_max_ (Δε) 203 (−18.73), 224 (+10.71), 278 (+6.69) nm; IR ν_max_ (neat) 2966, 2929, 2875, 1732, 1699, 1450, 1373, 1244, 1047 cm^–1^; HRESIMS *m*/*z* [M + H]^+^ Calcd for C_21_H_33_O_4_ 349.2379; Found 349.2375. ^1^H (600 MHz, CD_3_OD) and ^13^C (150 MHz, CD_3_OD) NMR data are presented in Table 1.

*Sclerophyllene A* (**4**): colorless oil; [α]_D_^25^ +23.4 (*c* 0.3, CHCl_3_); UV (MeOH) λ_max_ 196 nm; ECD (*c* 0.1 × 10^–3^ M, MeOH) λ_max_ (Δε) 206 (−48.44), 232 (−6.95), 287 (5.92) nm; IR ν_max_ (neat) 3412, 2968, 2931, 2866, 1681, 1624, 1454, 1375, 1319, 1043 cm^–1^; HRESIMS *m*/*z* [M + H]^+^ Calcd for C_14_H_23_O_2_ 223.1698; Found 223.1692. ^1^H (600 MHz, CD_3_OD) and ^13^C (150 MHz, CD_3_OD) NMR data are presented in Table 1.

### NMR, SOR, and ECD Calculations

NMR, SOR, and ECD calculations were performed for compounds **1–4** in this study. Conformational searches for each stereoisomer were initially conducted using the GMMX method implemented in GaussView 6.1 (Gaussian Inc., Wallingford, CT, USA). The resulting conformers were subjected to geometry optimization and vibrational frequency analysis at the B3LYP/6-31G­(d,p) level with the IEFPCM solvent model for MeOH, using Gaussian 16 software (Gaussian Inc., Wallingford, CT, USA). All optimized conformers were confirmed as true minima by the absence of imaginary frequencies. Gibbs free energies were calculated to estimate Boltzmann populations, and conformers with populations below 2% were excluded from subsequent calculations.^10,20,21^

The remaining conformers were subjected to NMR chemical shift calculations using the GIAO-DFT method at the *m*PW1PW91/6-311+G­(d,p) level with the IEFPCM solvent model for MeOH. Unscaled chemical shifts (δ_u_) were obtained relative to tetramethylsilane (TMS) according to the equation δ_u_ = σ_o_ – σ_*x*_, where σ_*x*_ represents the Boltzmann-averaged shielding constants of all significant conformers and σ_o_ corresponds to the TMS shielding constant calculated at the same level of theory. The experimental ^13^C NMR data of compounds **1–4** were then compared with the calculated values for isomers **1a**–**1h**, **2a**–**2b**, **3a**–**3h**, and **4a**–**4d** using mean absolute error (MAE), correlation coefficient (*R* ^2^), and DP4+ probability analyses.^10^

Theoretical SOR calculations were carried out using DFT at the APFD/6-311++G­(2d,2p) level. Specific rotation values were computed at 589 nm (sodium d-line) by evaluating molecular polarizability with inclusion of vibrational contributions.^37^ This approach enables reliable prediction of SOR, thereby facilitating comparison with experimental data for stereochemical assignment.

For the ECD calculations, excitation energies for the lowest 30 electronic transitions were computed using TDDFT at the B3LYP/6-311++G­(d,p) level with the IEFPCM solvent model for MeOH.^10,20,21^ The ECD spectra of individual conformers were subsequently Boltzmann-weighted to generate overall spectra using SpecDis 1.71, applying a Gaussian band broadening parameter (σ) of 0.30 eV. The resulting calculated spectra were then compared with the experimental CD spectra, recorded over the wavelength range of 200–400 nm (301 data points) with CD intensities expressed in millidegrees (mdeg).

### Acetylcholinesterase (AChE) and Butyrylcholinesterase (BChE) Inhibitory Assay

The inhibitory activities of the tested compounds against AChE and BChE were evaluated using a modified Ellman’s colorimetric method.^38–41^ Enzymes, including electric eel AChE and equine serum BChE, as well as reagents such as 5,5′-dithiobis­(2-nitrobenzoic acid) (DTNB), phosphate-buffered saline (PBS, pH 8.0), acetylthiocholine iodide (ATC), and butyrylthiocholine iodide (BTC), were purchased from Sigma-Aldrich (Steinheim, Germany). Galantamine and quercetin were used as reference inhibitors. Enzyme solutions were prepared at a concentration of 2.0 U/mL in 2 mL aliquots. Each reaction mixture consisted of 10 μL of enzyme solution, 40 μL of PBS, 20 μL of 0.01 M DTNB, and 10 μL of test compound, and was tested in triplicate (n = 3). The enzyme-compound mixtures were preincubated for 5 min prior to substrate addition. The reaction was initiated by adding 20 μL of 0.01 M substrate (ATC for AChE or BTC for BChE). Absorbance was monitored at 410 nm every 3 min at 37 °C using a microplate reader.

Kinetic studies of AChE inhibition were performed using Ellman’s method.^42,43^ Substrate concentrations were varied at 0.0625, 0.125, 0.25, and 0.5 μM to obtain Michaelis–Menten kinetic data.^20^ Lineweaver–Burk plots were generated using linear regression analysis to determine kinetic parameters and to elucidate the mode of inhibition.

### Cytotoxicity Assessment

The experimental procedures and analytical methods were adapted from previously reported protocols with minor modifications to the tested concentration ranges.^44,45^ Cells at approximately 80% confluence were washed with phosphate-buffered saline (PBS) and detached using 0.25% trypsin-0.02% EDTA (Gibco) at 37 °C for 5 min. The harvested cells were seeded into 96-well plates at a density of 3 × 10^4^ cells per well in 100 μL of fresh medium and incubated at 37 °C under 5% CO_2_ for 24 h. Subsequently, 100 μL of fresh medium containing the test compounds was added to each well, followed by further incubation for 72 h. Cell viability was assessed using the Cell Counting Kit-8 (CCK-8) assay according to the manufacturer’s instructions, and absorbance was measured at 450 nm using a microplate reader. Cell viability (%) was calculated as

$$\left(\%\right)\;Cell\;viability=\left[\left({Abs}_{sample}-{Abs}_{blank}{)/(Abs}_{control}-{Abs}_{blank}\right)\right]\times 100\%$$

All cytotoxicity assays were performed in triplicate (n = 3). Compounds showing more than 50% growth inhibition were further evaluated for IC_50_ values using GraphPad Prism (version 8.0.2). Quercetin was used as a positive control.

### *In Silico* Studies

Molecular docking studies were performed and analyzed following previously reported protocols.^20,46,47^ Ligand geometries were optimized in Gaussian 16 using the B3LYP/6-311++G­(d,p) basis set. The crystal structure of AChE was obtained from the RCSB Protein Data Bank (PDB ID: 1QTI), and all water molecules and cocrystallized ligands were removed using Discovery Studio Visualizer. Docking calculations were carried out in LeadIT, with grid boxes defined to cover residues within the catalytic gorge and the peripheral anionic site. For each ligand, 50 binding poses were generated and ranked based on docking scores and predicted binding energies (kJ/mol), and the top-ranked conformations were selected for interaction analysis.

### Statistical Analysis

Statistical evaluations for the in vitro studies were performed using GraphPad Prism (version 8.0.2). Data are expressed as the mean ± standard deviation (SD). Statistical significance was evaluated by one-way analysis of variance (ANOVA) followed by Tukey’s post hoc test for multiple comparisons. A *p*-value < 0.05 was considered statistically significant.

## Supplementary Material





## Data Availability

The data underlying this study are available in the published article and its Supporting Information.

## Notes

Detailed experimental procedures, computational calculations, and NMR, HRESIMS, UV, and IR spectra of compounds **1–4** (PDF)
Raw NMR data of compound **1** (Sclerohumin P), Raw NMR data of compound **2** (Sclerohumin Q), Raw NMR data of compound **3** (Sclerohumin R), Raw NMR data of compound **4** (Sclerophyllene A) (ZIP)
