# Supplementary material for: Xeniaphyllane-Type Diterpenoids from the Soft Coral Sclerophytum humesi: Resolving Absolute Configurations and Anti-Alzheimer’s Potential
Source: J Nat Prod. 2026 Jul 9;89(7):2143–56. doi: 10.1021/acs.jnatprod.6c00535 (PMC13418205; doi:10.1021/acs.jnatprod.6c00535)
Supplement: Supplementary file 1 [file np6c00535_si_001.pdf]

Supporting Information

Xeniaphyllane-Type Diterpenoids from the Soft Coral *Sclerophytum humesi*: Resolving Absolute Configurations and Anti-Alzheimer's Potential

Phuong Vu Luu[†], Cuong-Quoc Nguyen[‡], Huong Lien Ton-Nu[⁑], Thuy-Tien Thi Phan[‖,$], Quoc-Dung Tran Huynh[$,°], Ngoc-Thac Pham[#,Δ], Huong-Giang Le[#], Lo-Yun Chen[Δ], Yu-Chia Chang[□,◊], Jui-Hsin Su[∞,⦀,⁑⁑], Bo-Rong Peng[†,Δ,□,##,*], Kuei-Hung Lai[†,Δ,∞,⁋,⌂,*]

[†]*Graduate Institute of Pharmacognosy, College of Pharmacy, Taipei Medical University, Taipei 110301, Taiwan*

[‡]*Department of Health Sciences, College of Natural Sciences, Can Tho University, Can Tho 94000, Vietnam*

[⁑]*Department of Chemistry, College of Natural Sciences, Can Tho University, Can Tho 94000, Vietnam.*

[‖]*Graduate Institute of Biomedical Materials and Tissue Engineering, College of Biomedical Engineering, Taipei Medical University, Taipei 110301, Taiwan*

[$]*Institute of Pharmaceutical Education and Research, Binh Duong University, Thu Dau Mot, Binh Duong 820000, Vietnam*

[°]*Institute of Biological Chemistry, Academia Sinica, Taipei 115024, Taiwan*

[#]*Department of Pharmacognosy and Traditional Pharmacy, School of Pharmacy, University of Medicine and Pharmacy at Ho Chi Minh City, Ho Chi Minh City 700000, Vietnam*

[△]*PhD Program in Clinical Drug Development of Herbal Medicine, College of Pharmacy, Taipei Medical University, Taipei 110301, Taiwan*

[□]*Graduate Institute of Healthy Industry Technology, Center for Drug Research and Development, College of Human Ecology, Chang Gung University of Science and Technology, Taoyuan 333324, Taiwan*

[◊]*Department of Cosmetic Science, Chang Gung University of Science and Technology, Taoyuan City 33303, Taiwan*

[∞]*National Museum of Marine Biology & Aquarium, Pingtung 94450, Taiwan*

[‖]*Department of Marine Biotechnology and Resources, National Sun Yat-sen University, Kaohsiung 804, Taiwan*

[⁑]*Graduate Institute of Marine Biology, National Dong Hwa University, Pingtung 94450, Taiwan*

[##]*Department of Biochemistry and Molecular Cell Biology, School of Medicine, College of Medicine, Taipei Medical University, Taipei 110301, Taiwan*

[⁋]*Traditional Herbal Medicine Research Center, Taipei Medical University Hospital, Taipei 110301, Taiwan*

[⌂]*PhD Program in Drug Discovery and Development Industry, College of Pharmacy, Taipei Medical University, Taipei 110301, Taiwan*

***Corresponding Author:**

Email: brpeng@mail.cgust.edu.tw; Email: kueihunglai@tmu.edu.tw

Contents

1. Spectra data for compounds 1-4..S10

1.1. NMR, HRESIMS, IR, and UV spectra of sclerohumin P (1)..S10

1.2. NMR, HRESIMS IR, and UV spectra of sclerohumin Q (2)..S25

1.3. NMR, HRESIMS, IR, and UV spectra of sclerohumin R (3)..S40

1.4. NMR, HRESIMS, IR, and UV spectra of sclerophyllene A (4)......................................S55

2. Optical Rotation...S74

3. Computational details...S79

3.1. Structures of isomers studied..S79

3.2. Gibbs free energy of the conformers and the Boltzmann distribution.............................S83

3.3. The cartesian coordinates of the dominant conformers of compounds 1-4....................S108

3.4. DP4+ results of compounds 1-4...S130

3.5. Correlation plots of compounds 1-4...S134

4. Michaelis-Menten plots of enzyme activity...S139

Table legend

Table S1. 2D NMR data for compound **1**......S70

Table S2. 2D NMR data for compound **2**......S71

Table S3. 2D NMR data for compound **3**......S72

Table S4. 2D NMR data for compound **4**......S73

Table S5. Optical Rotation of compound **1**......S74

Table S6. Optical Rotation of compound **2**......S75

Table S7. Optical Rotation of compound **3**......S76

Table S8. Optical Rotation of compound **4**......S77

Table S9. Gibbs free energy of the conformers and the Boltzmann distribution for **1**-5S11*S*15*S*......S83

Table S10. Gibbs free energy of the conformers and the Boltzmann distribution for **1**-5*S*11*S*15*R*......S85

Table S11. Gibbs free energy of the conformers and the Boltzmann distribution for **1**-5*S*11*R*15*S*......S87

Table S12. Gibbs free energy of the conformers and the Boltzmann distribution for **1**-5*S*11*R*15*R*...... 88

Table S13. Gibbs free energy of the conformers and the Boltzmann distribution for **1**-5*R*11*S*15*S*......S89

Table S14. Gibbs free energy of the conformers and the Boltzmann distribution for **1**-5*R*11*S*15*R*...... S90

Table S15. Gibbs free energy of the conformers and the Boltzmann distribution for **1**-5*R*11*R*15*S*...... S91

Table S16. Gibbs free energy of the conformers and the Boltzmann distribution for **1**-5*R*11*R*15*R*...... S92

Table S17. Gibbs free energy of the conformers and the Boltzmann distribution for **2**-15*S*......S93

Table S18. Gibbs free energy of the conformers and the Boltzmann distribution for **2**-15*R*......S94

Table S19. Gibbs free energy of the conformers and the Boltzmann distribution for **3**-4*S*11*S*15*S*......S96

Table S20. Gibbs free energy of the conformers and the Boltzmann distribution for **3**-4*S*11*S*15*R*......S97

Table S21. Gibbs free energy of the conformers and the Boltzmann distribution for **3**-4*S*11*R*15*S*......S98

Table S22. Gibbs free energy of the conformers and the Boltzmann distribution for **3**-4*S*11*R*15*R*….. S99

Table S23. Gibbs free energy of the conformers and the Boltzmann distribution for **3**-4*R*11*S*15*S*….. S100

Table S24. Gibbs free energy of the conformers and the Boltzmann distribution for **3**-4*R*11*S*15*R*….. S101

Table S25. Gibbs free energy of the conformers and the Boltzmann distribution for **3**-4*R*11*R*15*S*….. S102

Table S26. Gibbs free energy of the conformers and the Boltzmann distribution for **3**-4*R*11*R*15*R*.. S103

Table S27. Gibbs free energy of the conformers and the Boltzmann distribution for **4**-1*S*4*S*...S104

Table S28. Gibbs free energy of the conformers and the Boltzmann distribution for **4**-1*S*4*R*.. S105

Table S29. Gibbs free energy of the conformers and the Boltzmann distribution for **4**-1*R*4*S*.. S106

Table S30. Gibbs free energy of the conformers and the Boltzmann distribution for **4**-1*R*4*R*..S107

Figure legend

Figure S1. ^{1}H NMR spectrum of sclerohumin P (**1**) (600 MHz, CD_3OD)................................S10

Figure S2. Expanded ^{1}H NMR spectrum of sclerohumin P (**1**) (600 MHz, CD_3OD).................S11

Figure S3. ^{1}H NMR spectrum of sclerohumin P (**1**) with chemical shift values displayed above the signals (600 MHz, CD_3OD)..S12

Figure S4. Expanded ^{1}H NMR spectrum of sclerohumin P (**1**) with chemical shift values displayed above the signals (600 MHz, CD_3OD)..S13

Figure S5. ^{13}C NMR spectrum of sclerohumin P (**1**) (150 MHz, CD_3OD)................................S14

Figure S6. ^{13}C NMR spectrum of sclerohumin P (**1**) with chemical shift values displayed above the signals (150 MHz, CD_3OD)..S15

Figure S7. DEPT spectra of sclerohumin P (**1**) (150 MHz, CD_3OD).......................................S16

Figure S8. HSQC spectrum of sclerohumin P (**1**) (600 and 150 MHz, CD_3OD).......................S17

Figure S9. Expanded HSQC spectrum of sclerohumin P (**1**) (600 and 150 MHz, CD_3OD).......S18

Figure S10. HMBC spectrum of sclerohumin P (**1**) (600 and 150 MHz, CD_3OD)....................S19

Figure S11. COSY spectrum of sclerohumin P (**1**) (600 MHz, CD_3OD)..................................S20

Figure S12. NOESY spectrum of sclerohumin P (**1**) (600 MHz, CD_3OD)................................S21

Figure S13. HRESIMS spectrum of sclerohumin P (**1**)..S22

Figure S14. Infrared (IR) spectrum sclerohumin P (**1**)..S23

Figure S15. Ultraviolet (UV) spectrum sclerohumin P (**1**)...S24

Figure S16. ^{1}H NMR spectrum of sclerohumin Q (**2**) (600 MHz, CD_3OD).............................S25

Figure S17. Expanded ^{1}H NMR spectrum of sclerohumin Q (**2**) (600 MHz, CD_3OD)..............S26

Figure S18. ^{1}H NMR spectrum of sclerohumin Q (**2**) with chemical shift values displayed above the signals (600 MHz, CD_3OD)...S27

Figure S19. Expanded ^{1}H NMR spectrum of sclerohumin Q (**2**) with chemical shift values displayed above the signals (600 MHz, CD_3OD)...S28

Figure S20. ^{13}C NMR spectrum of sclerohumin Q (**2**) (150 MHz, CD_3OD).............................S29

Figure S21. ^{13}C NMR spectrum of sclerohumin Q (**2**) with chemical shift values displayed above the signals (150 MHz, CD_3OD)...S30

Figure S22. DEPT spectra of sclerohumin Q (**2**) (150 MHz, CD_3OD).....................................S31

Figure S23. HSQC spectrum of sclerohumin Q (**2**) (600 and 150 MHz, CD_3OD)....................S32

Figure S24. Expanded HSQC spectrum of sclerohumin Q (**2**) (600 and 150 MHz, CD_3OD)….S33

Figure S25. HMBC spectrum of sclerohumin Q (**2**) (600 and 150 MHz, CD_3OD)...................S34

Figure S26. COSY spectrum of sclerohumin Q (**2**) (600 MHz, CD_3OD).................................S35

Figure S27. NOESY spectrum of sclerohumin Q (**2**) (600 MHz, CD_3OD)................................S36
Figure S28. HRESIMS spectrum of sclerohumin Q (**2**)..S37
Figure S29. Infrared (IR) spectrum sclerohumin Q (**2**)..S38
Figure S30. Ultraviolet (UV) spectrum sclerohumin Q (**2**)..S39
Figure S31. ^{1}H NMR spectrum of sclerohumin R (**3**) (600 MHz, CD_3OD)...............................S40
Figure S32. Expanded ^{1}H NMR spectrum of sclerohumin R (**3**) (600 MHz, CD_3OD)..............S41
Figure S33. ^{1}H NMR spectrum of sclerohumin R (**3**) with chemical shift values displayed above the signals (600 MHz, CD_3OD)...S42
Figure S34. Expanded ^{1}H NMR spectrum of sclerohumin R (**3**) with chemical shift values displayed above the signals (600 MHz, CD_3OD)...S43
Figure S35. ^{13}C NMR spectrum of of sclerohumin R (**3**) (600 MHz, CD_3OD)..........................S44
Figure S36. ^{13}C NMR spectrum of sclerohumin R (**3**) with chemical shift values displayed above the signals (150 MHz, CD_3OD)...S45
Figure S37. DEPT spectra of of sclerohumin R (**3**) (600 MHz, CD_3OD)..................................S46
Figure S38. HSQC spectrum of sclerohumin R (**3**) (600 and 150 MHz, CD_3OD).....................S47
Figure S39. Expanded HSQC spectrum of sclerohumin R (**3**) (600 and 150 MHz, CD_3OD).....S48
Figure S40. HMBC spectrum of sclerohumin R (**3**) (600 and 150 MHz, CD_3OD)...................S49
Figure S41. COSY spectrum of sclerohumin R (**3**) (600 MHz, CD_3OD).................................S50
Figure S42. NOESY spectrum of sclerohumin R (**3**) (600 MHz, CD_3OD)................................S51
Figure S43. HRESIMS spectrum of sclerohumin R (**3**)...S52
Figure S44. Infrared (IR) spectrum sclerohumin R (**3**)..S53
Figure S45. Ultraviolet (UV) spectrum sclerohumin R (**3**)...S54
Figure S46. ^{1}H NMR spectrum of sclerophyllene A (**4**) (600 MHz, CD_3OD)...........................S55
Figure S47. Expanded ^{1}H NMR spectrum of sclerophyllene A (**4**) (600 MHz, CD_3OD)...........S56
Figure S48. ^{1}H NMR spectrum of sclerophyllene A (**4**) with chemical shift values displayed above the signals (600 MHz, CD_3OD)...S57
Figure S49. Expanded ^{1}H NMR spectrum of sclerophyllene A (**4**) with chemical shift values displayed above the signals (600 MHz, CD_3OD)...S58
Figure S50. ^{13}C NMR spectrum of sclerophyllene A (**4**) (150 MHz, CD_3OD)...........................S59
Figure S51. ^{13}C NMR spectrum of sclerophyllene A (**4**) with chemical shift values displayed above the signals (150 MHz, CD_3OD)...S60
Figure S52. DEPT spectra of sclerophyllene A (**4**) (150 MHz, CD_3OD)..................................S61
Figure S53. HSQC spectrum of sclerophyllene A (**4**) (600 and 150 MHz, CD_3OD).................S62

Figure S54. Expanded HSQC spectrum of sclerophyllene A (**4**) (600 and 150 MHz, CD_3OD). S63
Figure S55. HMBC spectrum of sclerophyllene A (**4**) (600 and 150 MHz, CD_3OD)................S64
Figure S56. COSY spectrum of sclerophyllene A (**4**) (600 MHz, CD_3OD)...............................S65
Figure S57. NOESY spectrum of sclerophyllene A (**4**) (600 MHz, CD_3OD)............................S66
Figure S58. HRESIMS spectrum of sclerophyllene A (**4**)..S67
Figure S59. Infrared (IR) spectrum sclerophyllene A (**4**)...S68
Figure S60. Ultraviolet (UV) spectrum sclerophyllene A (**4**)...S69
Figure S61. Optical Rotation of sclerohumin Q (**2**)..S78
Figure S62. Structures of isomers **1a**-**1h** of compound **1**...S79
Figure S63. Structures of isomers **2a** and **2b** of compound **2**...S80
Figure S64. Structures of isomers **3a**-**3h** of compound **3**...S81
Figure S65. Structures of isomers **4a**-**4d** of compound **4**...S82
Figure S66. The cartesian coordinates of the dominant conformers for conformers **1**-5*S*11*S*15*S*...S109
Figure S67. The cartesian coordinates of the dominant conformers for conformers **1**-5*S*11*S*15*R*...S110
Figure S68. The cartesian coordinates of the dominant conformers for conformers **1**-5*S*11*R*15*S*...S111
Figure S69. The cartesian coordinates of the dominant conformers for conformers **1**-5*S*11*R*15*R*...S112
Figure S70. The cartesian coordinates of the dominant conformers for conformers **1**-5*R*11*S*15*S*...S113
Figure S71. The cartesian coordinates of the dominant conformers for conformers **1**-5*R*11*S*15*R*...S114
Figure S72. The cartesian coordinates of the dominant conformers for conformers **1**-5*R*11*R*15*S*...S115
Figure S73. The cartesian coordinates of the dominant conformers for conformers **1**-5*R*11*R*15*R*...S116
Figure S74. The cartesian coordinates of the dominant conformers for conformers **2**-15*S*......S117
Figure S75. The cartesian coordinates of the dominant conformers for conformers **2**-15*R*......S118
Figure S76. The cartesian coordinates of the dominant conformers for conformers **3**-4*S*11*S*15*S*...S119
Figure S77. The cartesian coordinates of the dominant conformers for conformers **3**-4*S*11*S*15*R*...S120

Figure S78. The cartesian coordinates of the dominant conformers for conformers **3**-4*S*11*R*15*R*...........S121

Figure S79. The cartesian coordinates of the dominant conformers for conformers **3**-4*R*11*S*15*S*...........S122

Figure S80. The cartesian coordinates of the dominant conformers for conformers **3**-4*R*11*S*15*R*...........S123

Figure S81. The cartesian coordinates of the dominant conformers for conformers **3**-4*R*11*R*15*S*...........S124

Figure S82. The cartesian coordinates of the dominant conformers for conformers **3**-4*R*11*R*15*R*...........S125

Figure S83. The cartesian coordinates of the dominant conformers for conformers **4**-1*S*4*S*....S126

Figure S84. The cartesian coordinates of the dominant conformers for conformers **4**-1*S*4*R*.. .S127

Figure S85. The cartesian coordinates of the dominant conformers for conformers **4**-1*R*4*S*.. .S128

Figure S86. The cartesian coordinates of the dominant conformers for conformers **4**-1*R*4*R*...S129

Figure S87. DP4+ results obtained using experimental data of **1** *versus* isomers 1-8.............S130

Figure S88. DP4+ results obtained using experimental data of **2** *versus* isomers 1 and 2........S131

Figure S89. DP4+ results obtained using experimental data of **3** *versus* isomers 1-8.............S132

Figure S90. DP4+ results obtained using experimental data of **4** *versus* isomers 1-4.............S133

Figure S91. Linear correlations of the calculated isomers of **1** with the experimentally observed ^{13}C NMR chemical shifts...........S135

Figure S92. Linear correlations of the calculated isomers of **3** with the experimentally observed ^{13}C NMR chemical shifts...........S137

Figure S93. Linear correlations of the calculated isomers of **4** with the experimentally observed ^{13}C NMR chemical shifts...........S138

Figure S94. Michaelis-Menten plots of enzyme activity in the absence and presence of inhibitor at different concentrations. (**A**) Michaelis–Menten plot obtained in the absence of inhibitor. (**B**) Michaelis–Menten plots in the presence of inhibitor at concentrations of 1 and 2 µM of compound **1**. (**C**) Michaelis–Menten plots in the presence of inhibitor at concentrations of 2.5 and 5 µM of compound **2**...........S139

1. Spectra data for compounds 1-4

1.1. NMR, HRESIMS, IR, and UV spectra of sclerohumin P (1)

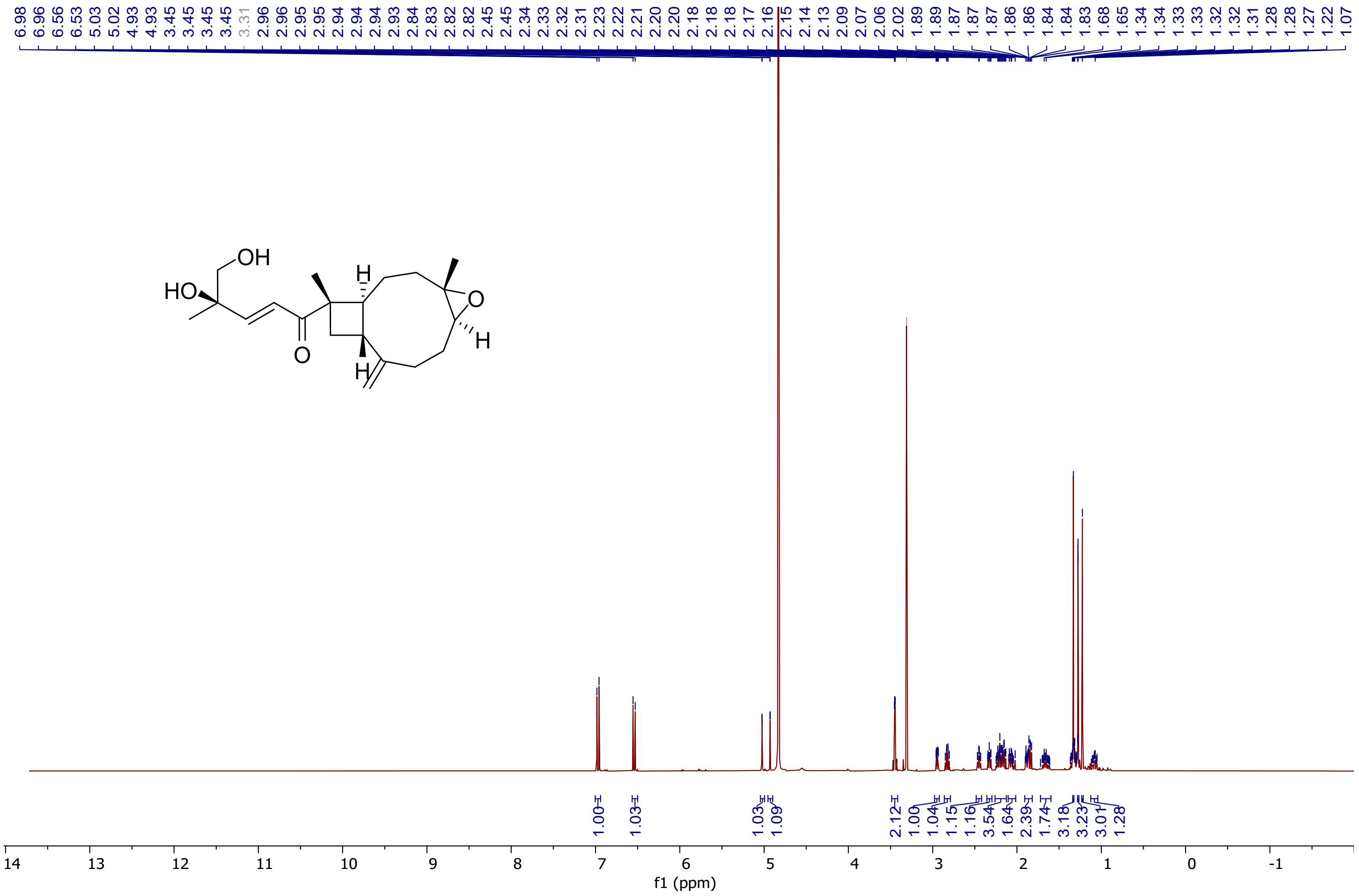

Figure S1. ^{1}H NMR spectrum of sclerohumin P (**1**) (600 MHz, CD_3OD).

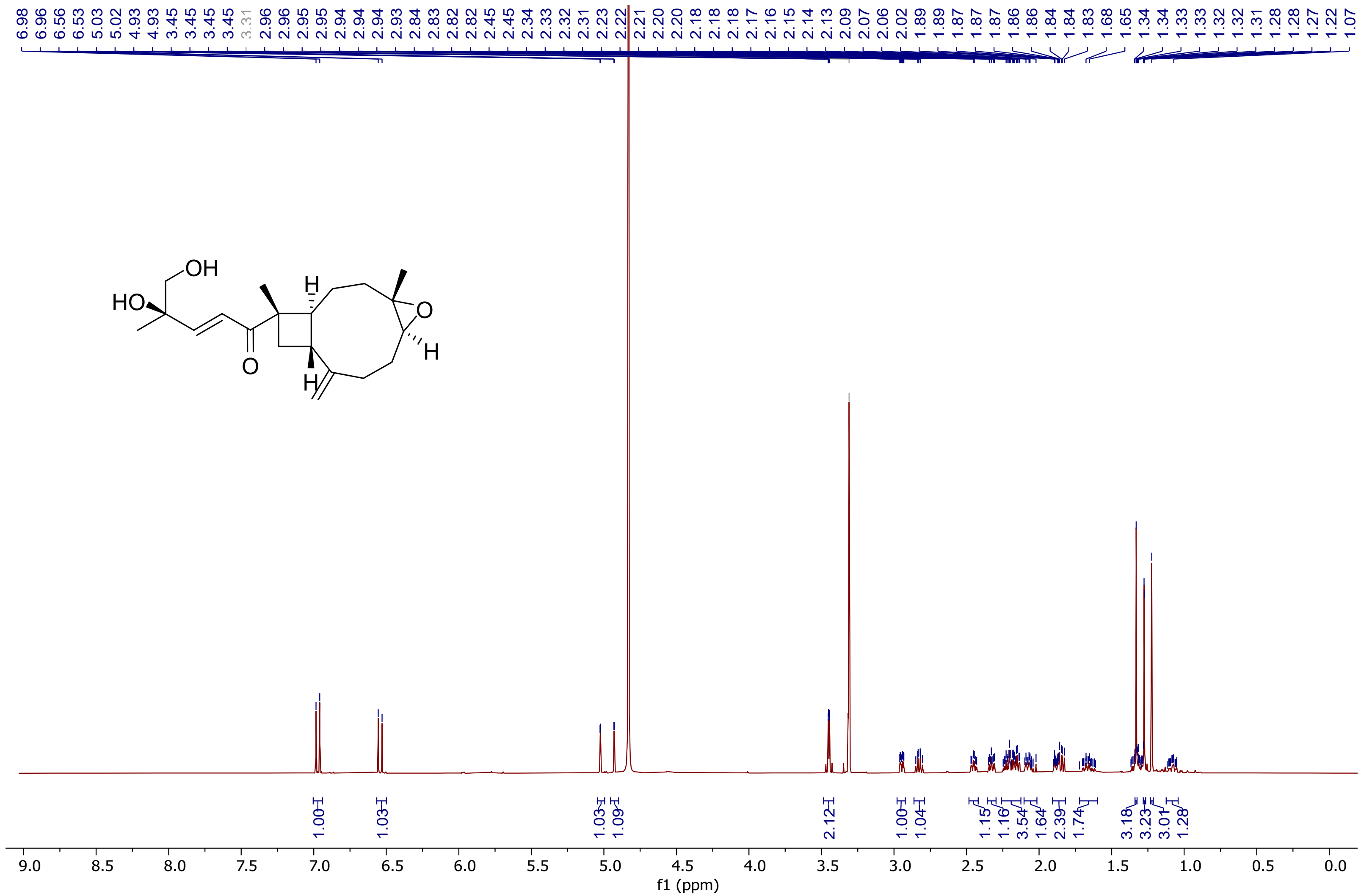

Figure S2. Expanded ^{1}H NMR spectrum of sclerohumin P (**1**) (600 MHz, CD_3OD).

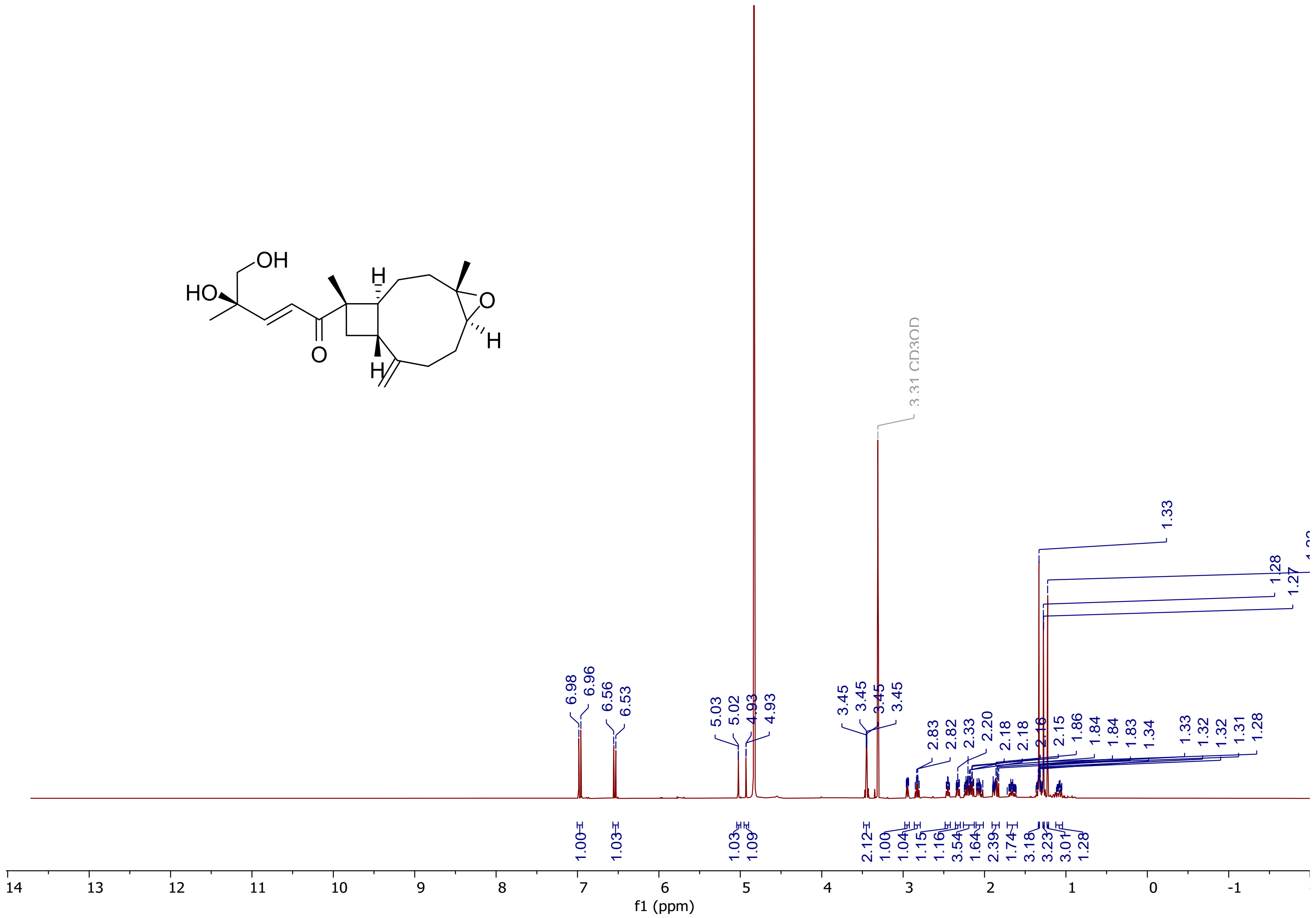

Figure S3. ^{1}H NMR spectrum of sclerohumin P (**1**) with chemical shift values displayed above the signals (600 MHz, CD_3OD).

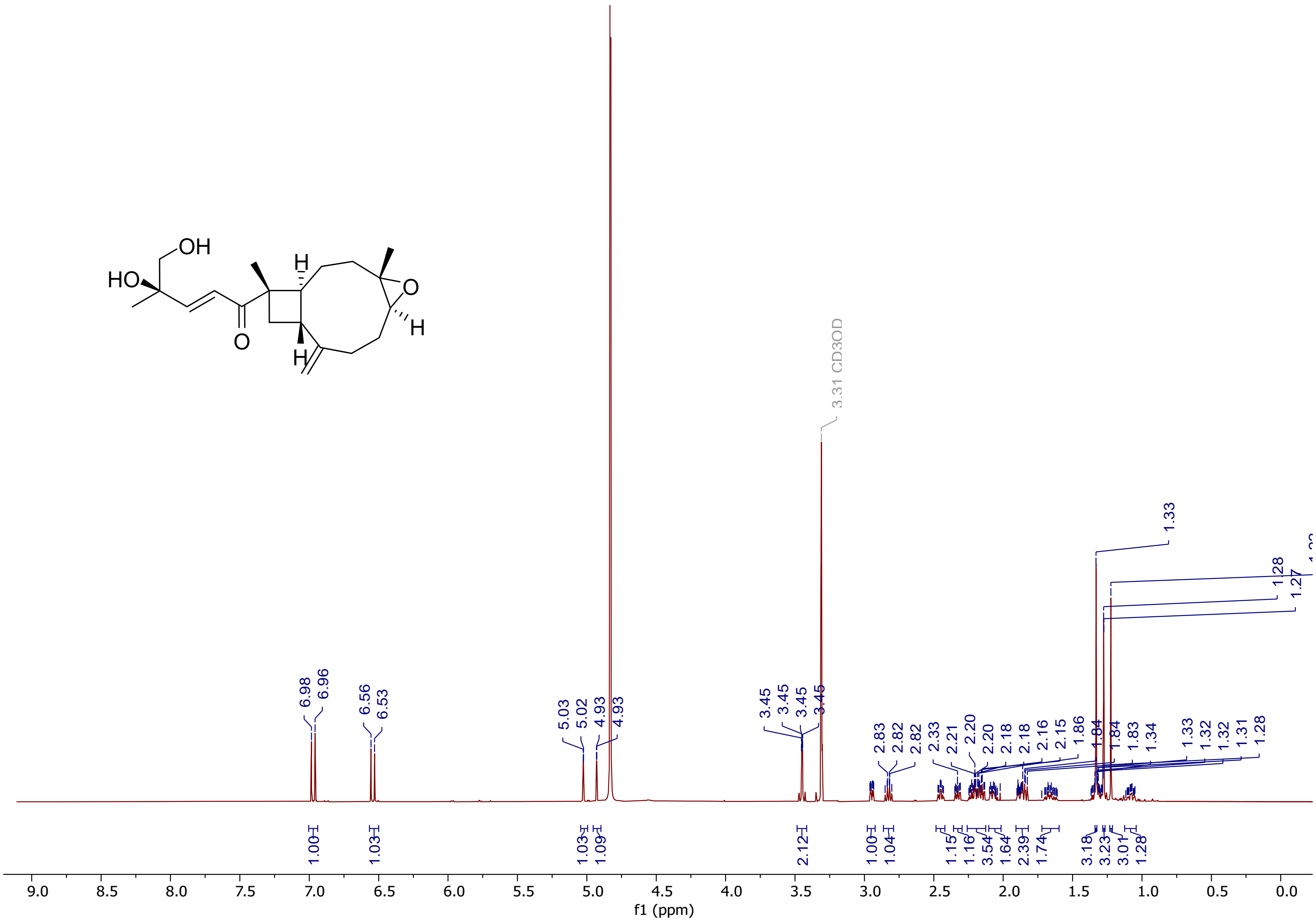

Figure S4. Expanded ^{1}H NMR spectrum of sclerohumin P (**1**) with chemical shift values displayed above the signals (600 MHz, CD_3OD).

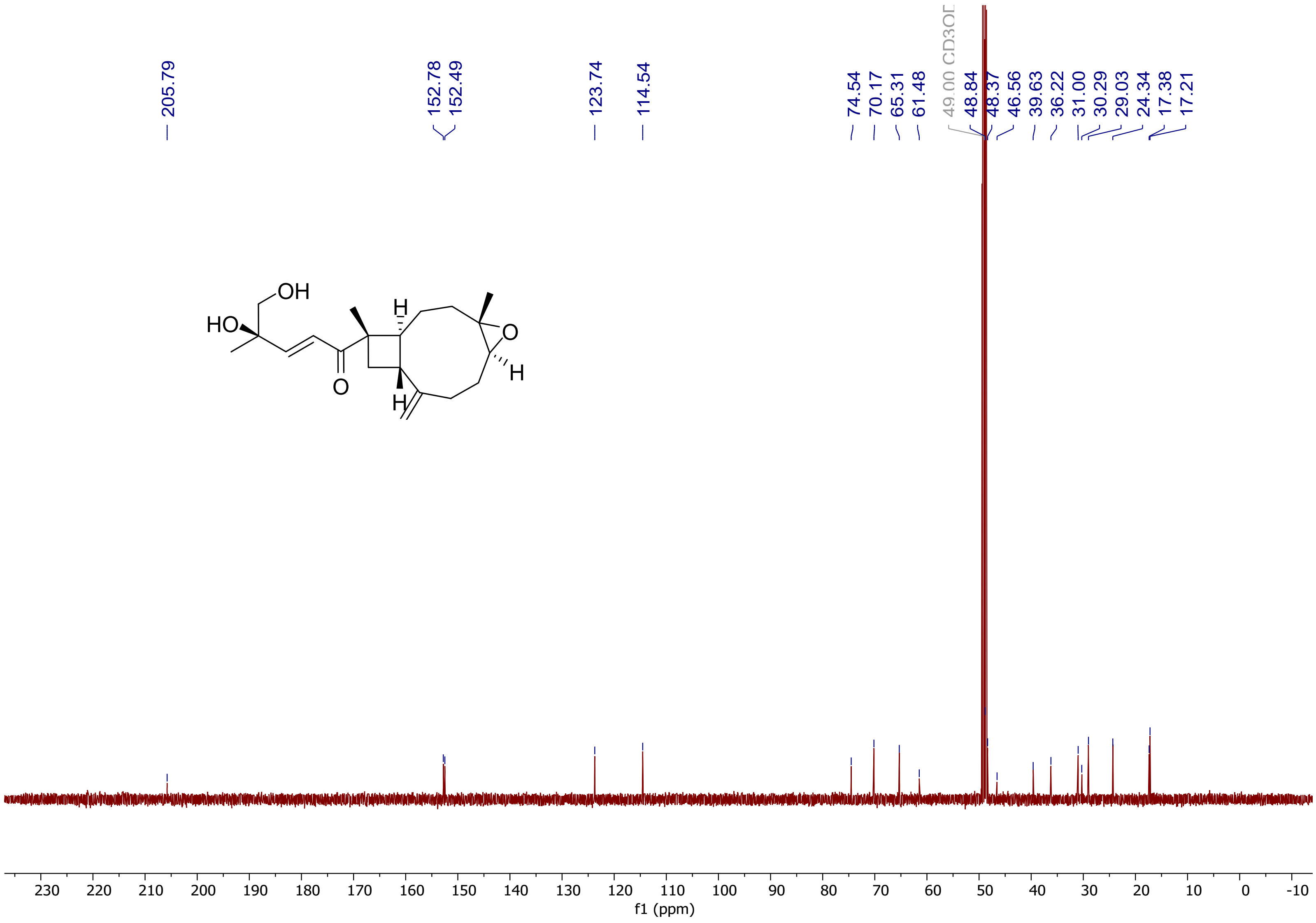

Figure S5. ^{13}C NMR spectrum of sclerohumin P (**1**) (150 MHz, CD_3OD).

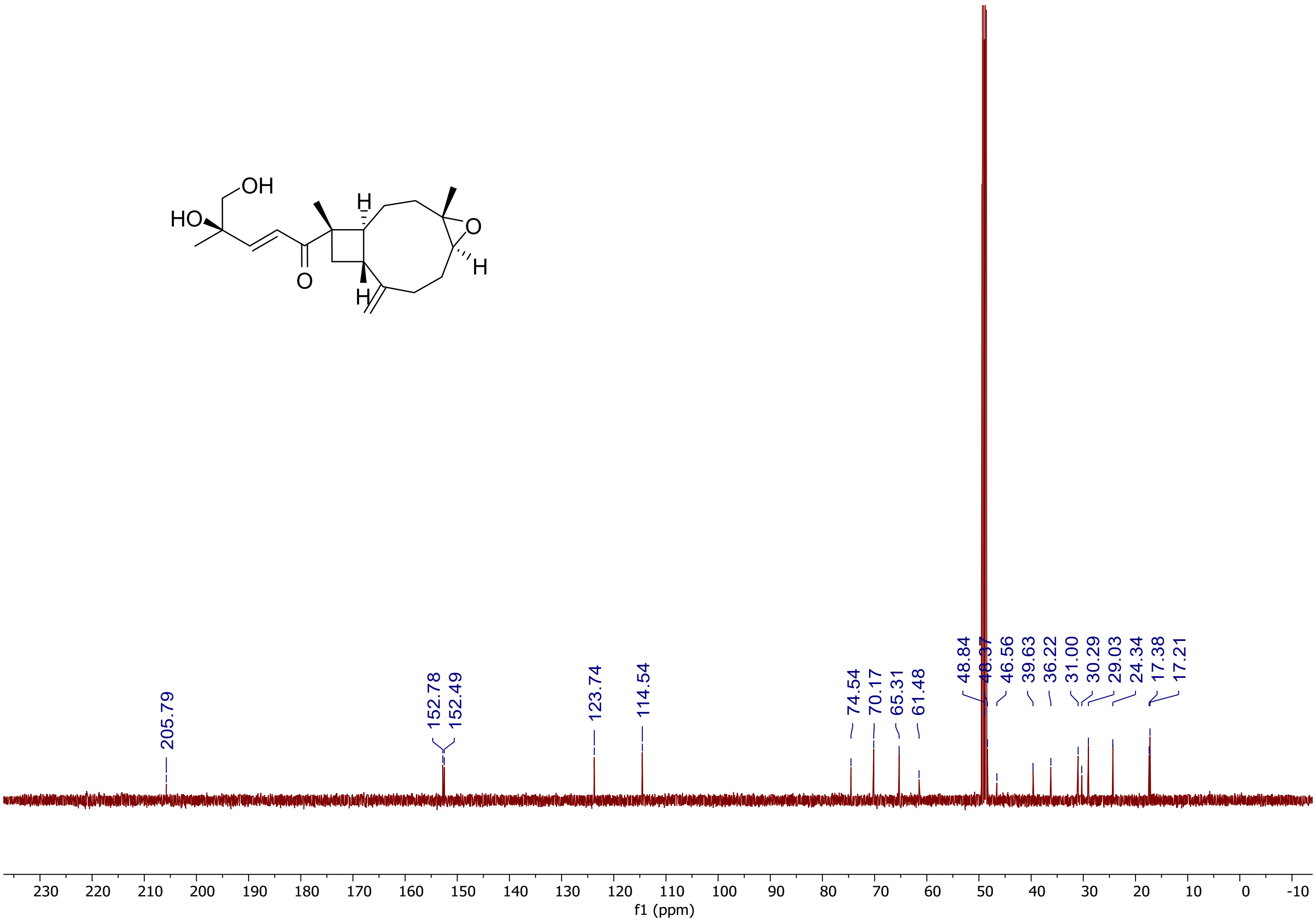

Figure S6. ^{13}C NMR spectrum of sclerohumin P (**1**) with chemical shift values displayed above the signals (150 MHz, CD_3OD).

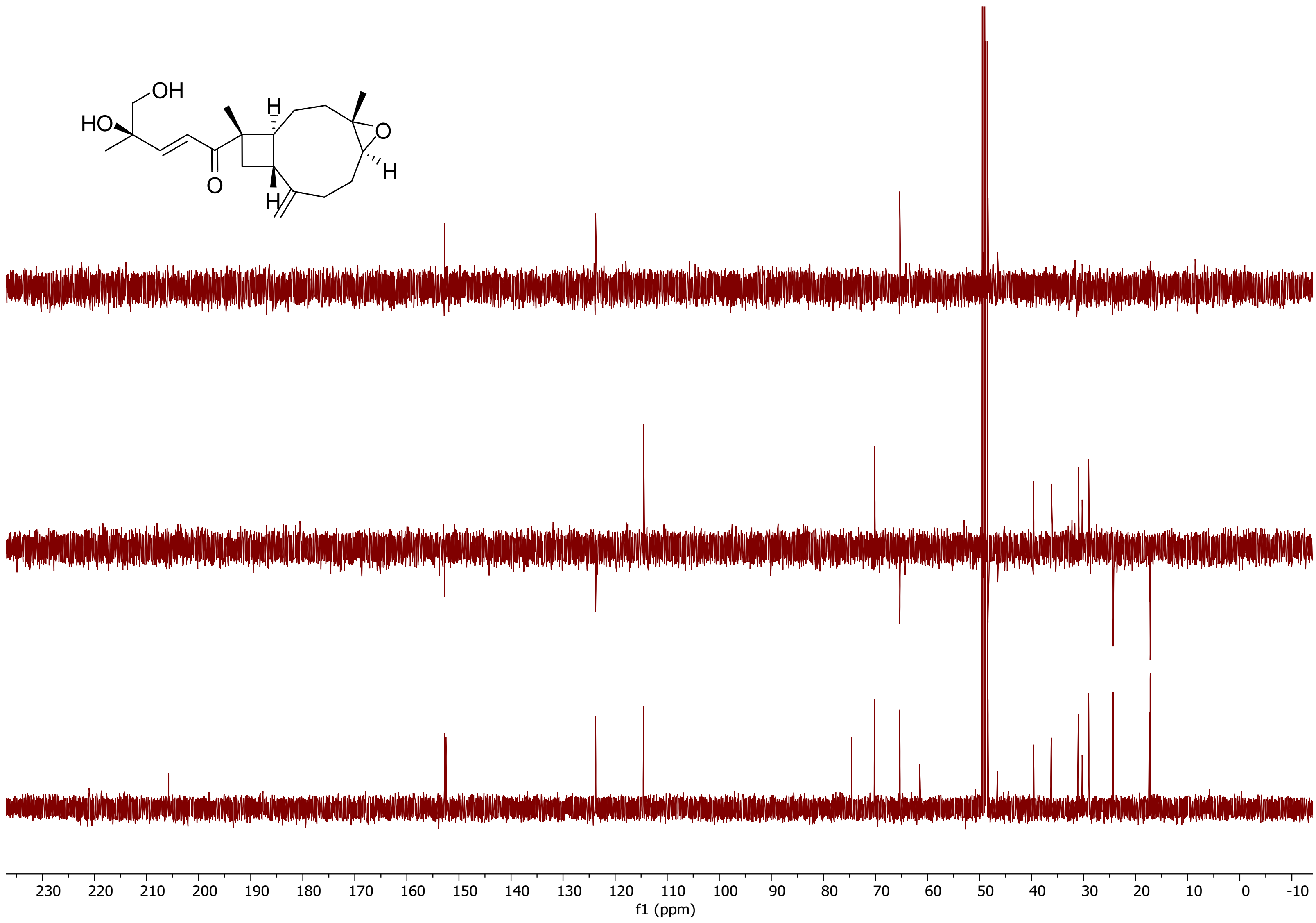

Figure S7. DEPT spectra of sclerohumin P (**1**) (150 MHz, CD_3OD).

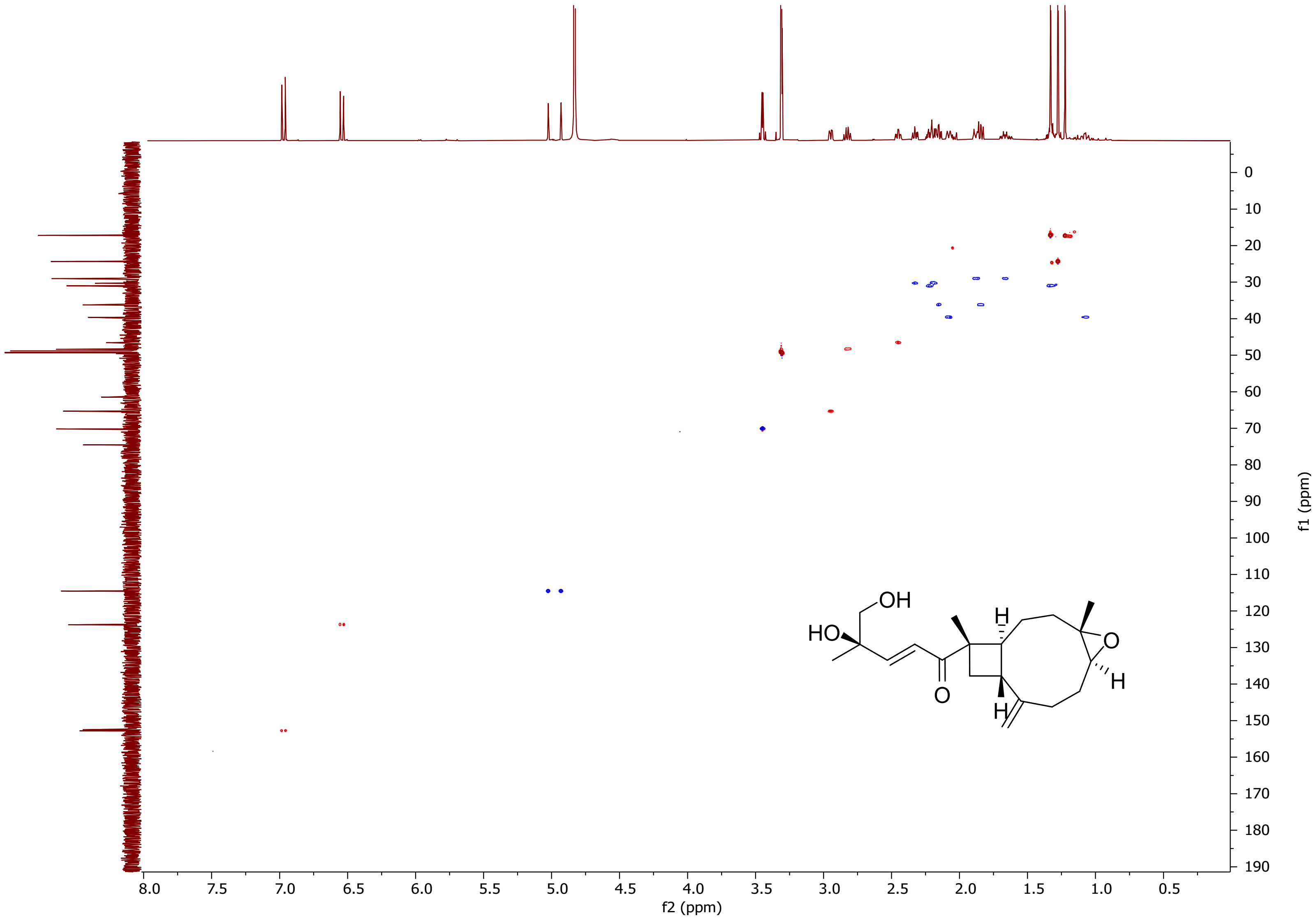

Figure S8. HSQC spectrum of sclerohumin P (**1**) (600 and 150 MHz, CD_3OD).

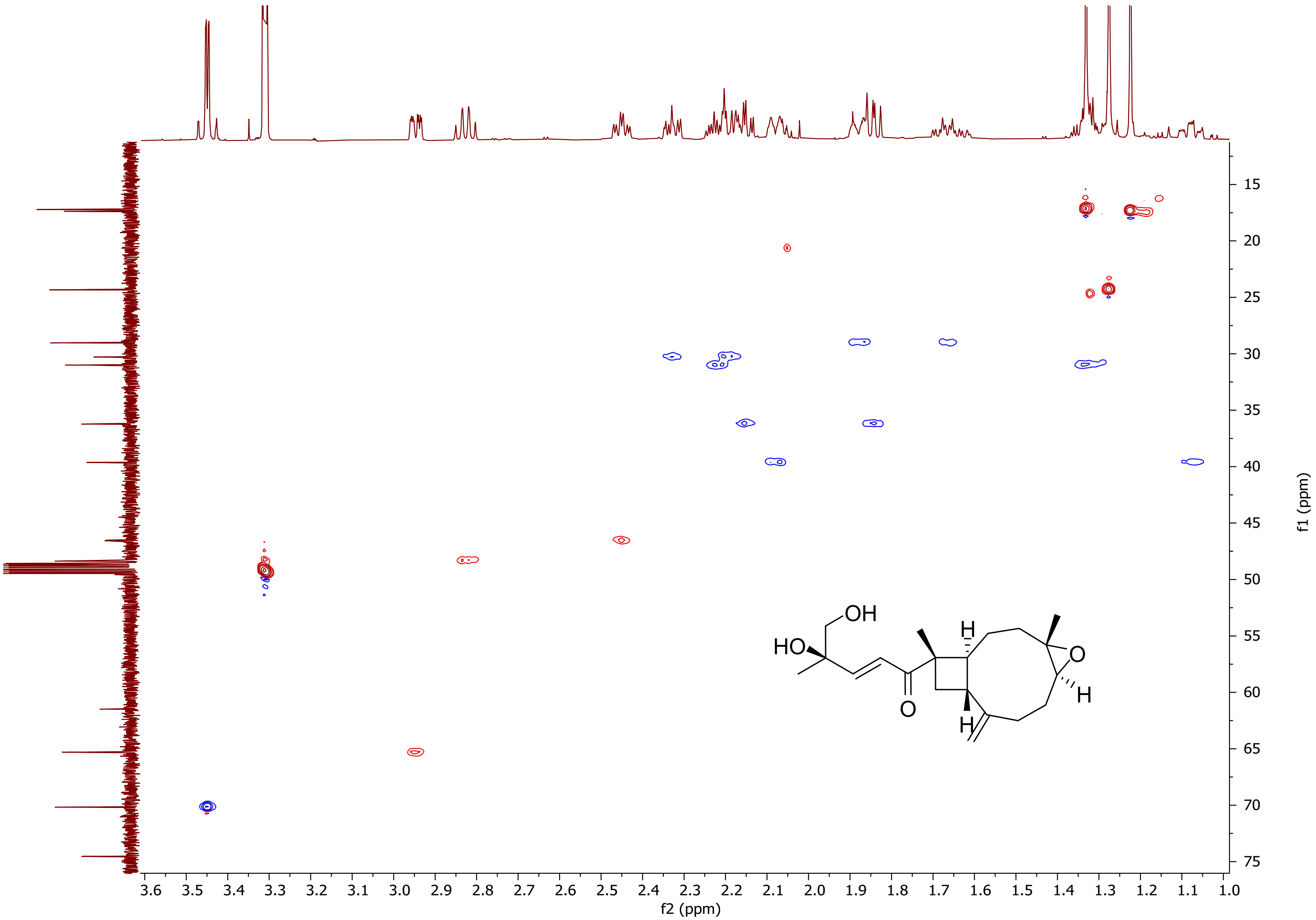

Figure S9. Expanded HSQC spectrum of sclerohumin P (**1**) (600 and 150 MHz, CD_3OD).

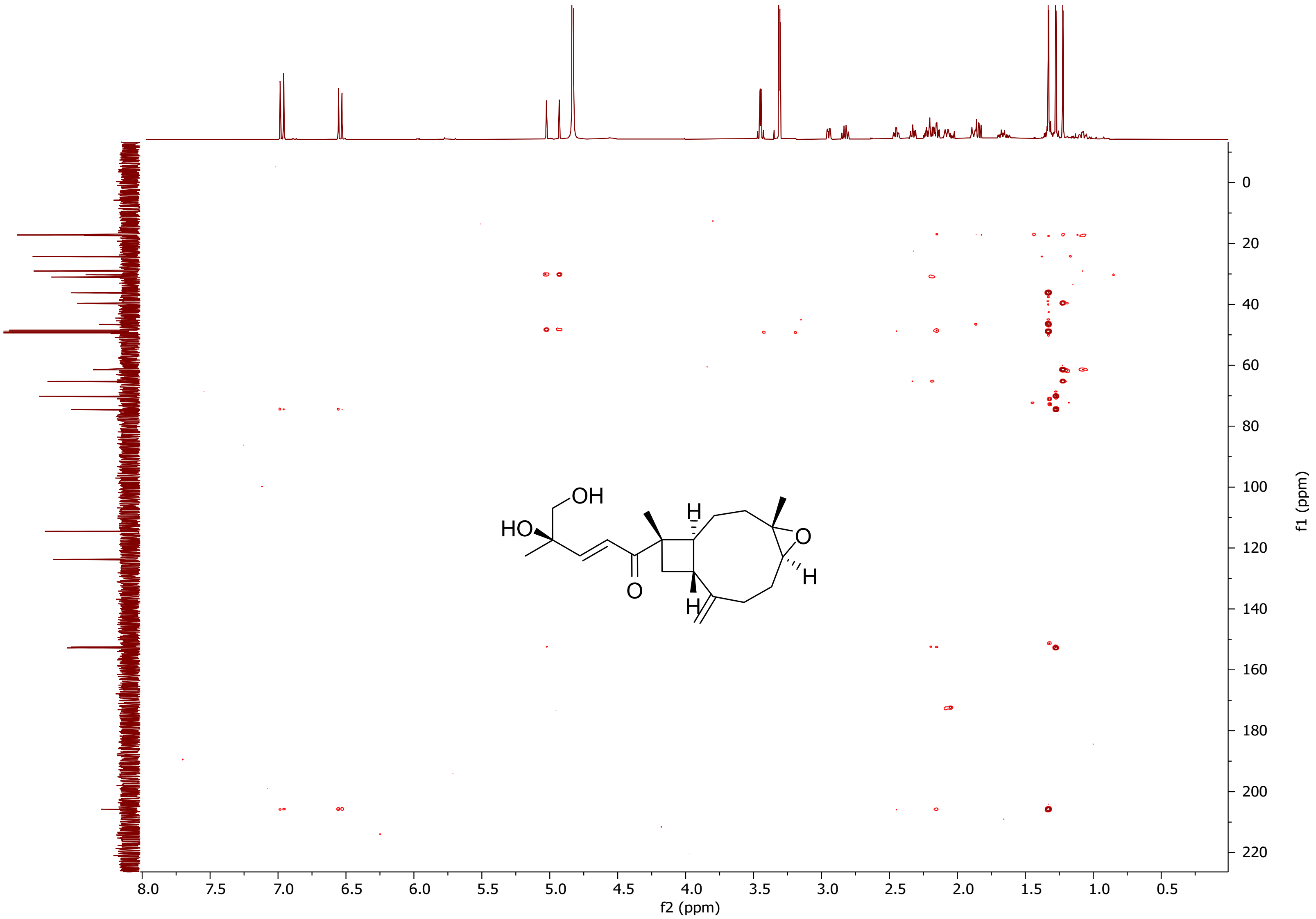

Figure S10. HMBC spectrum of sclerohumin P (**1**) (600 and 150 MHz, CD_3OD).

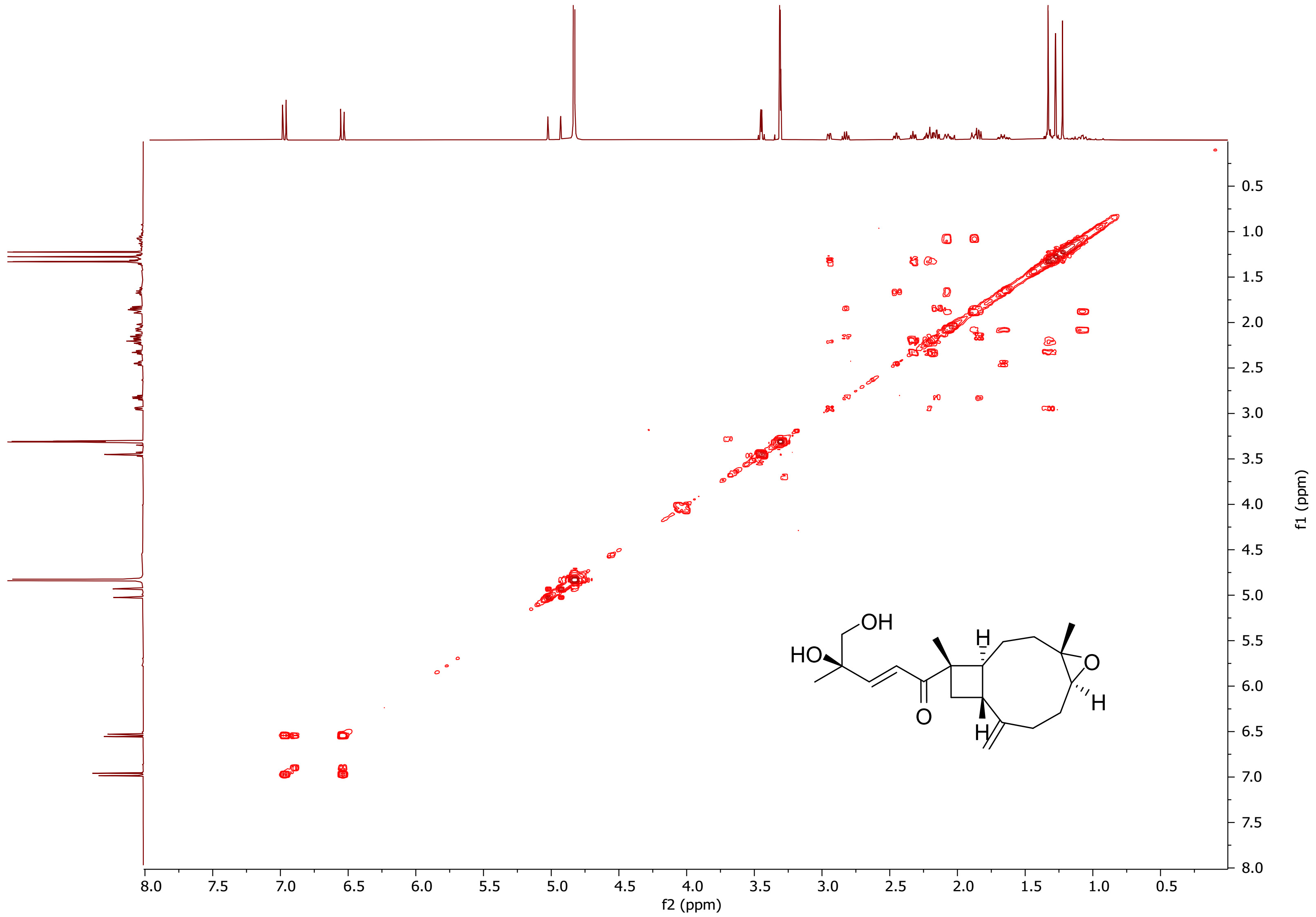

Figure S11. COSY spectrum of sclerohumin P (**1**) (600 MHz, CD_3OD).

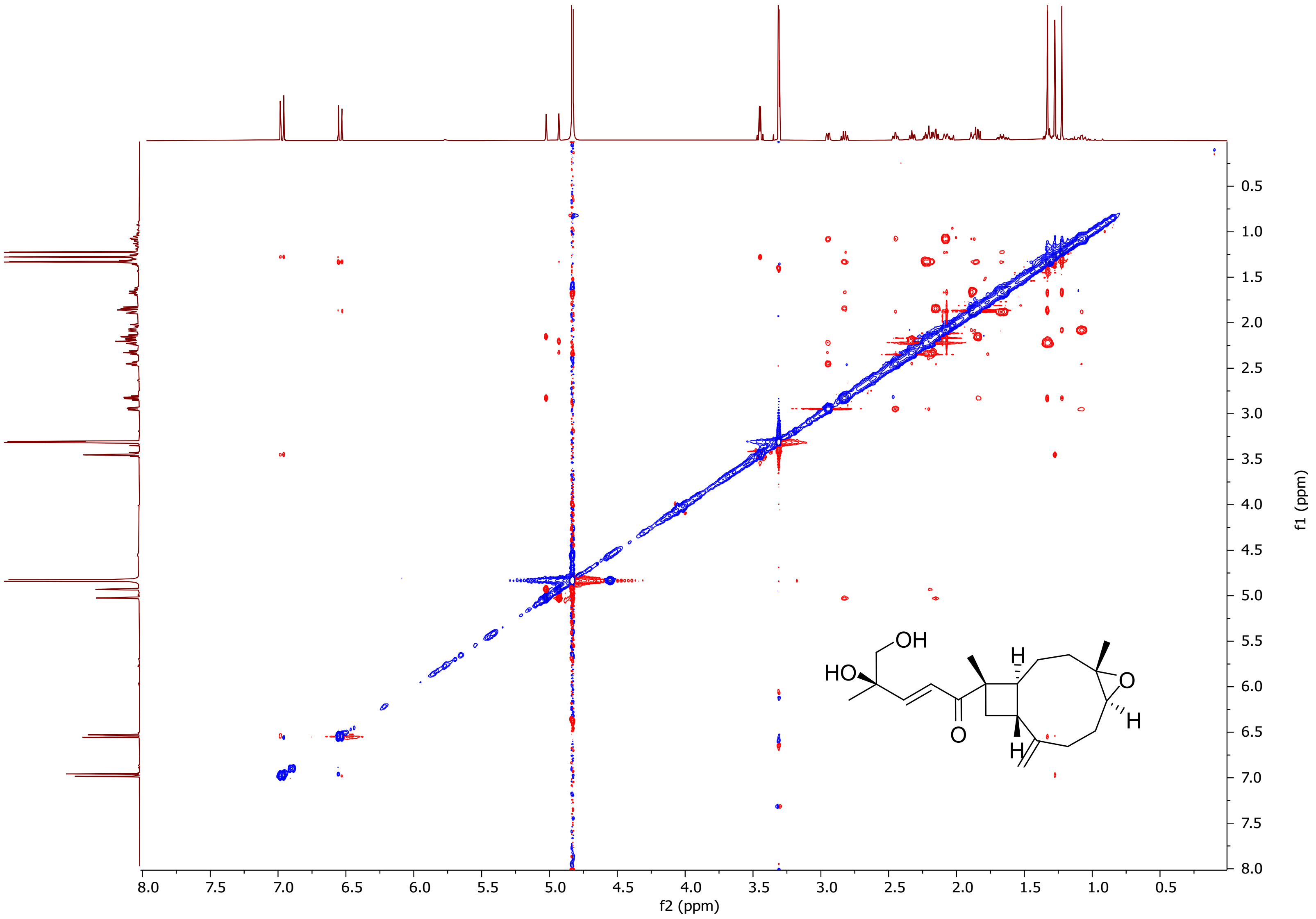

Figure S12. NOESY spectrum of sclerohumin P (**1**) (600 MHz, CD_3OD).

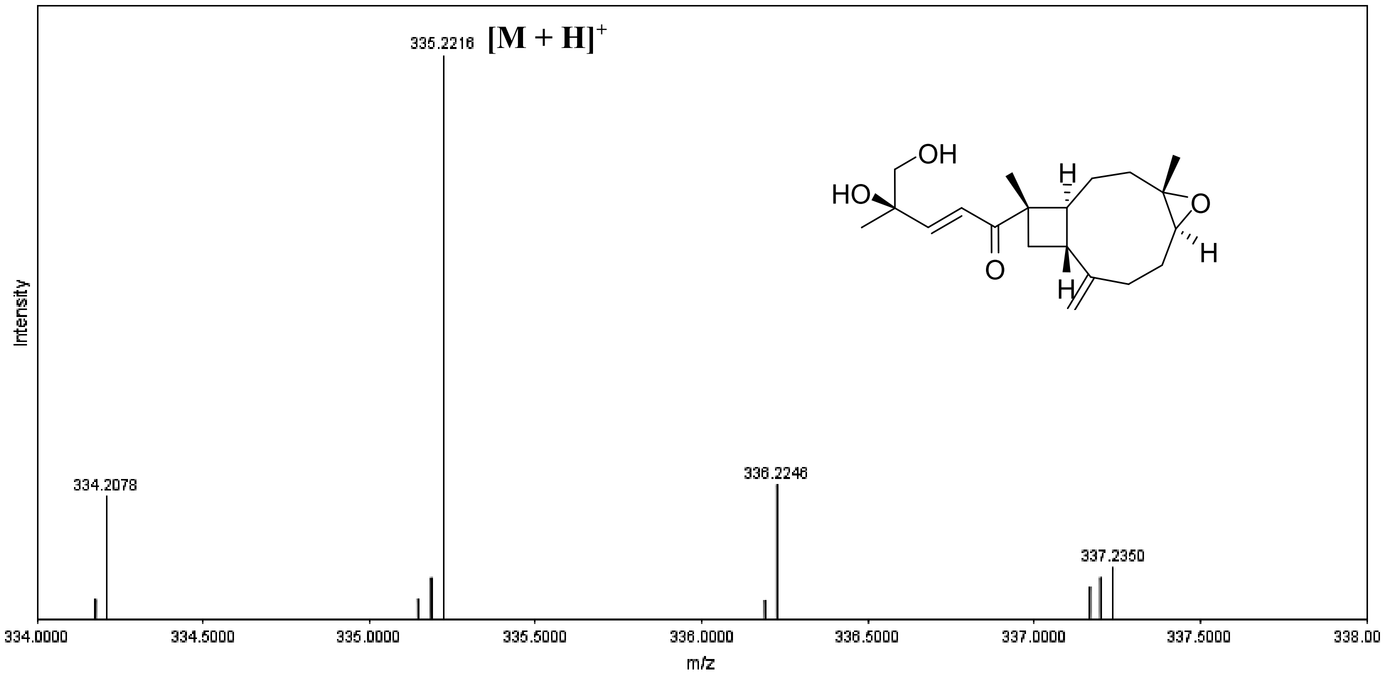

Hit	Formula	Calculated Mass	Target Mass	Error (mmu)	Error (ppm)
1	$C_{20}H_{31}O_4$	335.2222	335.2216	-0.6	-1.79

Figure S13. HRESIMS spectrum of sclerohumin P (**1**).

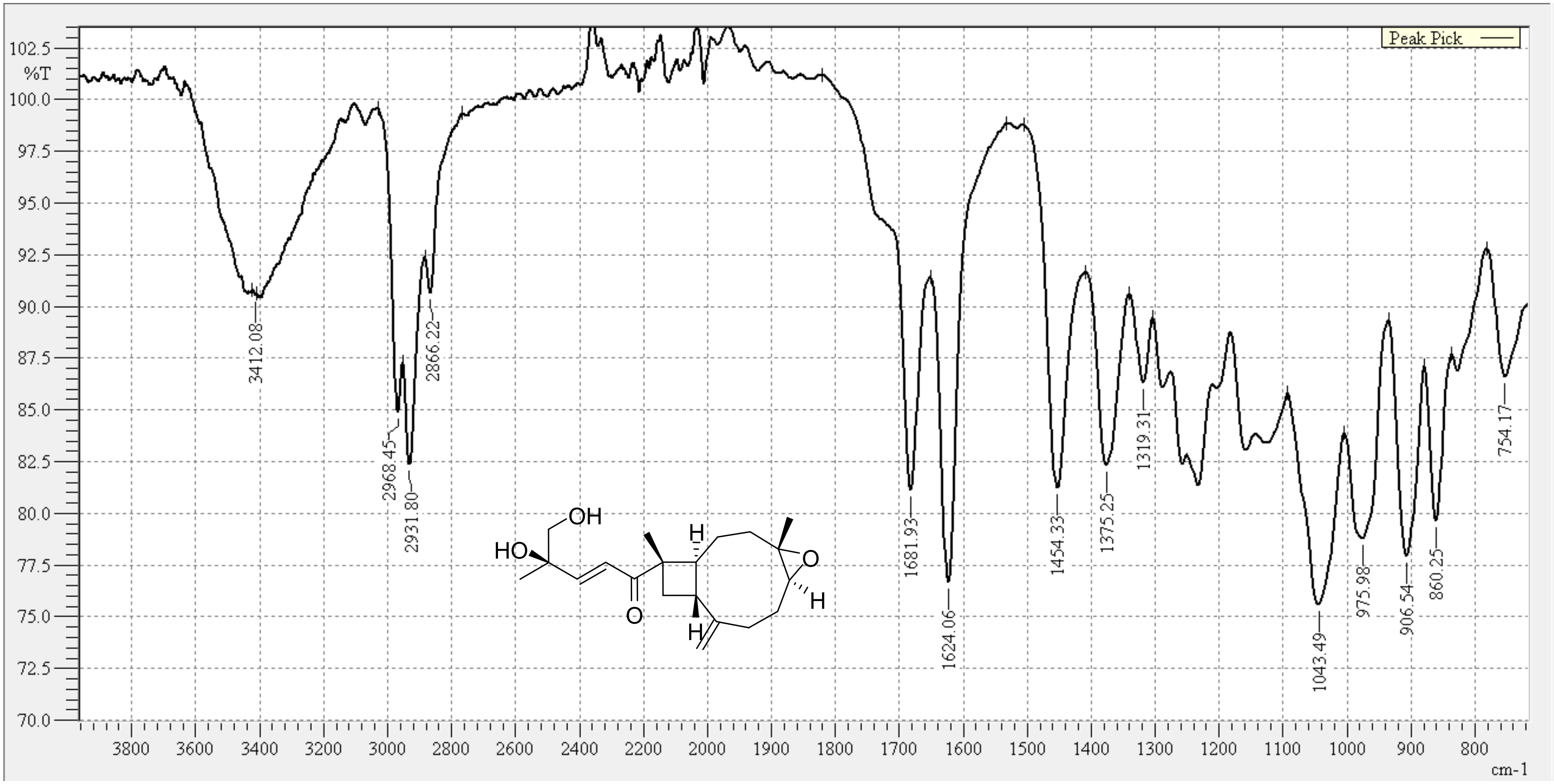

Figure S14. Infrared (IR) spectrum sclerohumin P (**1**).

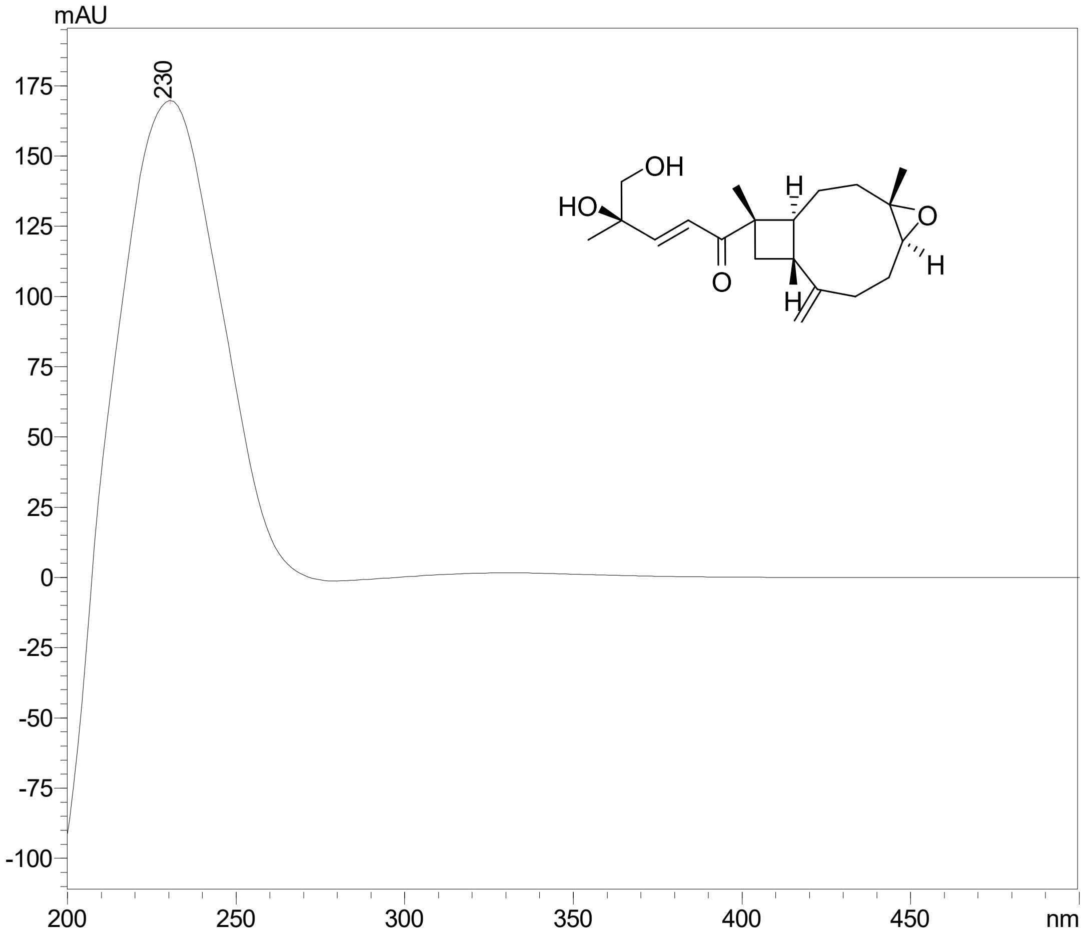

Figure S15. Ultraviolet (UV) spectrum sclerohumin P (**1**).

1.2. NMR, HRESIMS IR, and UV spectra of sclerohumin Q (2)

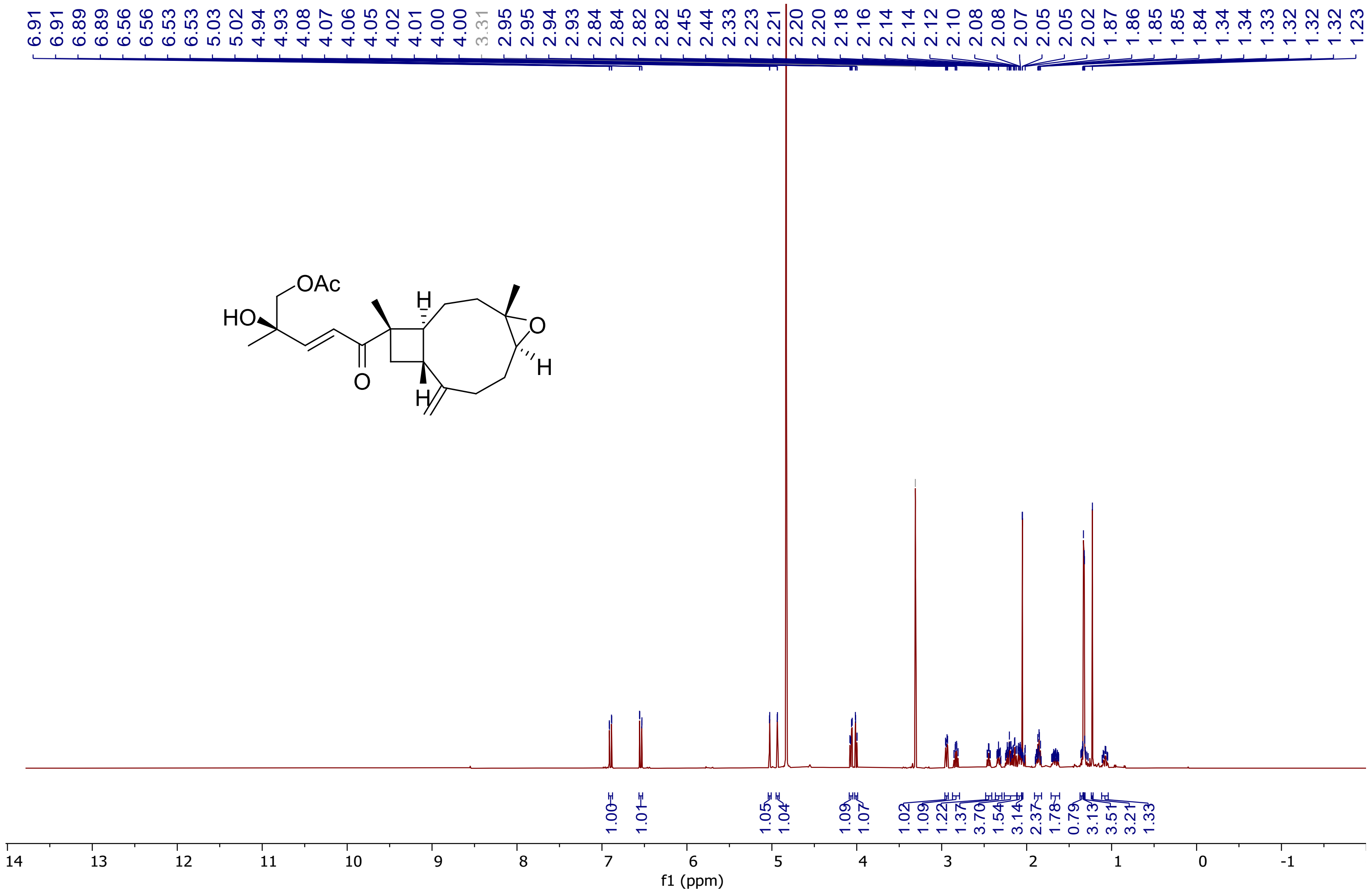

Figure S16. ^{1}H NMR spectrum of sclerohumin Q (**2**) (600 MHz, CD_3OD).

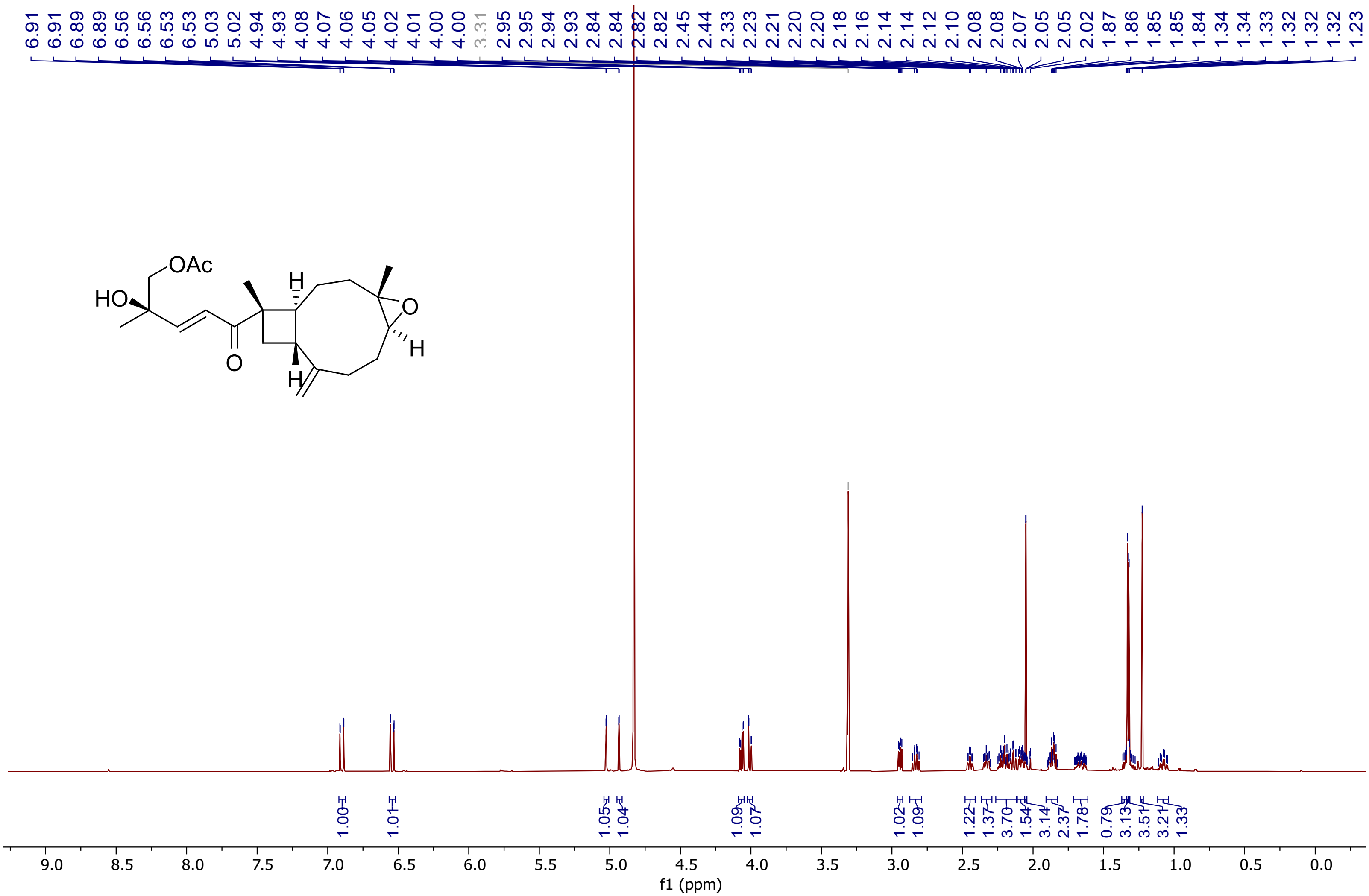

Figure S17. Expanded ^{1}H NMR spectrum of sclerohumin Q (**2**) (600 MHz, CD_3OD).

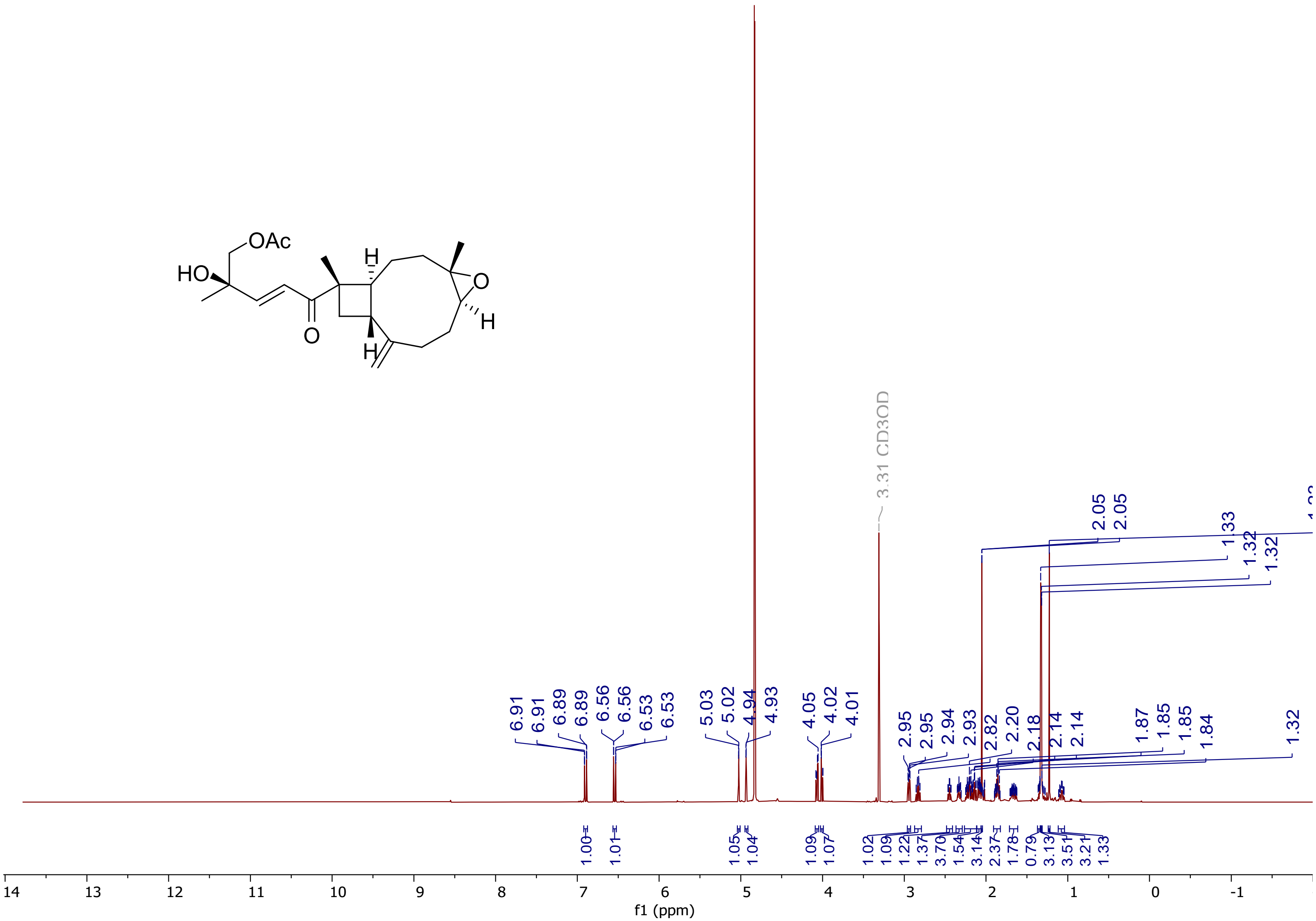

Figure S18. ^{1}H NMR spectrum of sclerohumin Q (**2**) with chemical shift values displayed above the signals (600 MHz, CD_3OD).

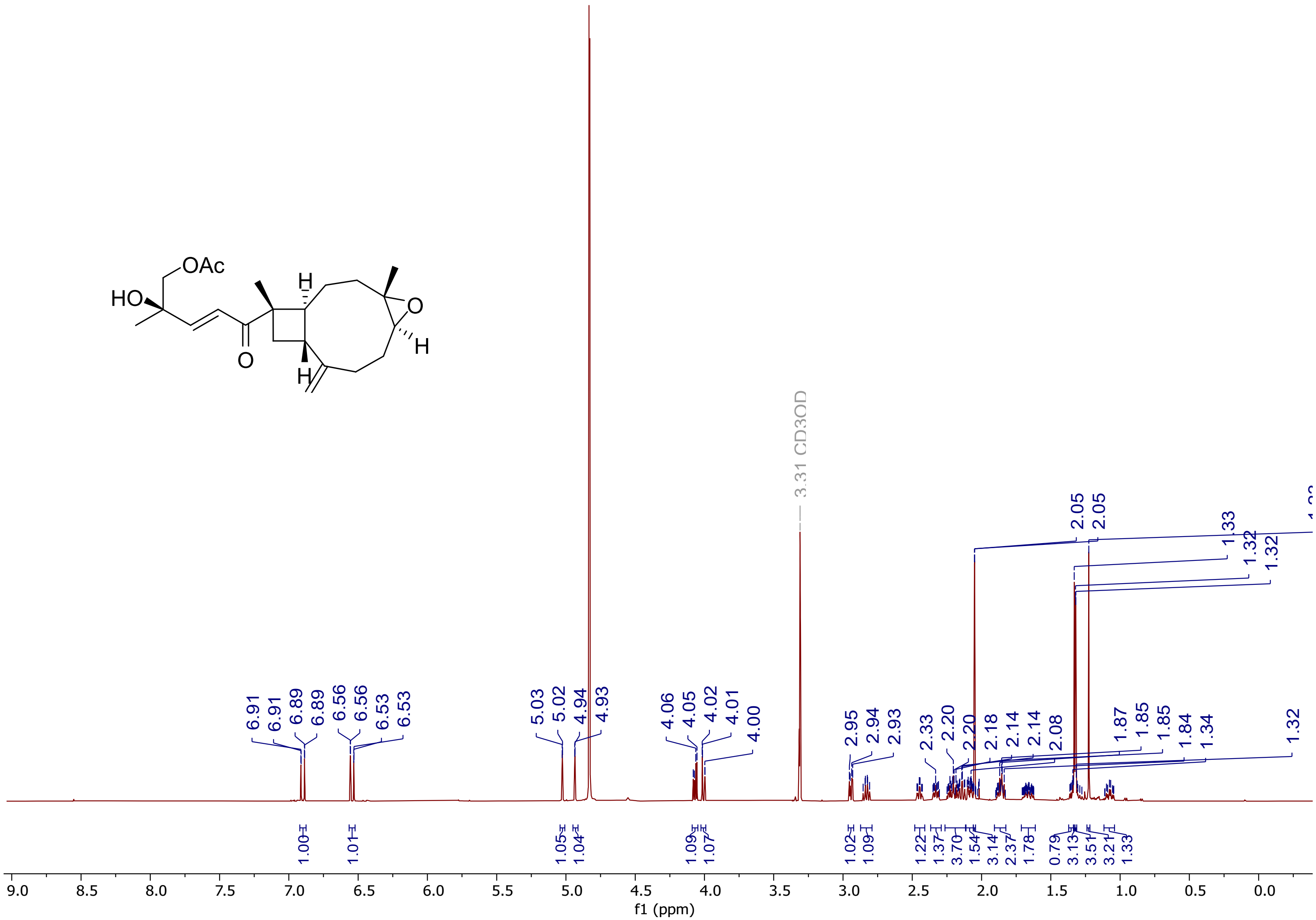

Figure S19. Expanded ^{1}H NMR spectrum of sclerohumin Q (**2**) with chemical shift values displayed above the signals (600 MHz, CD_3OD).

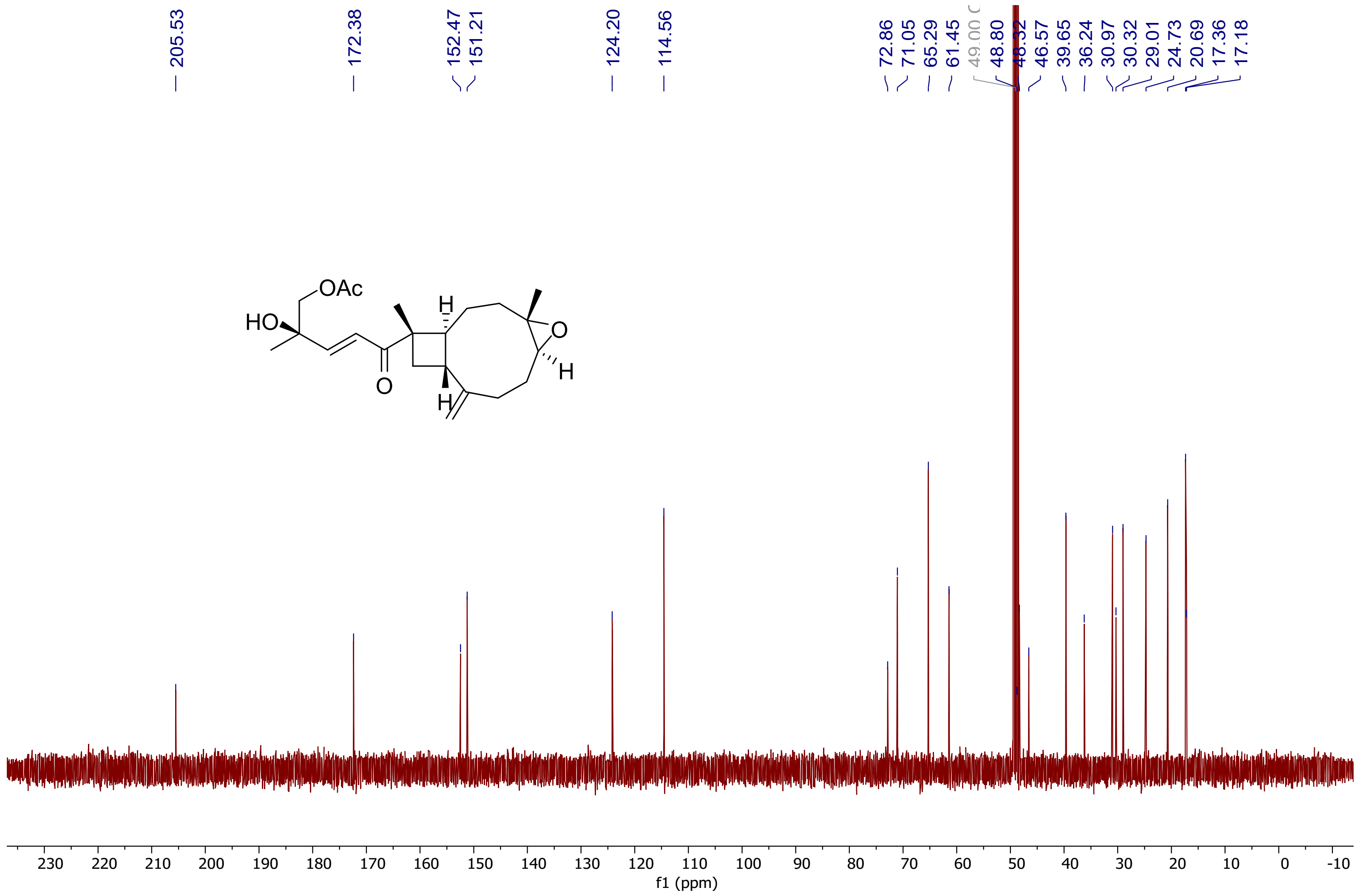

Figure S20. ^{13}C NMR spectrum of sclerohumin Q (**2**) (150 MHz, CD_3OD).

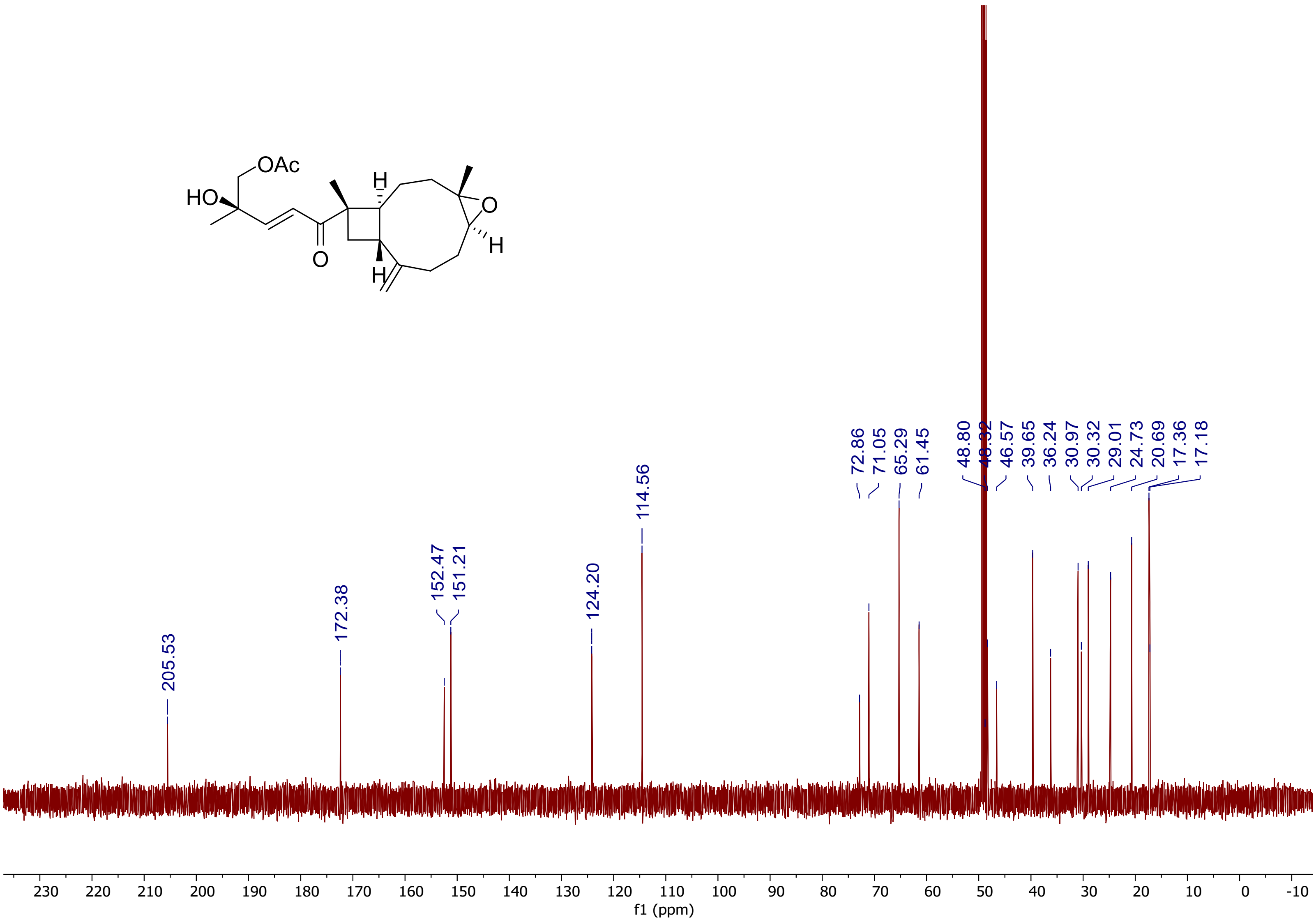

Figure S21. ^{13}C NMR spectrum of sclerohumin Q (**2**) with chemical shift values displayed above the signals (150 MHz, CD_3OD).

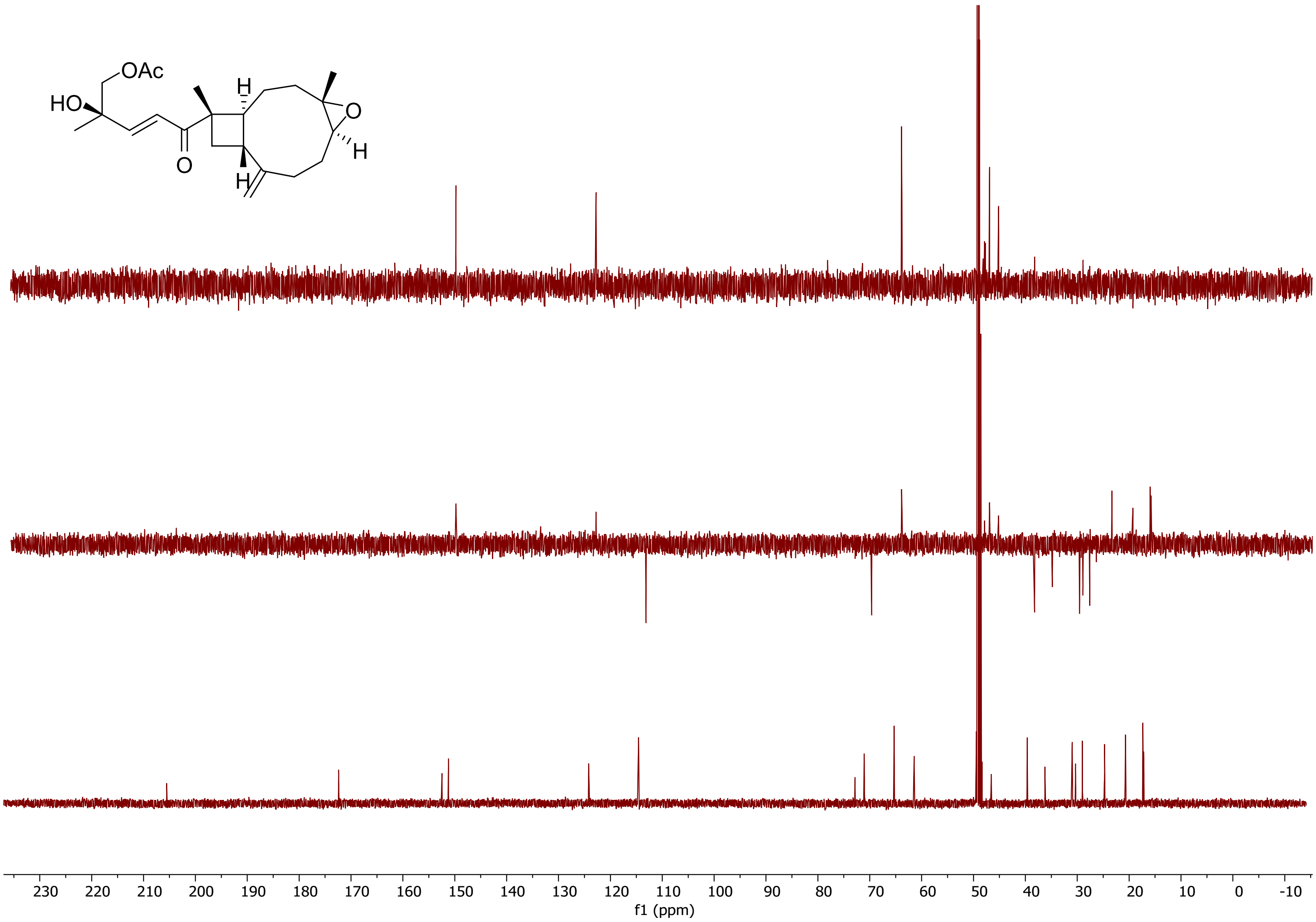

Figure S22. DEPT spectra of sclerohumin Q (**2**) (150 MHz, CD_3OD).

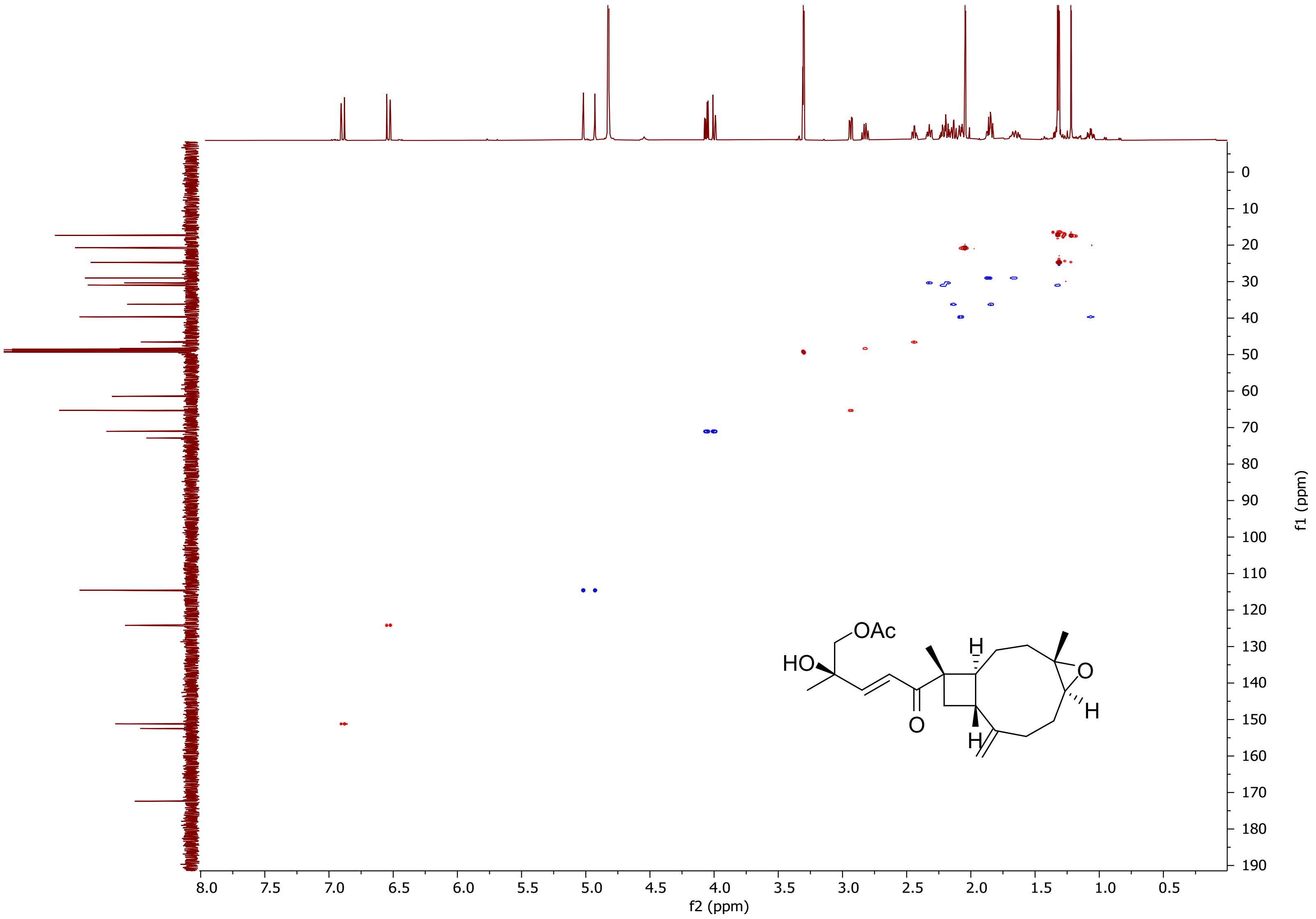

Figure S23. HSQC spectrum of sclerohumin Q (**2**) (600 and 150 MHz, CD_3OD).

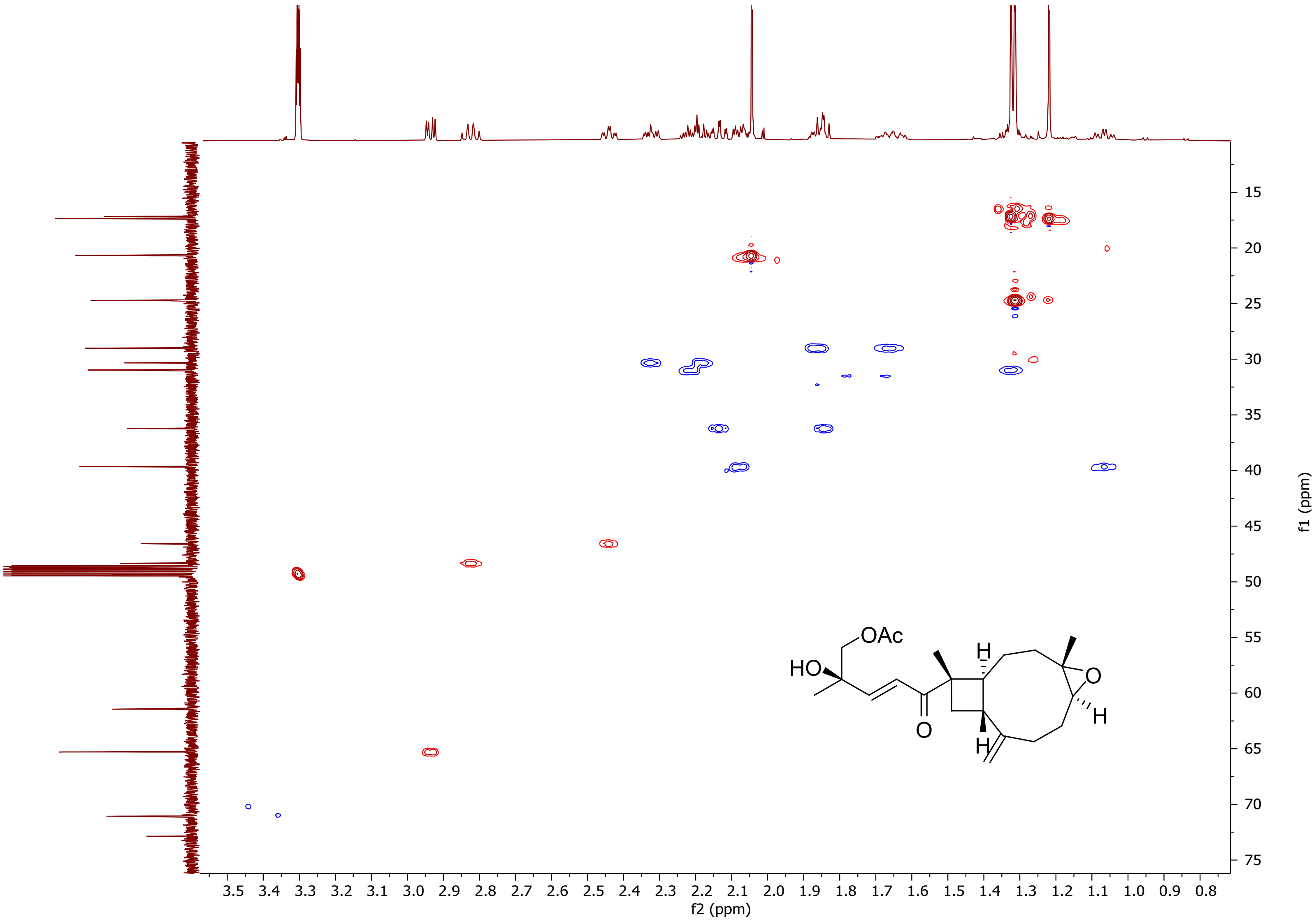

Figure S24. Expanded HSQC spectrum of sclerohumin Q (**2**) (600 and 150 MHz, CD_3OD).

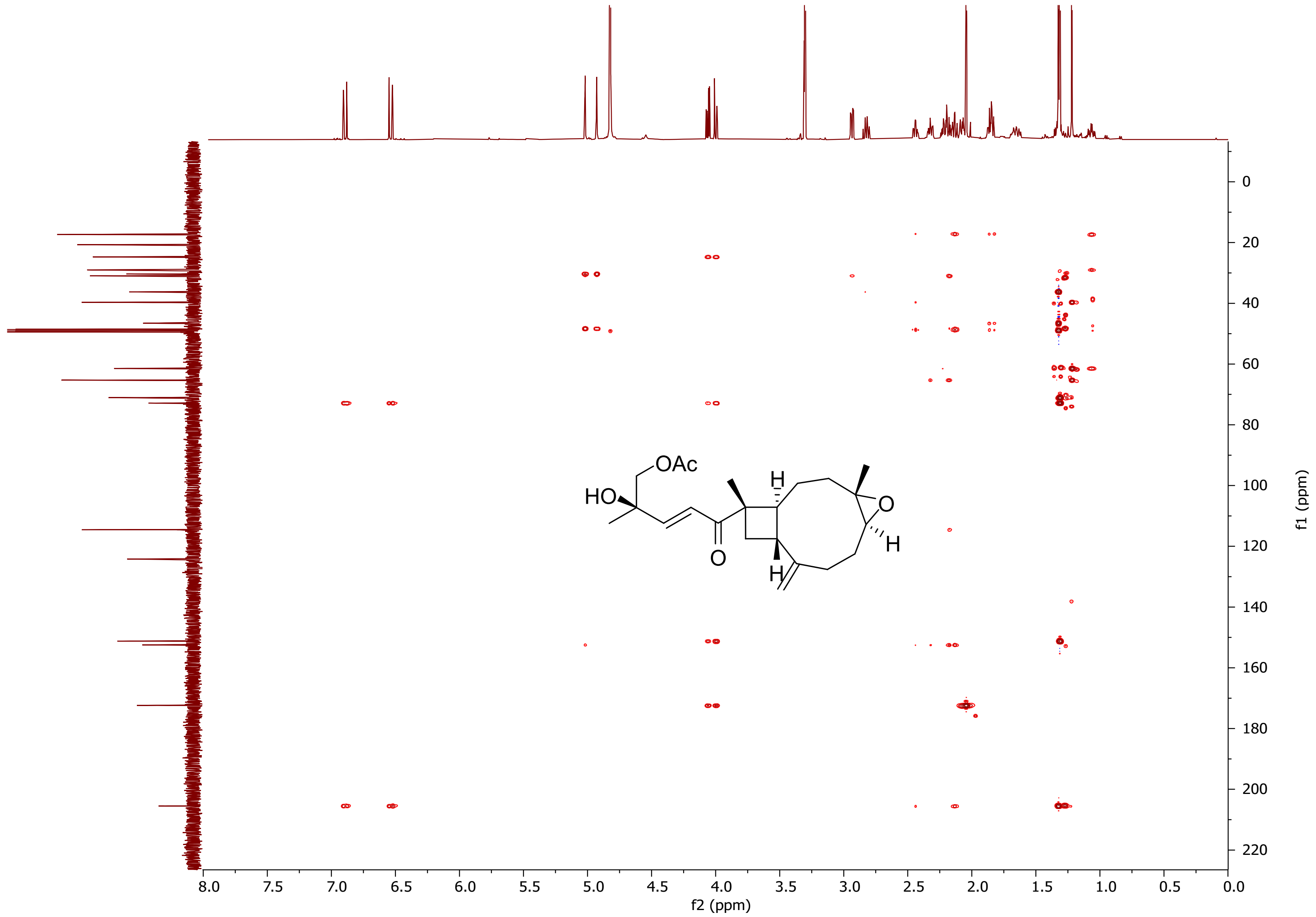

Figure S25. HMBC spectrum of sclerohumin Q (**2**) (600 and 150 MHz, CD_3OD).

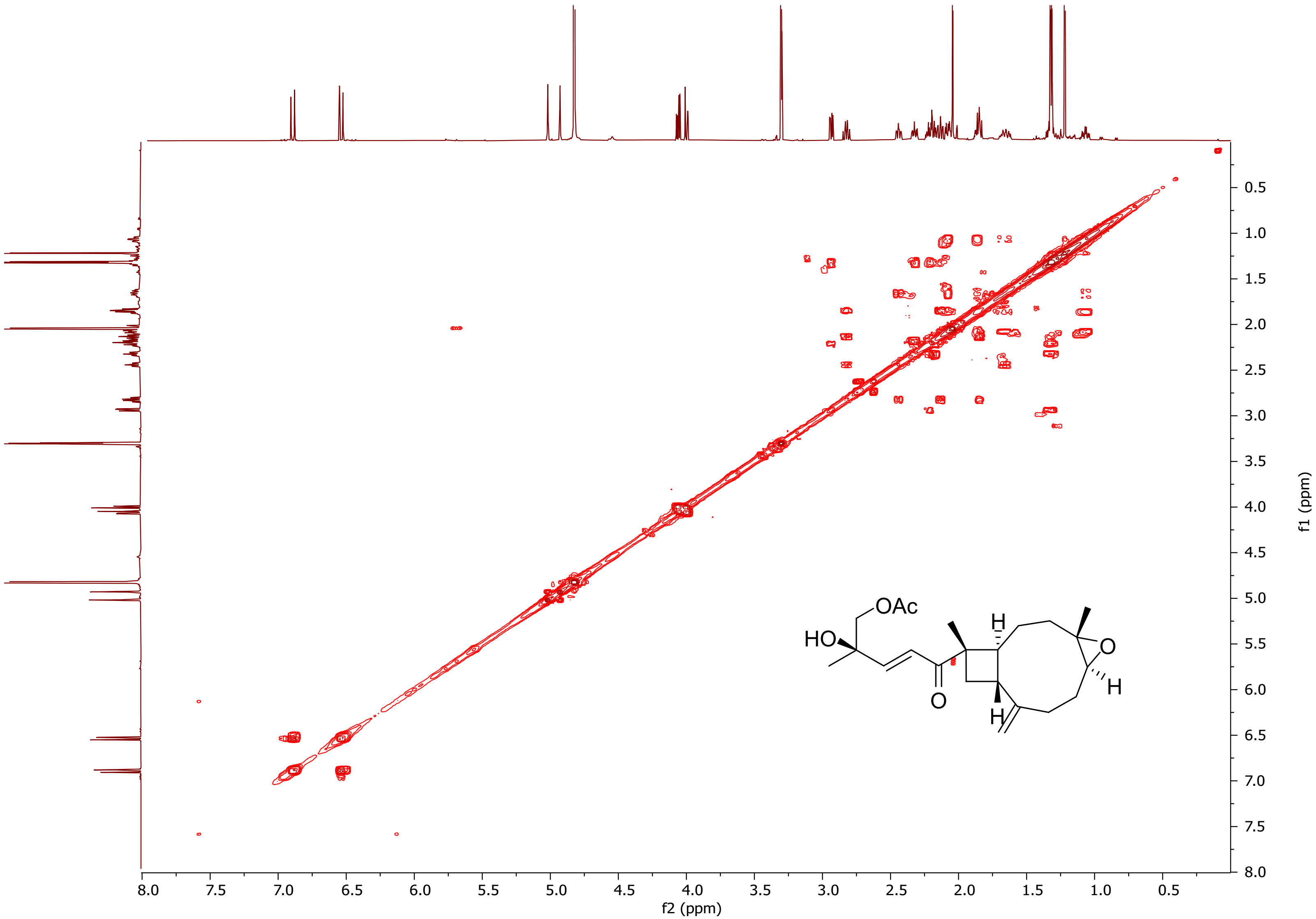

Figure S26. COSY spectrum of sclerohumin Q (**2**) (600 MHz, CD_3OD).

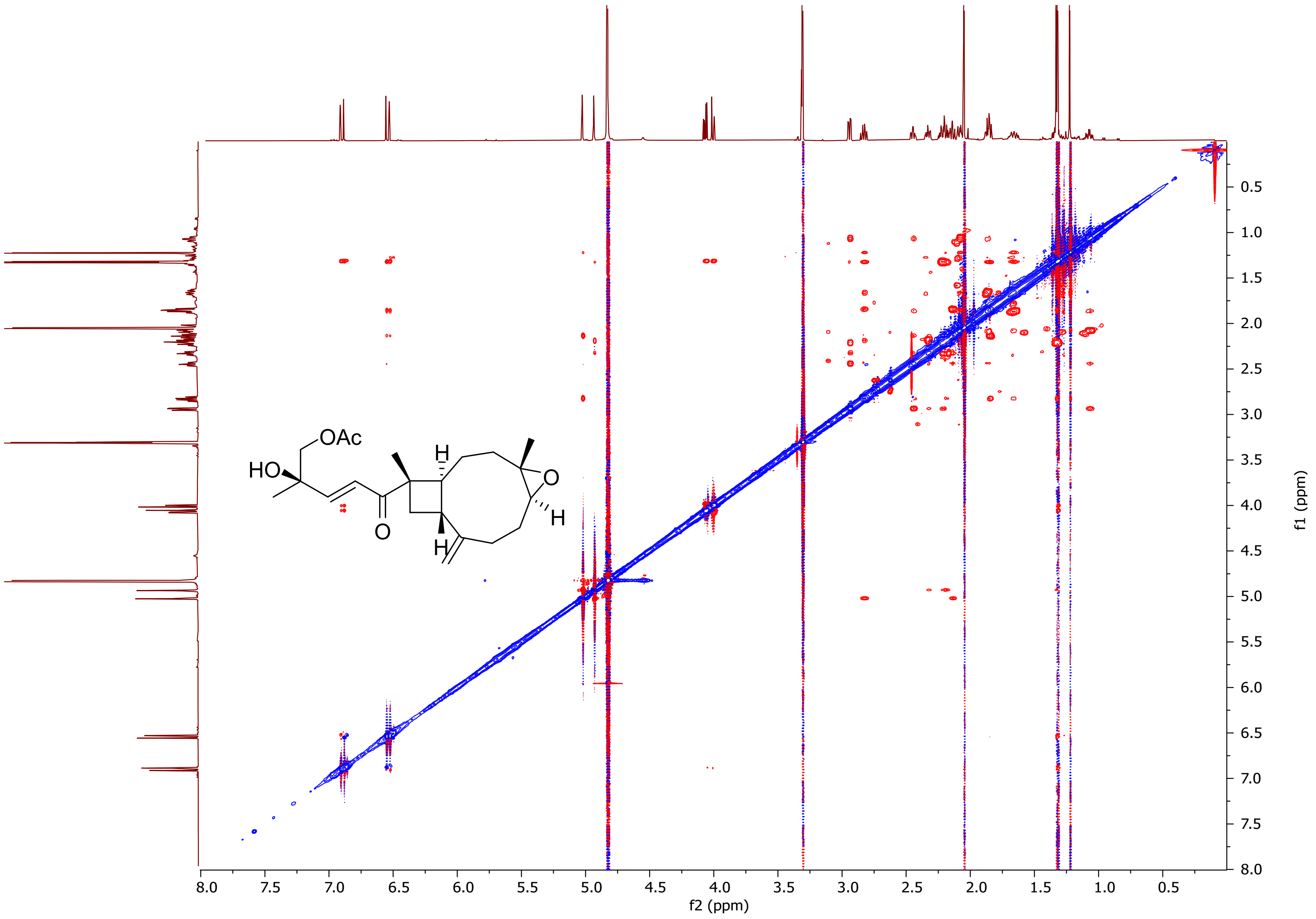

Figure S27. NOESY spectrum of sclerohumin Q (**2**) (600 MHz, CD_3OD).

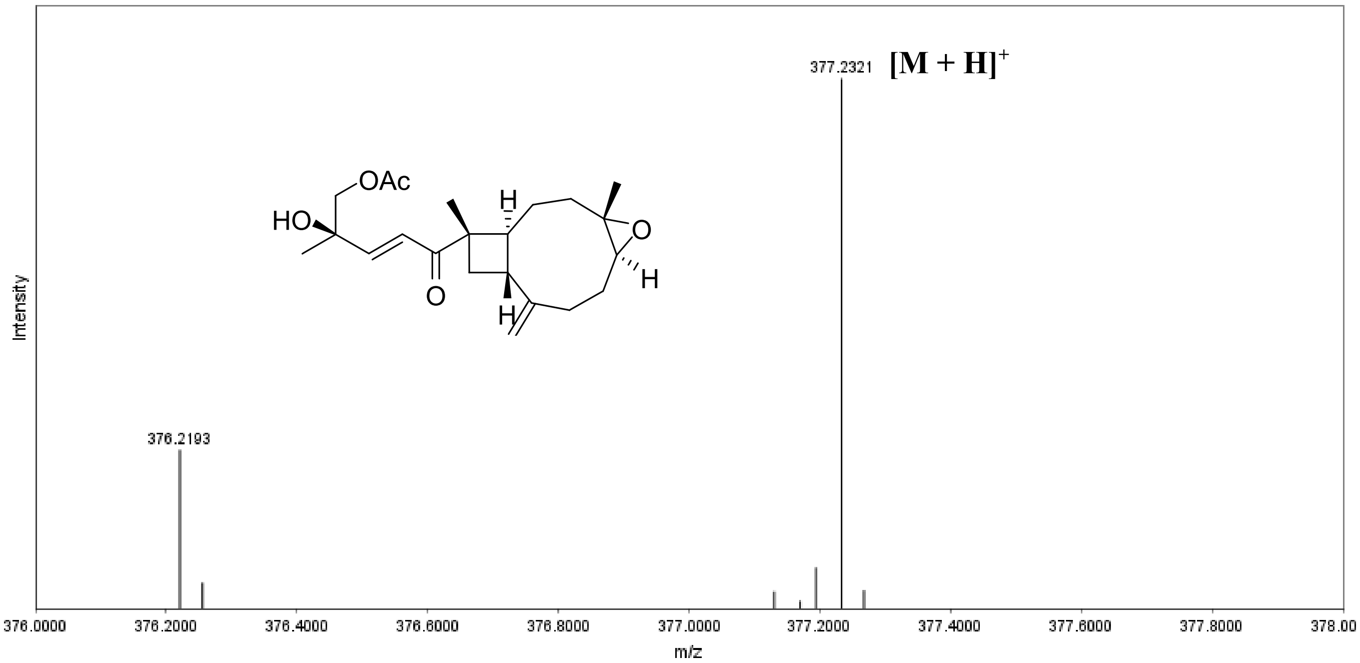

Hit	Formula	Calculated Mass	Target Mass	Error (mmu)	Error (ppm)
1	$C_{22}H_{33}O_5$	377.2328	377.2321	-0.7	-1.86

Figure S28. HRESIMS spectrum of sclerohumin Q (**2**).

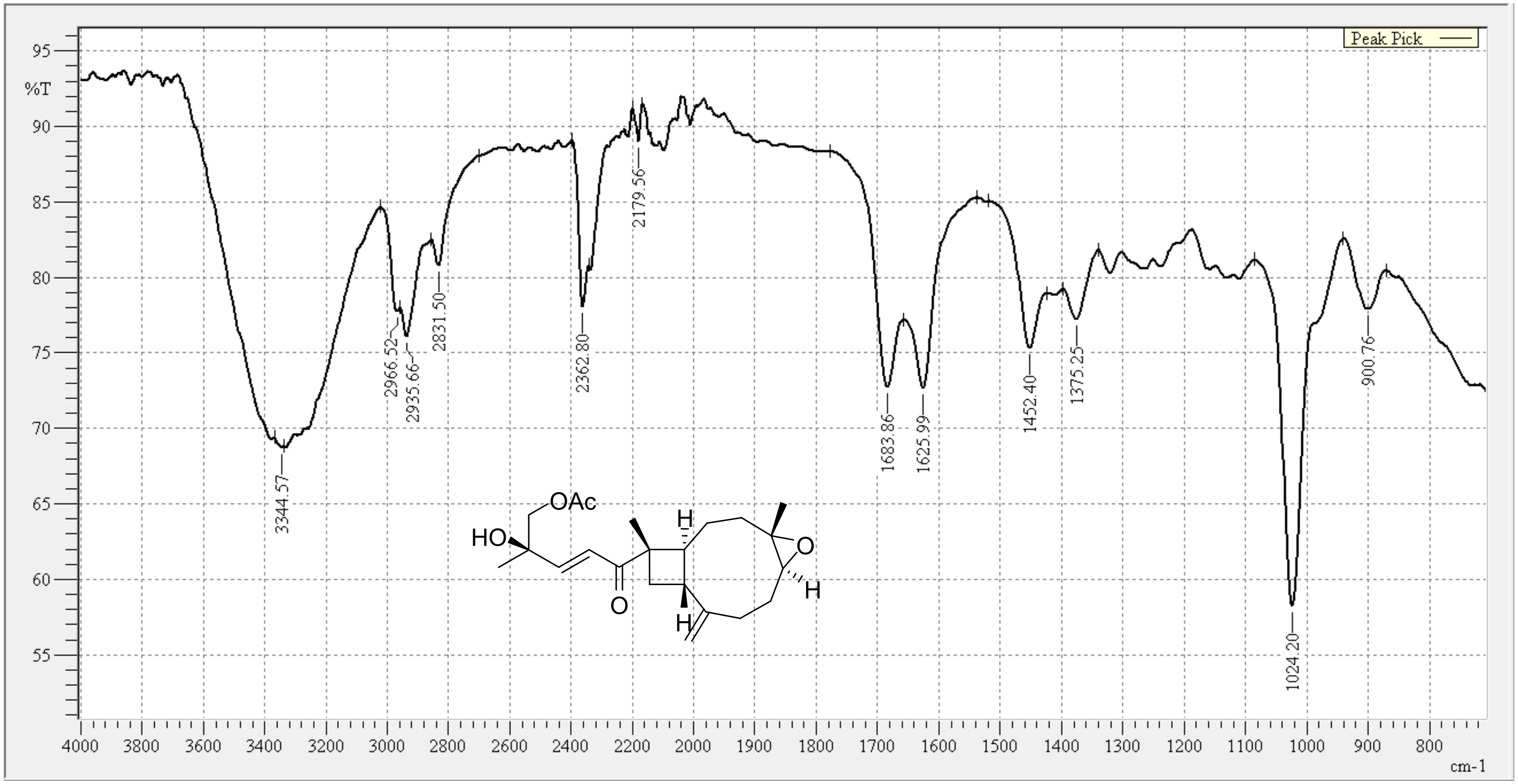

Figure S29. Infrared (IR) spectrum sclerohumin Q (**2**).

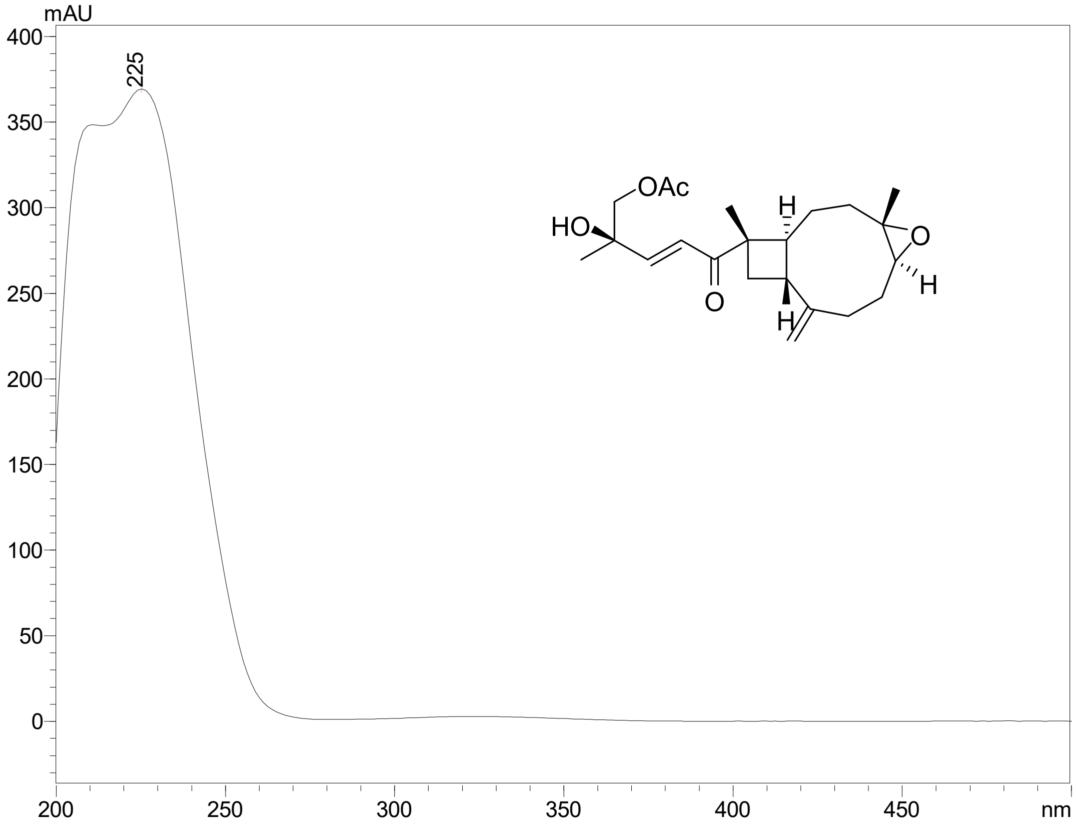

Figure S30. Ultraviolet (UV) spectrum sclerohumin Q (**2**).

1.3. NMR, HRESIMS, IR, and UV spectra of sclerohumin R (3)

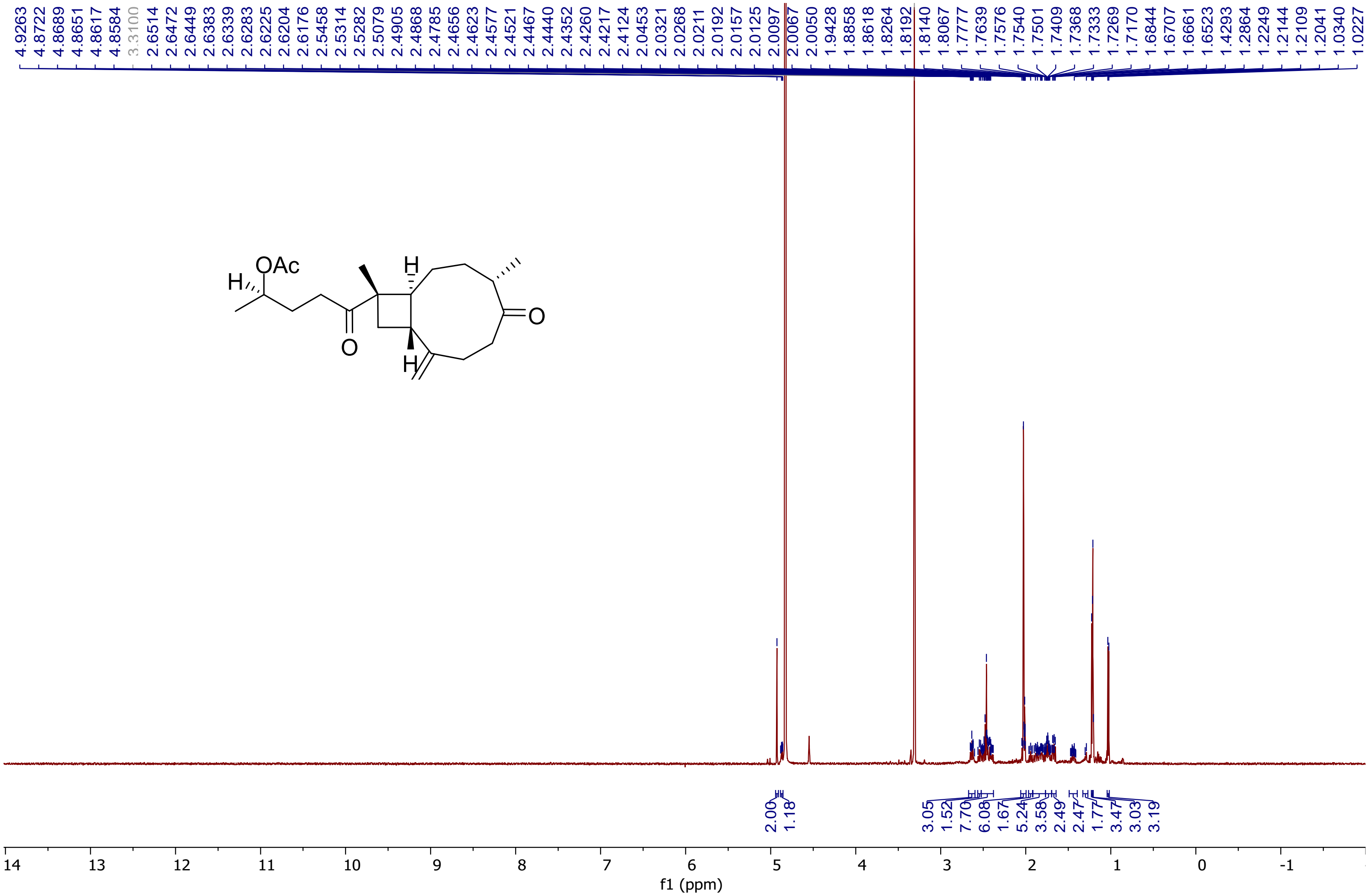

Figure S31. ^1H NMR spectrum of sclerohumin R (**3**) (600 MHz, CD_3OD).

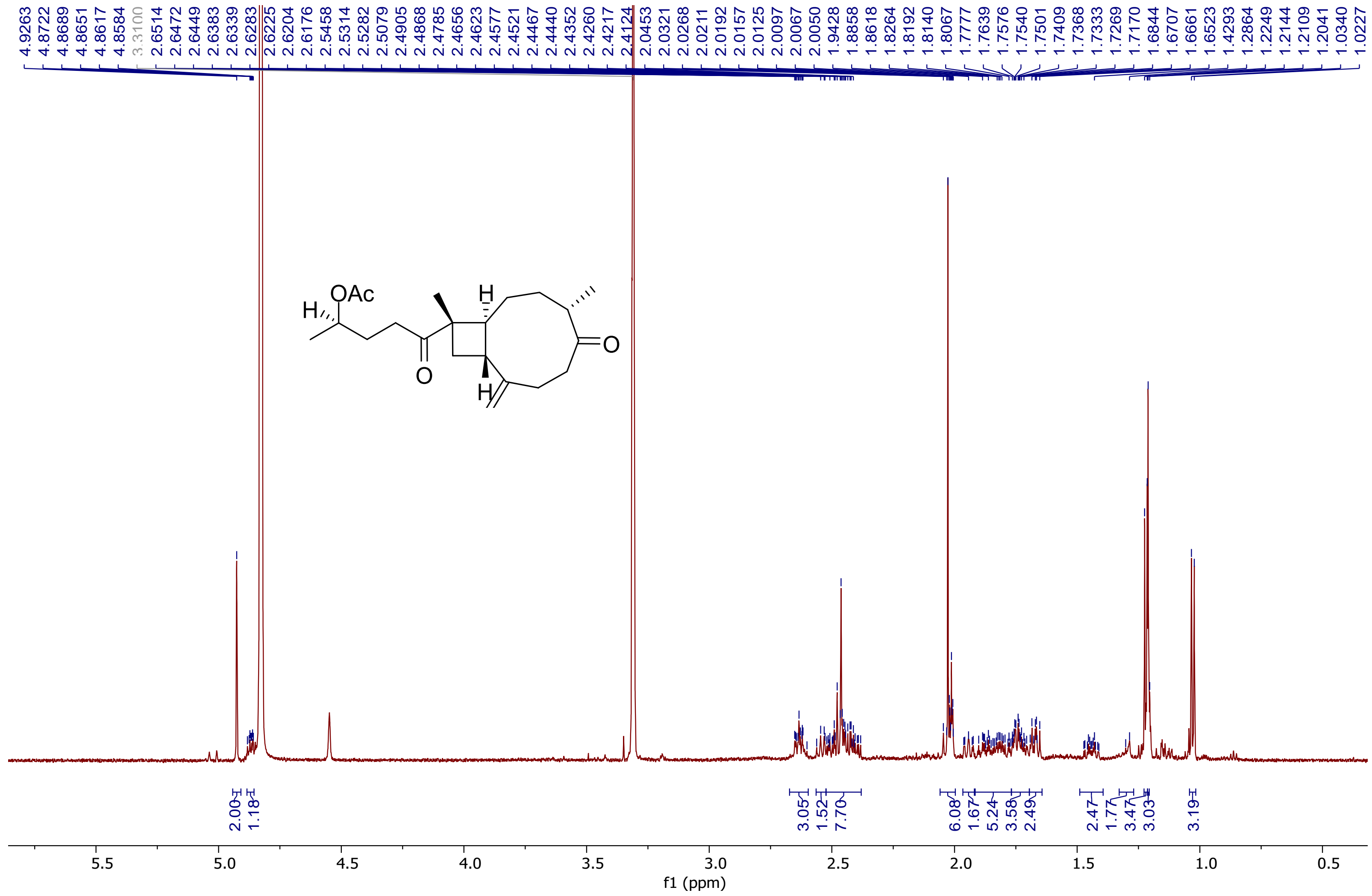

Figure S32. Expanded ^{1}H NMR spectrum of sclerohumin R (**3**) (600 MHz, CD_3OD).

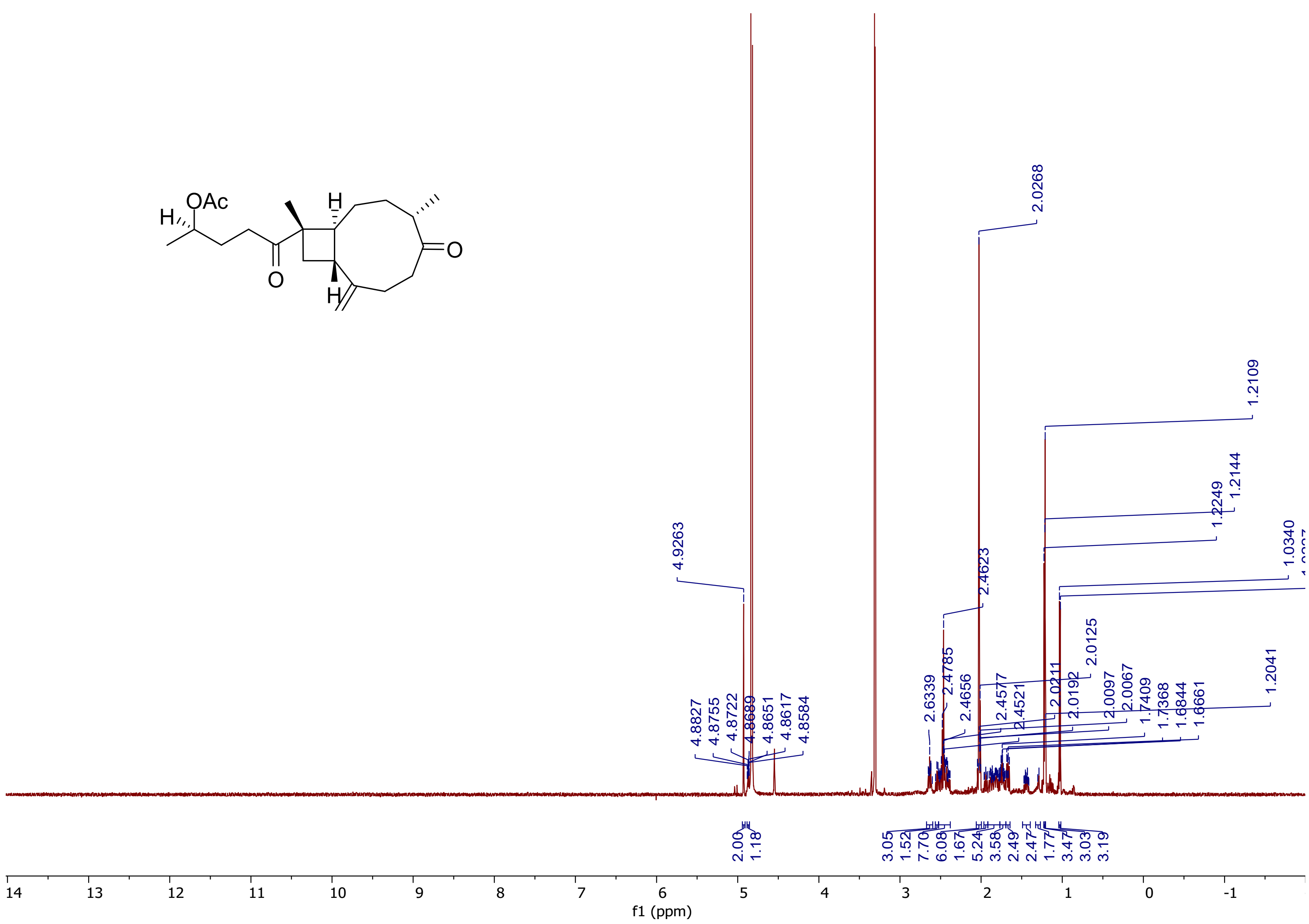

Figure S33. ^{1}H NMR spectrum of sclerohumin R (**3**) with chemical shift values displayed above the signals (600 MHz, CD_3OD).

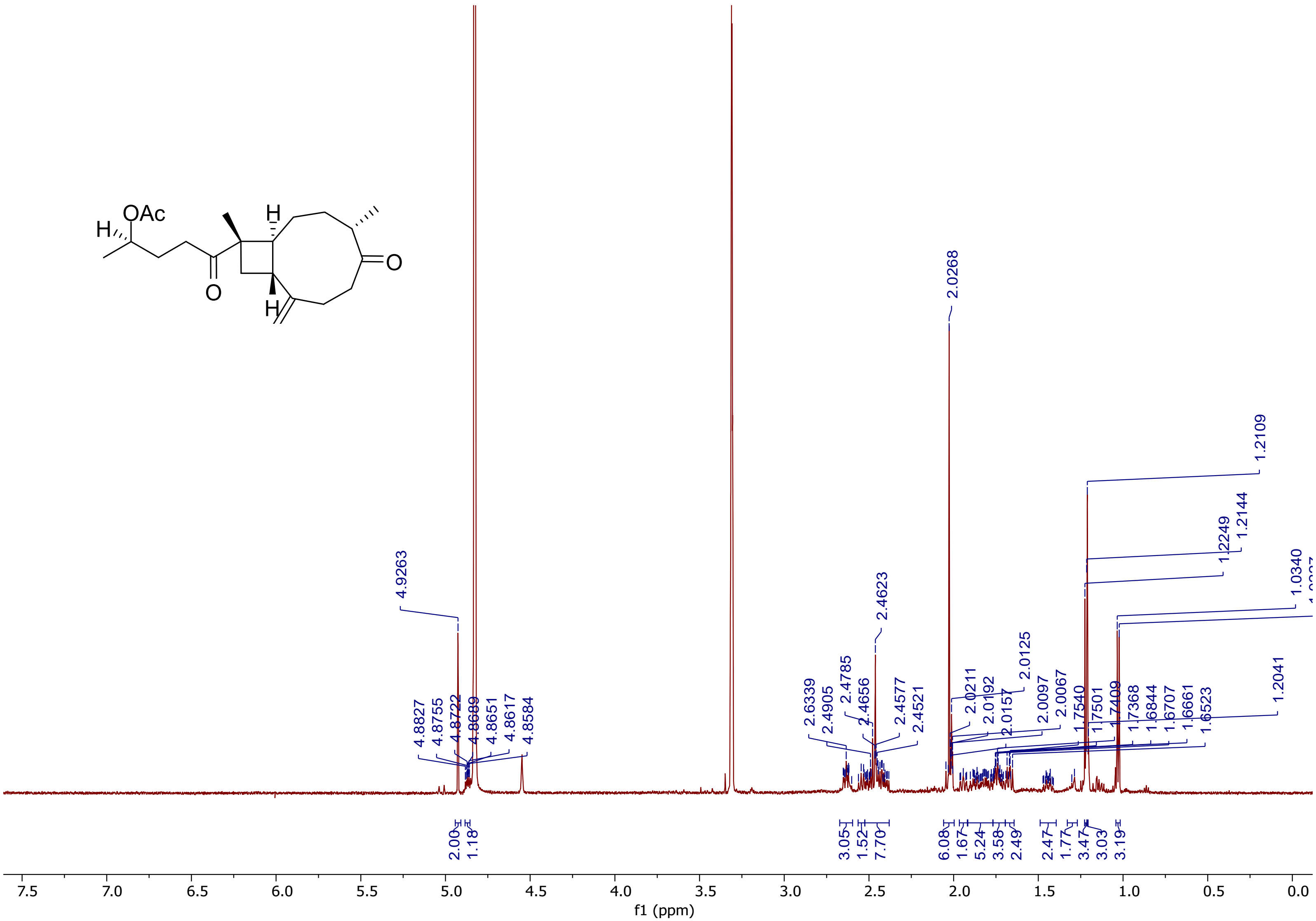

Figure S34. Expanded ^{1}H NMR spectrum of sclerohumin R (**3**) with chemical shift values displayed above the signals (600 MHz, CD_3OD).

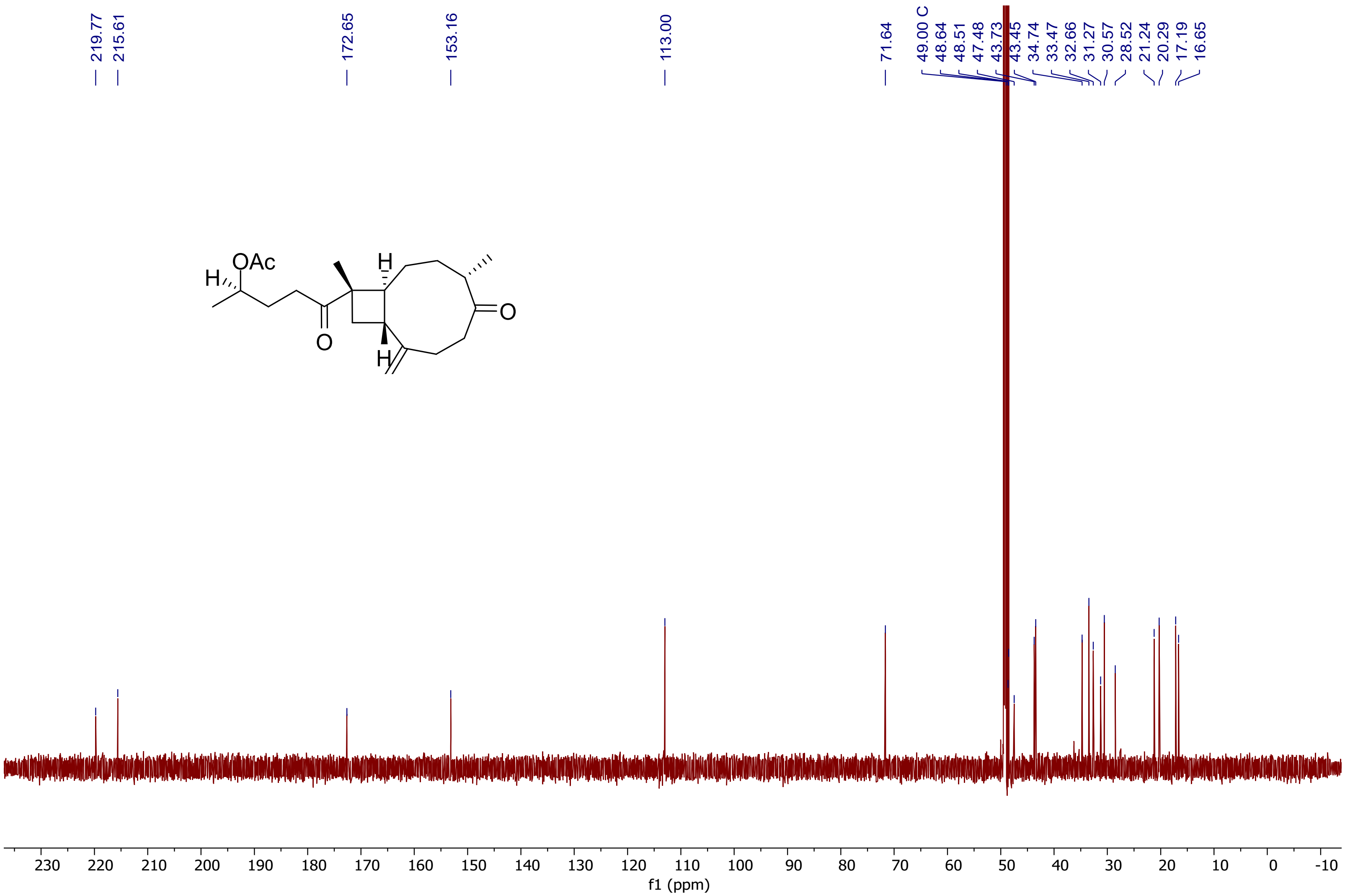

Figure S35. ^{13}C NMR spectrum of of sclerohumin R (**3**) (600 MHz, CD_3OD).

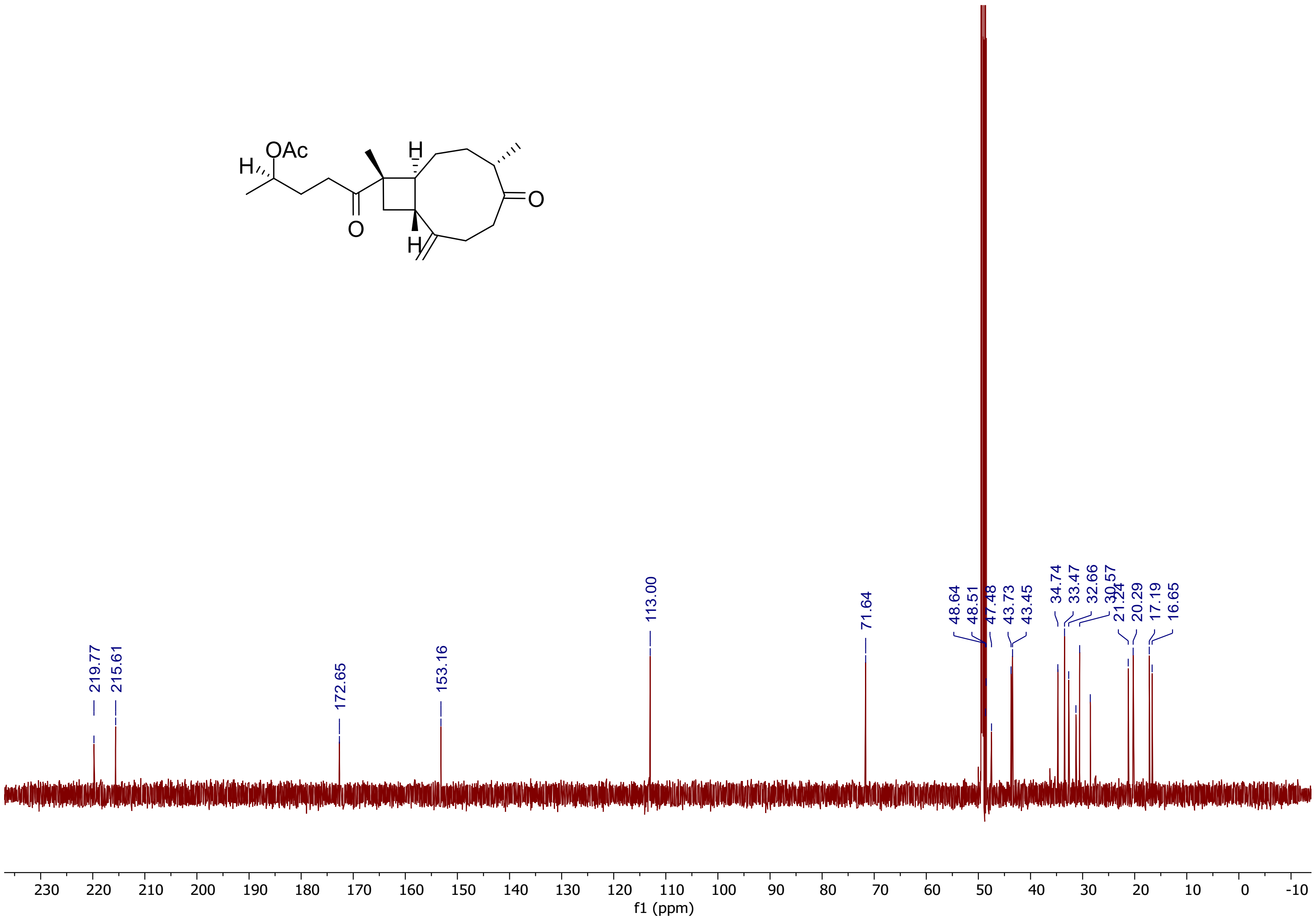

Figure S36. ^{13}C NMR spectrum of sclerohumin R (**3**) with chemical shift values displayed above the signals (150 MHz, CD_3OD).

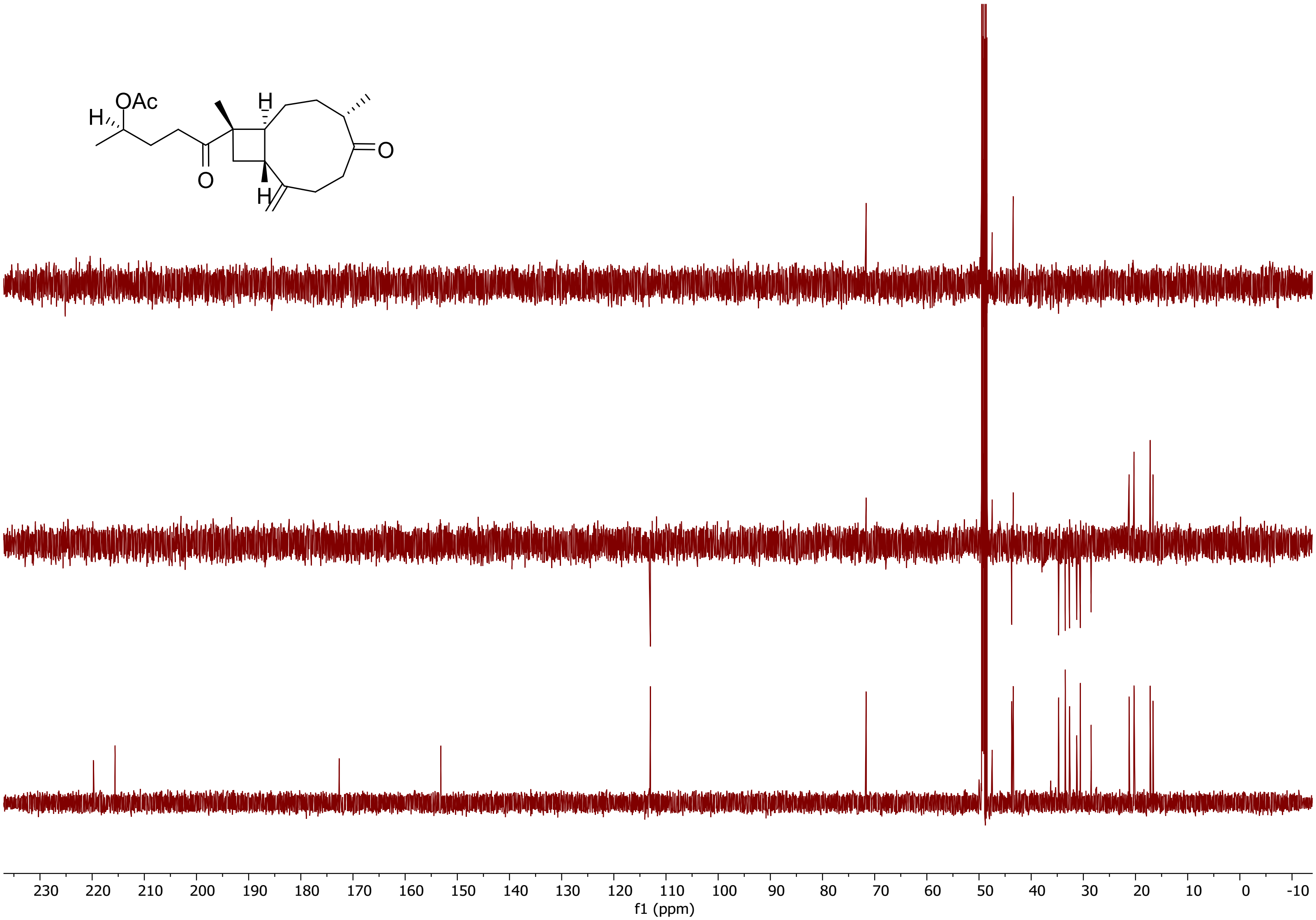

Figure S37. DEPT spectra of of sclerohumin R (**3**) (600 MHz, CD_3OD).

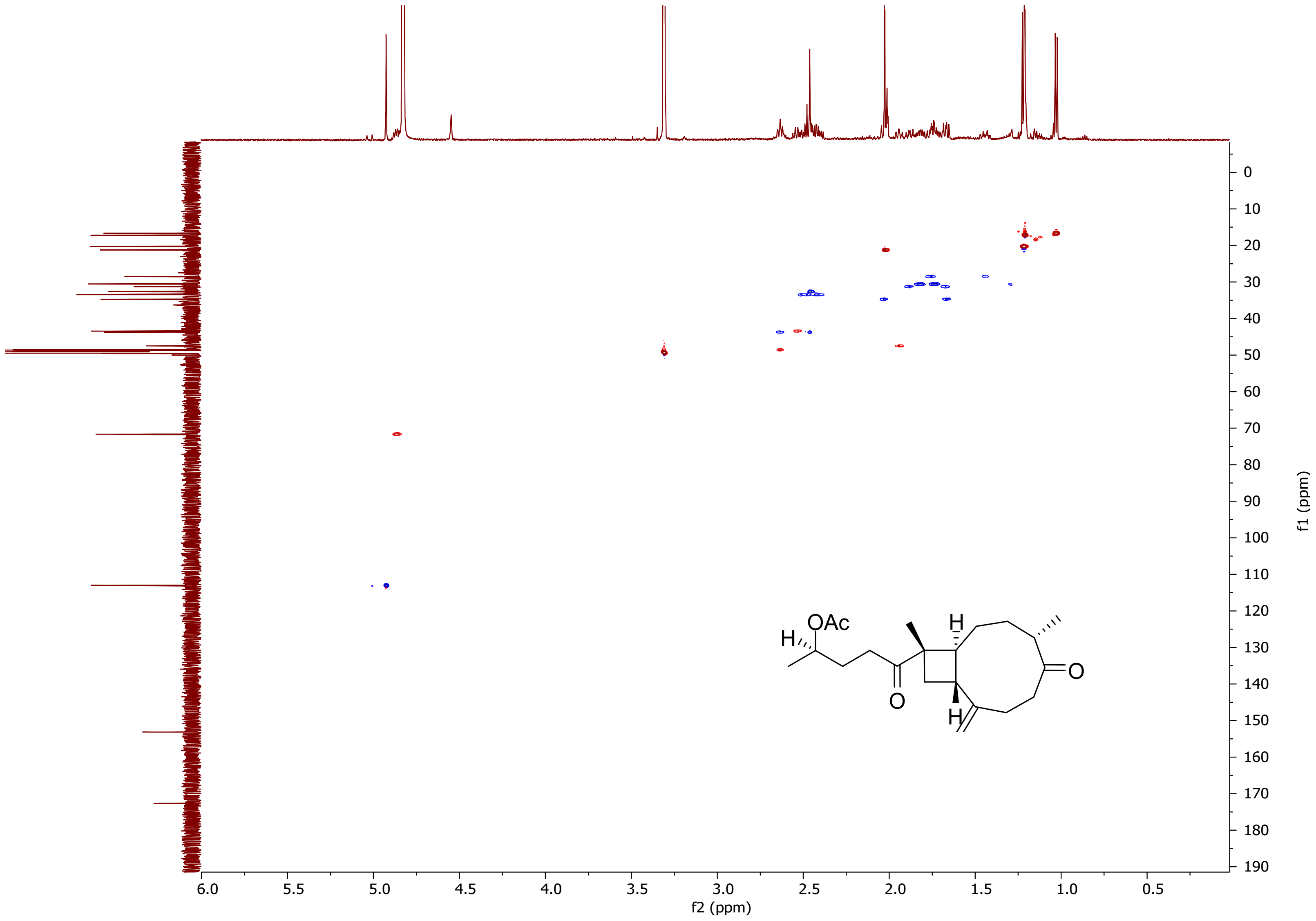

Figure S38. HSQC spectrum of sclerohumin R (**3**) (600 and 150 MHz, CD_3OD).

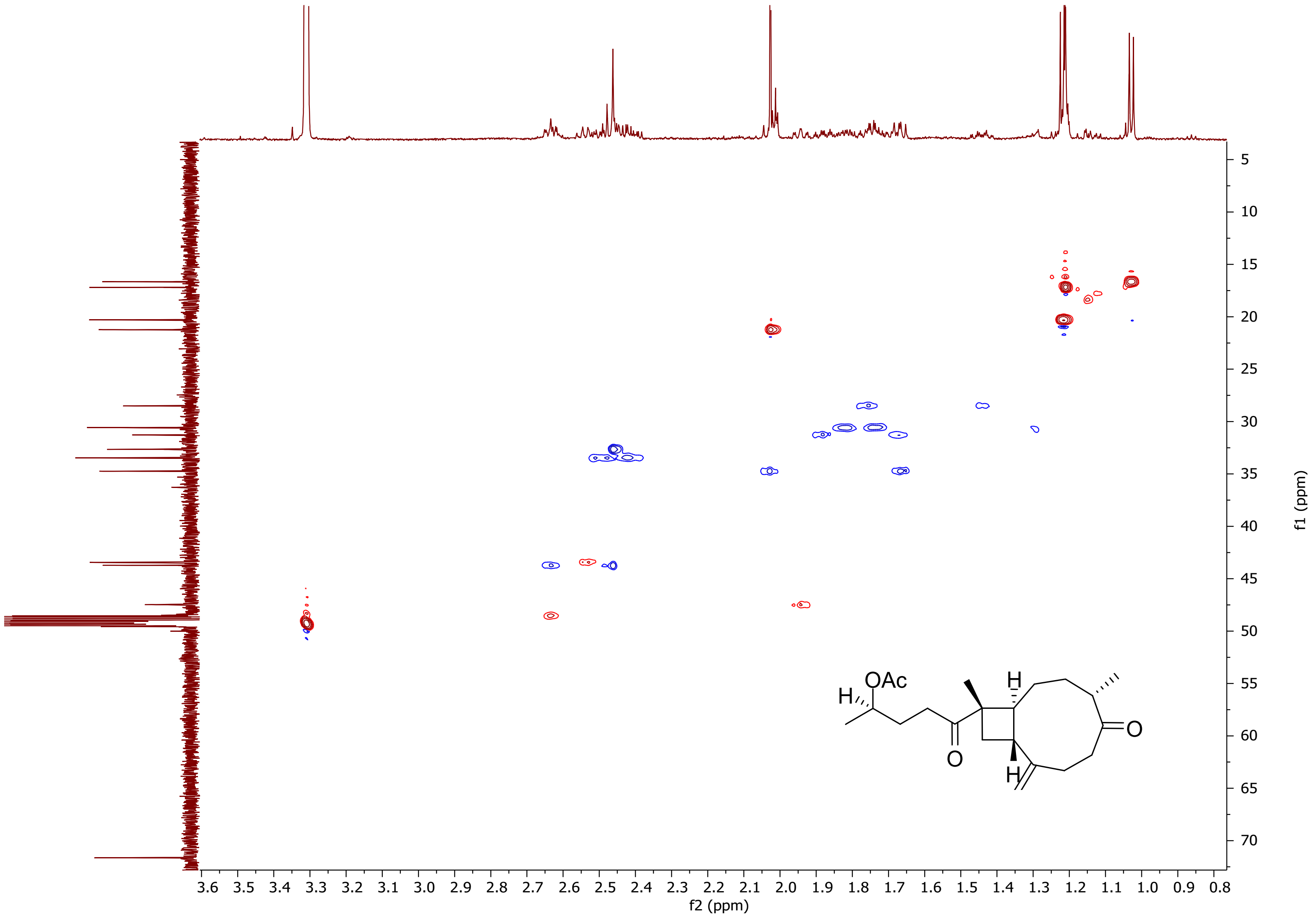

Figure S39. Expanded HSQC spectrum of sclerohumin R (**3**) (600 and 150 MHz, CD_3OD).

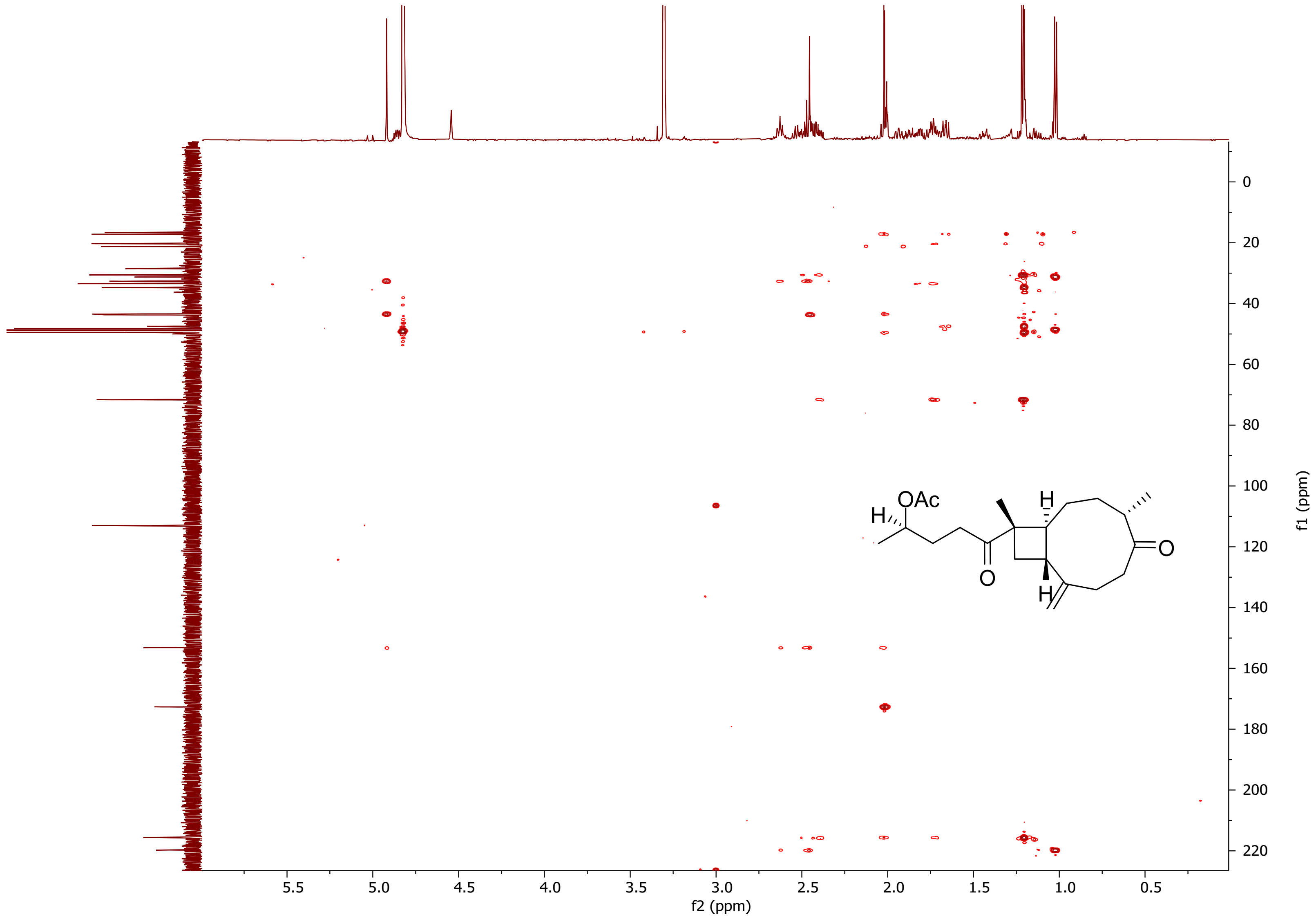

Figure S40. HMBC spectrum of sclerohumin R (**3**) (600 and 150 MHz, CD_3OD).

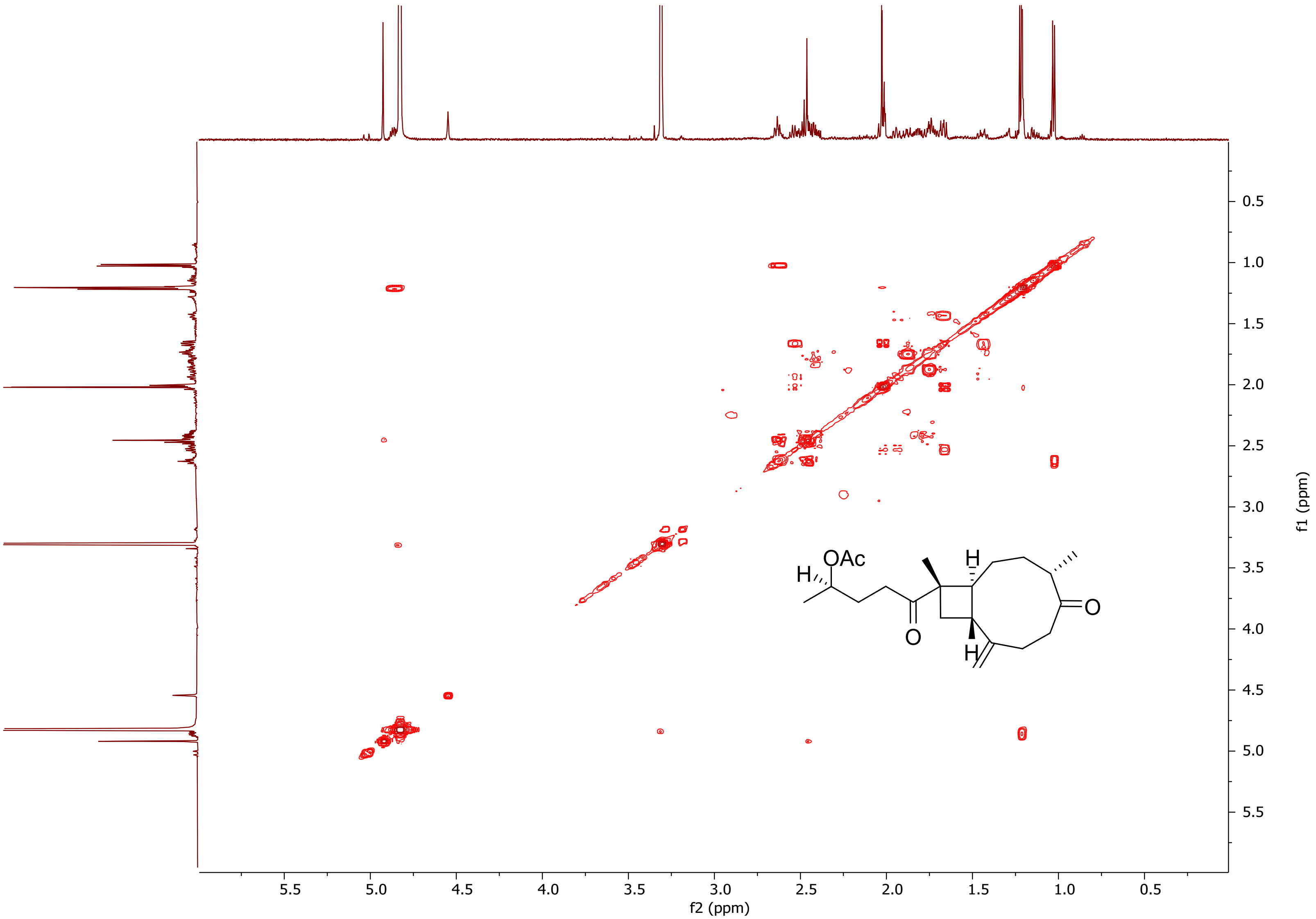

Figure S41. COSY spectrum of sclerohumin R (**3**) (600 MHz, CD_3OD).

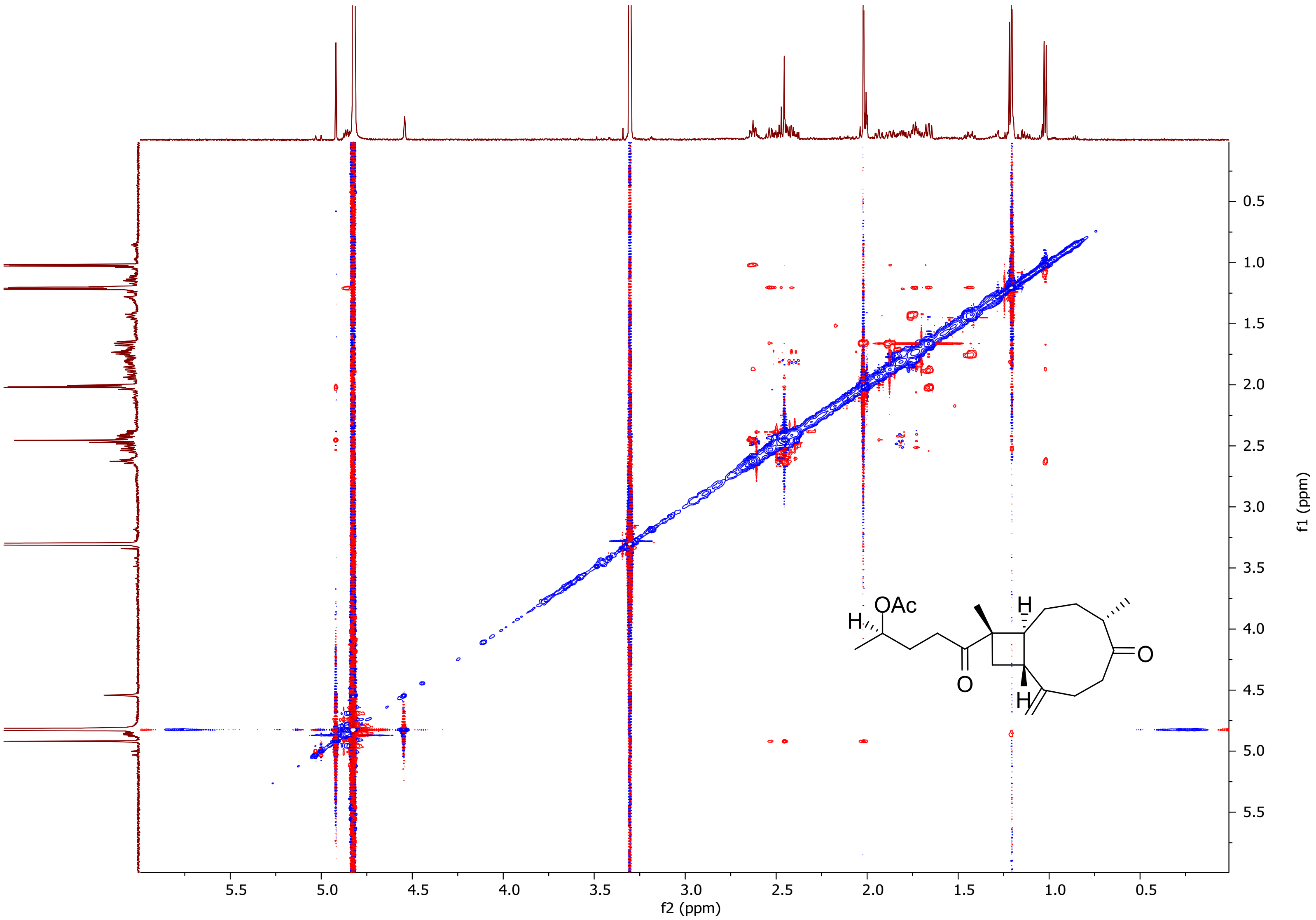

Figure S42. NOESY spectrum of sclerohumin R (**3**) (600 MHz, CD_3OD).

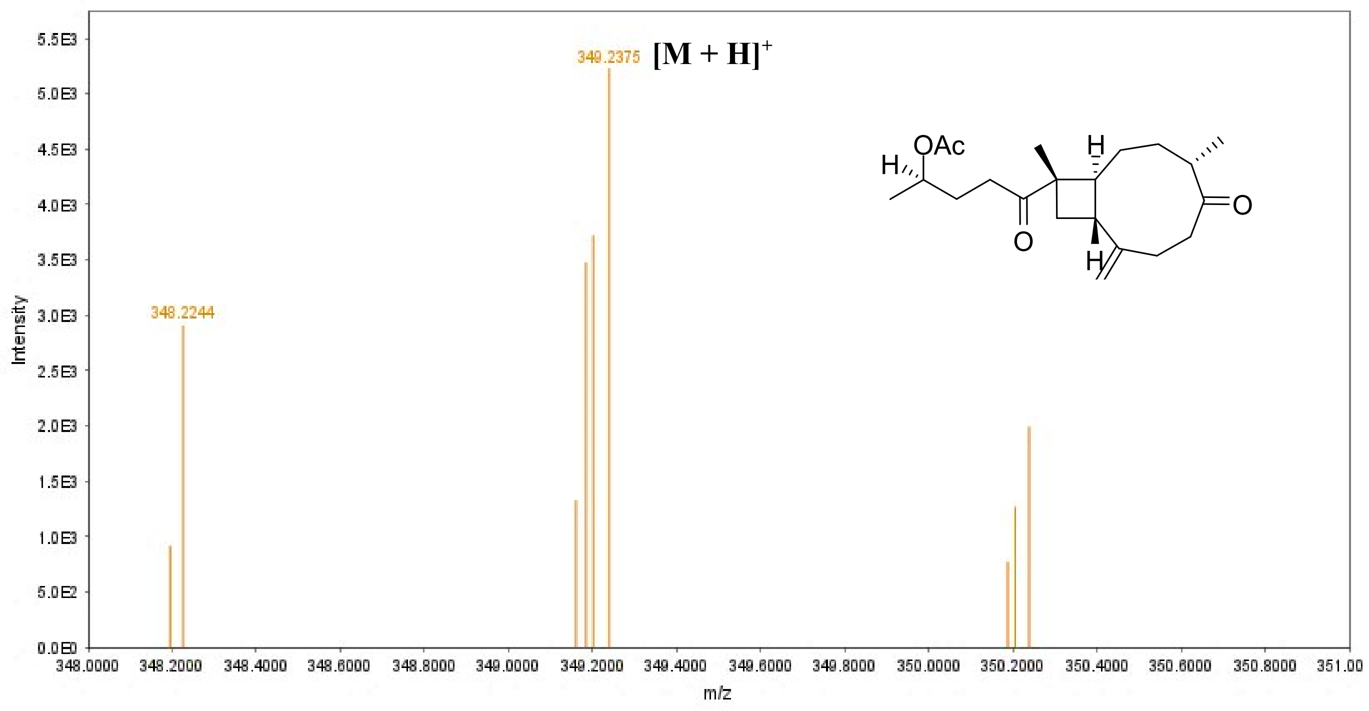

Hit	Formula	Calculated Mass	Target Mass	Error (mmu)	Error (ppm)
1	$C_{21}H_{33}O_4$	349.2379	349.2375	-0.4	-1.15

Figure S43. HRESIMS spectrum of sclerohumin R (**3**).

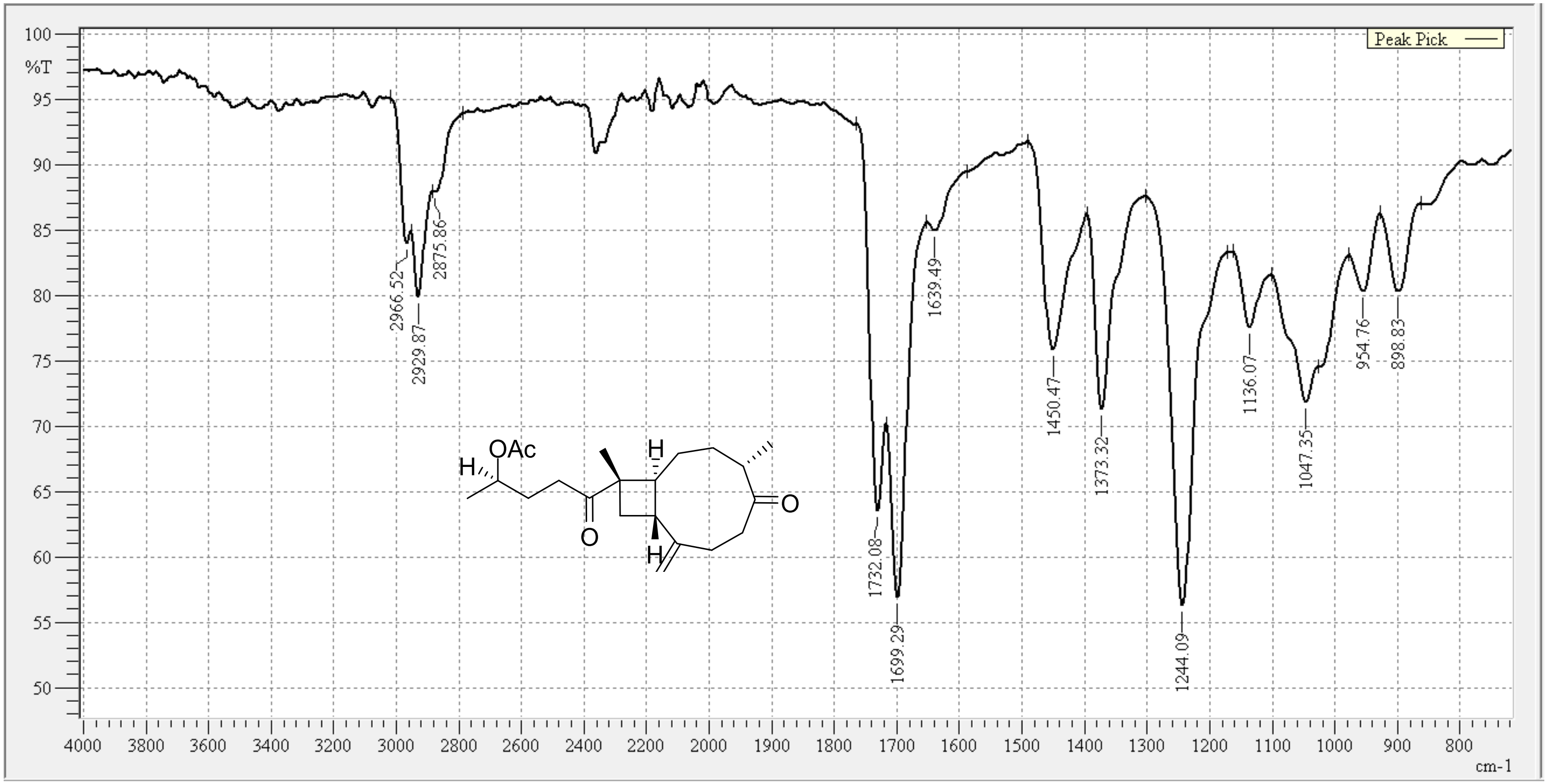

Figure S44. Infrared (IR) spectrum sclerohumin R (**3**).

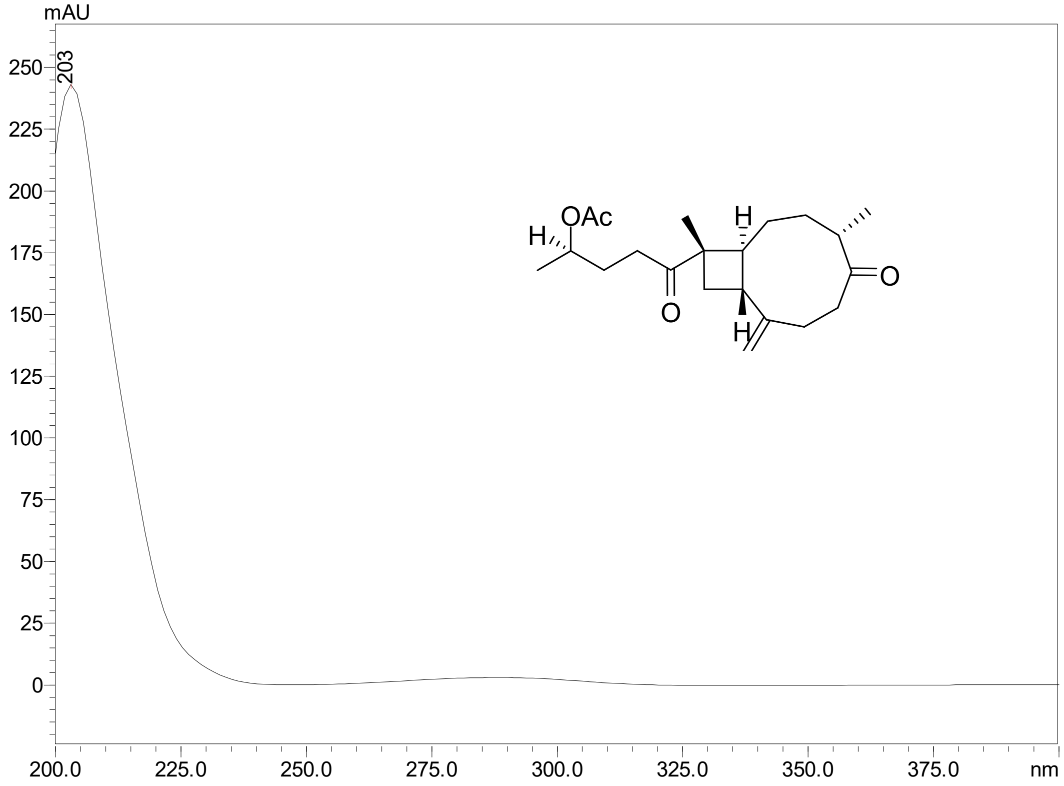

Figure S45. Ultraviolet (UV) spectrum sclerohumin R (**3**).

1.4. NMR, HRESIMS, IR, and UV spectra of sclerophyllene A (4)

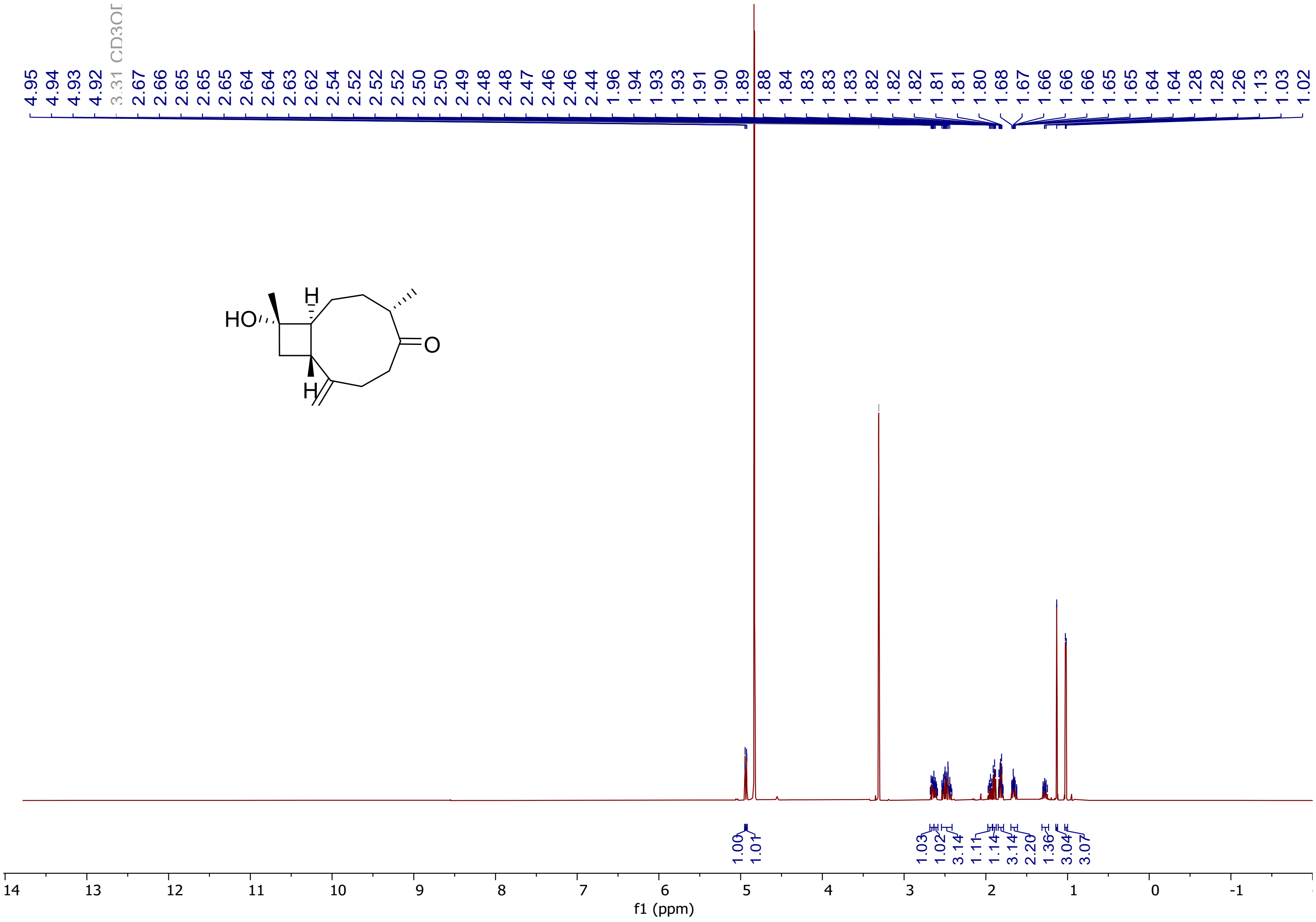

Figure S46. ^{1}H NMR spectrum of sclerophyllene A (**4**) (600 MHz, CD_3OD).

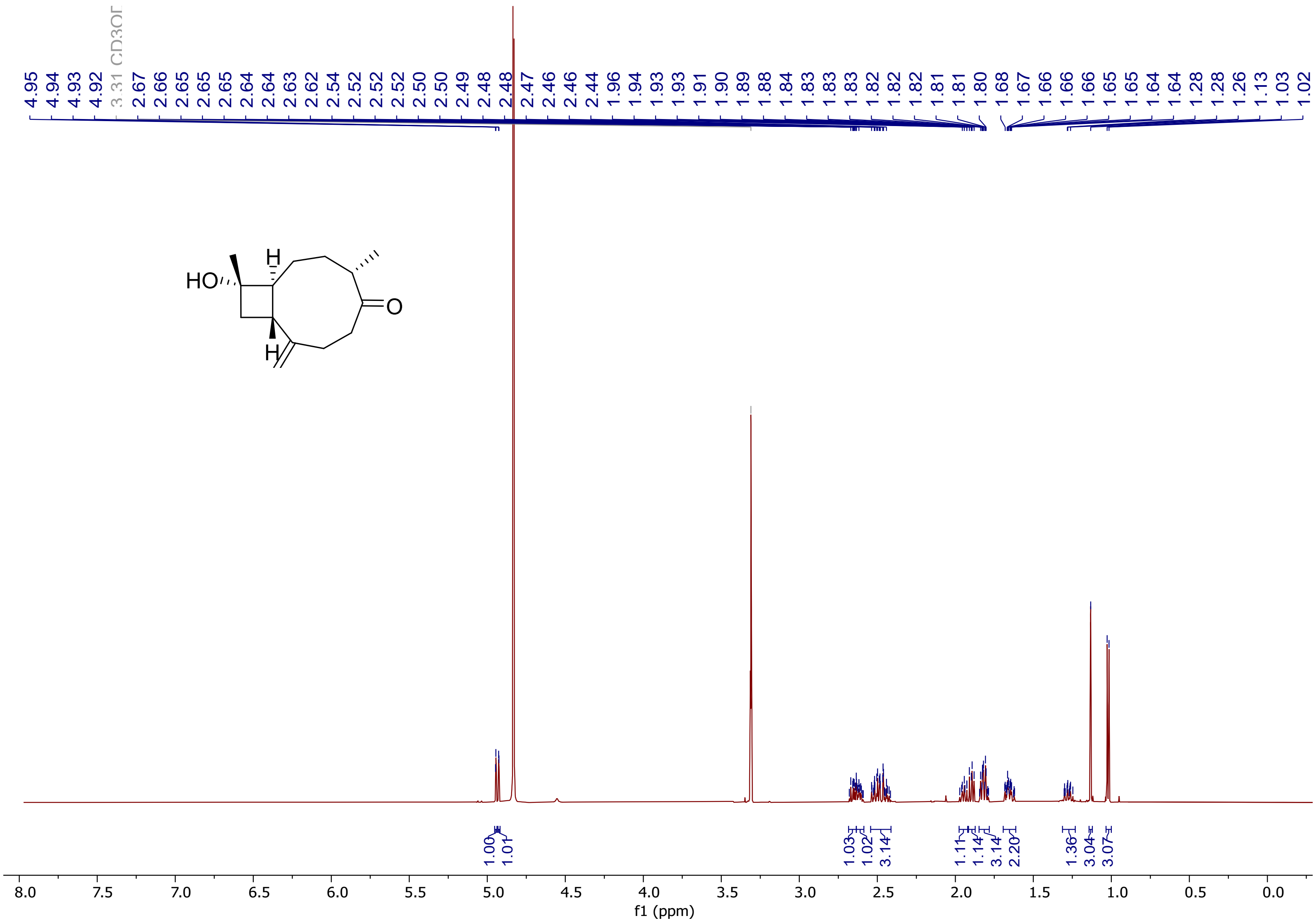

Figure S47. Expanded ^{1}H NMR spectrum of sclerophyllene A (**4**) (600 MHz, CD_3OD).

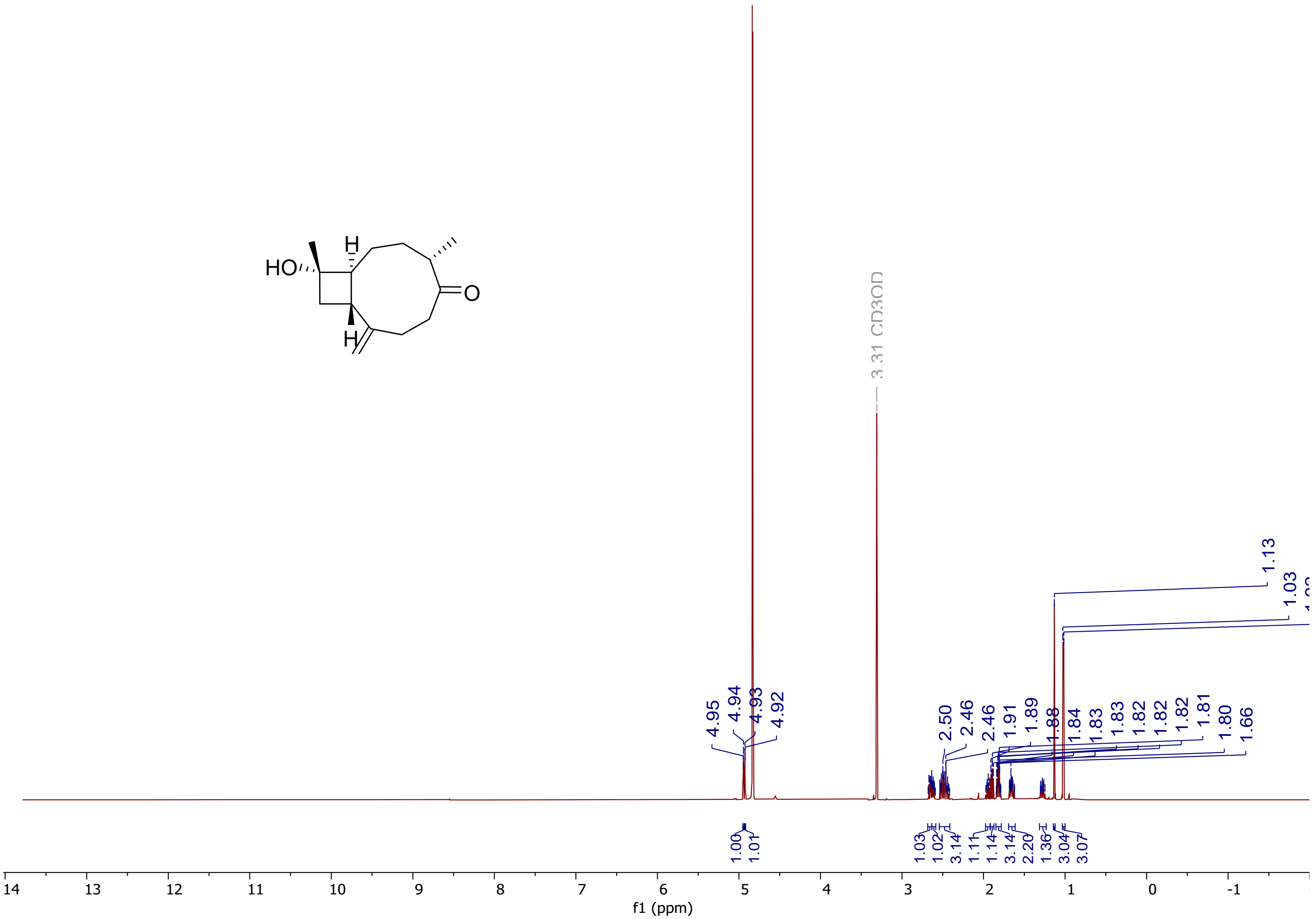

Figure S48. ^{1}H NMR spectrum of sclerophyllene A (**4**) with chemical shift values displayed above the signals (600 MHz, CD_3OD).

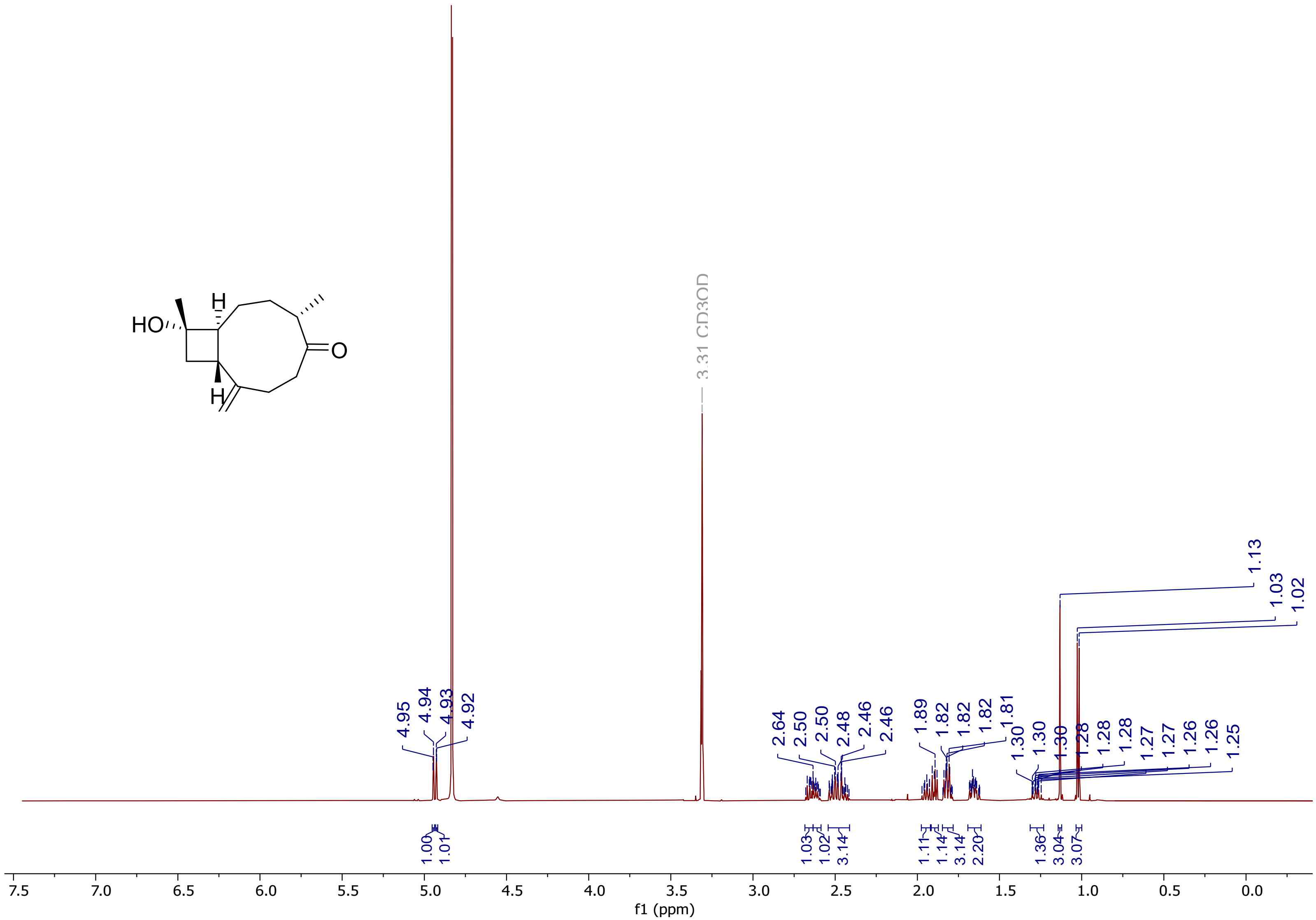

Figure S49. Expanded ^{1}H NMR spectrum of sclerophyllene A (**4**) with chemical shift values displayed above the signals (600 MHz, CD_3OD).

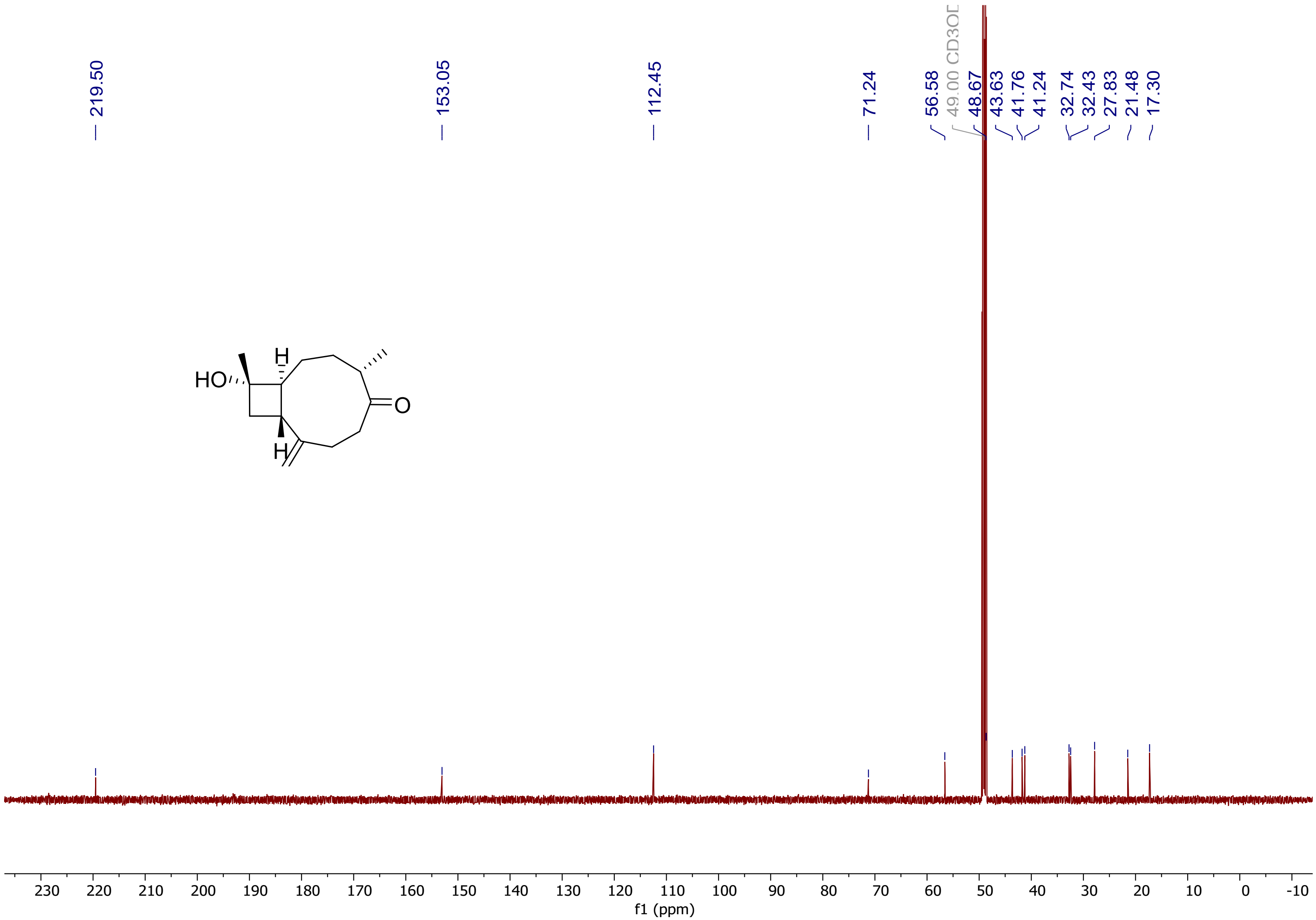

Figure S50. ^{13}C NMR spectrum of sclerophyllene A (**4**) (150 MHz, CD_3OD).

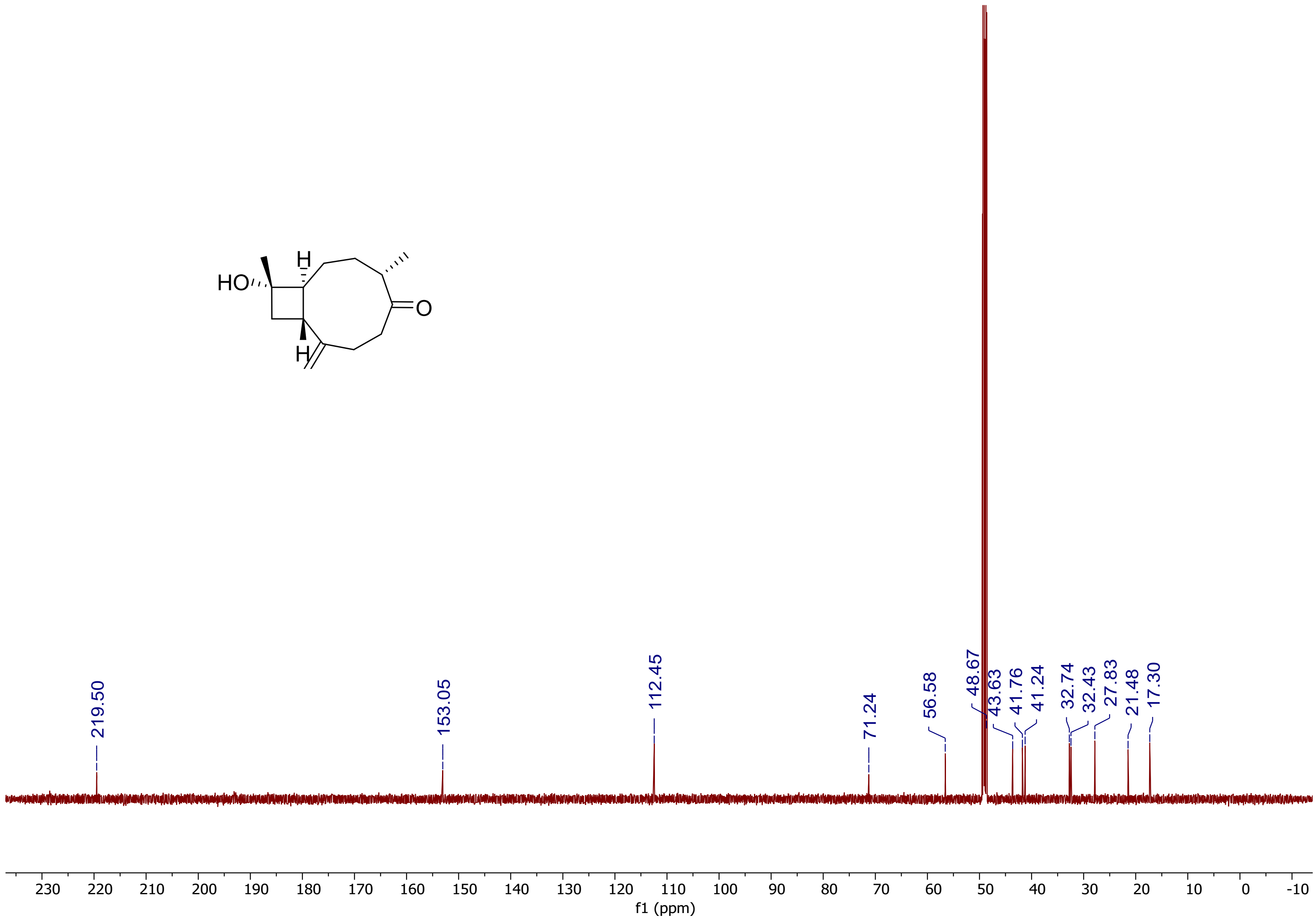

Figure S51. ^{13}C NMR spectrum of sclerophyllene A (**4**) with chemical shift values displayed above the signals (150 MHz, CD_3OD).

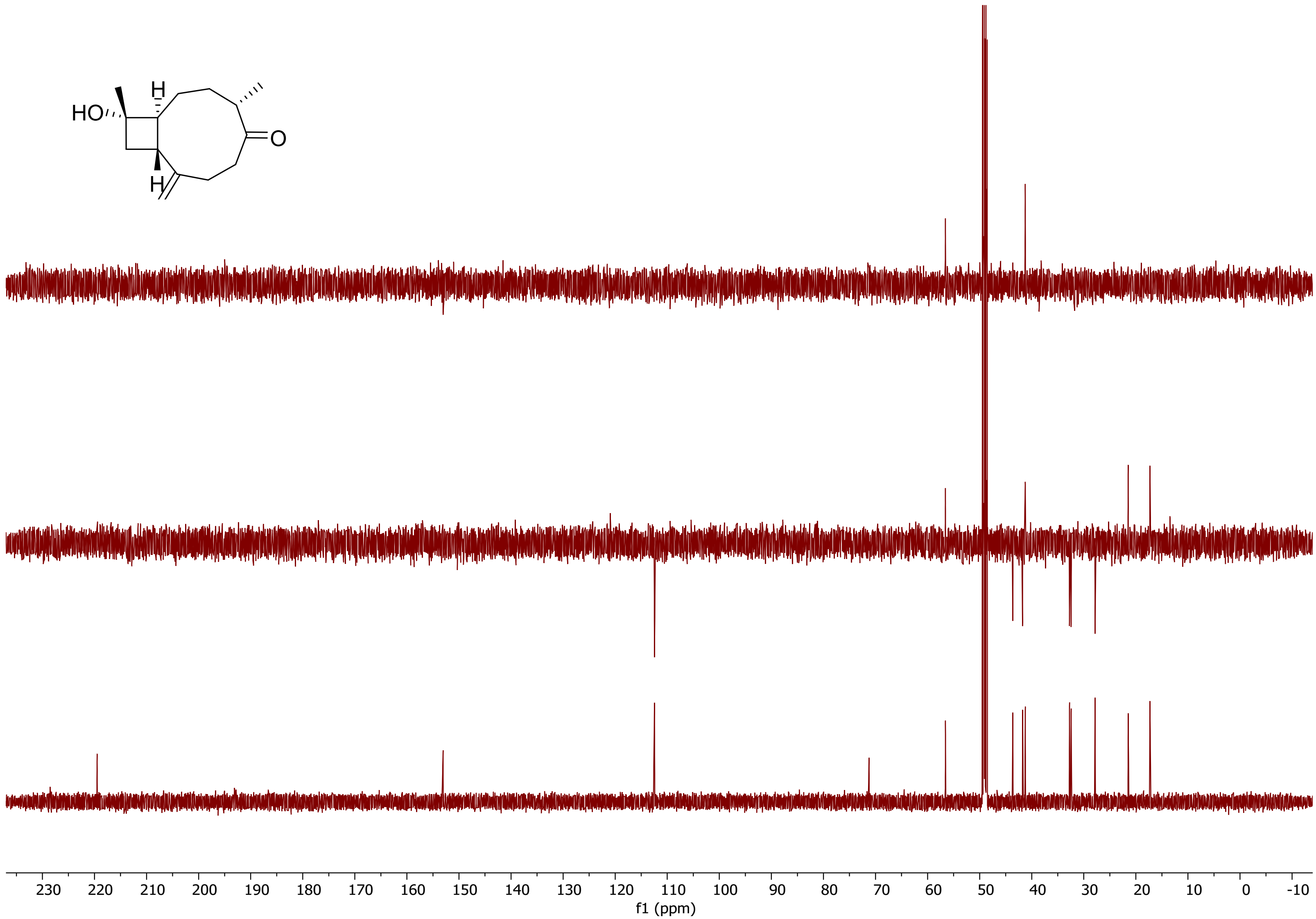

Figure S52. DEPT spectra of sclerophyllene A (**4**) (150 MHz, CD_3OD).

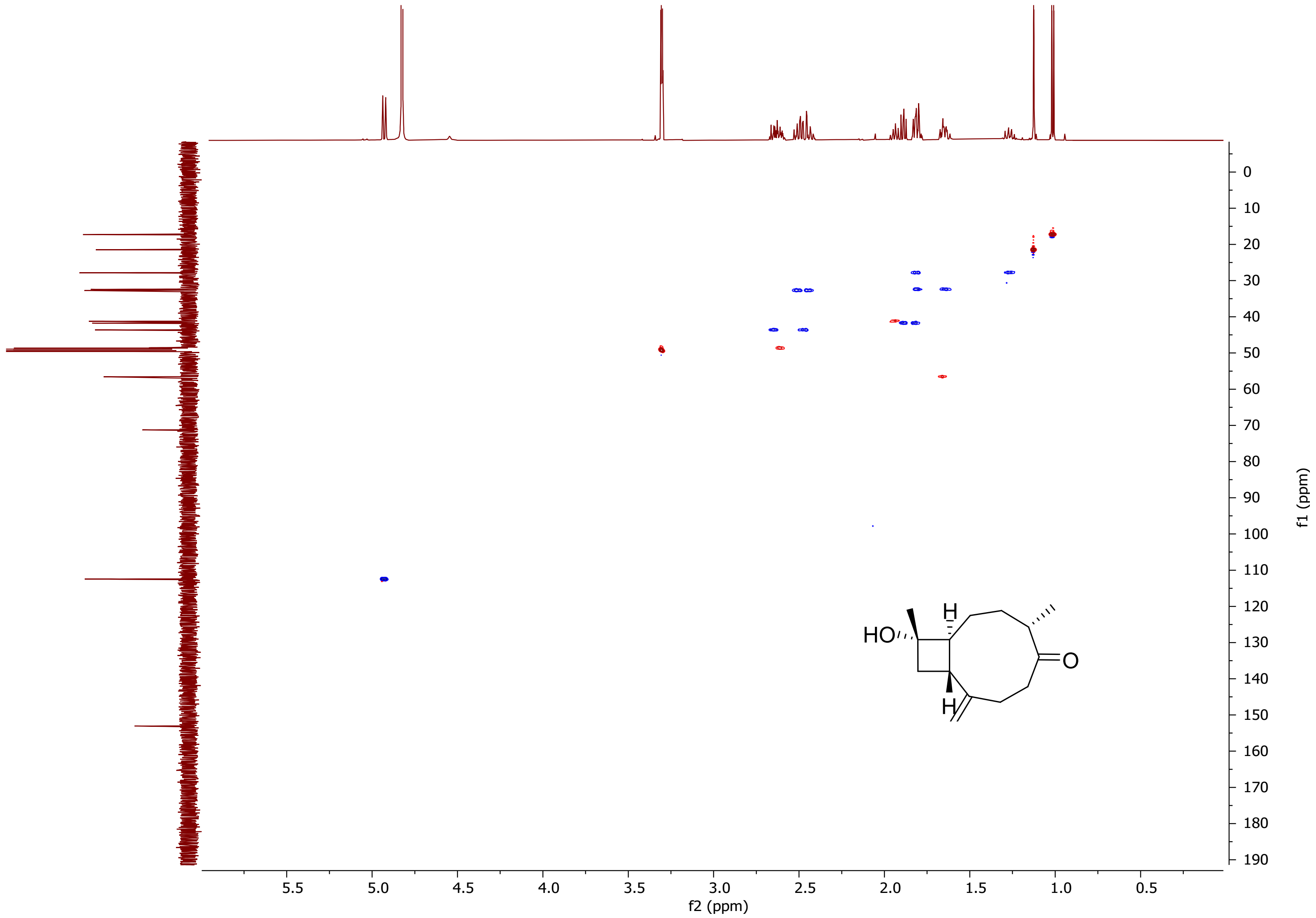

Figure S53. HSQC spectrum of sclerophyllene A (**4**) (600 and 150 MHz, CD_3OD).

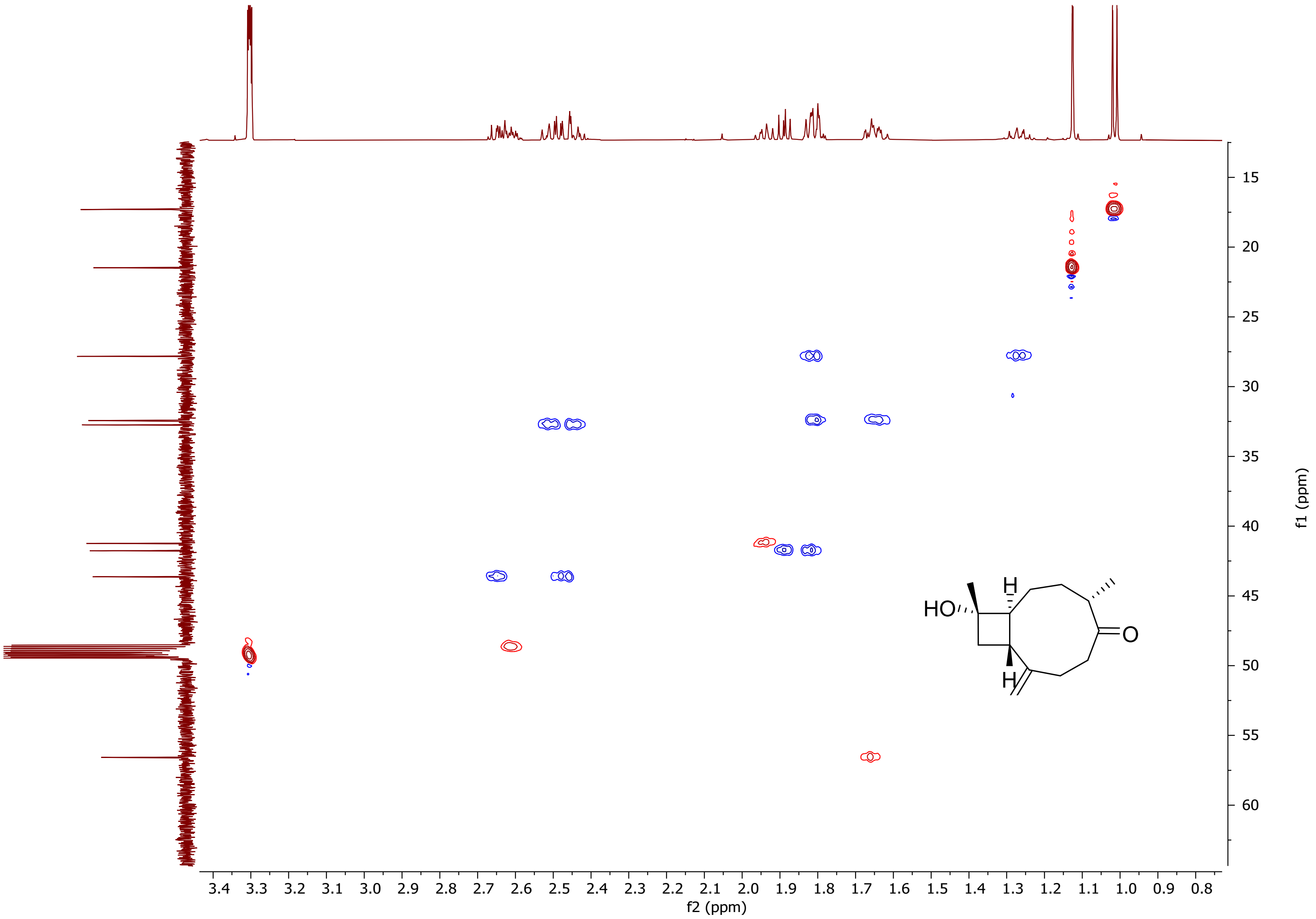

Figure S54. Expanded HSQC spectrum of sclerophyllene A (**4**) (600 and 150 MHz, CD_3OD).

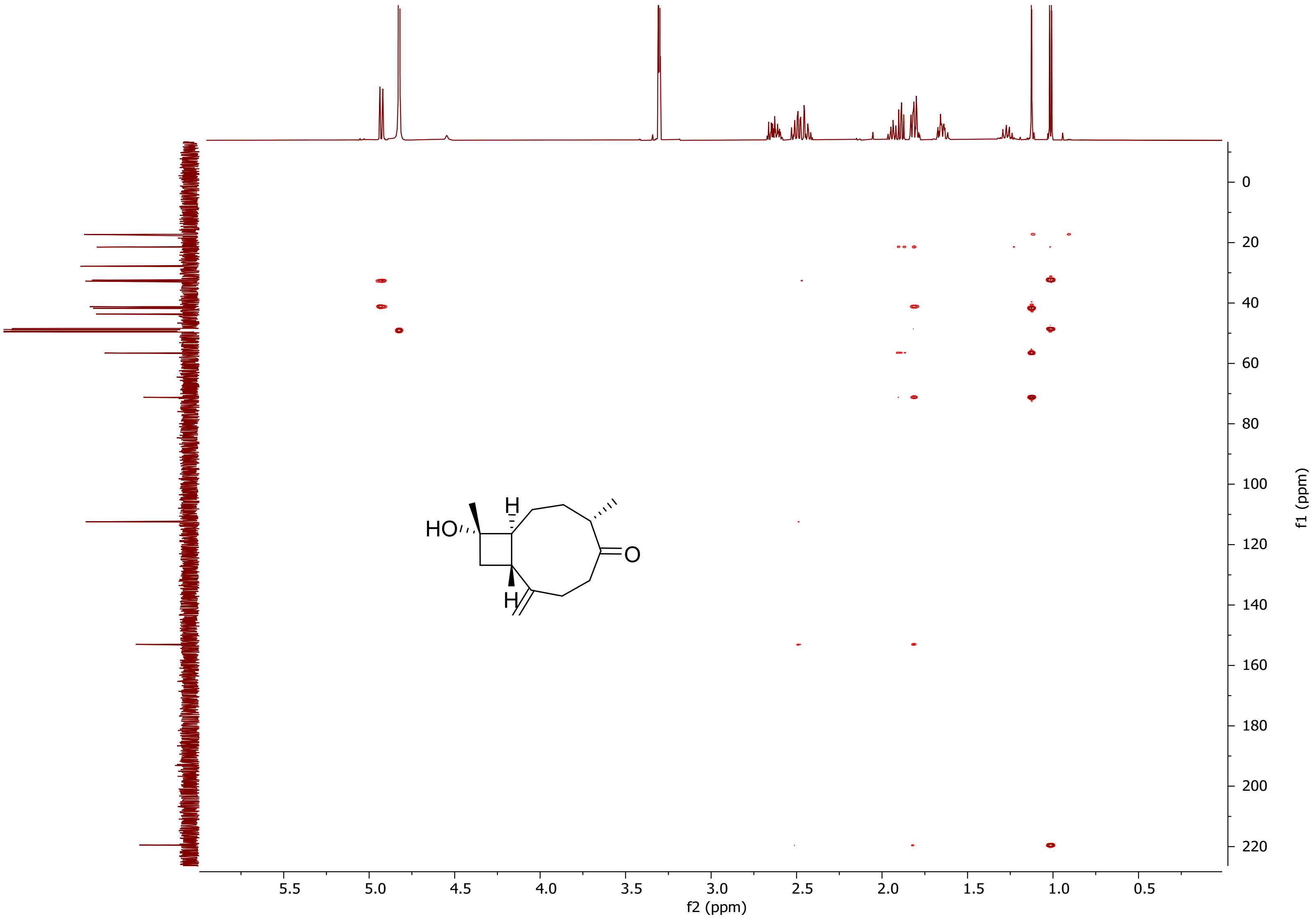

Figure S55. HMBC spectrum of sclerophyllene A (**4**) (600 and 150 MHz, CD_3OD).

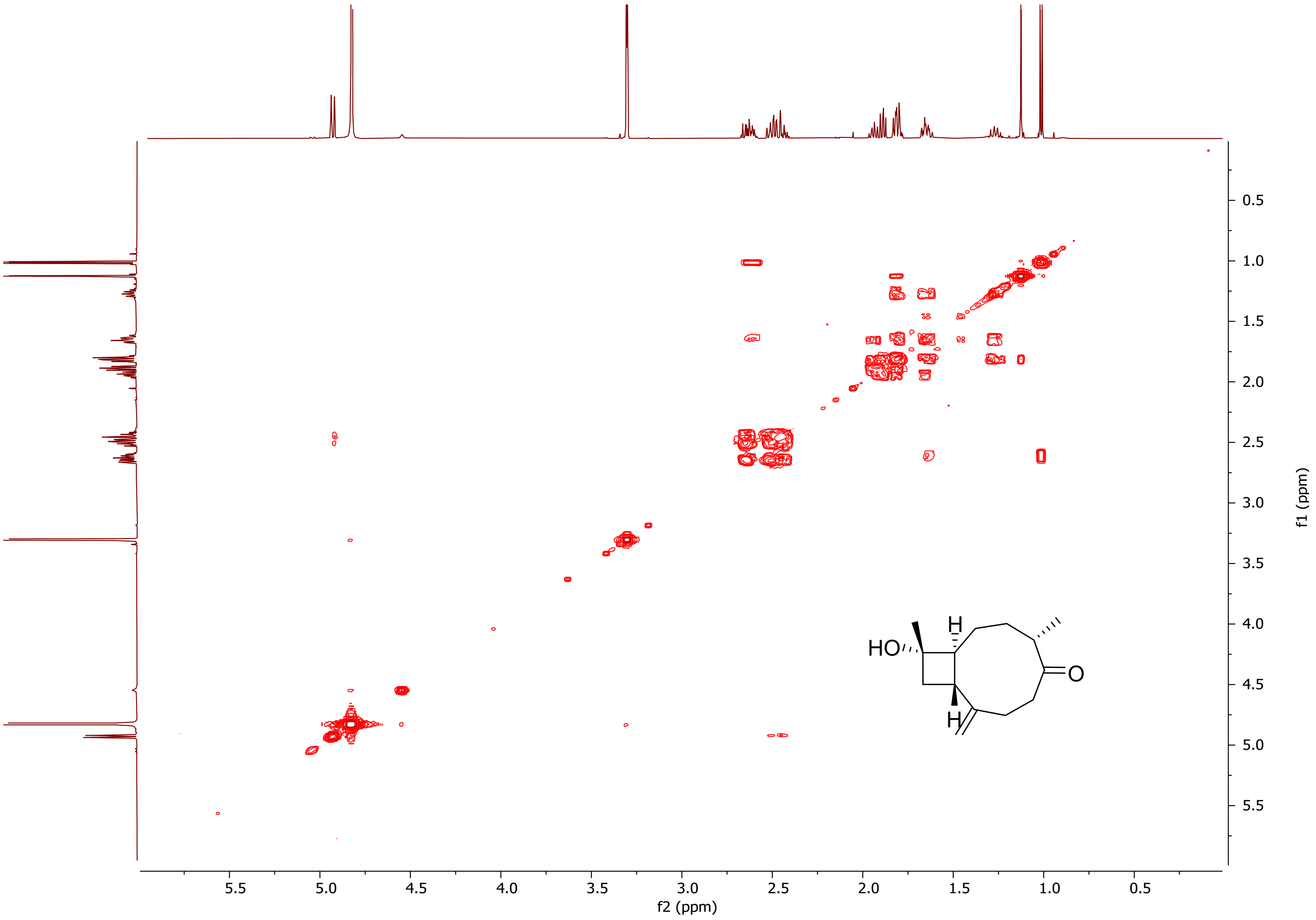

Figure S56. COSY spectrum of sclerophyllene A (**4**) (600 MHz, CD_3OD).

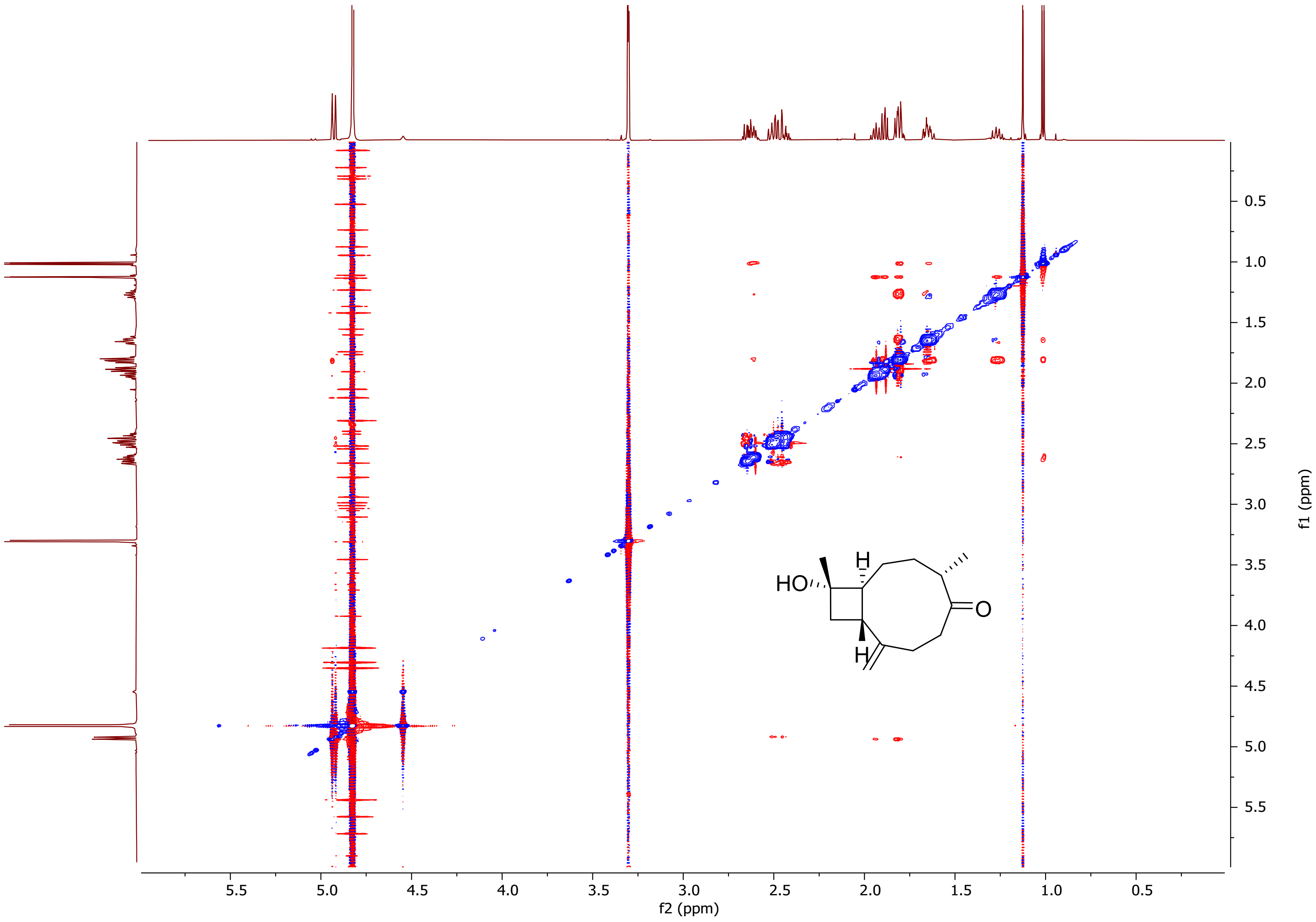

Figure S57. NOESY spectrum of sclerophyllene A (**4**) (600 MHz, CD_3OD).

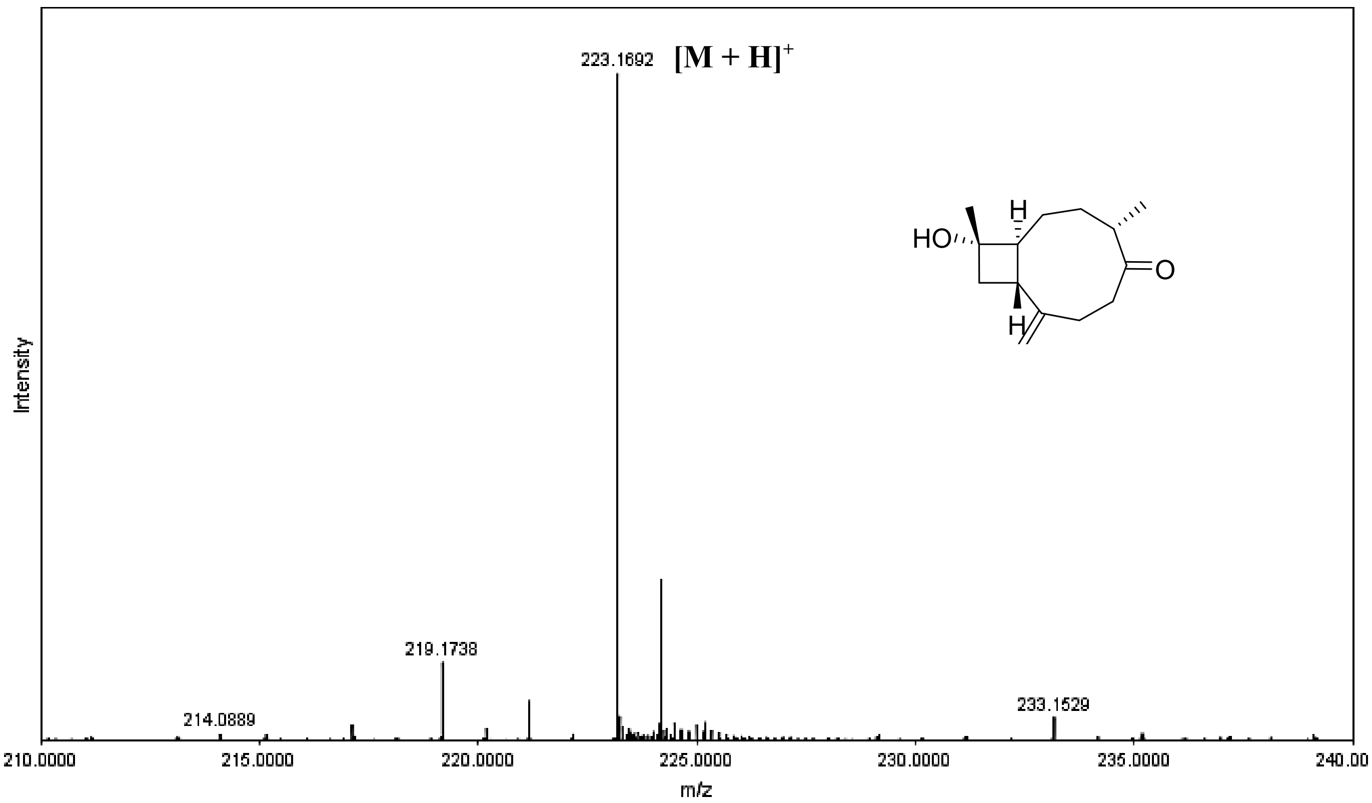

Hit	Formula	Calculated Mass	Target Mass	Error (mmu)	Error (ppm)
1	$C_{14}H_{23}O_2$	223.1698	223.1692	-0.6	-2.69

Figure S58. HRESIMS spectrum of sclerophyllene A (**4**).

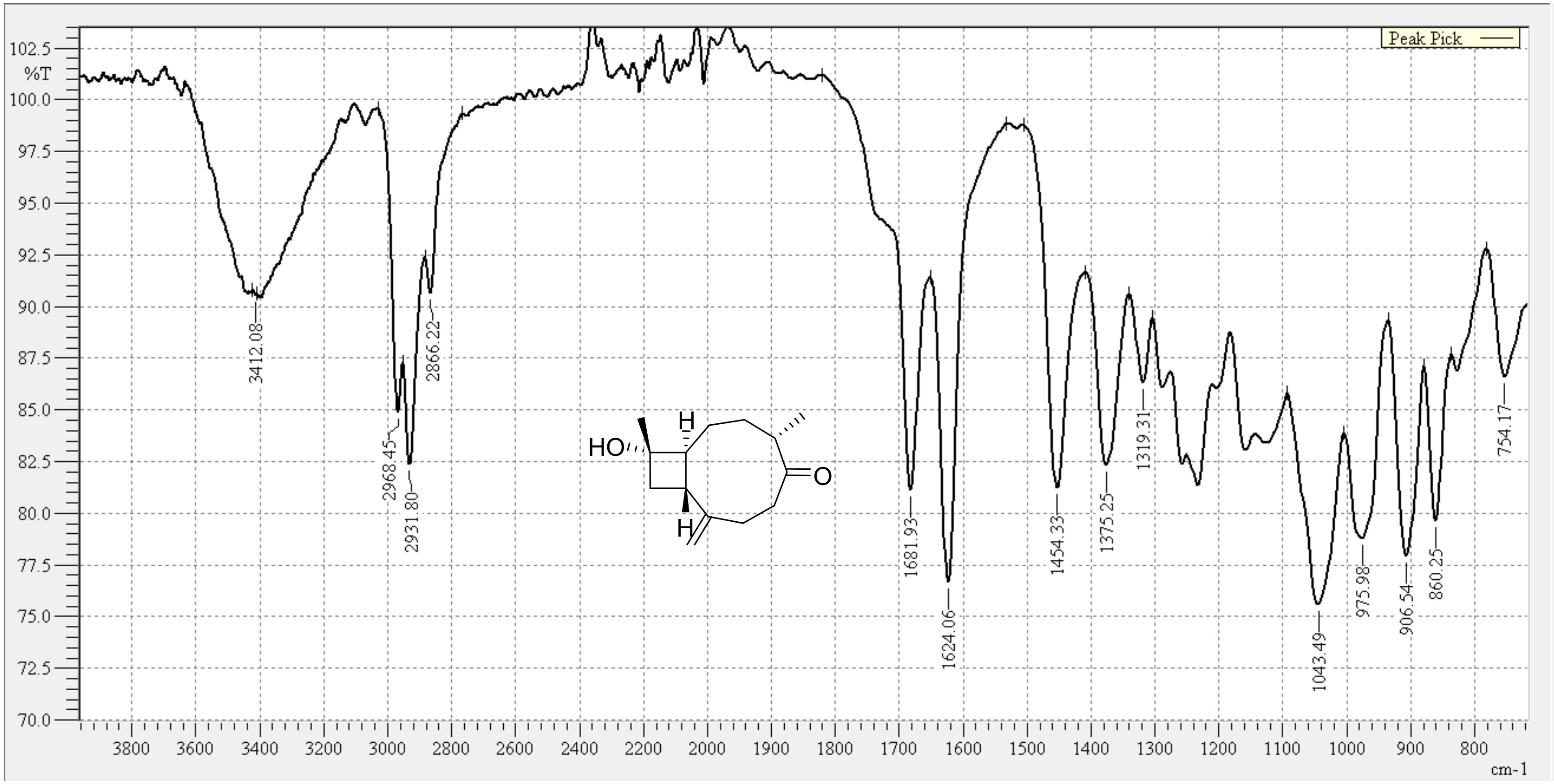

Figure S59. Infrared (IR) spectrum sclerophyllene A (**4**).

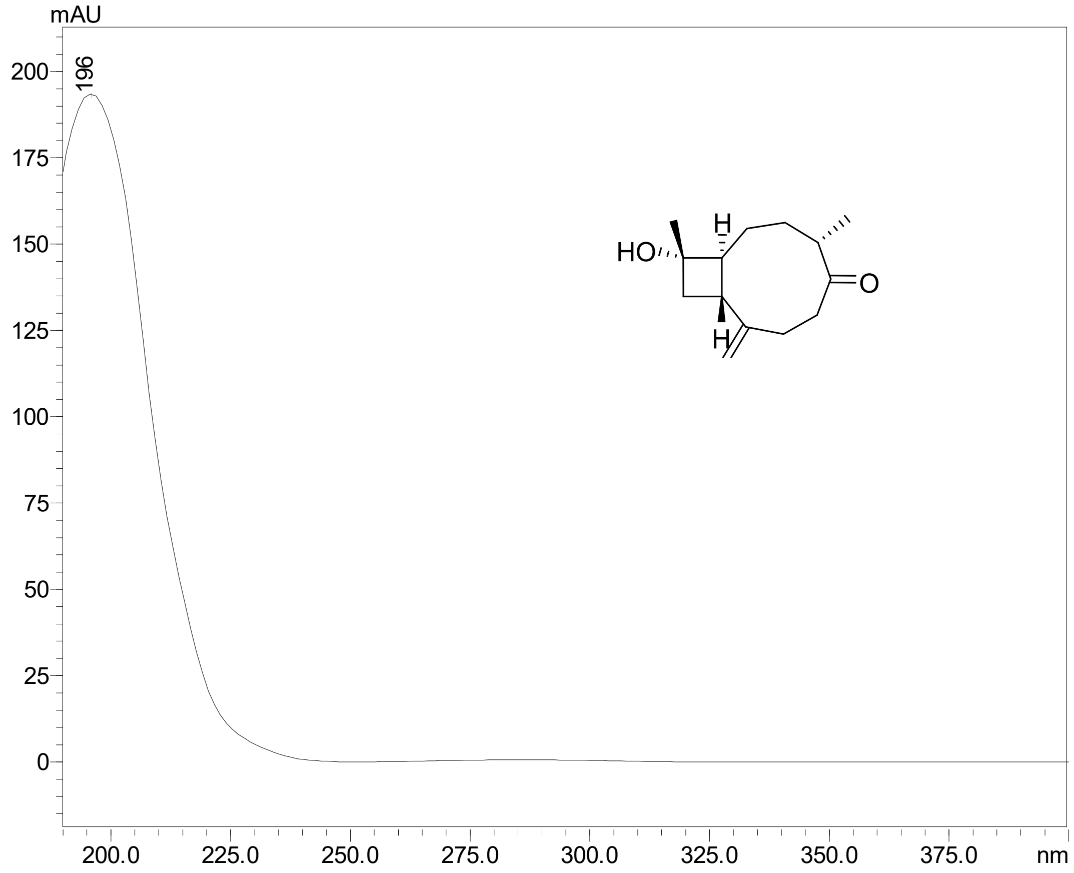

Figure S60. Ultraviolet (UV) spectrum sclerophyllene A (**4**).

Table S1. 2D NMR data for compound **1**

No.	**1**[a]		
	^{1}H-^{1}H COSY	^{1}H-^{13}C HMBC	NOESY
1	H-9, H_2-2	-	H-3α, H-5
2α	H-1, H_2-3	-	-
2β			H-9
3α	H_2-2	-	H-1
3β			-
4	-	-	-
5	H_2-6	-	H-1
6	H-5, H_2-7	-	-
7	H_2-6	-	-
8	-	-	-
9	H-1, H_2-10	-	H_3-18, H-2β, H_3-20
10	H-9	C-8, C-11, C-18	-
11	-	-	-
12	-	-	-
13	H-14	C-12, C-14, C-15	-
14	H-13	C-12, C-13, C-15, C-16, C-17	-
15	-	-	-
16	-	C-14, C-15, C-17	-
17	-	C-14, C-15, C-16	-
18	-	C-1, C-10, C-11, C-12	H-9
19	-	C-1, C-7, C-8	-<br>-
20	-	C-3, C-4, C-5	H-9

[a]Spectra recorded in CD_3OD at 600 MHz (^{1}H NMR) and 150 MHz (^{13}C NMR).

Table S2. 2D NMR data for compound **2**

No.	**2**[a]		
	^{1}H-^{1}H COSY	^{1}H-^{13}C HMBC	NOESY
1	H-9, H_2-2	-	H-3α, H-5, H-10α
2α	H-1, H_2-3	-	-
2β			H-9
3α	H_2-2	-	H-1
3β			-
4	-	-	-
5	H_2-6	-	H-1
6	H-5, H_2-7	-	-
7α	H_2-6	-	-
7β			H-9
8	-	-	-
9	H-1, H_2-10	-	H_3-18, H-2β, H-7β, H_3-20
10α	H-9	C-8, C-11, C-18	H-1
10β			-
11	-	-	-
12	-	-	-
13	H-14	C-12, C-14, C-15	-
14	H-13	C-12, C-13, C-15, C-16, C-17	-
15	-	-	-
16	-	C-14, C-15, C-17	-
17	-	C-14, C-15, C-16	-
18	-	C-1, C-10, C-11, C-12	H-9
19	-	C-1, C-7, C-8	-<br>-
20	-	C-3, C-4, C-5	H-9

[a]Spectra recorded in CD_3OD at 600 MHz (^{1}H NMR) and 150 MHz (^{13}C NMR).

Table S3. 2D NMR data for compound **3**

No.	**3**[a]		
	^{1}H-^{1}H COSY	^{1}H-^{13}C HMBC	NOESY
1	H-9, H_2-2	-	H-3α
2α<br>2β	H-1, H_2-3	-	-
3α<br>3β	H_2-2, H-4	-	H-1, H_3-20<br>-
4	H_2-3, H_3-20	-	-
5	-	-	H-1
6	H_2-7	-	-
7α<br>7β	H_2-6	-	-<br>-
8	-	-	-
9	H-1, H_2-10	-	H_3-18
10	H-9	C-8, C-11, C-18	-<br>-
11	-	-	-
12	-	-	-
13	H-14	-	-
14	H-13	-	-
15	-	-	-
16	-	-	-
17	H-15	C-14, C-15	-
18	-	C-1, C-10, C-11, C-12	H-9
19	-	C-1, C-7, C-8	-<br>-
20	-	C-3, C-4, C-5	H-3α

[a]Spectra recorded in CD_3OD at 600 MHz (^{1}H NMR) and 150 MHz (^{13}C NMR).

Table S4. 2D NMR data for compound **4**

No.	4[a]		
	^{1}H-^{1}H COSY	^{1}H-^{13}C HMBC	NOESY
1	H-9, H_2-2	-	H_3-15
2α	H-1, H_2-3	-	-
2β			H-4, H-9
3α	H_2-2, H-4	-	-
3β			-
4	H_2-3, H_3-15	C-5	H-2β
5	-	-	-
6	H_2-7	-	-
7α	H_2-6	-	-
7β			H-9
8	-	-	-
9	H-1, H_2-10	C-8	H_3-12, H-2β, H-7β
10	H-9	C-8, C-11, C-18	- -
11	-	-	-
12	-	C-1, C-10, C-11	H-9
13	-	-	-
14	-	C-7, C-8, C-9	-
15	-	C-3, C-4, C-5	H-1

[a]Spectra recorded in CD_3OD at 600 MHz (^{1}H NMR) and 150 MHz (^{13}C NMR).

2. Optical Rotation

Table S5. Optical Rotation of compound **1**

[Data Information]
Creation Date 2026/2/25 下午 01:37

[Measurement Information]
Instrument Name P-2000
Model Name P-2000
Serial No. A062461232
Polarizer Dichrom
Faraday Cell Flint Glass

Accessory RSC-200
Accessory S/N B045761260

Light Source Na
Monitor wavelength 589 nm
D.I.T. 5 sec
No. of cycle 5
Cycle interval 2 sec
Temp. Monitor Cell
Temp. Corr. Factor None
Aperture (S) 3.0 nm
Aperture (L) Auto
Mode Specific O.R.
Path Length 50 nm
Cencentration 0.3 w/v%
Water content of sample 0%
Factor 1

No.	Sample Name	Measurement Date	PMT Voltage[V]	Temperature[C]	Optical Rotation Monito	Specific O.R.
1	1			25.00	-0.0165	-16.0208
2	1	2026/2/25 下午 01:37	350	25.00	-0.0172	-14.3001
3	1	2026/2/25 下午 01:37	425	25.00	-0.0009	-14.3203
4	1	2026/2/25 下午 01:37	431	25.00	-0.0136	-14.7206
5	1	2026/2/25 下午 01:37	247	25.00	-0.0104	-15.0014
6	1	2026/2/25 下午 01:37	437	25.00	-0.0135	-17.0902

Table S6. Optical Rotation of compound **2**

[Data Information]
Creation Date 2026/2/25 下午 01:52

[Measurement Information]
Instrument Name P-2000
Model Name P-2000
Serial No. A062461232
Polarizer Dichrom
Faraday Cell Flint Glass

Accessory RSC-200
Accessory S/N B045761260

Light Source Na
Monitor wavelength 589 nm
D.I.T. 5 sec
No. of cycle 5
Cycle interval 2 sec
Temp. Monitor Cell
Temp. Corr. Factor None
Aperture (S) 3.0 nm
Aperture (L) Auto
Mode Specific O.R.
Path Length 50 nm
Cencentration 0.3 w/v%
Water content of sample 0%
Factor 1

No.	Sample Name	Measurement Date	PMT Voltage[V]	Temperature[C]	Optical Rotation Monito	Specific O.R.
1	2			25.00	-0.0089	-12.0016
2	2	2026/2/25 下午 01:52	430	25.00	-0.0090	-11.9052
3	2	2026/2/25 下午 01:52	430	25.00	-0.0103	-12.7203
4	2	2026/2/25 下午 01:52	425	25.00	-0.0125	-13.0084
5	2	2026/2/25 下午 01:52	432	25.00	-0.0009	-12.6931
6	2	2026/2/25 下午 01:52	375	25.00	-0.0120	-13.1825

Table S7. Optical Rotation of compound **3**

[Data Information]
Creation Date 2026/2/25 下午 01:59

[Measurement Information]
Instrument Name P-2000
Model Name P-2000
Serial No. A062461232
Polarizer Dichrom
Faraday Cell Flint Glass

Accessory RSC-200
Accessory S/N B045761260

Light Source Na
Monitor wavelength 589 nm
D.I.T. 5 sec
No. of cycle 5
Cycle interval 2 sec
Temp. Monitor Cell
Temp. Corr. Factor None
Aperture (S) 3.0 nm
Aperture (L) Auto
Mode Specific O.R.
Path Length 50 nm
Cencentration 0.3 w/v%
Water content of sample 0%
Factor 1

No.	Sample Name	Measurement Date	PMT Voltage[V]	Temperature[C]	Optical Rotation Monito	Specific O.R.
1	3			25.00	0.0106	16.5901
2	3	2026/2/25 下午 01:59	262	25.00	0.0133	15.5576
3	3	2026/2/25 下午 01:59	306	25.00	0.0093	18.6889
4	3	2026/2/25 下午 01:59	308	25.00	0.0096	18.8461
5	3	2026/2/25 下午 01:59	345	25.00	0.0031	15.0172
6	3	2026/2/25 下午 01:59	343	25.00	0.0033	16.9782

Table S8. Optical Rotation of compound **4**

[Data Information]
Creation Date 2026/2/25 下午 02:12

[Measurement Information]
Instrument Name P-2000
Model Name P-2000
Serial No. A062461232
Polarizer Dichrom
Faraday Cell Flint Glass

Accessory RSC-200
Accessory S/N B045761260

Light Source Na
Monitor wavelength 589 nm
D.I.T. 5 sec
No. of cycle 5
Cycle interval 2 sec
Temp. Monitor Cell
Temp. Corr. Factor None
Aperture (S) 3.0 nm
Aperture (L) Auto
Mode Specific O.R.
Path Length 50 nm
Cencentration 0.3 w/v%
Water content of sample 0%
Factor 1

No.	Sample Name	Measurement Date	PMT Voltage[V]	Temperature[C]	Optical Rotation Monito	Specific O.R.
1	4			25.00	0.0029	22.7241
2	4	2026/2/25 下午 02:12	373	25.00	0.0075	21.1986
3	4	2026/2/25 下午 02:12	358	25.00	0.0047	24.1538
4	4	2026/2/25 下午 02:12	299	25.00	0.0142	23.7832
5	4	2026/2/25 下午 02:12	397	25.00	0.0056	24.4198
6	4	2026/2/25 下午 02:12	401	25.00	0.0120	24.0273

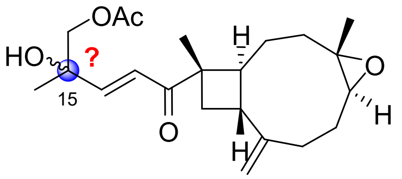

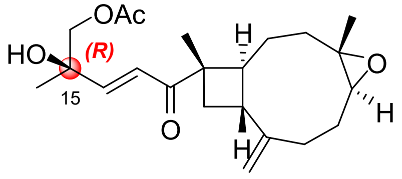

	Gibberosin H	2
**Calcd.**	+25.5 for 15*S*	-18.3 for 15*R*
**Explt.**	$[\alpha]^{25}_{D}$ **+20.0** (c 0.3, $CHCl_3$)	$[\alpha]^{25}_{D}$ **-12.6** (c 0.3, $CHCl_3$)

Figure S61. Optical Rotation of sclerohumin Q (**2**).

3. Computational details

3.1. Structures of isomers studied

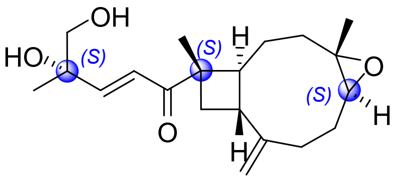

1a

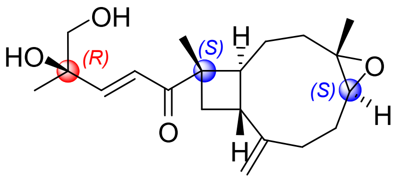

1b

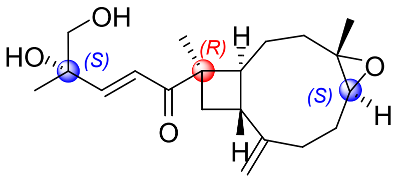

1c

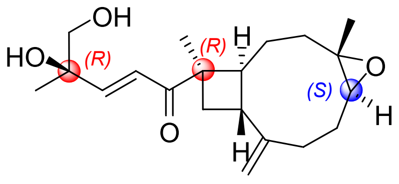

1d

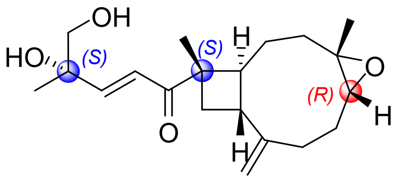

1e

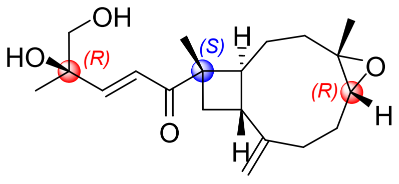

1f

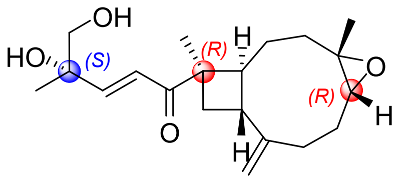

1g

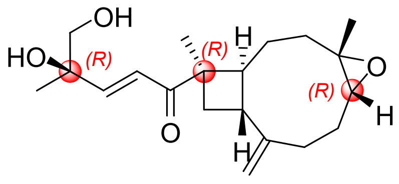

1h

Figure S62. Structures of isomers **1a-1h** of compound **1**.

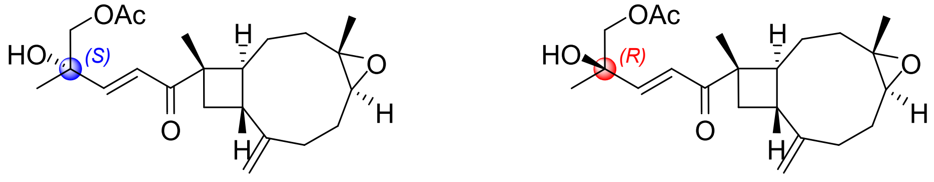

2a **2b**

Figure S63. Structures of isomers **2a** and **2b** of compound **2**.

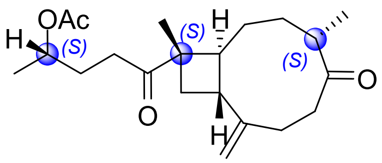

3a

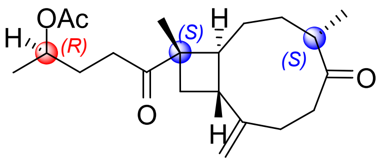

3b

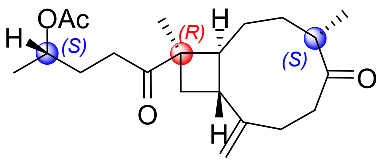

3c

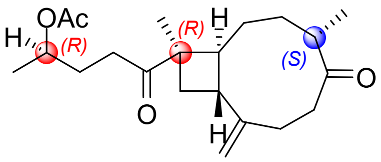

3d

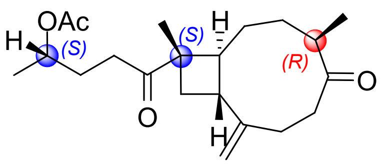

3e

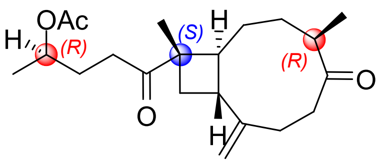

3f

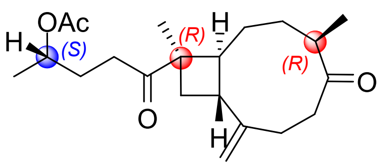

3g

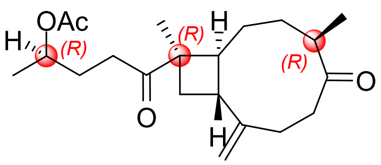

3h

Figure S64. Structures of isomers **3a-3h** of compound **3**.

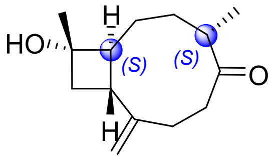

4a

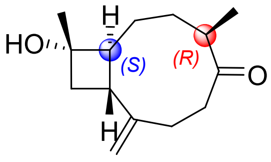

4b

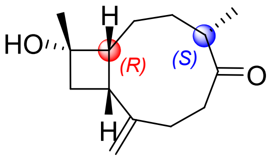

4c

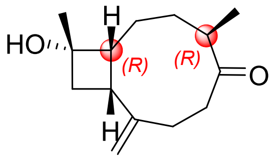

4d

Figure S65. Structures of isomers **4a-4d** of compound **4**.

3.2. Gibbs free energy of the conformers and the Boltzmann distribution

Table S9. Gibbs free energy of the conformers and the Boltzmann distribution for **1**-5S11*S*15*S*.

Conformer	Gibbs free energy (Hartree)	Boltzmann population (%)
**1**-5*S*11*S*15*S*-conf. 1	-1080.62599	2.37
**1**-5*S*11*S*15*S*-conf. 2	-1080.628243	25.79
**1**-5*S*11*S*15*S*-conf. 3	-1080.626026	2.46
**1**-5*S*11*S*15*S*-conf. 4	-1080.627253	9.04
**1**-5*S*11*S*15*S*-conf. 5	-1080.624851	0.71
**1**-5*S*11*S*15*S*-conf. 6	-1080.626424	3.76
**1**-5*S*11*S*15*S*-conf. 7	-1080.624956	0.79
**1**-5*S*11*S*15*S*-conf. 8	-1080.627383	10.37
**1**-5*S*11*S*15*S*-conf. 9	-1080.625116	0.94
**1**-5*S*11*S*15*S*-conf. 10	-1080.624892	0.74
**1**-5*S*11*S*15*S*-conf. 11	-1080.626693	4.99
**1**-5*S*11*S*15*S*-conf. 12	-1080.626703	5.05
**1**-5*S*11*S*15*S*-conf. 13	-1080.624297	0.39
**1**-5*S*11*S*15*S*-conf. 14	-1080.625807	1.95
**1**-5*S*11*S*15*S*-conf. 15	-1080.623614	0.19
**1**-5*S*11*S*15*S*-conf. 16	-1080.623554	0.18
**1**-5*S*11*S*15*S*-conf. 17	-1080.623251	0.13
**1**-5*S*11*S*15*S*-conf. 18	-1080.623182	0.12
**1**-5*S*11*S*15*S*-conf. 18	-1080.623264	0.13
**1**-5*S*11*S*15*S*-conf. 20	-1080.622584	0.06
**1**-5*S*11*S*15*S*-conf. 21	-1080.626678	4.92

**1**-5*S*11*S*15*S*-conf. 22	-1080.623221	0.13
**1**-5*S*11*S*15*S*-conf. 23	-1080.626339	3.43
**1**-5*S*11*S*15*S*-conf. 24	-1080.626356	3.50
**1**-5*S*11*S*15*S*-conf. 25	-1080.622803	0.08
**1**-5*S*11*S*15*S*-conf. 26	-1080.625935	2.24
**1**-5*S*11*S*15*S*-conf. 27	-1080.626455	3.88
**1**-5*S*11*S*15*S*-conf. 28	-1080.626468	3.94
**1**-5*S*11*S*15*S*-conf. 29	-1080.626364	3.53
**1**-5*S*11*S*15*S*-conf. 30	-1080.626525	4.18

Table S10. Gibbs free energy of the conformers and the Boltzmann distribution for **1**-5*S*11*S*15*R*.

Conformer	Gibbs free energy (Hartree)	Boltzmann population (%)
**1**-5*S*11*S*15*R*-conf. 1	-1080.625391	1.20
**1**-5*S*11*S*15*R*-conf. 2	-1080.62548	1.32
**1**-5*S*11*S*15*R*-conf. 3	-1080.627041	6.89
**1**-5*S*11*S*15*R*-conf. 4	-1080.624865	0.69
**1**-5*S*11*S*15*R*-conf. 5	-1080.626591	4.28
**1**-5*S*11*S*15*R*-conf. 6	-1080.628448	30.58
**1**-5*S*11*S*15*R*-conf. 7	-1080.625855	1.96
**1**-5*S*11*S*15*R*-conf. 8	-1080.626566	4.17
**1**-5*S*11*S*15*R*-conf. 9	-1080.627489	11.08
**1**-5*S*11*S*15*R*-conf. 10	-1080.625766	1.79
**1**-5*S*11*S*15*R*-conf. 11	-1080.625212	0.99
**1**-5*S*11*S*15*R*-conf. 12	-1080.624947	0.75
**1**-5*S*11*S*15*R*-conf. 13	-1080.626713	4.87
**1**-5*S*11*S*15*R*-conf. 14	-1080.624141	0.32
**1**-5*S*11*S*15*R*-conf. 15	-1080.623422	0.15
**1**-5*S*11*S*15*R*-conf. 16	-1080.622999	0.10
**1**-5*S*11*S*15*R*-conf. 17	-1080.62379	0.22
**1**-5*S*11*S*15*R*-conf. 18	-1080.6228	0.08
**1**-5*S*11*S*15*R*-conf. 18	-1080.623685	0.20
**1**-5*S*11*S*15*R*-conf. 20	-1080.623361	0.14
**1**-5*S*11*S*15*R*-conf. 21	-1080.623102	0.11
**1**-5*S*11*S*15*R*-conf. 22	-1080.622569	0.06

**1**-5*S*11*S*15*R*-conf. 23	-1080.626264	3.03
**1**-5*S*11*S*15*R*-conf. 24	-1080.626051	2.42
**1**-5*S*11*S*15*R*-conf. 25	-1080.627161	7.83
**1**-5*S*11*S*15*R*-conf. 26	-1080.626151	2.69
**1**-5*S*11*S*15*R*-conf. 27	-1080.62481	0.65
**1**-5*S*11*S*15*R*-conf. 28	-1080.626519	3.96
**1**-5*S*11*S*15*R*-conf. 29	-1080.627121	7.50

Table S11. Gibbs free energy of the conformers and the Boltzmann distribution for **1**-5*S*11*R*15*S*.

Conformer	Gibbs free energy (Hartree)	Boltzmann population (%)
**1**-5*S*11*R*15*S*-conf. 1	-1080.62084	0.15
**1**-5*S*11*R*15*S*-conf. 2	-1080.622787	1.22
**1**-5*S*11*R*15*S*-conf. 3	-1080.624225	5.59
**1**-5*S*11*R*15*S*-conf. 4	-1080.623929	4.08
**1**-5*S*11*R*15*S*-conf. 5	-1080.625336	18.12
**1**-5*S*11*R*15*S*-conf. 6	-1080.625789	29.28
**1**-5*S*11*R*15*S*-conf. 7	-1080.622731	1.15
**1**-5*S*11*R*15*S*-conf. 8	-1080.619779	0.05
**1**-5*S*11*R*15*S*-conf. 9	-1080.623653	3.05
**1**-5*S*11*R*15*S*-conf. 10	-1080.624273	5.88
**1**-5*S*11*R*15*S*-conf. 11	-1080.625637	24.92
**1**-5*S*11*R*15*S*-conf. 12	-1080.623497	2.58
**1**-5*S*11*R*15*S*-conf. 13	-1080.622071	0.57
**1**-5*S*11*R*15*S*-conf. 14	-1080.621824	0.44
**1**-5*S*11*R*15*S*-conf. 15	-1080.623613	2.92

Table S12. Gibbs free energy of the conformers and the Boltzmann distribution for **1**-5*S*11*R*15*R*.

Conformer	Gibbs free energy (Hartree)	Boltzmann population (%)
**1**-5*S*11*R*15*R*-conf. 1	-1080.622184	1.40
**1**-5*S*11*R*15*R*-conf. 2	-1080.623288	4.52
**1**-5*S*11*R*15*R*-conf. 3	-1080.623843	8.13
**1**-5*S*11*R*15*R*-conf. 4	-1080.620742	0.30
**1**-5*S*11*R*15*R*-conf. 5	-1080.625285	37.46
**1**-5*S*11*R*15*R*-conf. 6	-1080.6231	3.70
**1**-5*S*11*R*15*R*-conf. 7	-1080.623567	6.07
**1**-5*S*11*R*15*R*-conf. 8	-1080.624514	16.55
**1**-5*S*11*R*15*R*-conf. 9	-1080.621903	1.04
**1**-5*S*11*R*15*R*-conf. 10	-1080.622614	2.21
**1**-5*S*11*R*15*R*-conf. 11	-1080.623129	3.82
**1**-5*S*11*R*15*R*-conf. 12	-1080.617336	0.01
**1**-5*S*11*R*15*R*-conf. 13	-1080.624144	11.19
**1**-5*S*11*R*15*R*-conf. 14	-1080.620582	0.26
**1**-5*S*11*R*15*R*-conf. 15	-1080.622941	3.13
**1**-5*S*11*R*15*R*-conf. 16	-1080.620362	0.20

Table S13. Gibbs free energy of the conformers and the Boltzmann distribution for **1**-5*R*11*S*15*S*.

Conformer	Gibbs free energy (Hartree)	Boltzmann population (%)
**1**-5*R*11*S*15*S*-conf. 1	-1080.628599	7.57
**1**-5*R*11*S*15*S*-conf. 2	-1080.628807	9.44
**1**-5*R*11*S*15*S*-conf. 3	-1080.627403	2.13
**1**-5*R*11*S*15*S*-conf. 4	-1080.627988	3.96
**1**-5*R*11*S*15*S*-conf. 5	-1080.627651	2.77
**1**-5*R*11*S*15*S*-conf. 6	-1080.629659	23.27
**1**-5*R*11*S*15*S*-conf. 7	-1080.629234	14.84
**1**-5*R*11*S*15*S*-conf. 8	-1080.627158	1.65
**1**-5*R*11*S*15*S*-conf. 9	-1080.626698	1.01
**1**-5*R*11*S*15*S*-conf. 10	-1080.628639	7.90
**1**-5*R*11*S*15*S*-conf. 11	-1080.627437	2.21
**1**-5*R*11*S*15*S*-conf. 12	-1080.629181	14.03
**1**-5*R*11*S*15*S*-conf. 13	-1080.62652	0.84
**1**-5*R*11*S*15*S*-conf. 14	-1080.628637	7.88
**1**-5*R*11*S*15*S*-conf. 15	-1080.62555	0.30
**1**-5*R*11*S*15*S*-conf. 16	-1080.625044	0.18
**1**-5*R*11*S*15*S*-conf. 17	-1080.622114	0.01

Table S14. Gibbs free energy of the conformers and the Boltzmann distribution for **1**-5*R*11*S*15*R*.

Conformer	Gibbs free energy (Hartree)	Boltzmann population (%)
**1**-5*R*11*S*15*R*-conf. 1	-1080.627572	3.08
**1**-5*R*11*S*15*R*-conf. 2	-1080.628221	6.13
**1**-5*R*11*S*15*R*-conf. 3	-1080.628988	13.81
**1**-5*R*11*S*15*R*-conf. 4	-1080.627174	2.02
**1**-5*R*11*S*15*R*-conf. 5	-1080.628057	5.15
**1**-5*R*11*S*15*R*-conf. 6	-1080.627929	4.50
**1**-5*R*11*S*15*R*-conf. 7	-1080.628456	7.86
**1**-5*R*11*S*15*R*-conf. 8	-1080.628322	6.82
**1**-5*R*11*S*15*R*-conf. 9	-1080.629452	22.58
**1**-5*R*11*S*15*R*-conf. 10	-1080.62727	2.24
**1**-5*R*11*S*15*R*-conf. 11	-1080.628226	6.16
**1**-5*R*11*S*15*R*-conf. 12	-1080.627147	1.97
**1**-5*R*11*S*15*R*-conf. 13	-1080.629132	16.09
**1**-5*R*11*S*15*R*-conf. 14	-1080.626388	0.88
**1**-5*R*11*S*15*R*-conf. 15	-1080.626166	0.70

Table S15. Gibbs free energy of the conformers and the Boltzmann distribution for **1**-5*R*11*R*15*S*.

Conformer	Gibbs free energy (Hartree)	Boltzmann population (%)
**1**-5*R*11*R*15*S*-conf. 1	-1080.627372	12.44
**1**-5*R*11*R*15*S*-conf. 2	-1080.626124	3.32
**1**-5*R*11*R*15*S*-conf. 3	-1080.628361	35.46
**1**-5*R*11*R*15*S*-conf. 4	-1080.623696	0.25
**1**-5*R*11*R*15*S*-conf. 5	-1080.624784	0.80
**1**-5*R*11*R*15*S*-conf. 6	-1080.624729	0.76
**1**-5*R*11*R*15*S*-conf. 7	-1080.626473	4.80
**1**-5*R*11*R*15*S*-conf. 8	-1080.623173	0.15
**1**-5*R*11*R*15*S*-conf. 9	-1080.626306	4.02
**1**-5*R*11*R*15*S*-conf. 10	-1080.627194	10.30
**1**-5*R*11*R*15*S*-conf. 11	-1080.628049	25.48
**1**-5*R*11*R*15*S*-conf. 12	-1080.624635	0.69
**1**-5*R*11*R*15*S*-conf. 13	-1080.621326	0.02
**1**-5*R*11*R*15*S*-conf. 14	-1080.62517	1.21
**1**-5*R*11*R*15*S*-conf. 15	-1080.623877	0.31

Table S16. Gibbs free energy of the conformers and the Boltzmann distribution for **1**-5*R*11*R*15*R*.

Conformer	Gibbs free energy (Hartree)	Boltzmann population (%)
**1**-5*R*11*R*15*R*-conf. 1	-1080.624541	1.37
**1**-5*R*11*R*15*R*-conf. 2	-1080.62594	6.03
**1**-5*R*11*R*15*R*-conf. 3	-1080.627503	31.57
**1**-5*R*11*R*15*R*-conf. 4	-1080.624614	1.48
**1**-5*R*11*R*15*R*-conf. 5	-1080.625541	3.95
**1**-5*R*11*R*15*R*-conf. 6	-1080.623422	0.42
**1**-5*R*11*R*15*R*-conf. 7	-1080.626229	8.19
**1**-5*R*11*R*15*R*-conf. 8	-1080.627751	41.05
**1**-5*R*11*R*15*R*-conf. 9	-1080.623742	0.59
**1**-5*R*11*R*15*R*-conf. 10	-1080.624674	1.58
**1**-5*R*11*R*15*R*-conf. 11	-1080.621736	0.07
**1**-5*R*11*R*15*R*-conf. 12	-1080.624091	0.85
**1**-5*R*11*R*15*R*-conf. 13	-1080.624985	2.19
**1**-5*R*11*R*15*R*-conf. 14	-1080.619179	0.00
**1**-5*R*11*R*15*R*-conf. 15	-1080.623837	0.65

Table S17. Gibbs free energy of the conformers and the Boltzmann distribution for **2**-15*S*.

Conformer	Gibbs free energy (Hartree)	Boltzmann population (%)
**2**-15*S*-conf. 1	-1233.264872	9.84
**2**-15*S*-conf. 2	-1233.2649	10.14
**2**-15*S*-conf. 3	-1233.265394	17.11
**2**-15*S*-conf. 4	-1233.264199	4.83
**2**-15*S*-conf. 5	-1233.264234	5.01
**2**-15*S*-conf. 6	-1233.264357	5.71
**2**-15*S*-conf. 7	-1233.265464	18.43
**2**-15*S*-conf. 8	-1233.263896	3.50
**2**-15*S*-conf. 9	-1233.25925	0.03
**2**-15*S*-conf. 10	-1233.263123	1.54
**2**-15*S*-conf. 11	-1233.260105	0.06
**2**-15*S*-conf. 12	-1233.260108	0.06
**2**-15*S*-conf. 13	-1233.259104	0.02
**2**-15*S*-conf. 14	-1233.264811	9.23
**2**-15*S*-conf. 15	-1233.259603	0.04
**2**-15*S*-conf. 16	-1233.265234	14.44
**2**-15*S*-conf. 17	-1233.258703	0.01

Table S18. Gibbs free energy of the conformers and the Boltzmann distribution for **2**-15*R*.

Conformer	Gibbs free energy (Hartree)	Boltzmann population (%)
**2**-15*R*-conf. 1	-1233.261214	0.14
**2**-15*R*-conf. 2	-1233.263631	1.83
**2**-15*R*-conf. 3	-1233.265358	11.41
**2**-15*R*-conf. 4	-1233.265343	11.23
**2**-15*R*-conf. 5	-1233.264252	3.54
**2**-15*R*-conf. 6	-1233.264785	6.22
**2**-15*R*-conf. 7	-1233.261627	0.22
**2**-15*R*-conf. 8	-1233.263894	2.42
**2**-15*R*-conf. 9	-1233.259413	0.02
**2**-15*R*-conf. 10	-1233.259746	0.03
**2**-15*R*-conf. 11	-1233.262309	0.45
**2**-15*R*-conf. 12	-1233.265074	8.45
**2**-15*R*-conf. 13	-1233.265721	16.76
**2**-15*R*-conf. 14	-1233.25924	0.02
**2**-15*R*-conf. 15	-1233.264965	7.53
**2**-15*R*-conf. 16	-1233.258411	0.01
**2**-15*R*-conf. 17	-1233.262122	0.37
**2**-15*R*-conf. 18	-1233.264169	3.24
**2**-15*R*-conf. 19	-1233.264769	6.12
**2**-15*R*-conf. 20	-1233.264974	7.60
**2**-15*R*-conf. 21	-1233.264718	5.79
**2**-15*R*-conf. 22	-1233.264199	3.34

**2**-15*R*-conf. 23	-1233.264173	3.25

Table S19. Gibbs free energy of the conformers and the Boltzmann distribution for **3**-4*S*11*S*15*S*.

Conformer	Gibbs free energy (Hartree)	Boltzmann population (%)
**3**-4*S*11*S*15*S*-conf. 1	-1120.009583	31.73
**3**-4*S*11*S*15*S*-conf. 2	-1120.006835	1.73
**3**-4*S*11*S*15*S*-conf. 3	-1112.897755	0.00
**3**-4*S*11*S*15*S*-conf. 4	-1120.009522	29.75
**3**-4*S*11*S*15*S*-conf. 5	-1120.003904	0.08
**3**-4*S*11*S*15*S*-conf. 6	-1120.008208	7.40
**3**-4*S*11*S*15*S*-conf. 7	-1120.00661	1.36
**3**-4*S*11*S*15*S*-conf. 8	-1120.000006	0.00
**3**-4*S*11*S*15*S*-conf. 9	-1120.00415	0.10
**3**-4*S*11*S*15*S*-conf. 10	-1120.000245	0.00
**3**-4*S*11*S*15*S*-conf. 11	-1120.008186	7.23
**3**-4*S*11*S*15*S*-conf. 12	-1120.004199	0.11
**3**-4*S*11*S*15*S*-conf. 13	-1120.000215	0.00
**3**-4*S*11*S*15*S*-conf. 14	-1120.009171	20.51

Table S20. Gibbs free energy of the conformers and the Boltzmann distribution for **3**-4*S*11*S*15*R*.

Conformer	Gibbs free energy (Hartree)	Boltzmann population (%)
**3**-4*S*11*S*15*R*-conf. 1	-1120.004515	0.10
**3**-4*S*11*S*15*R*-conf. 2	-1120.005929	0.45
**3**-4*S*11*S*15*R*-conf. 3	-1120.0052	0.21
**3**-4*S*11*S*15*R*-conf. 4	-1120.005988	0.48
**3**-4*S*11*S*15*R*-conf. 5	-1120.004275	0.08
**3**-4*S*11*S*15*R*-conf. 6	-1120.008301	5.56
**3**-4*S*11*S*15*R*-conf. 7	-1120.004816	0.14
**3**-4*S*11*S*15*R*-conf. 8	-1120.008968	11.27
**3**-4*S*11*S*15*R*-conf. 9	-1120.009107	13.06
**3**-4*S*11*S*15*R*-conf. 10	-1120.008314	5.64
**3**-4*S*11*S*15*R*-conf. 11	-1120.008328	5.72
**3**-4*S*11*S*15*R*-conf. 12	-1120.00831	5.61
**3**-4*S*11*S*15*R*-conf. 13	-1120.00563	0.33
**3**-4*S*11*S*15*R*-conf. 14	-1120.004025	0.06
**3**-4*S*11*S*15*R*-conf. 15	-1120.009099	12.95
**3**-4*S*11*S*15*R*-conf. 16	-1120.009387	17.57
**3**-4*S*11*S*15*R*-conf. 17	-1120.009544	20.75
**3**-4*S*11*S*15*R*-conf. 18	-1120.001518	0.00
**3**-4*S*11*S*15*R*-conf. 19	-1120.000064	0.00
**3**-4*S*11*S*15*R*-conf. 20	-1120.002431	0.01
**3**-4*S*11*S*15*R*-conf. 21	-1120.001166	0.00

Table S21. Gibbs free energy of the conformers and the Boltzmann distribution for **3**-4*S*11*R*15*S*.

**Conformer**	**Gibbs free energy (Hartree)**	**Boltzmann population (%)**
**3**-4*S*11*R*15*S*-conf. 1	-1120.00627	19.84
**3**-4*S*11*R*15*S*-conf. 2	-1120.005238	6.65
**3**-4*S*11*R*15*S*-conf. 3	-1120.006262	19.67
**3**-4*S*11*R*15*S*-conf. 4	-1120.000578	0.05
**3**-4*S*11*R*15*S*-conf. 5	-1120.005365	7.61
**3**-4*S*11*R*15*S*-conf. 6	-1120.005234	6.62
**3**-4*S*11*R*15*S*-conf. 7	-1120.005776	11.76
**3**-4*S*11*R*15*S*-conf. 8	-1120.00617	17.84
**3**-4*S*11*R*15*S*-conf. 9	-1120.000278	0.03
**3**-4*S*11*R*15*S*-conf. 10	-1120.005518	8.95
**3**-4*S*11*R*15*S*-conf. 11	-1120.003334	0.89
**3**-4*S*11*R*15*S*-conf. 12	-1120.000057	0.03
**3**-4*S*11*R*15*S*-conf. 13	-1120.000928	0.07

Table S22. Gibbs free energy of the conformers and the Boltzmann distribution for **3**-4*S*11*R*15*R*.

**Conformer**	**Gibbs free energy (Hartree)**	**Boltzmann population (%)**
**3**-4*S*11*R*15*R*-conf. 1	-1120.002651	70.89
**3**-4*S*11*R*15*R*-conf. 2	-1120.001718	26.39
**3**-4*S*11*R*15*R*-conf. 3	-1119.999572	2.72

Table S23. Gibbs free energy of the conformers and the Boltzmann distribution for **3**-4*R*11*S*15*S*.

Conformer	Gibbs free energy (Hartree)	Boltzmann population (%)
**3**-4*R*11*S*15*S*-conf. 1	-1120.010106	32.33
**3**-4*R*11*S*15*S*-conf. 2	-1120.008735	7.57
**3**-4*R*11*S*15*S*-conf. 3	-1120.008251	4.53
**3**-4*R*11*S*15*S*-conf. 4	-1120.003602	0.03
**3**-4*R*11*S*15*S*-conf. 5	-1120.003504	0.03
**3**-4*R*11*S*15*S*-conf. 6	-1120.008747	7.66
**3**-4*R*11*S*15*S*-conf. 7	-1120.010239	37.22
**3**-4*R*11*S*15*S*-conf. 8	-1120.004486	0.08
**3**-4*R*11*S*15*S*-conf. 9	-1120.008567	6.33
**3**-4*R*11*S*15*S*-conf. 10	-1120.006535	0.74
**3**-4*R*11*S*15*S*-conf. 11	-1120.005215	0.18
**3**-4*R*11*S*15*S*-conf. 12	-1120.006872	1.05
**3**-4*R*11*S*15*S*-conf. 13	-1120.004668	0.10
**3**-4*R*11*S*15*S*-conf. 14	-1120.004668	0.10
**3**-4*R*11*S*15*S*-conf. 15	-1120.007496	2.04

Table S24. Gibbs free energy of the conformers and the Boltzmann distribution for **3**-4*R*11*S*15*R*.

**Conformer**	**Gibbs free energy (Hartree)**	**Boltzmann population (%)**
**3**-4*R*11*S*15*R*-conf. 1	-1120.004477	0.13
**3**-4*R*11*S*15*R*-conf. 2	-1120.006057	0.69
**3**-4*R*11*S*15*R*-conf. 3	-1120.008592	10.08
**3**-4*R*11*S*15*R*-conf. 4	-1120.00792	4.95
**3**-4*R*11*S*15*R*-conf. 5	-1120.00504	0.23
**3**-4*R*11*S*15*R*-conf. 6	-1120.004511	0.13
**3**-4*R*11*S*15*R*-conf. 7	-1120.009751	34.39
**3**-4*R*11*S*15*R*-conf. 8	-1120.00792	4.95
**3**-4*R*11*S*15*R*-conf. 9	-1120.007915	4.92
**3**-4*R*11*S*15*R*-conf. 10	-1120.004373	0.12
**3**-4*R*11*S*15*R*-conf. 11	-1120.00792	4.95
**3**-4*R*11*S*15*R*-conf. 12	-1120.009752	34.42
**3**-4*R*11*S*15*R*-conf. 13	-1120.003661	0.05

Table S25. Gibbs free energy of the conformers and the Boltzmann distribution for **3**-4*R*11*R*15*S*.

Conformer	Gibbs free energy (Hartree)	Boltzmann population (%)
**3**-4*R*11*R*15*S*-conf. 1	-1120.005207	8.81
**3**-4*R*11*R*15*S*-conf. 2	-1120.006301	28.07
**3**-4*R*11*R*15*S*-conf. 3	-1120.005349	10.24
**3**-4*R*11*R*15*S*-conf. 4	-1120.000098	0.04
**3**-4*R*11*R*15*S*-conf. 5	-1120.001117	0.12
**3**-4*R*11*R*15*S*-conf. 6	-1120.005339	10.13
**3**-4*R*11*R*15*S*-conf. 7	-1120.00469	5.10
**3**-4*R*11*R*15*S*-conf. 8	-1119.99971	0.03
**3**-4*R*11*R*15*S*-conf. 9	-1120.001508	0.18
**3**-4*R*11*R*15*S*-conf. 10	-1119.999176	0.01
**3**-4*R*11*R*15*S*-conf. 11	-1120.006283	27.54
**3**-4*R*11*R*15*S*-conf. 12	-1120.000869	0.09
**3**-4*R*11*R*15*S*-conf. 13	-1119.999644	0.02
**3**-4*R*11*R*15*S*-conf. 14	-1120.00529	9.62

Table S26. Gibbs free energy of the conformers and the Boltzmann distribution for **3**-4*R*11*R*15*R*.

Conformer	Gibbs free energy (Hartree)	Boltzmann population (%)
**3**-4*R*11*R*15*R*-conf. 1	-1120.005735	14.42
**3**-4*R*11*R*15*R*-conf. 2	-1120.005207	8.24
**3**-4*R*11*R*15*R*-conf. 3	-1120.004591	4.29
**3**-4*R*11*R*15*R*-conf. 4	-1120.006301	26.26
**3**-4*R*11*R*15*R*-conf. 5	-1120.005336	9.45
**3**-4*R*11*R*15*R*-conf. 6	-1120.000098	0.04
**3**-4*R*11*R*15*R*-conf. 7	-1119.999472	0.02
**3**-4*R*11*R*15*R*-conf. 8	-1120.005268	8.79
**3**-4*R*11*R*15*R*-conf. 9	-1120.001117	0.11
**3**-4*R*11*R*15*R*-conf. 10	-1120.002221	0.35
**3**-4*R*11*R*15*R*-conf. 11	-1120.005691	13.76
**3**-4*R*11*R*15*R*-conf. 12	-1119.999862	0.03
**3**-4*R*11*R*15*R*-conf. 13	-1120.005337	9.46
**3**-4*R*11*R*15*R*-conf. 14	-1120.00469	4.77
**3**-4*R*11*R*15*R*-conf. 15	-1119.999712	0.02

Table S27. Gibbs free energy of the conformers and the Boltzmann distribution for **4**-1*S*4*S*.

Conformer	Gibbs free energy (Hartree)	Boltzmann population (%)
**4**-1*S*4*S*-conf. 1	-696.887711	34.87
**4**-1*S*4*S*-conf. 2	-696.887477	27.22
**4**-1*S*4*S*-conf. 3	-696.88779	37.91

Table S28. Gibbs free energy of the conformers and the Boltzmann distribution for **4**-1*S*4*R*.

Conformer	Gibbs free energy (Hartree)	Boltzmann population (%)
**4**-1*S*4*R*-conf. 1	-696.88812	48.35
**4**-1*S*4*R*-conf. 2	-696.887718	31.58
**4**-1*S*4*R*-conf. 3	-696.88729	20.07

Table S29. Gibbs free energy of the conformers and the Boltzmann distribution for **4**-1*R*4*S*.

Conformer	Gibbs free energy (Hartree)	Boltzmann population (%)
**4**-1*R*4*S*-conf. 1	-696.881106	16.80
**4**-1*R*4*S*-conf. 2	-696.881674	30.65
**4**-1*R*4*S*-conf. 3	-696.87936	2.64
**4**-1*R*4*S*-conf. 4	-696.880025	5.35
**4**-1*R*4*S*-conf. 5	-696.879435	2.86
**4**-1*R*4*S*-conf. 6	-696.879465	2.95
**4**-1*R*4*S*-conf. 7	-696.88155	26.88
**4**-1*R*4*S*-conf. 8	-696.875281	0.04
**4**-1*R*4*S*-conf. 9	-696.880775	11.83

Table S30. Gibbs free energy of the conformers and the Boltzmann distribution for **4**-1*R*4*R*.

**Conformer**	**Gibbs free energy (Hartree)**	**Boltzmann population (%)**
**4**-1*R*4*R*-conf. 1	-696.88204	2.44
**4**-1*R*4*R*-conf. 2	-696.882626	4.53
**4**-1*R*4*R*-conf. 3	-696.883509	11.54
**4**-1*R*4*R*-conf. 4	-696.88429	26.39
**4**-1*R*4*R*-conf. 5	-696.883236	8.64
**4**-1*R*4*R*-conf. 6	-696.883935	18.12
**4**-1*R*4*R*-conf. 7	-696.883086	7.37
**4**-1*R*4*R*-conf. 8	-696.883941	18.24
**4**-1*R*4*R*-conf. 9	-696.882146	2.72

3.3. The cartesian coordinates of the dominant conformers of compounds 1-4

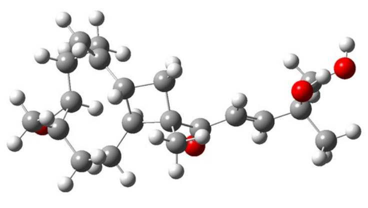	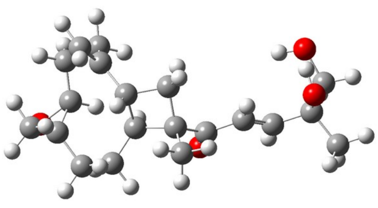	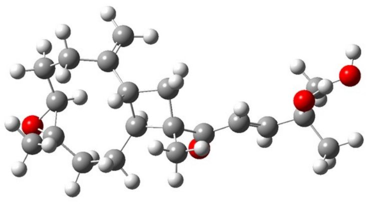
**1**-5*S*11*S*15*S*-conf. 1 (2.37%)	**1**-5*S*11*S*15*S*-conf. 2 (25.79%)	**1**-5*S*11*S*15*S*-conf. 3 (2.46%)
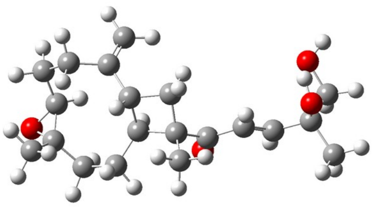	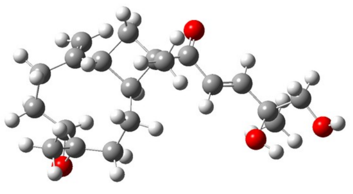	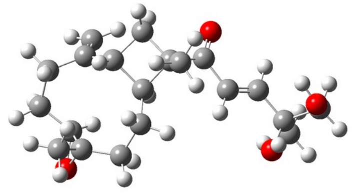
**1**-5*S*11*S*15*S*-conf. 4 (9.04%)	**1**-5*S*11*S*15*S*-conf. 6 (3.76%)	**1**-5*S*11*S*15*S*-conf. 8 (10.37%)
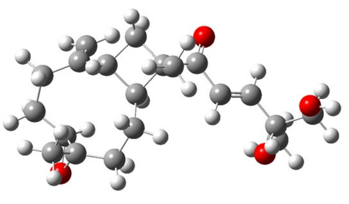	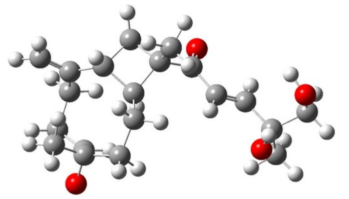	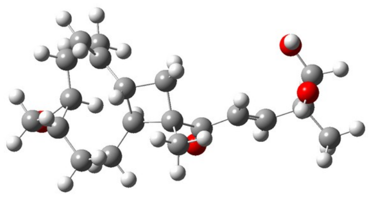
**1**-5*S*11*S*15*S*-conf. 11 (4.99%)	**1**-5*S*11*S*15*S*-conf. 12 (5.05%)	**1**-5*S*11*S*15*S*-conf. 21 (4.92%)
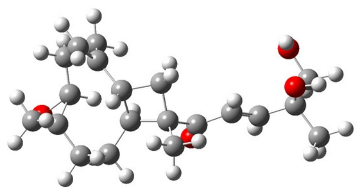	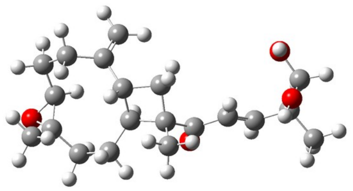	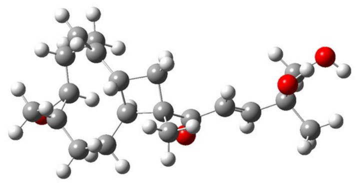
**1**-5*S*11*S*15*S*-conf. 23 (3.43%)	**1**-5*S*11*S*15*S*-conf. 24 (3.50%)	**1**-5*S*11*S*15*S*-conf. 26 (2.24%)
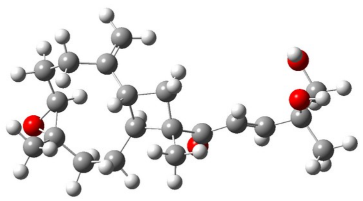	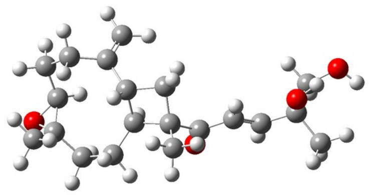	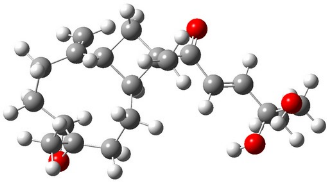
**1**-5*S*11*S*15*S*-conf. 27 (3.88%)	**1**-5*S*11*S*15*S*-conf. 28 (3.94%)	**1**-5*S*11*S*15*S*-conf. 29 (3.53%)

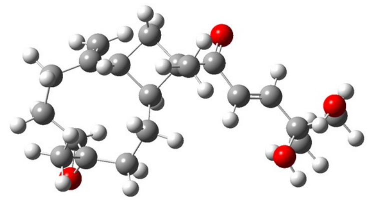

**1**-5*S*11*S*15*S*-conf. 30 (4.18%)

Figure S66. The cartesian coordinates of the dominant conformers for conformers **1**-5*S*11*S*15*S*.

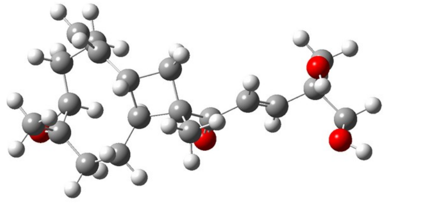	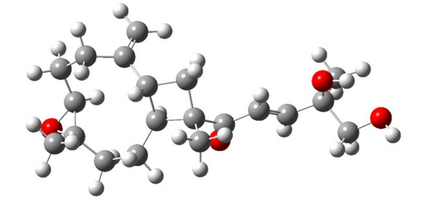	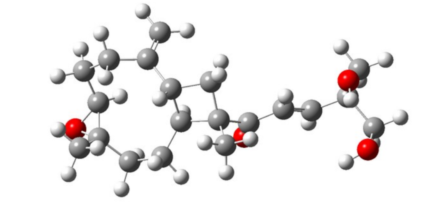
**1**-5*S*11*S*15*R*-conf. 3 (6.89%)	**1**-5*S*11*S*15*R*-conf. 5 (4.28%)	**1**-5*S*11*S*15*R*-conf. 6 (30.58%)
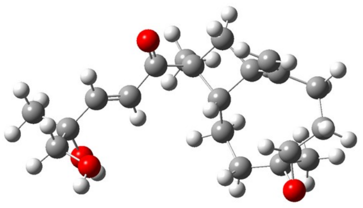	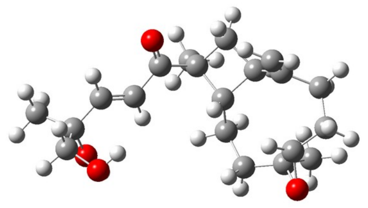	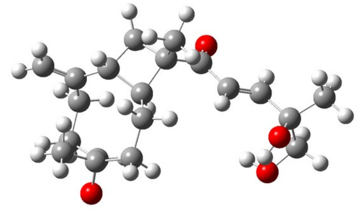
**1**-5*S*11*S*15*R*-conf. 8 (4.17%)	**1**-5*S*11*S*15*R*-conf. 9 (11.08%)	**1**-5*S*11*S*15*R*-conf. 13 (4.87%)
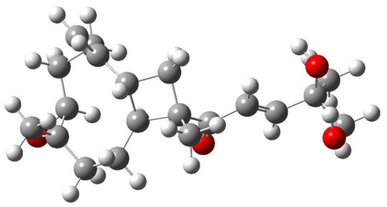	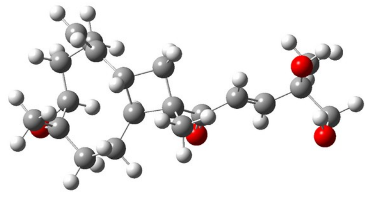	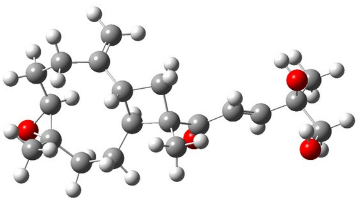
**1**-5*S*11*S*15*R*-conf. 23 (3.03%)	**1**-5*S*11*S*15*R*-conf. 24 (2.42%)	**1**-5*S*11*S*15*R*-conf. 25 (7.83%)
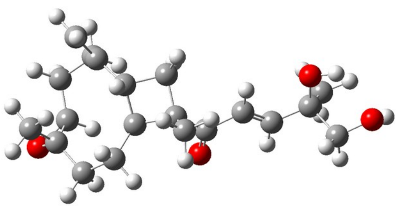	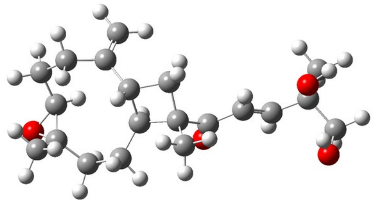	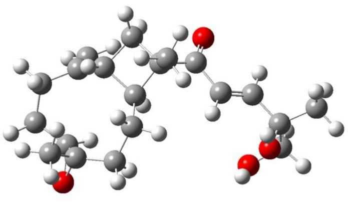
**1**-5*S*11*S*15*R*-conf. 26 (2.69%)	**1**-5*S*11*S*15*R*-conf. 28 (3.96%)	**1**-5*S*11*S*15*R*-conf. 29 (7.50%)

Figure S67. The cartesian coordinates of the dominant conformers for conformers **1**-5*S*11*S*15*R*.

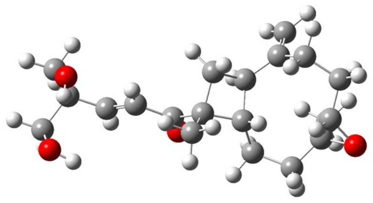	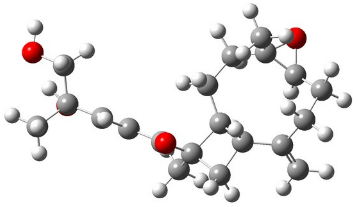	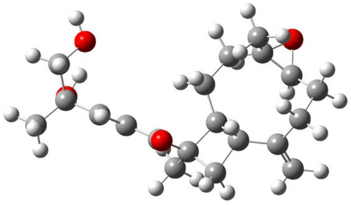
**1**-5*S*11*R*15*S*-conf. 3 (5.59%)	**1**-5*S*11*R*15*S*-conf. 4 (4.08%)	**1**-5*S*11*R*15*S*-conf. 5 (18.12%)
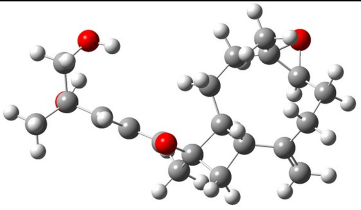	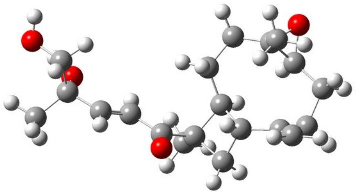	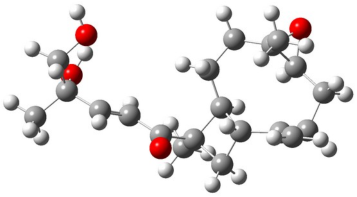
**1**-5*S*11*R*15*S*-conf. 6 (29.28%)	**1**-5*S*11*R*15*S*-conf. 9 (3.05%)	**1**-5*S*11*R*15*S*-conf. 10 (5.88%)
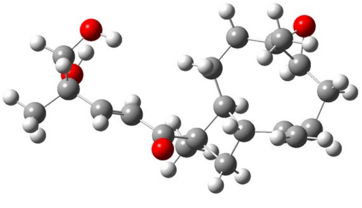	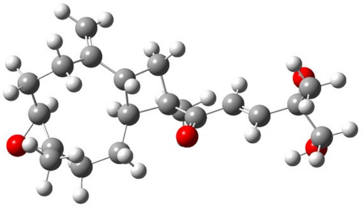	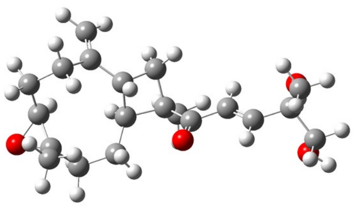
**1**-5*S*11*R*15*S*-conf. 11 (24.92%)	**1**-5*S*11*R*15*S*-conf. 12 (2.58%)	**1**-5*S*11*R*15*S*-conf. 15 (2.92%)

Figure S68. The cartesian coordinates of the dominant conformers for conformers **1**-5*S*11*R*15*S*.

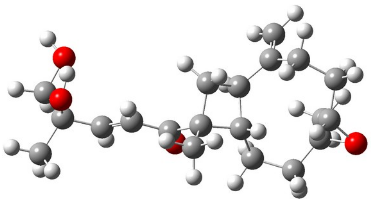	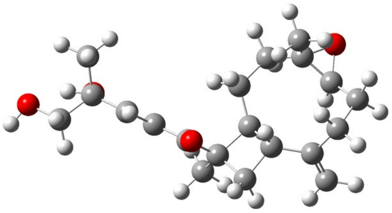	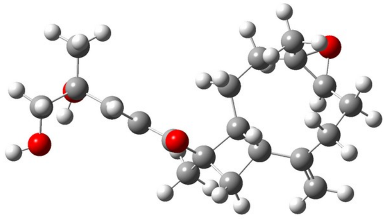
**1**-5*S*11*R*15*R*-conf. 2 (4.52%)	**1**-5*S*11*R*15*R*-conf. 3 (8.13%)	**1**-5*S*11*R*15*R*-conf. 5 (37.46%)
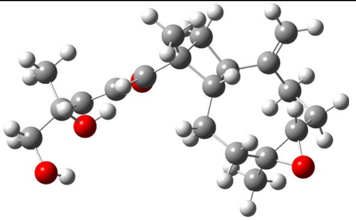	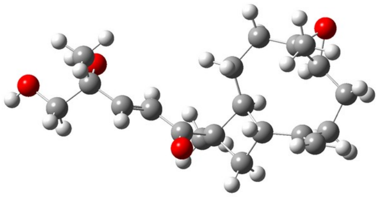	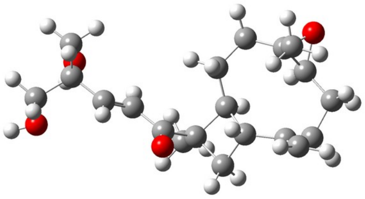
**1**-5*S*11*R*15*R*-conf. 6 (3.70%)	**1**-5*S*11*R*15*R*-conf. 7 (6.07%)	**1**-5*S*11*R*15*R*-conf. 8 (16.55%)
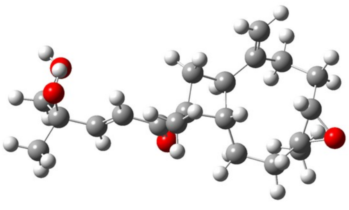	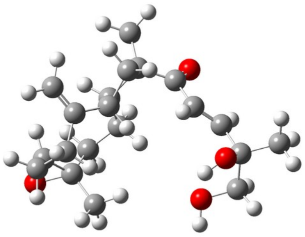	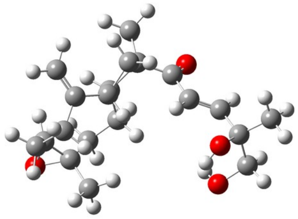
**1**-5*S*11*R*15*R*-conf. 10 (2.21%)	**1**-5*S*11*R*15*R*-conf. 11 (3.82%)	**1**-5*S*11*R*15*R*-conf. 13 (11.19%)
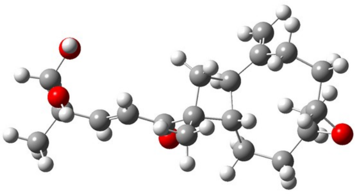		
**1**-5*S*11*R*15*R*-conf. 15 (3.13%)		

Figure S69. The cartesian coordinates of the dominant conformers for conformers **1**-5*S*11*R*15*R*.

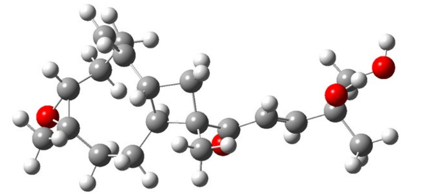	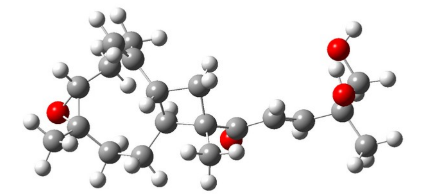	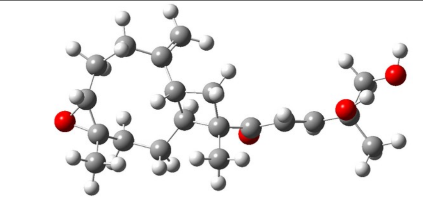
**1**-5*R*11*S*15*S*-conf. 1 (7.57%)	**1**-5*R*11*S*15*S*-conf. 2 (9.44%)	**1**-5*R*11*S*15*S*-conf. 3 (2.13%)
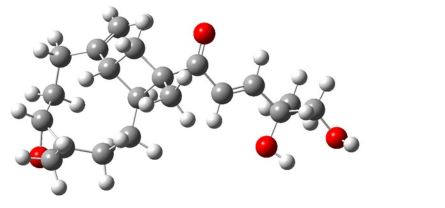	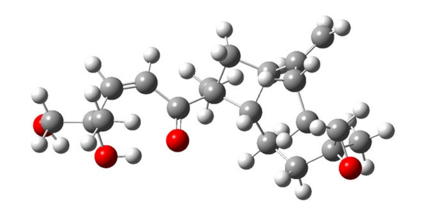	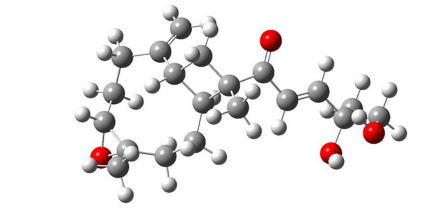
**1**-5*R*11*S*15*S*-conf. 4 (3.96%)	**1**-5*R*11*S*15*S*-conf. 5 (2.77%)	**1**-5*R*11*S*15*S*-conf. 6 (23.27%)
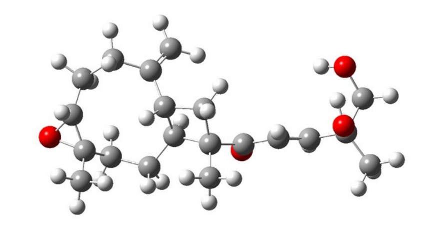	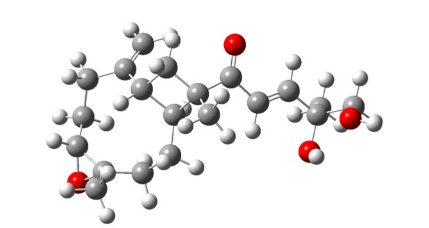	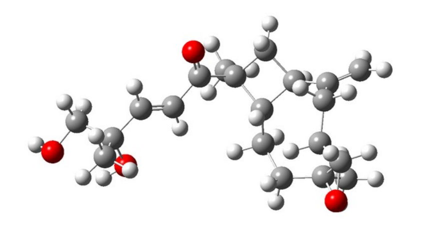
**1**-5*R*11*S*15*S*-conf. 7 (14.84%)	**1**-5*R*11*S*15*S*-conf. 10 (7.90%)	**1**-5*R*11*S*15*S*-conf. 11 (2.21%)
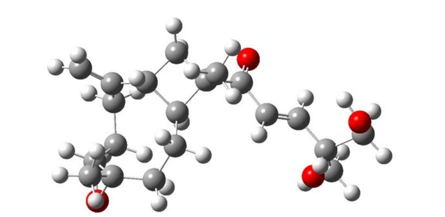	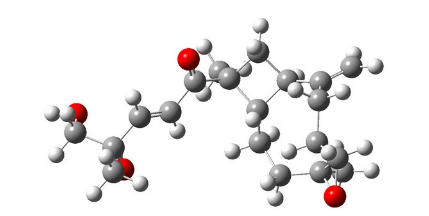	
**1**-5*R*11*S*15*S*-conf. 12 (14.03%)	**1**-5*R*11*S*15*S*-conf. 14 (7.88%)	

Figure S70. The cartesian coordinates of the dominant conformers for conformers **1**-5*R*11*S*15*S*.

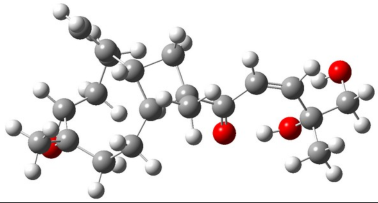	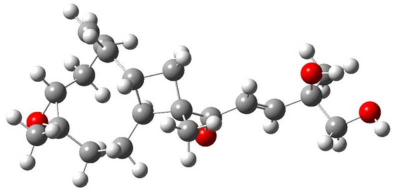	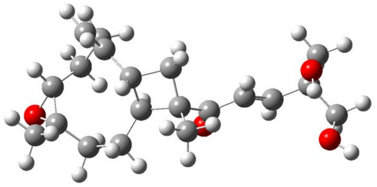
**1**-5*R*11*S*15*R*-conf. 1 (3.08%)	**1**-5*R*11*S*15*R*-conf. 2 (6.13%)	**1**-5*R*11*S*15*R*-conf. 3 (13.81%)
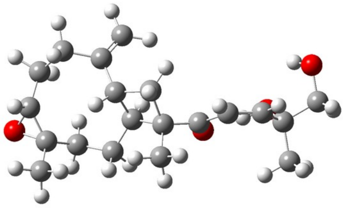	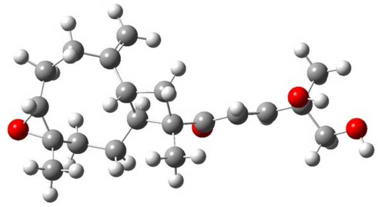	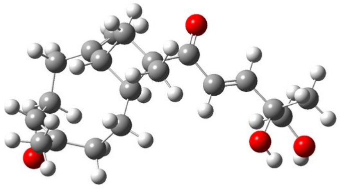
**1**-5*R*11*S*15*R*-conf. 4 (2.02%)	**1**-5*R*11*S*15*R*-conf. 5 (5.15%)	**1**-5*R*11*S*15*R*-conf. 6 (4.50%)
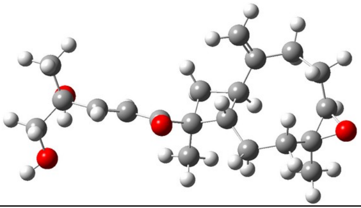	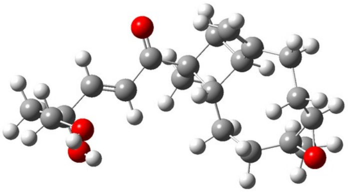	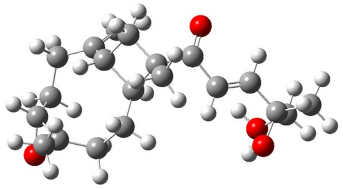
**1**-5*R*11*S*15*R*-conf. 7 (7.86%)	**1**-5*R*11*S*15*R*-conf. 8 (6.82%)	**1**-5*R*11*S*15*R*-conf. 9 (22.58%)
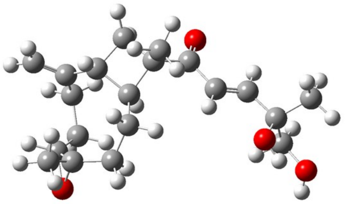	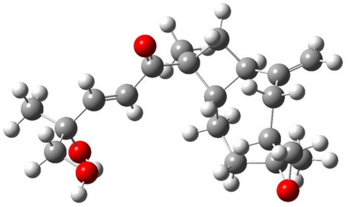	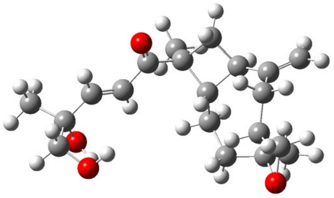
**1**-5*R*11*S*15*R*-conf. 10 (2.24%)	**1**-5*R*11*S*15*R*-conf. 11 (6.16%)	**1**-5*R*11*S*15*R*-conf. 13 (16.09%)

Figure S71. The cartesian coordinates of the dominant conformers for conformers **1**-5*R*11*S*15*R*.

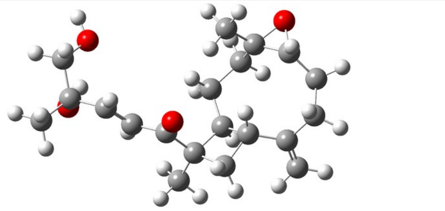	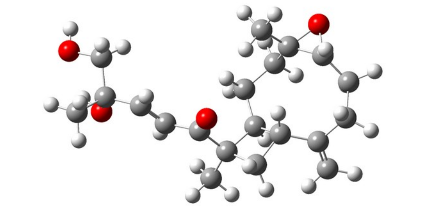	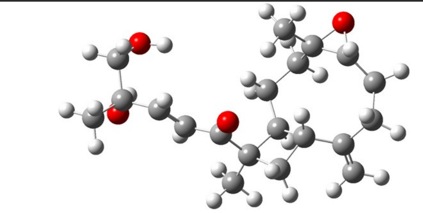
**1**-5*R*11*R*15*S*-conf. 1 (12.44%)	**1**-5*R*11*R*15*S*-conf. 2 (3.32%)	**1**-5*R*11*R*15*S*-conf. 3 (35.46%)
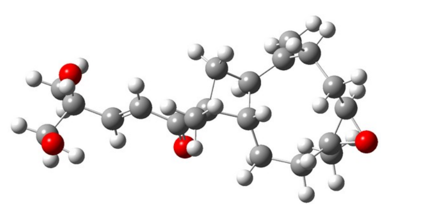	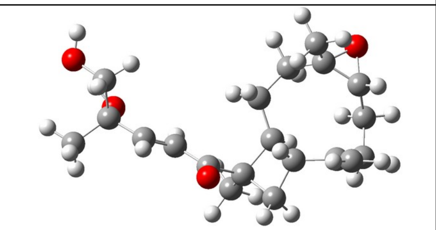	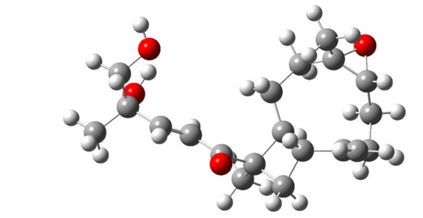
**1**-5*R*11*R*15*S*-conf. 7 (4.80%)	**1**-5*R*11*R*15*S*-conf. 9 (4.02%)	**1**-5*R*11*R*15*S*-conf. 10 (10.30%)
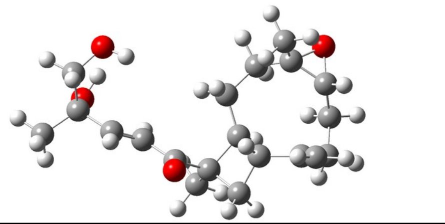		
**1**-5*R*11*R*15*S*-conf. 11 (25.48%)		

Figure S72. The cartesian coordinates of the dominant conformers for conformers **1**-5*R*11*R*15*S*.

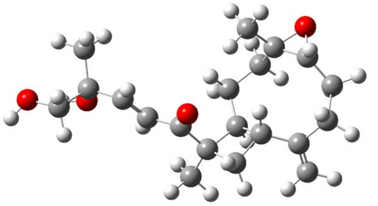	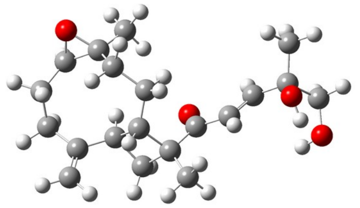	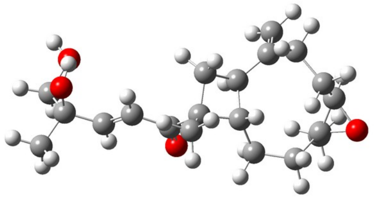
**1**-5*R*11*R*15*R*-conf. 2 (6.03%)	**1**-5*R*11*R*15*R*-conf. 3 (31.57%)	**1**-5*R*11*R*15*R*-conf. 5 (3.95%)
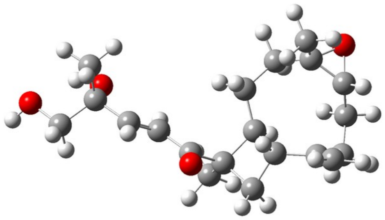	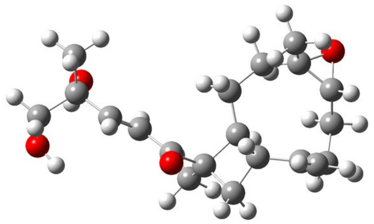	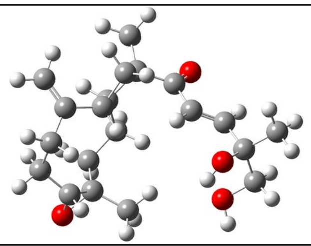
**1**-5*R*11*R*15*R*-conf. 7 (8.19%)	**1**-5*R*11*R*15*R*-conf. 8 (41.05%)	**1**-5*R*11*R*15*R*-conf. 13 (2.19%)

Figure S73. The cartesian coordinates of the dominant conformers for conformers **1**-5*R*11*R*15*R*.

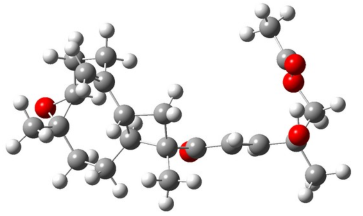	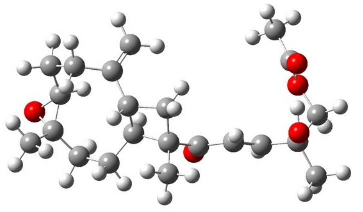	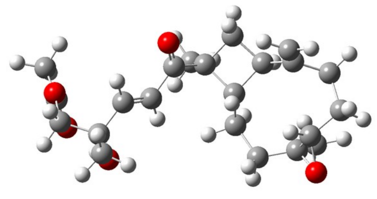
**2**-15*S*-conf. 1 (9.84%)	**2**-15*S*-conf. 2 (10.14%)	**2**-15*S*-conf. 3 (17.11%)
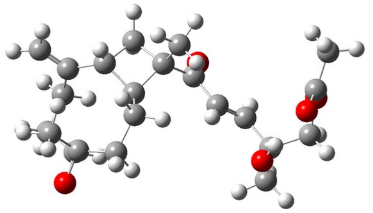	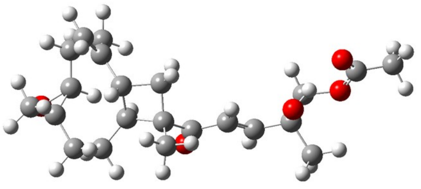	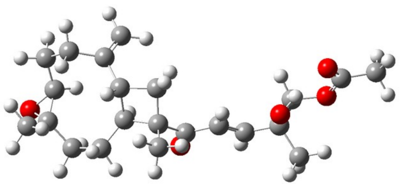
**2**-15*S*-conf. 4 (4.83%)	**2**-15*S*-conf. 5 (5.01%)	**2**-15*S*-conf. 6 (5.71%)
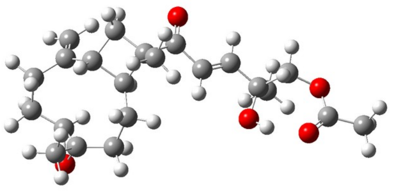	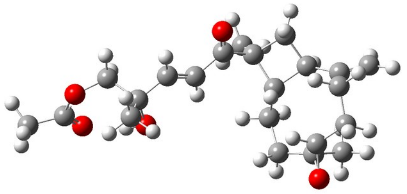	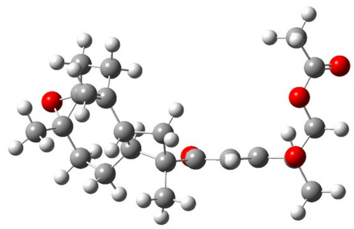
**2**-15*S*-conf. 7 (18.43%)	**2**-15*S*-conf. 8 (3.50%)	**2**-15*S*-conf. 14 (9.23%)
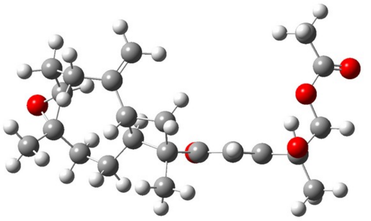		
**2**-15*S*-conf. 16 (14.44%)		

Figure S74. The cartesian coordinates of the dominant conformers for conformers **2**-15*S*.

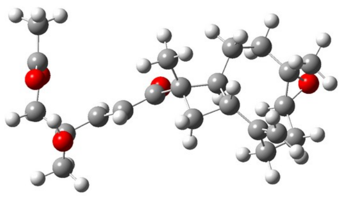	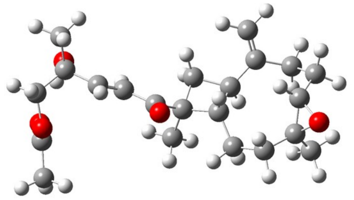	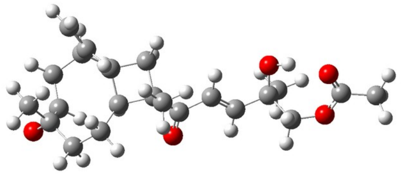
**2**-15*R*-conf. 3 (11.41%)	**2**-15*R*-conf. 4 (11.23%)	**2**-15*R*-conf. 5 (3.54%)
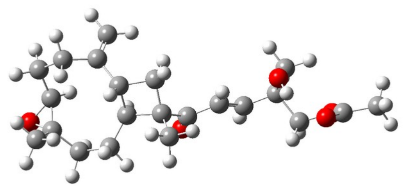	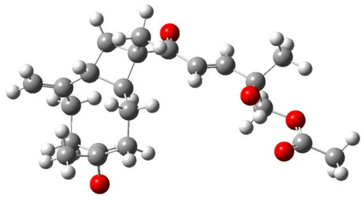	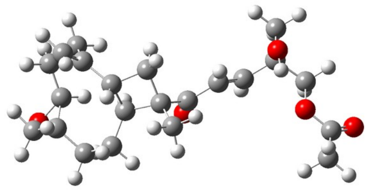
**2**-15*R*-conf. 6 (6.22%)	**2**-15*R*-conf. 8 (2.42%)	**2**-15*R*-conf. 12 (8.45%)
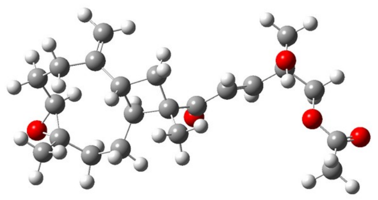	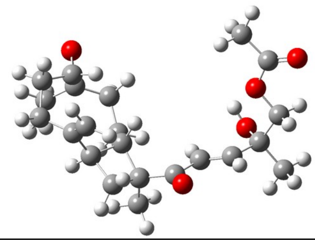	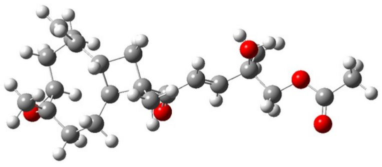
**2**-15*R*-conf. 13 (16.76%)	**2**-15*R*-conf. 15 (7.53%)	**2**-15*R*-conf. 18 (3.24%)
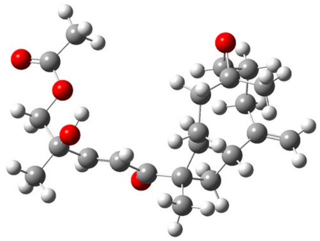	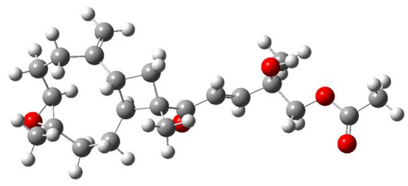	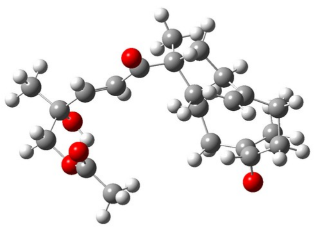
**2**-15*R*-conf. 19 (6.12%)	**2**-15*R*-conf. 20 (7.60%)	**2**-15*R*-conf. 21 (5.79%)
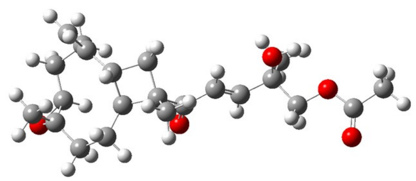	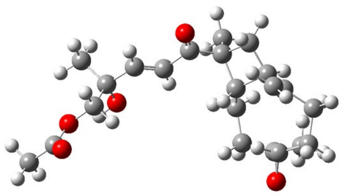	
**2**-15*R*-conf. 22 (3.34%)	**2**-15*R*-conf. 23 (3.25%)	

Figure S75. The cartesian coordinates of the dominant conformers for conformers **2**-15*R*.

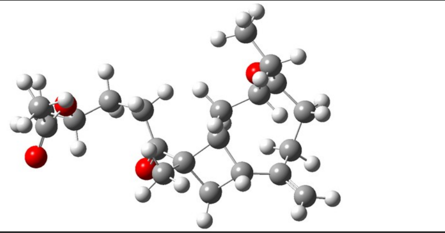	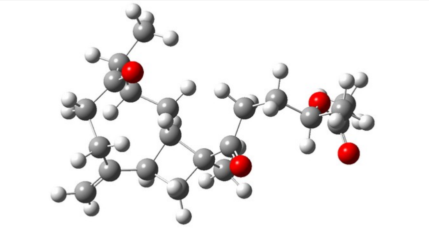	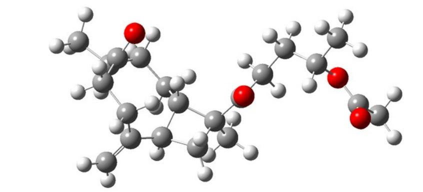
**3**-4*S*11*S*15*S*-conf. 1 (31.73%)	**3**-4*S*11*S*15*S*-conf. 4 (29.75%)	**3**-4*S*11*S*15*S*-conf. 6 (7.40%)
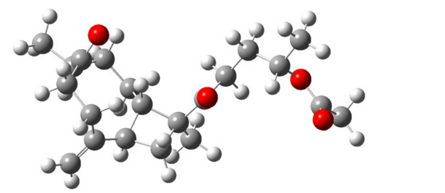	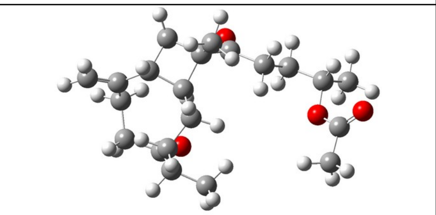	
**3**-4*S*11*S*15*S*-conf. 11 (7.23%)	**3**-4*S*11*S*15*S*-conf. 14 (20.51%)	

Figure S76. The cartesian coordinates of the dominant conformers for conformers **3**-4*S*11*S*15*S*.

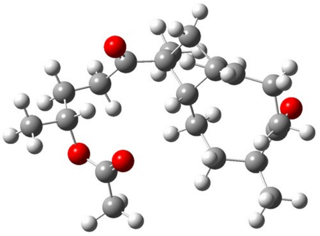	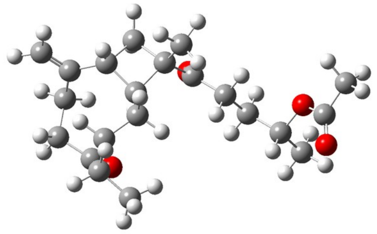	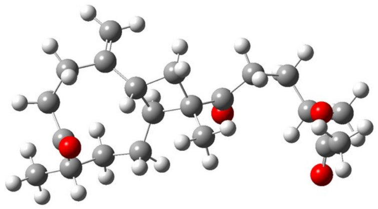
**3**-4*S*11*S*15*R*-conf. 6 (5.56%)	**3**-4*S*11*S*15*R*-conf. 8 (11.27%)	**3**-4*S*11*S*15*R*-conf. 9 (13.06%)
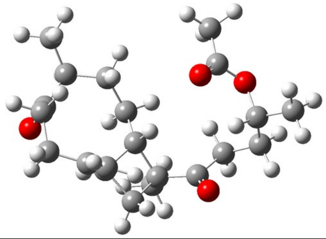	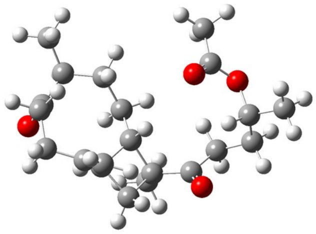	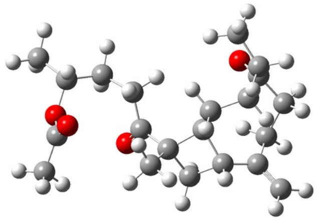
**3**-4*S*11*S*15*R*-conf. 10 (5.64%)	**3**-4*S*11*S*15*R*-conf. 11 (5.72%)	**3**-4*S*11*S*15*R*-conf. 12 (5.61%)
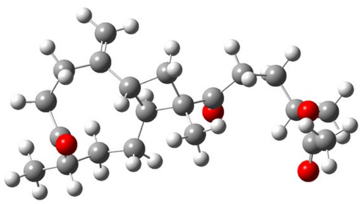	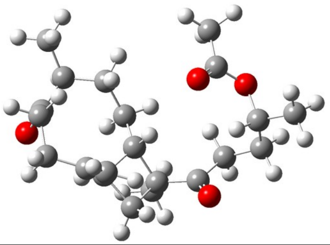	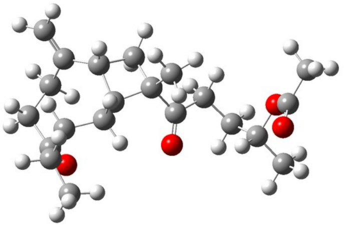
**3**-4*S*11*S*15*R*-conf. 15 (12.95%)	**3**-4*S*11*S*15*R*-conf. 16 (17.57%)	**3**-4*S*11*S*15*R*-conf. 17 (20.75%)

Figure S77. The cartesian coordinates of the dominant conformers for conformers **3**-4*S*11*S*15*R*.

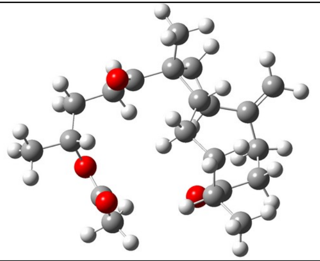	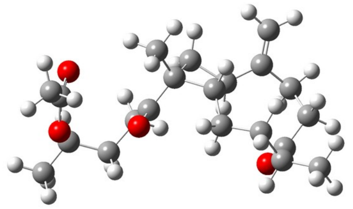	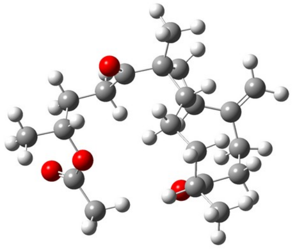
**3**-4*S*11*R*15*R*-conf. 1 (70.89%)	**3**-4*S*11*R*15*R*-conf. 2 (26.39%)	**3**-4*S*11*R*15*R*-conf. 3 (2.72%)

Figure S78. The cartesian coordinates of the dominant conformers for conformers **3**-4*S*11*R*15*R*.

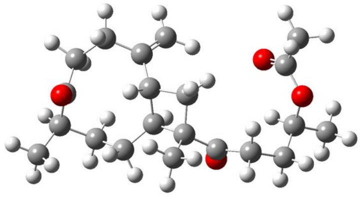	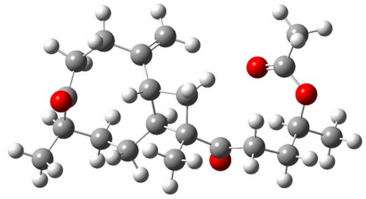	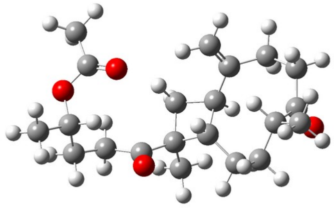
**3**-4*R*11*S*15*S*-conf. 1 (32.33%)	**3**-4*R*11*S*15*S*-conf. 2 (7.57%)	**3**-4*R*11*S*15*S*-conf. 3 (4.53%)
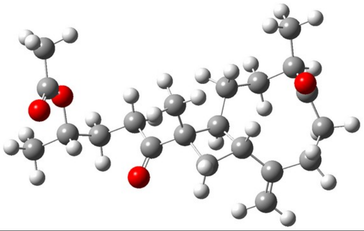	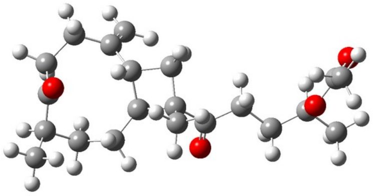	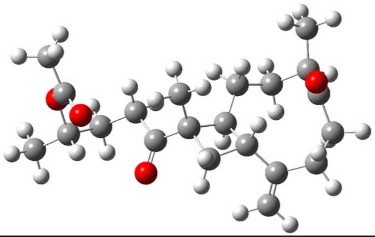
**3**-4*R*11*S*15*S*-conf. 6 (7.66%)	**3**-4*R*11*S*15*S*-conf. 7 (37.22%)	**3**-4*R*11*S*15*S*-conf. 9 (6.33%)
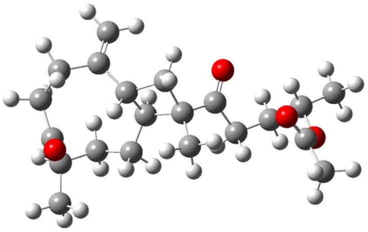		
**3**-4*R*11*S*15*S*-conf. 15 (2.04%)		

Figure S79. The cartesian coordinates of the dominant conformers for conformers **3**-4*R*11*S*15*S*.

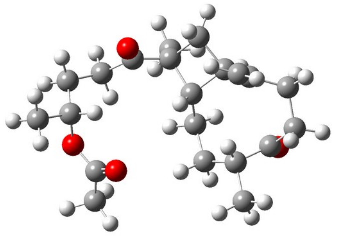	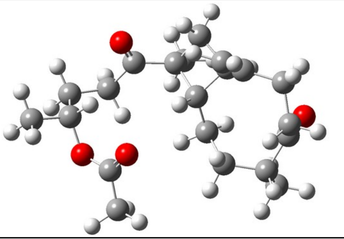	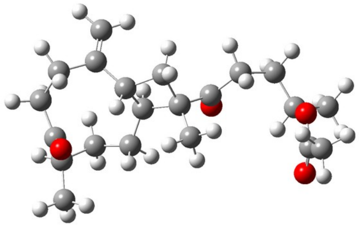
**3**-4*R*11*S*15*R*-conf. 3 (10.08%)	**3**-4*R*11*S*15*R*-conf. 4 (4.95%)	**3**-4*R*11*S*15*R*-conf. 7 (34.39%)
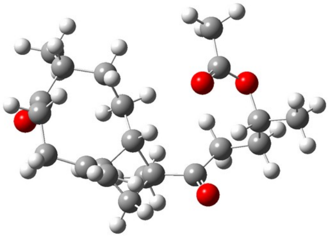	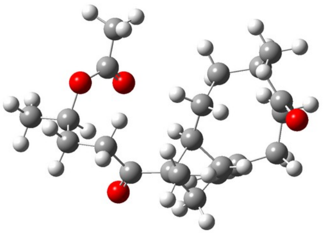	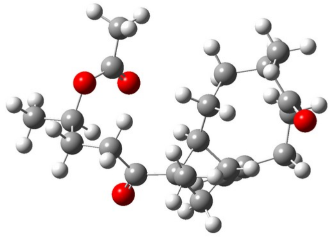
**3**-4*R*11*S*15*R*-conf. 8 (4.95%)	**3**-4*R*11*S*15*R*-conf. 9 (4.92%)	**3**-4*R*11*S*15*R*-conf. 11 (4.95%)
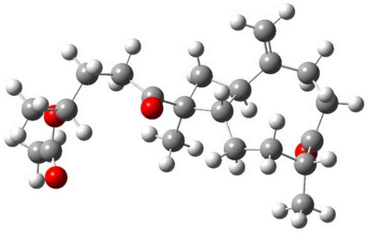		
**3**-4*R*11*S*15*R*-conf. 12 (34.42%)		

Figure S80. The cartesian coordinates of the dominant conformers for conformers **3**-4*R*11*S*15*R*.

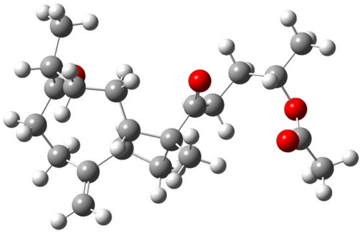	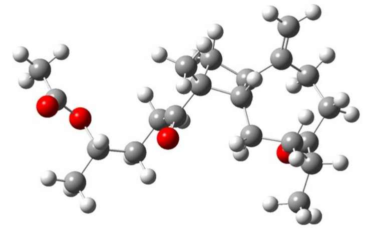	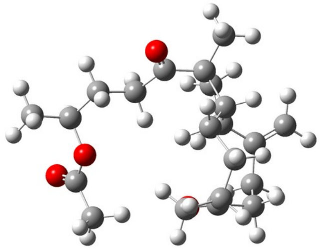
**3**-4*R*11*R*15*S*-conf. 1 (8.81%)	**3**-4*R*11*R*15*S*-conf. 2 (28.07%)	**3**-4*R*11*R*15*S*-conf. 3 (10.24%)
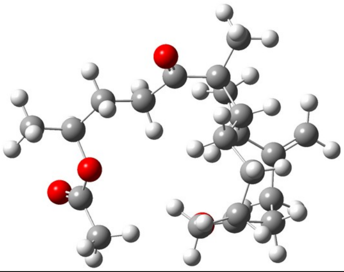	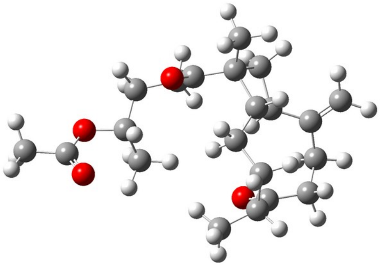	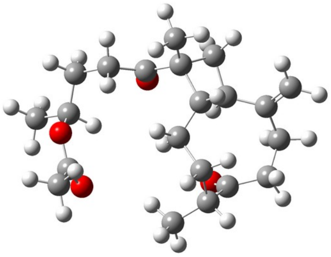
**3**-4*R*11*R*15*S*-conf. 6 (10.13%)	**3**-4*R*11*R*15*S*-conf. 7 (5.10%)	**3**-4*R*11*R*15*S*-conf. 11 (27.54%)
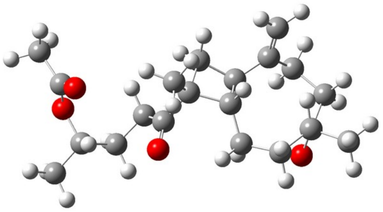		
**3**-4*R*11*R*15*S*-conf. 14 (9.62%)		

Figure S81. The cartesian coordinates of the dominant conformers for conformers **3**-4*R*11*R*15*S*.

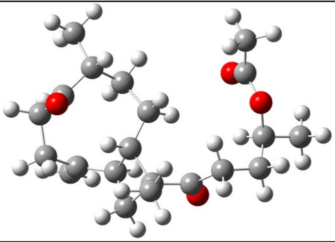	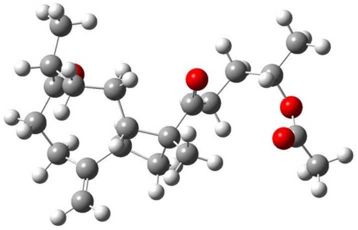	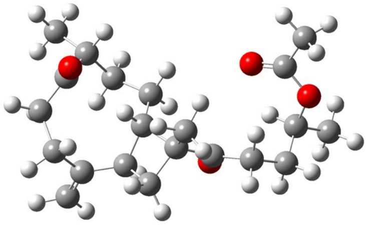
**3**-4*R*11*R*15*R*-conf. 1 (14.42%)	**3**-4*R*11*R*15*R*-conf. 2 (8.24%)	**3**-4*R*11*R*15*R*-conf. 3 (4.29%)
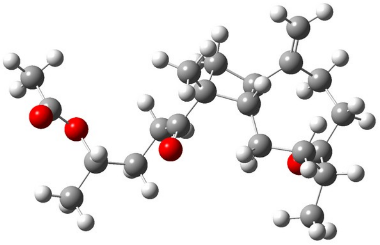	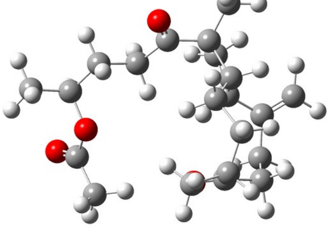	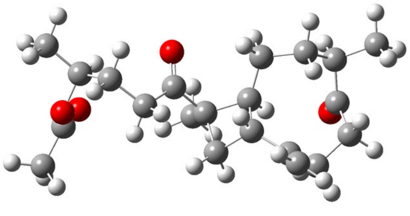
**3**-4*R*11*R*15*R*-conf. 4 (26.26%)	**3**-4*R*11*R*15*R*-conf. 5 (9.45%)	**3**-4*R*11*R*15*R*-conf. 8 (8.79%)
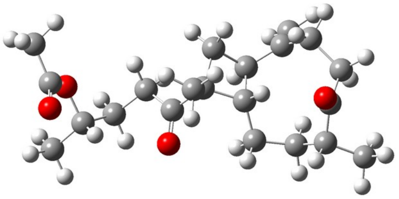	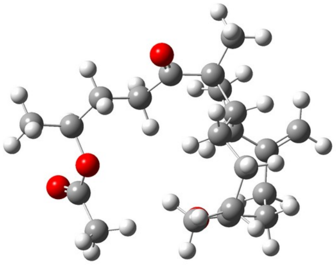	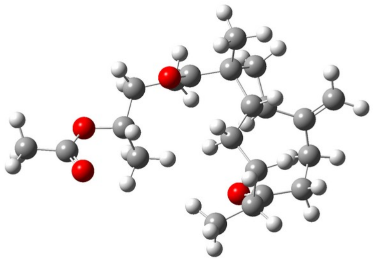
**3**-4*R*11*R*15*R*-conf. 11 (13.76%)	**3**-4*R*11*R*15*R*-conf. 13 (9.46%)	**3**-4*R*11*R*15*R*-conf. 14 (4.77%)

Figure S82. The cartesian coordinates of the dominant conformers for conformers **3**-4*R*11*R*15*R*

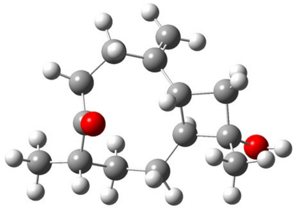	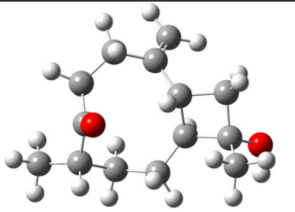	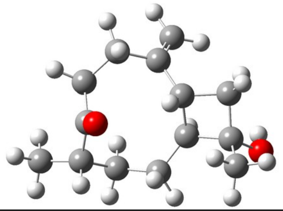
**4**-1*S*4*S*-conf. 1 (34.87%)	**4**-1*S*4*S*-conf. 2 (27.22%)	**4**-1*S*4*S*-conf. 3 (37.91%)

Figure S83. The cartesian coordinates of the dominant conformers for conformers **4**-1*S*4*S*.

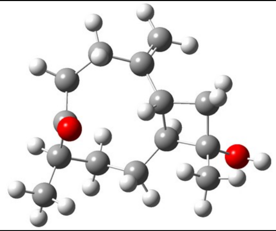	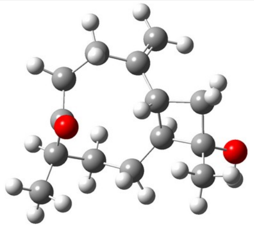	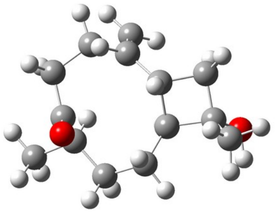
**4**-1*S*4*R*-conf. 1 (48.35%)	**4**-1*S*4*R*-conf. 2 (21.58%)	**4**-1*S*4*R*-conf. 3 (20.07%)

Figure S84. The cartesian coordinates of the dominant conformers for conformers **4**-1*S*4*R*.

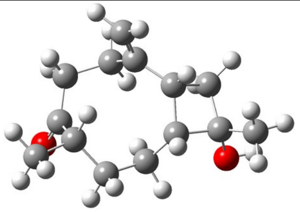	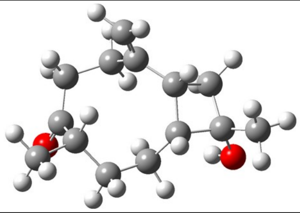	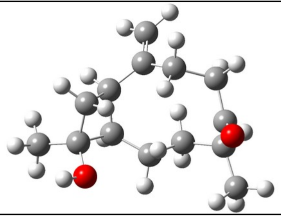
**4**-1*R*4*S*-conf. 1 (16.80%)	**4**-1*R*4*S*-conf. 2 (30.65%)	**4**-1*R*4*S*-conf. 3 (2.64%)
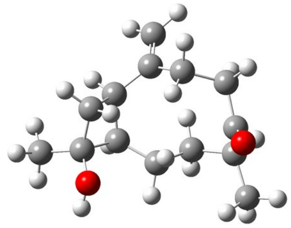	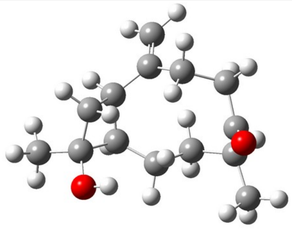	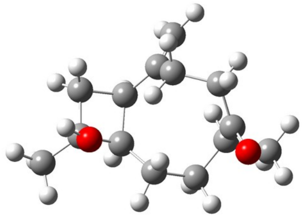
**4**-1*R*4*S*-conf. 4 (5.35%)	**4**-1*R*4*S*-conf. 5 (2.86%)	**4**-1*R*4*S*-conf. 6 (2.95%)
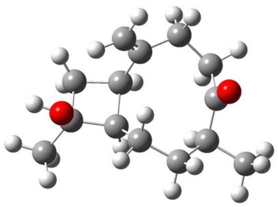	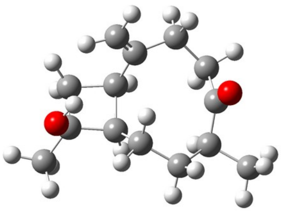	
**4**-1*R*4*S*-conf. 7 (26.88%)	**4**-1*R*4*S*-conf. 9 (11.83%)	

Figure S85. The cartesian coordinates of the dominant conformers for conformers **4**-1*R*4*S*.

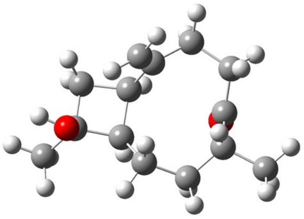	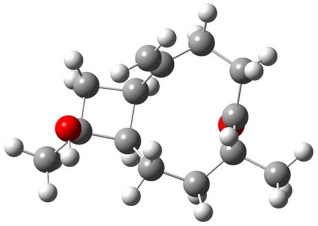	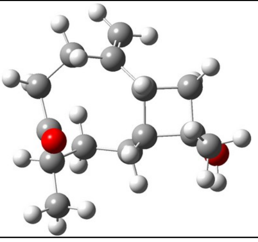
**4**-1*R*4*R*-conf. 1 (2.44%)	**4**-1*R*4*R*-conf. 2 (4.53%)	**4**-1*R*4*R*-conf. 3 (11.54%)
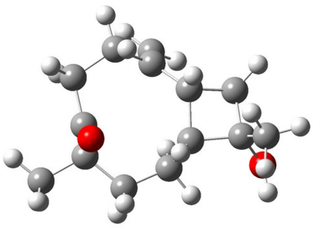	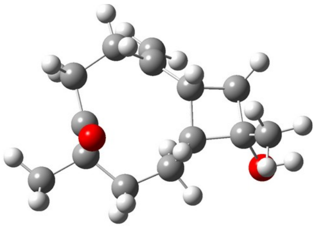	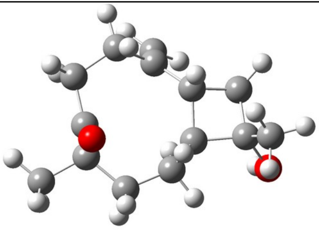
**4**-1*R*4*R*-conf. 4 (26.39%)	**4**-1*R*4*R*-conf. 5 (8.64%)	**4**-1*R*4*R*-conf. 6 (18.12%)
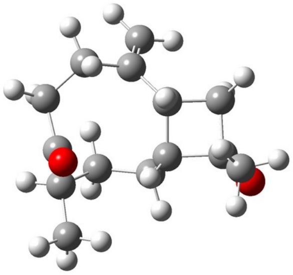	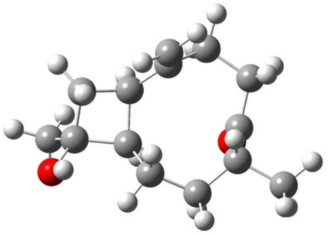	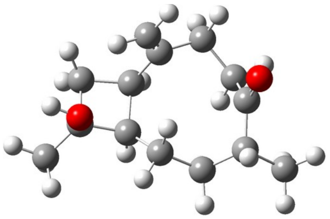
**4**-1*R*4*R*-conf. 7 (7.37%)	**4**-1*R*4*R*-conf. 8 (18.24%)	**4**-1*R*4*R*-conf. 9 (2.72%)

Figure S86. The cartesian coordinates of the dominant conformers for conformers **4**-1*R*4*R*.

3.4. DP4+ results of compounds 1-4

Functional	Solvent?	Basis Set	Type of Data
mPW1PW91	PCM	6-311+G(d,p)	Unscaled Shifts

		DP4+	0.00%	100.00%	0.00%	0.00%	0.00%	0.00%	0.00%	0.00%
Nuclei	sp2?	Experimental	Isomer 1	Isomer 2	Isomer 3	Isomer 4	Isomer 5	Isomer 6	Isomer 7	Isomer 8
C		46.6	37.8	49.6	59.0	59.0	53.0865	53.0916	58.9294	51.1519
C		29	25.5	26.4	27.0	27.0	23.2871	23.3071	23.3809	25.4421
C		39.6	35.6	37.5	37.9	38.0	28.2207	28.3516	26.3289	32.6481
C		61.5	59.5	61.2	58.0	57.7	61.4148	61.3704	61.142	60.4952
C		65.3	63.4	66.4	60.4	60.3	64.7035	64.675	63.9427	63.0839
C		31	29.8	27.6	27.3	27.4	27.8302	27.6545	29.516	22.9007
C		30.3	24.8	33.0	32.9	33.0	34.3718	34.2229	34.0721	26.8621
C	x	152.5	158.6	159.1	159.9	159.9	166.9288	166.8437	167.0159	156.5339
C		48.4	45.9	43.4	45.2	45.2	35.0159	35.0574	35.6994	44.2647
C		36.2	33.9	31.1	31.7	32.0	30.6629	30.6453	36.2843	33.4795
C		48.8	49.4	46.4	48.0	47.9	46.094	46.1922	48.5032	50.9377
C	x	205.8	208.57	209.11	208.49	208.90	205.7165	205.8333	209.6655	206.4169
C	x	123.7	125.07	125.29	126.60	124.66	125.8145	125.9521	126.5102	126.9781
C	x	152.8	157.79	157.87	157.94	160.48	158.588	158.6243	158.4623	157.257
C		74.3	73.43	73.53	73.39	72.86	73.4476	73.5012	73.5051	73.3973
C		70.2	66.87	66.82	66.98	67.69	66.7421	66.9447	66.9877	66.7755
C		24.3	18.27	18.19	18.22	18.48	18.2038	18.2149	18.2862	18.1854
C		17.2	11.81	13.42	19.46	19.62	14.6128	14.7059	18.7839	19.3109
C	x	114.5	115.77	112.68	113.08	112.99	111.2505	111.0588	111.8893	114.3504
C		17.4	13.25	11.58	11.47	11.46	18.6741	18.5987	18.9564	18.1215
H		2.45	2.81	2.34	2.03	2.04	2.0915	2.0682	2.3726	2.4607
H		1.88	1.63	1.77	1.65	1.68	2.1272	2.1322	1.7345	1.1561
H		1.66	1.85	1.78	1.32	1.37	1.9067	1.8909	1.1742	1.8216
H		1.08	2.06	1.13	0.94	0.98	1.3374	1.3112	1.7202	1.4025
H		2.06	1.22	2.19	2.06	2.13	2.0565	2.0571	1.2243	1.967
H		2.95	3.0451	2.858	2.8003	2.7798	2.8927	2.8706	2.8738	2.8255
H		2.22	1.361	2.2916	1.4339	1.4305	1.1693	2.2613	1.1261	1.7126
H		1.34	2.259	1.4564	2.2939	2.2918	2.2742	1.1525	2.3411	2.0759
H		2.33	2.2344	2.7294	2.7197	1.961	2.3066	2.3071	2.5241	2.5523
H		2.18	2.4358	1.95	1.931	2.7119	2.4967	2.4918	2.2923	2.8079
H		2.83	2.9768	2.6206	2.645	2.6753	2.5592	2.5378	2.5408	2.8553
H		2.15	1.8628	2.2278	2.7111	2.725	2.8647	2.871	2.9644	2.7298
H		1.84	1.9673	2.0055	1.7474	1.7334	1.9304	1.9432	1.4613	1.5646
H		6.54	6.9716	7.0449	7.3256	7.328	6.9435	6.92	7.3691	7.2235
H		6.97	7.6384	7.657	7.6491	7.8066	7.7269	7.7335	7.685	7.6513
H		3.44	3.4331	3.4338	3.9789	3.7471	3.9194	3.9578	3.4437	3.9551
H		1.28	1.2805	1.2753	1.2794	1.3201	1.2675	1.2701	1.2755	1.2888
H		1.33	1.3941	1.3881	1.4288	1.3118	1.4209	1.4425	1.4042	1.2553
H		4.93	5.2541	5.4904	5.7137	5.7037	5.2118	5.1922	5.467	5.2562
H		5.02	5.4211	5.6147	5.6712	5.6724	5.4056	5.3504	5.5618	5.144
H		1.22	1.2623	1.3911	1.2926	1.3148	1.5693	1.5623	1.316	1.3536

Functional	Solvent?	Basis Set	Type of Data
mPW1PW91	PCM	6-311+G(d,p)	Unscaled Shifts

	Isomer 1	Isomer 2	Isomer 3	Isomer 4	Isomer 5	Isomer 6	Isomer 7	Isomer 8
sDP4+ (H data)	0.00%	99.92%	0.00%	0.00%	0.00%	0.08%	0.00%	0.00%
sDP4+ (C data)	99.94%	0.06%	0.00%	0.00%	0.00%	0.00%	0.00%	0.00%
sDP4+ (all data)	1.80%	98.20%	0.00%	0.00%	0.00%	0.00%	0.00%	0.00%
uDP4+ (H data)	0.00%	99.93%	0.00%	0.00%	0.00%	0.07%	0.00%	0.00%
uDP4+ (C data)	0.00%	65.77%	1.66%	1.84%	0.00%	0.00%	0.06%	30.67%
uDP4+ (all data)	0.00%	100.00%	0.00%	0.00%	0.00%	0.00%	0.00%	0.00%
DP4+ (H data)	0.00%	100.00%	0.00%	0.00%	0.00%	0.00%	0.00%	0.00%
DP4+ (C data)	1.13%	98.87%	0.00%	0.00%	0.00%	0.00%	0.00%	0.00%
DP4+ (all data)	0.00%	100.00%	0.00%	0.00%	0.00%	0.00%	0.00%	0.00%

Figure S87. DP4+ results obtained using experimental data of **1** *versus* isomers 1-8.

Functional	Solvent?	Basis Set	Type of Data
mPW1PW91	PCM	6-311+G(d,p)	Unscaled Shifts

		DP4+	0.00%	100.00%	-	-	-
Nuclei	sp2?	Experimental	Isomer 1	Isomer 2	Isomer 3	Isomer 4	Isomer 5
C		46.6	41.8	49.7			
C		29	26.9	26.5			
C		39.7	37.6	37.5			
C		61.5	57.9	58.3			
C		65.3	60.4	60.4			
C		31	30.4	27.6			
C		30.3	33.1	33.0			
C	x	152.5	159.6	159.2			
C		48.3	44.1	43.4			
C		36.2	28.6	31.2			
C		48.8	46.2	46.4			
C	x	205.5	207.27	209.24			
C	x	124.2	125.27	123.54			
C	x	151.2	156.31	159.07			
C		72.7	74.50	72.28			
C		71.1	70.76	70.17			
C		24.7	19.70	18.73			
C		17.2	13.21	13.47			
C	x	114.6	113.39	112.60			
C		17.4	11.67	11.54			
C	x	172.4	182.05	175.74			
C		20.7	17.67	17.24			
H		2.45	1.92	2.33			
H		1.86	2.13	1.79			
H		1.66	2.02	1.79			
H		1.07	2.2869	1.1354			
H		2.08	1.0761	2.1905			
H		2.94	2.7098	2.8652			
H		2.22	1.4347	2.3036			
H		1.34	2.2815	1.4629			
H		2.33	2.0051	2.7439			
H		2.18	2.7234	1.9527			
H		2.83	2.6271	2.6556			
H		2.14	2.834	2.254			
H		1.84	1.6951	2.0176			
H		6.54	7.1851	7.0628			
H		6.9	7.2003	7.7899			
H		4.01	4.4689	4.0336			
H		4.07	4.0217	4.2014			
H		1.32	1.3716	1.249			
H		1.33	1.5226	1.446			
H		4.94	4.9702	5.4745			
H		5.03	5.0632	5.5905			
H		1.23	1.4248	1.3996			
H		2.05	2.0374	2.2395			

Functional	Solvent?	Basis Set	Type of Data
mPW1PW91	PCM	6-311+G(d,p)	Unscaled Shifts

	Isomer 1	Isomer 2	Isomer 3	Isomer 4	Isomer 5	Isomer 6
sDP4+ (H data)	0.00%	100.00%	-	-	-	-
sDP4+ (C data)	1.81%	98.19%	-	-	-	-
sDP4+ (all data)	0.00%	100.00%	-	-	-	-
uDP4+ (H data)	0.00%	100.00%	-	-	-	-
uDP4+ (C data)	39.17%	60.83%	-	-	-	-
uDP4+ (all data)	0.00%	100.00%	-	-	-	-
DP4+ (H data)	0.00%	100.00%	-	-	-	-
DP4+ (C data)	1.18%	98.82%	-	-	-	-
DP4+ (all data)	0.00%	100.00%	-	-	-	-

Figure S88. DP4+ results obtained using experimental data of **2** *versus* isomers 1 and 2.

Functional	Solvent?	Basis Set	Type of Data
mPW1PW91	PCM	6-311+G(d,p)	Unscaled Shifts

		DP4+	0.00%	100.00%	0.00%	0.00%	0.00%	0.00%	0.00%	0.00%
Nuclei	sp2?	Experimental	Isomer 1	Isomer 2	Isomer 3	Isomer 4	Isomer 5	Isomer 6	Isomer 7	Isomer 8
C		47.5	39.5	49.8	46.5	59.9	38.7048	54.034	60.2374	60.2375
C		28.5	22.0	23.9	22.6	26.9	22.687	20.2571	21.884	21.8839
C		31.3	24.0	27.8	23.5	29.8	24.3955	26.6833	27.0105	27.0104
C		48.5	46.6	48.8	46.3	51.7	46.6172	47.0018	46.9856	46.9856
C	x	219.8	229.8	224.2	229.4	226.4	229.0668	224.4212	224.4482	224.4478
C		43.7	43.3	43.6	43.2	35.4	42.5446	40.5352	40.6126	40.6125
C		32.7	29.9	34.4	29.8	34.3	29.9202	34.3336	34.254	34.254
C	x	153.2	161.3	162.8	161.5	163.4	160.8981	162.8105	162.8985	162.8983
C		43.5	39.5	39.6	41.0	38.8	39.88	36.6394	39.7912	39.7914
C		34.7	39.5	35.6	41.0	38.8	39.88	36.6394	39.7912	39.7914
C		48.6	49.1	49.3	50.3	49.1	49.0335	47.2472	49.4369	49.4369
C	x	215.6	224.87	221.74	223.27	224.36	225.5938	226.7678	224.134	224.1336
C		33.5	30.15	31.78	34.81	35.47	29.2665	29.7303	35.284	35.2839
C		30.6	25.36	26.46	25.96	26.16	25.2676	25.216	26.0291	26.0291
C		71.6	68.69	70.52	68.57	68.23	68.6231	68.6059	69.0571	69.0571
C		20.3	15.80	17.85	16.10	16.09	15.8356	15.6748	15.6636	15.6636
C		17.2	13.03	14.54	19.38	21.82	13.0635	14.222	22.3307	22.3306
C		113	113.82	113.63	113.86	111.76	113.9762	111.5611	111.9032	111.903
C		16.7	8.57	9.40	8.21	12.99	8.4109	9.3932	9.4807	9.4808
C	x	172.7	176.09	175.89	175.98	176.77	176.1226	176.068	176.1004	176.1004
C		21.2	18.09	18.07	18.07	19.60	18.0826	18.0858	18.0671	18.0671
H		1.94	2.04	2.01	2.03	1.86	2.0793	2.0905	1.8409	1.8409
H		1.76	1.54	1.73	1.61	0.63	1.8372	1.6328	1.5631	1.5631
H		1.44	1.92	1.43	0.79	1.70	1.3537	1.3756	0.6397	0.6397
H		1.68	2.34	1.72	1.57	1.81	1.7444	2.0352	1.8833	1.8833
H		1.89	1.762	1.9113	2.1631	1.4118	2.2969	1.9265	1.8933	1.8933
H		2.63	2.764	2.7592	2.6961	2.4681	2.7069	2.9384	2.8938	2.8938
H		2.46	2.564	2.4713	2.7408	2.1794	2.536	2.9545	2.9535	2.9535
H		2.63	2.7649	2.6238	2.5595	2.8846	2.7762	2.4146	2.4184	2.4184
H		2.46	2.4995	2.5187	2.6045	2.975	2.479	2.9265	2.929	2.929
H		2.54	2.7118	2.6583	2.8996	2.2692	2.6971	2.1497	2.1162	2.1162
H		1.67	1.9111	1.8386	2.4881	2.7181	1.8021	2.0475	2.7354	2.7354
H		2.03	1.687	2.0529	1.3081	1.8955	1.9616	2.0011	1.8524	1.8524
H		2.41	2.4785	2.4938	2.6623	3.0726	2.4167	2.5447	3.0342	3.0342
H		2.5	2.9319	2.5762	2.694	2.7923	2.9382	2.9662	2.7858	2.7858
H		1.73	2.1441	1.7784	2.0846	2.0486	1.6015	2.1489	2.0522	1.6459
H		1.82	1.6612	1.9321	1.6061	1.6585	2.1286	1.6206	1.6459	2.0523
H		4.87	4.8	4.9117	5.1319	5.1744	4.8069	4.7672	4.9927	4.9927
H		1.22	1.1845	1.1969	1.1381	1.2296	1.1993	1.1577	1.2399	1.2399
H		1.21	1.1235	1.2567	1.219	1.0315	1.1572	1.3787	1.0645	1.0645
H		4.93	5.2936	5.1556	5.2826	5.5583	5.287	5.4526	5.532	5.532
H		1.03	1.127	1.0902	1.1019	1.0622	1.2103	0.9739	0.9221	0.922
H		2.03	2.2627	2.2121	2.2295	2.1381	2.2555	2.2693	2.2565	2.2565

Functional	Solvent?	Basis Set	Type of Data
mPW1PW91	PCM	6-311+G(d,p)	Unscaled Shifts

	Isomer 1	Isomer 2	Isomer 3	Isomer 4	Isomer 5	Isomer 6	Isomer 7	Isomer 8
sDP4+ (H data)	0.00%	100.00%	0.00%	0.00%	0.00%	0.00%	0.00%	0.00%
sDP4+ (C data)	8.93%	70.52%	0.00%	0.00%	20.55%	0.00%	0.00%	0.00%
sDP4+ (all data)	0.00%	100.00%	0.00%	0.00%	0.00%	0.00%	0.00%	0.00%
uDP4+ (H data)	0.00%	0.00%	0.00%	100.00%	0.00%	0.00%	0.00%	0.00%
uDP4+ (C data)	0.00%	2.06%	0.00%	97.94%	0.00%	0.00%	0.00%	0.00%
uDP4+ (all data)	0.00%	0.00%	0.00%	100.00%	0.00%	0.00%	0.00%	0.00%
DP4+ (H data)	0.00%	96.40%	0.00%	3.60%	0.00%	0.00%	0.00%	0.00%
DP4+ (C data)	0.00%	100.00%	0.00%	0.00%	0.00%	0.00%	0.00%	0.00%
DP4+ (all data)	0.00%	100.00%	0.00%	0.00%	0.00%	0.00%	0.00%	0.00%

Figure S89. DP4+ results obtained using experimental data of **3** *versus* isomers 1-8.

Functional	Solvent?	Basis Set	Type of Data
mPW1PW91	PCM	6-311+G(d,p)	Unscaled Shifts

		DP4+	100.00%	0.00%	0.00%	0.00%	-
**Nuclei**	**sp2?**	**Experimental**	**Isomer 1**	**Isomer 2**	**Isomer 3**	**Isomer 4**	**Isomer 5**
C		56.6	56.8	58.2	55.2	51.7	
C		27.8	25.6	18.7	18.7	20.4	
C		32.4	32.4	26.9	34.2	32.3	
C		48.7	49.0	47.0	42.5	49.6	
C	x	219.5	223.1	224.1	229.5	229.4	
C		43.6	43.8	40.8	47.2	42.9	
C		32.7	34.4	34.3	24.2	36.5	
C	x	153.1	158.2	163.0	157.8	153.1	
C		41.2	38.9	34.5	38.0	37.4	
C		41.8	40.9	39.7	40.1	32.7	
C		71.2	70.5	68.8	69.3	67.6	
C		21.5	15.50	17.93	21.60	23.23	
C	x	112.5	111.10	111.33	116.80	115.90	
C		17.3	12.91	9.48	14.89	15.53	
H		1.66	1.61	1.89	2.40	2.06	
H		1.28	1.21	1.56	1.36	1.76	
H		1.81	1.87	1.23	1.65	1.41	
H		1.66	1.54	2.02	1.25	1.47	
H		1.82	1.87	1.92	2.02	1.83	
H		2.61	2.43	2.95	3.22	2.81	
H		2.48	2.25	2.97	3.08	3.05	
H		2.65	2.94	2.44	2.52	2.34	
H		2.51	2.95	2.94	2.32	2.26	
H		2.44	2.41	2.38	2.63	2.92	
H		1.94	1.68	1.68	3.03	2.51	
H		1.82	1.7908	1.8811	2.1085	2.0498	
H		1.89	2.1333	2.0183	2.2081	1.7487	
H		1.13	1.1034	1.1659	1.3062	1.1374	
H		4.93	5.3258	5.3894	5.6011	5.7913	
H		4.94	5.4809	5.5242	5.6978	5.4631	
H		1.02	1.1366	0.9606	1.0132	1.0396	

Functional	Solvent?	Basis Set	Type of Data
mPW1PW91	PCM	6-311+G(d,p)	Unscaled Shifts

	Isomer 1	Isomer 2	Isomer 3	Isomer 4	Isomer 5	Isomer 6
sDP4+ (H data)	99.48%	0.51%	0.01%	0.00%	-	-
sDP4+ (C data)	100.00%	0.00%	0.00%	0.00%	-	-
sDP4+ (all data)	100.00%	0.00%	0.00%	0.00%	-	-
uDP4+ (H data)	100.00%	0.00%	0.00%	0.00%	-	-
uDP4+ (C data)	99.86%	0.00%	0.11%	0.03%	-	-
uDP4+ (all data)	100.00%	0.00%	0.00%	0.00%	-	-
DP4+ (H data)	100.00%	0.00%	0.00%	0.00%	-	-
DP4+ (C data)	100.00%	0.00%	0.00%	0.00%	-	-
DP4+ (all data)	100.00%	0.00%	0.00%	0.00%	-	-

Figure S90. DP4+ results obtained using experimental data of **4** *versus* isomers 1-4.

3.5. Correlation plots of compounds 1-4

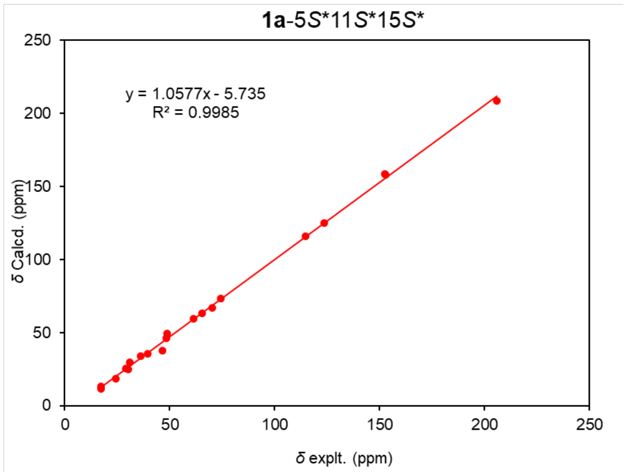

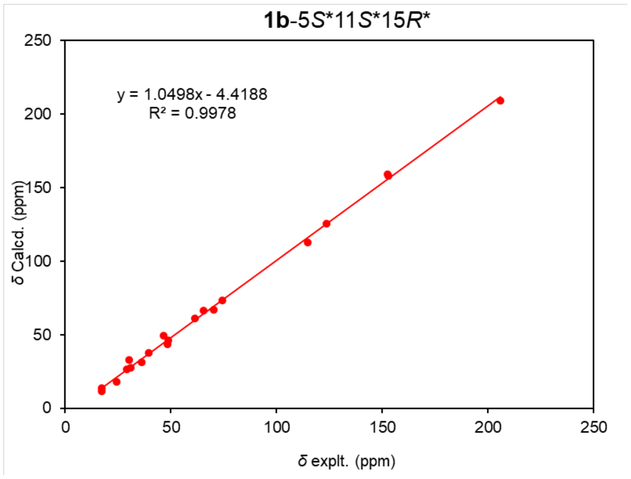

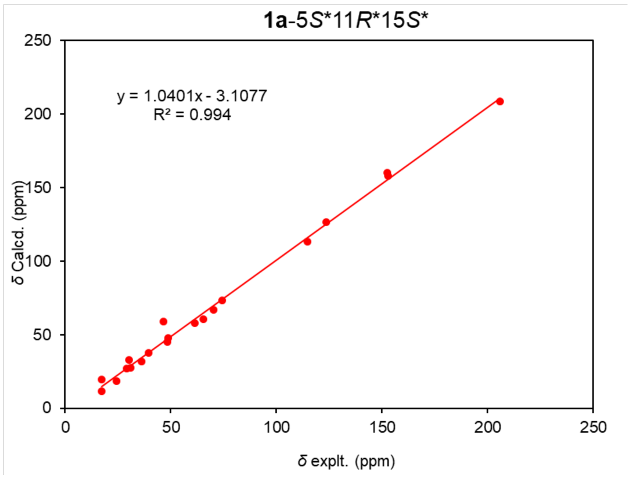

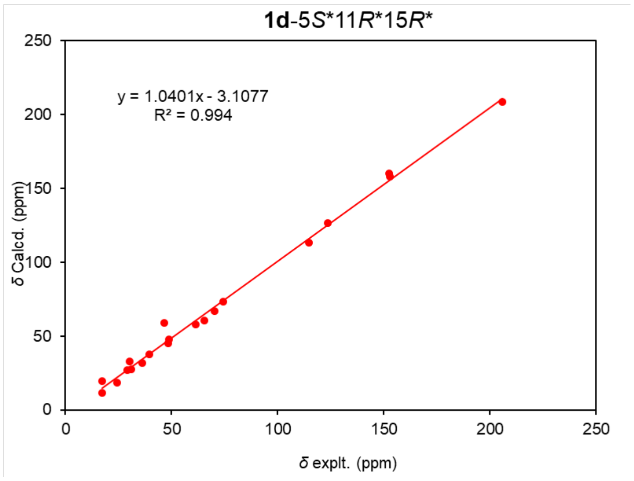

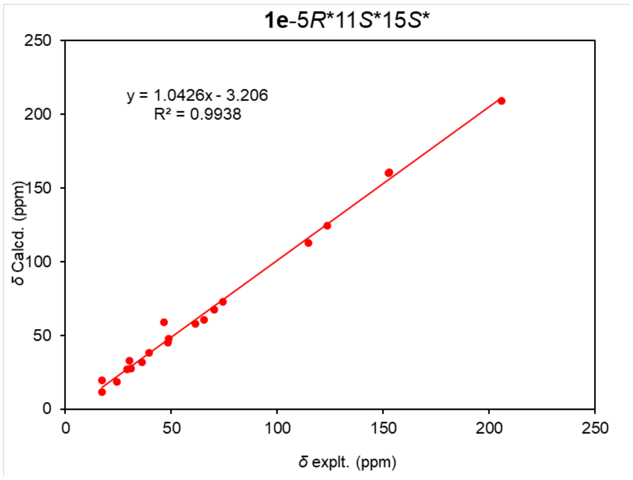

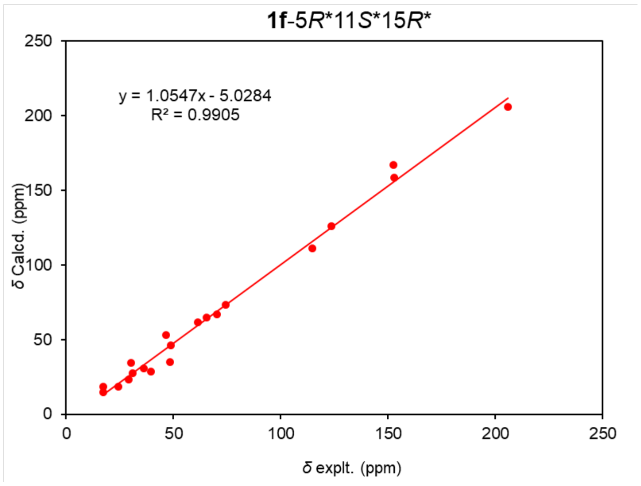

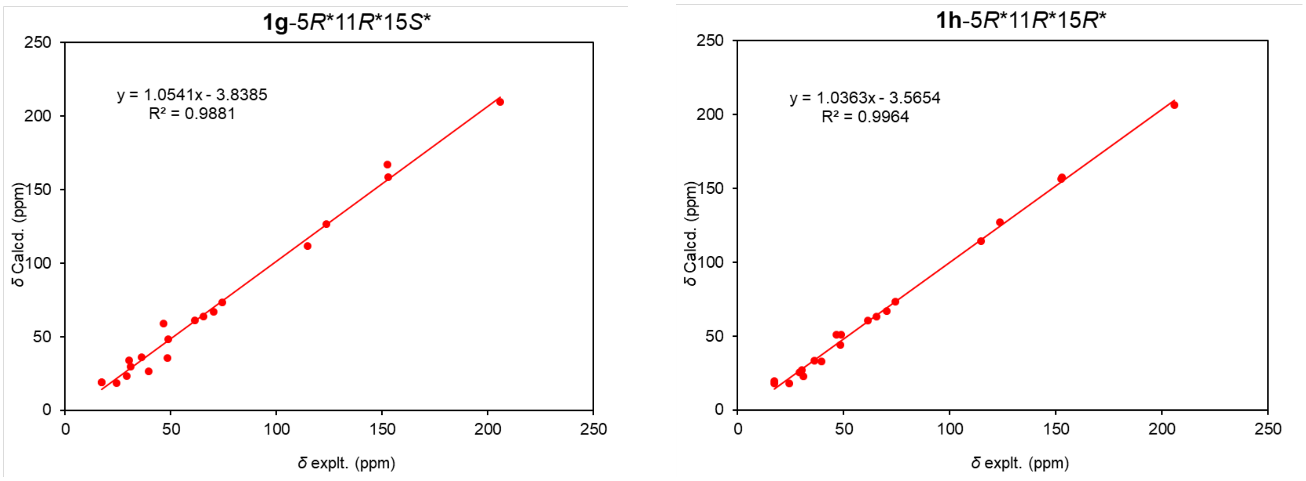

Figure S91. Linear correlations of the calculated isomers of **1** with the experimentally observed ^{13}C NMR chemical shifts.

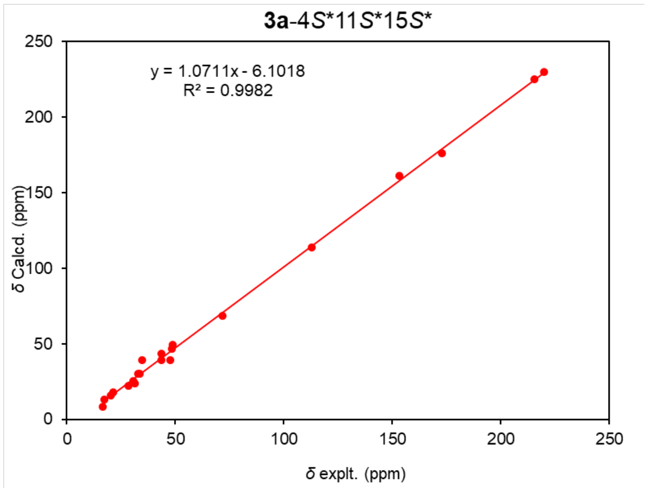
3a-4*S**11*S**15*S**
y = 1.0711x - 6.1018
R² = 0.9982
δ Calcd. (ppm)
δ explt. (ppm)

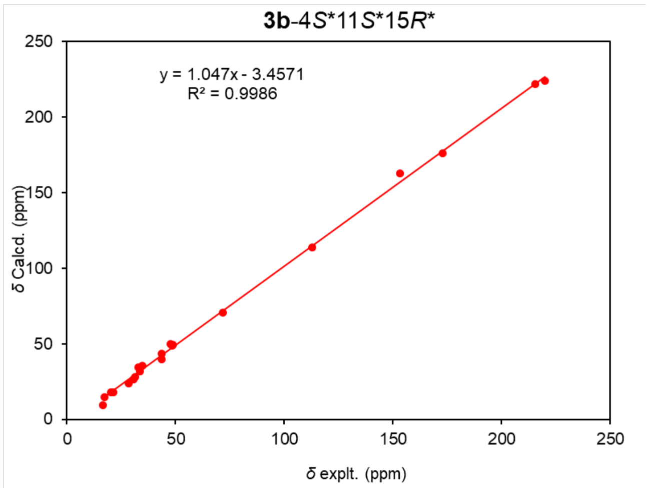
3b-4*S**11*S**15*R**
y = 1.047x - 3.4571
R² = 0.9986
δ Calcd. (ppm)
δ explt. (ppm)

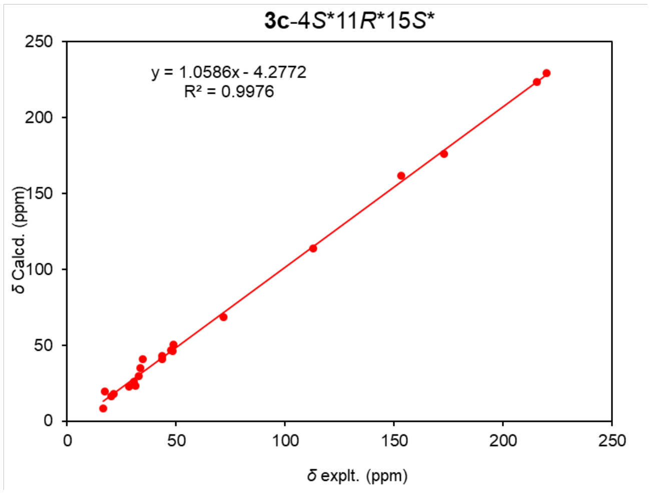
3c-4*S**11*R**15*S**
y = 1.0586x - 4.2772
R² = 0.9976
δ Calcd. (ppm)
δ explt. (ppm)

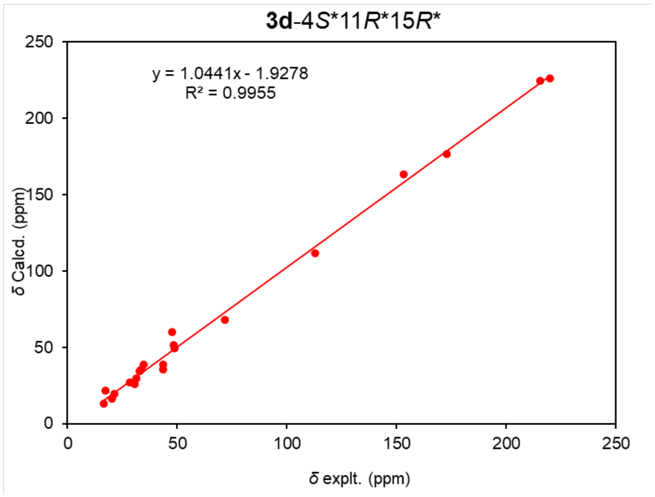
3d-4*S**11*R**15*R**
y = 1.0441x - 1.9278
R² = 0.9955
δ Calcd. (ppm)
δ explt. (ppm)

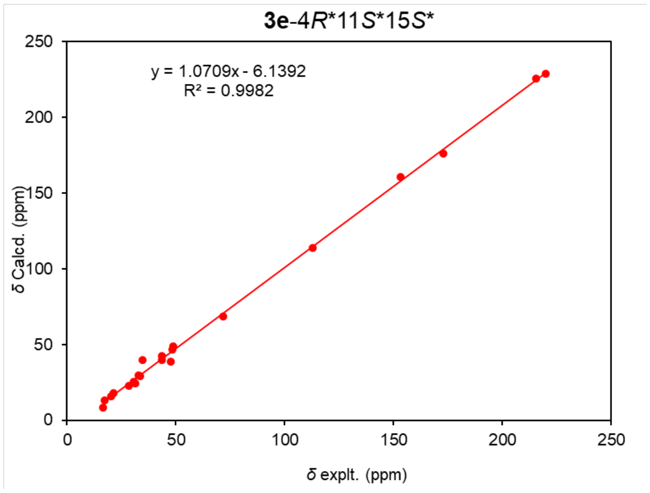
3e-4*R**11*S**15*S**
y = 1.0709x - 6.1392
R² = 0.9982
δ Calcd. (ppm)
δ explt. (ppm)

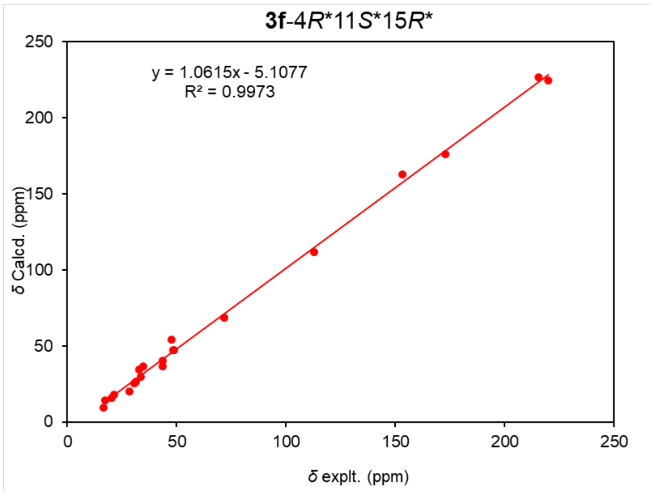
3f-4*R**11*S**15*R**
y = 1.0615x - 5.1077
R² = 0.9973
δ Calcd. (ppm)
δ explt. (ppm)

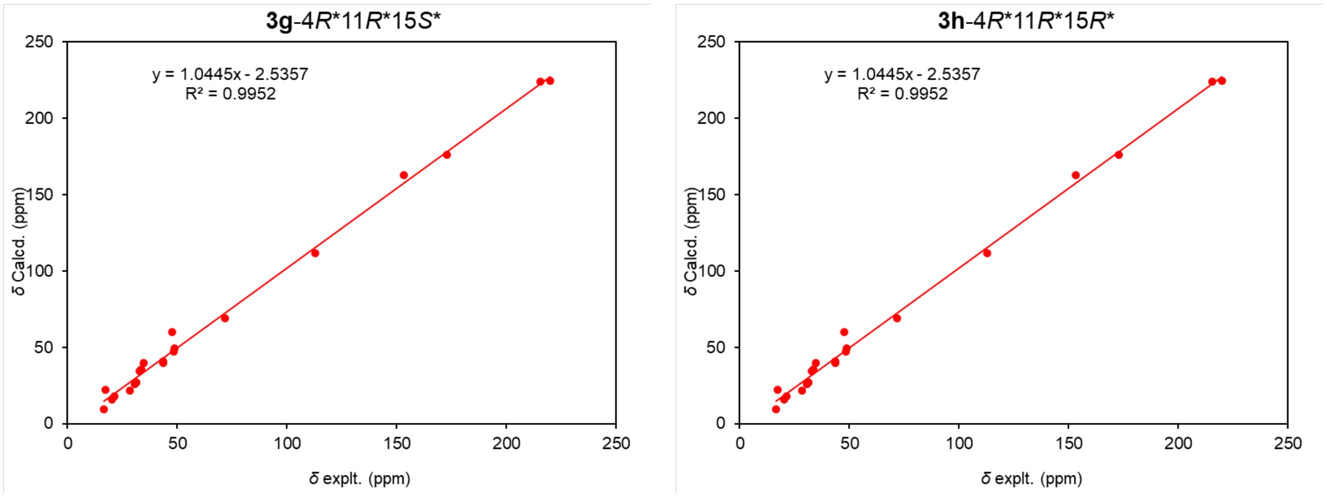

Figure S92. Linear correlations of the calculated isomers of **3** with the experimentally observed ^{13}C NMR chemical shifts.

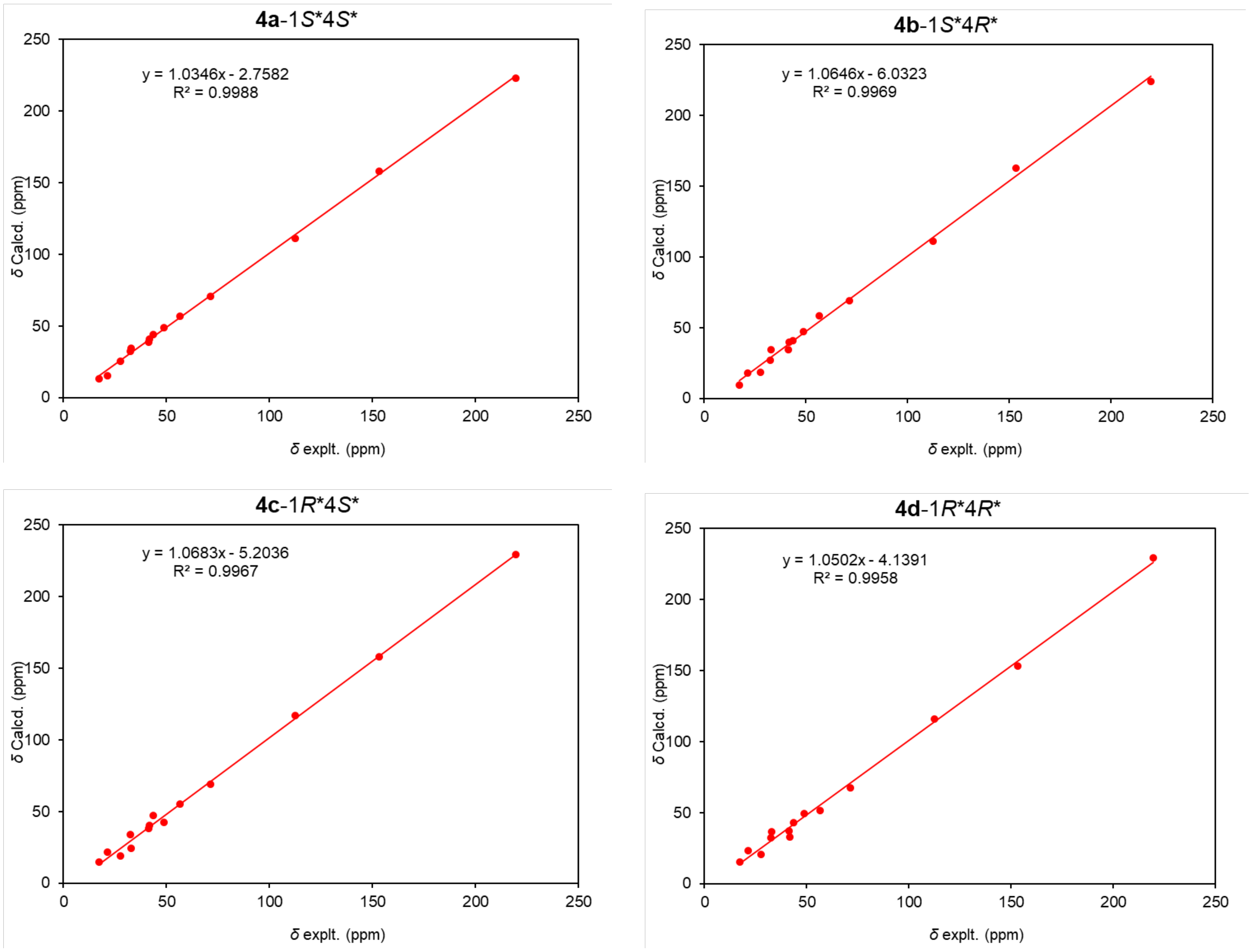

Figure S93. Linear correlations of the calculated isomers of **4** with the experimentally observed ^{13}C NMR chemical shifts.

4. Michaelis-Menten plots of enzyme activity

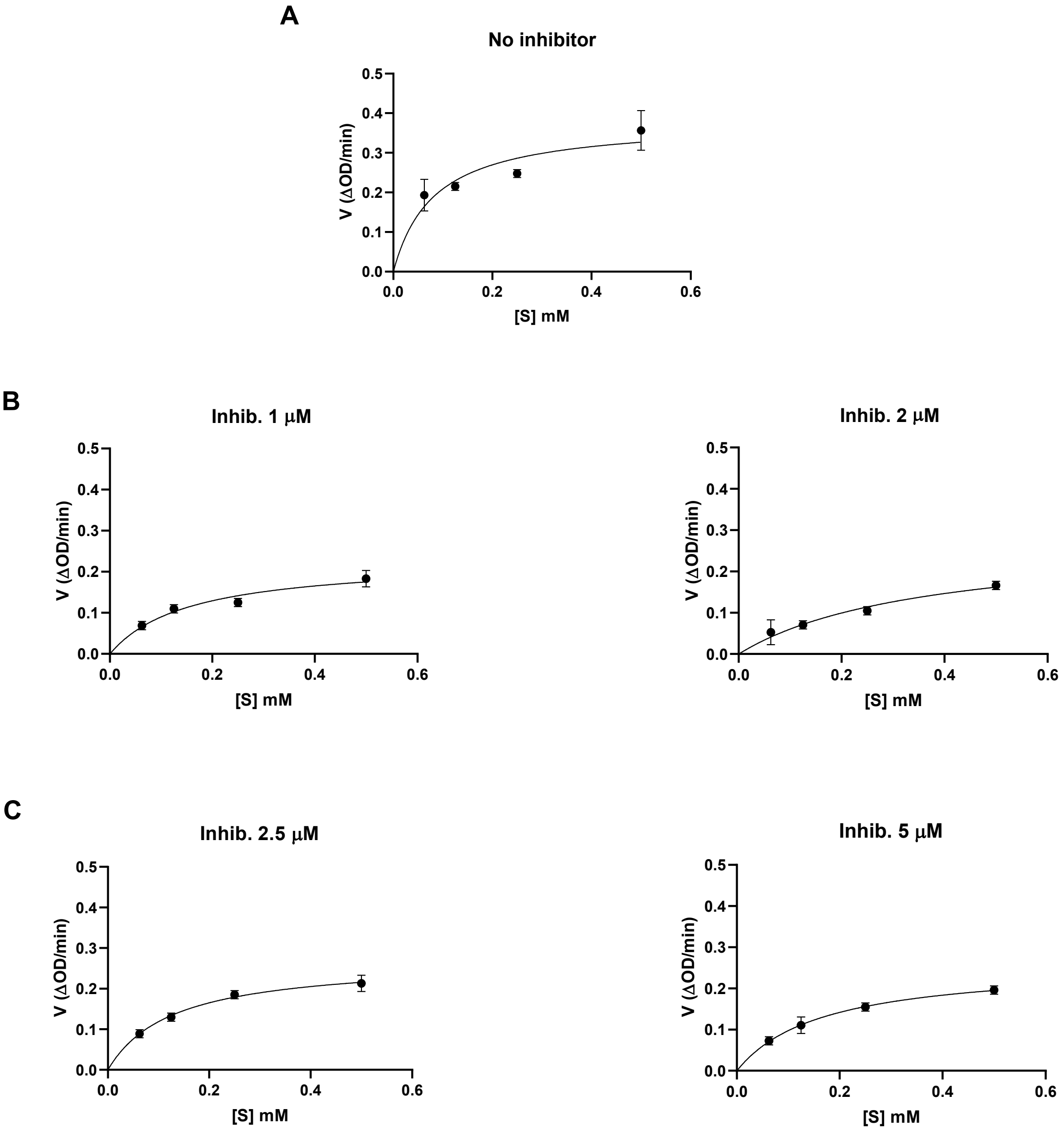

Figure S94. Michaelis-Menten plots of enzyme activity in the absence and presence of inhibitor at different concentrations. (**A**) Michaelis–Menten plot obtained in the absence of inhibitor. (**B**) Michaelis–Menten plots in the presence of inhibitor at concentrations of 1 and 2 μM of compound **1**. (**C**) Michaelis–Menten plots in the presence of inhibitor at concentrations of 2.5 and 5 μM of compound **2**.
